# Ventilatory Parameters and Adjunctive Therapies Associated with ICU Mortality in Severe Pneumonia: A Large Retrospective Cohort Study

**DOI:** 10.3390/jcm15187171

**Published:** 2026-09-15

**Authors:** Weishan Nie, Jiulin Guo, Ningchang Tang, Zongan Liang, Yongfang Zhou

**Affiliations:** 1 Department of Respiratory Care, West China Hospital, Sichuan University, Chengdu 610041, China; 2020324020148@stu.scu.edu.cn (W.N.); keroppi_tt@163.com (N.T.); 2 Information Center, West China Hospital, Sichuan University, Chengdu 610041, China; gjlper90@wschscu.cn; 3 Department of Respiratory and Critical Care Medicine, West China Hospital, Sichuan University, Chengdu 610041, China

**Keywords:** severe pneumonia, mechanical ventilation, ICU mortality, PEEP, respiratory rate, driving pressure, tidal volume, time-dependent Cox regression

## Abstract

**Background:** Severe pneumonia (SP) comprises clinically diverse infections with variable lung mechanics, host responses, and recruitability. Yet ventilatory settings and adjunctive therapies are frequently guided by ARDS-derived evidence. We aimed to examine the associations of serial ventilatory parameters, gas-exchange indices, and selected adjunctive therapies with ICU mortality among patients with SP receiving invasive mechanical ventilation. **Methods:** This single-center retrospective cohort study included 2480 adults with microbiologically confirmed SP who received invasive mechanical ventilation in the ICU from 2015 to 2025. Daily ventilatory, gas-exchange, vital-sign, laboratory, and management variables were collected during the early ventilation course. Multivariable time-dependent Cox regression models were stratified by ventilation mode and adjusted for demographics, non-pulmonary SOFA score, comorbidities, pathogen type, shock, gas exchange, inflammatory markers, organ dysfunction, and time. Exploratory subgroup analyses were performed according to viral or fungal infection, hypoxemia severity, and immunocompromised status. **Results:** ICU mortality was 38.23%. In primary imputed models, higher PEEP, systemic glucocorticoid use, and neuromuscular blocker use were each associated with higher ICU mortality (HR 1.04, 1.40–1.41, and 1.53–1.56, respectively; all *p* < 0.001). Complete-case sensitivity analyses attenuated the PEEP association, indicating sensitivity to missing-data handling. V_T_/kg and respiratory rate showed significant U/J-shaped non-linear associations with mortality (*p* = 0.005 and *p* = 0.027), with the lowest mortality risk at VT/kg 6–8 mL/kg and respiratory rate 18–26 breaths/min. Driving pressure, ventilatory ratio, minute ventilation, and [(4 × ΔP) + RR] were not significantly associated with mortality in linear models, although [(4 × ΔP) + RR] showed a non-linear association in sensitivity analyses. In severe hypoxemia, associations for PEEP, glucocorticoids, and neuromuscular blockers were not statistically significant. **Conclusions:** In this large SP cohort, ventilatory settings were generally within protective ranges. Mortality associations varied by parameter and subgroup, suggesting that physiology-guided ventilation and individualized PEEP titration may be reasonable approaches in light of the observed U-shaped (VT/kg) and J-shaped (respiratory rate) non-linear relationships, as well as a non-linear pattern for [(4 × ΔP) + RR], while adjunctive therapy signals require cautious interpretation given residual confounding.

## 1. Background

Severe pneumonia (SP) is a life-threatening lung infection complicated by acute respiratory failure, circulatory dysfunction, or multi-organ failure requiring ICU care. It includes severe community-acquired pneumonia (SCAP), severe hospital-acquired pneumonia (HAP), and severe ventilator-associated pneumonia (VAP), but definitions are not fully unified across these entities. SCAP is most commonly defined by the 2007 IDSA/ATS major and minor criteria [1], whereas HAP/VAP severity is often assessed by analogy with SCAP criteria or with general scores such as SOFA and APACHE II [2]. More recent guidelines define SCAP pragmatically as CAP requiring ICU admission or use severity scores such as A-DROP [3,4]. This variation complicates both clinical classification and the interpretation of studies that enroll a broad, mechanically ventilated SP population.

The clinical burden of SP remains substantial. Lower respiratory tract infections are a leading global cause of death [5]. In the United States, CAP causes more than 1.5 million hospitalizations each year, and a considerable proportion of hospitalized patients require ICU admission and invasive mechanical ventilation [6,7]. Severe CAP has high short- and long-term mortality [8], while HAP and VAP are also associated with substantial mortality in China [2]. Invasive mechanical ventilation is therefore central to life support, but injurious ventilation can contribute to ventilator-induced lung injury through volutrauma, barotrauma, atelectrauma, and biotrauma [9]. Optimizing ventilation in SP requires adequate gas exchange while minimizing mechanical stress. This balance is particularly difficult because hypoxemia, hypercapnia, shock, sedation, and spontaneous breathing effort can change rapidly during the first days of ICU treatment.

SP is clinically heterogeneous at both clinically important pathogen and host levels. Bacterial, viral, and fungal pneumonia can produce different inflammatory pathways, respiratory mechanics, and trajectories of host injury [10,11,12]. Immunocompromised hosts have higher rates of opportunistic and mixed infections, atypical presentation, and adverse outcomes [13,14]. ARDS is defined by physiologic and radiographic criteria [15], but SP includes a broader range of infectious and host phenotypes; responses to PEEP, corticosteroids, neuromuscular blockers, and ventilatory settings may therefore vary [16]. We conducted this retrospective cohort study to evaluate associations between daily ventilatory parameters, adjunctive therapies, and ICU mortality in invasively ventilated SP patients and to explore effect modification by pathogen type, hypoxemia severity, and immunocompromised status.

## 2. Methods

### 2.1. Study Design and Participants

This retrospective cohort study was conducted at West China Hospital, Sichuan University. Adult patients with SP who received invasive mechanical ventilation in the ICU between 1 January 2015 and 30 December 2025 were screened. The study was approved by the Biomedical Research Ethics Committee of West China Hospital, Sichuan University (No. 2024-317), with informed consent waived because of the retrospective and non-interventional design.

Patients were included when they were 18–84 years old, admitted to the ICU with SP requiring invasive ventilation, and had a pulmonary pathogen identified within 48 h of ICU admission and initiation of invasive ventilation by sputum culture, targeted next-generation sequencing, or multiplex respiratory viral panel testing. Mixed infections were defined as detection of two or more pathogen types from the same or different specimens within the 48 h window. For baseline description (Table 1), each pathogen type was coded as a binary indicator of detection; for survival analyses, patients were grouped into mutually exclusive categories according to the specific combination of pathogens detected; for subgroup analyses, patients were grouped by the presence versus absence of each pathogen type, regardless of co-infection. SP was defined according to the 2007 IDSA/ATS SCAP criteria, namely one major criterion (invasive mechanical ventilation or septic shock requiring vasopressors) or at least three minor criteria, including tachypnea, PaO_2_/FiO_2_ ≤ 250 mmHg, multilobar infiltrates, confusion, elevated blood urea nitrogen, leukopenia, thrombocytopenia, hypothermia, or hypotension requiring aggressive fluid resuscitation [1]. We did not further classify patients into CAP, HAP, or VAP subgroups, as our inclusion criterion was “severe pneumonia requiring ICU admission and invasive mechanical ventilation” regardless of the site of pneumonia acquisition. The 2007 IDSA/ATS criteria were used as the severity framework for all patients because no equivalent consensus definition of “severe” exists for HAP or VAP. Exclusion criteria were ventilation < 48 h, COVID-19, pregnancy, ECMO at ICU admission, moribund or end-stage disease, pre-existing neuromuscular disease, negative or missing pathogen results, or missing ventilatory parameter data within the first 48 h of invasive ventilation.

### 2.2. Outcomes and Data Collection

The primary outcome was ICU mortality. Secondary outcomes were 28-day mortality, in-hospital mortality, successful extubation, tracheostomy, hospital and ICU length of stay, ventilator-free days at day 28, and complications including barotrauma, septic shock, and acute kidney injury.

Day 0 was defined as ICU admission with initiation of invasive ventilation; for patients intubated after ICU admission, day 0 was the date of invasive ventilation. Demographics, admission department, comorbidities, SOFA score, pathogen type, clinical management, ventilatory settings, gas exchange variables, vital signs, laboratory results, and outcomes were extracted from electronic records. Systemic glucocorticoid and neuromuscular blocker exposure was assessed within the first 7 days after day 0. Ventilatory parameters, gas exchange variables, and vital signs were recorded daily at 10:00 AM from day 0 to day 14 and again on days 21 and 30; laboratory variables were recorded on days 0, 3, 7, 14, and 21.

Immunocompromised host status was defined by active malignancy with recent chemotherapy or neutropenia, solid-organ or stem-cell/bone-marrow transplantation, high-dose or prolonged immunosuppressive therapy, or HIV infection [13]. Ventilatory ratio was calculated as [minute ventilation (mL/min) × PaCO_2_ (mmHg)]/[predicted body weight (kg) × 100 × 37.5] [17]. The composite variable [(4 × driving pressure) + respiratory rate] was calculated as previously described [18].

### 2.3. Statistical Analysis

Continuous variables were summarized as mean (SD) or median (IQR), and categorical variables as counts and percentages. Survivors and non-survivors were compared using *t*-tests, Wilcoxon rank-sum tests, or chi-square tests as appropriate. Kaplan–Meier curves were used for 28-day survival. Because 948 ICU deaths occurred, the number of events was adequate for the prespecified multivariable models under the conventional events-per-variable rule [19]. Longitudinal trends in ventilatory and gas-exchange variables during the first 10 days were assessed with mixed-effects models with random intercepts.

The inferential analyses were organized hierarchically into primary linear analyses, secondary non-linear analyses, and exploratory analyses. The primary analysis evaluated associations between serial ventilatory parameters and ICU mortality using multivariable time-dependent Cox proportional hazards models with repeated daily measurements during the first 10 days. Robust sandwich variance estimators were used to account for within-patient clustering, and analyses were conducted separately according to daily ventilation mode (volume-controlled ventilation [VCV] and pressure-controlled ventilation [PCV]). For the primary time-dependent Cox analyses, patients were followed until ICU death or ICU discharge; patients discharged alive from the ICU were censored at the time of ICU discharge. To reduce collinearity among correlated ventilatory parameters, five prespecified models were fitted. Model 1 included VT/kg, respiratory rate, and PEEP in VCV; Model 2 included minute ventilation and PEEP in VCV; Model 3 included ventilatory ratio and PEEP in VCV; Model 4 included driving pressure, respiratory rate, and PEEP in PCV; and Model 5 included [(4 × driving pressure) + respiratory rate] and PEEP in PCV.

All primary models were adjusted for age, non-pulmonary SOFA score, septic shock, chronic lung disease, heart disease, neurologic disease, liver disease, chronic kidney disease, immunocompromised status, pathogen type, glucocorticoid and neuromuscular blocker use, mean arterial pressure, heart rate, fluid balance, PaO_2_/FiO_2_ ratio, PaCO_2_, pH, lactate, CRP, IL-6, PCT, BNP, albumin, absolute neutrophil and lymphocyte counts, hemoglobin, D-dimer, creatinine, platelet count, and measurement time. Model 3 excluded PaCO_2_ because PaCO_2_ is incorporated into the calculation of the ventilatory ratio. The proportional hazards assumption for the principal ventilatory exposure in each model was formally assessed by adding an exposure-by-time interaction term, with a significant interaction indicating evidence of a time-varying effect.

Missing covariate data were handled using multiple imputation under a missing-at-random (MAR) assumption, conditional on observed clinical, physiological, laboratory, and temporal information. The extent of missingness for each variable before imputation is reported in Supplementary Table S2. For longitudinal variables, missing-ness was calculated among eligible patient-day observations, with structurally unavailable or non-applicable measurements excluded from the denominator. Multiple imputation was performed using multivariate imputation by chained equations (MICE), with classification and regression trees (CART) as the conditional imputation algorithm. Five imputed datasets were generated. Variables selected for imputation and their prespecified predictor variables, based on clinically and physiologically relevant relationships, are detailed in Supplementary Table S3; variables not designated for imputation were handled according to the prespecified imputation structure. Each multivariable Cox model was fitted separately in the five imputed datasets, and regression coefficients and variances were combined according to Rubin’s rules.

Four sensitivity analyses were performed to assess the robustness of the primary findings. First, the primary models were repeated using complete cases only. Second, reduced-covariate models excluded adjustment covariates with substantial missingness (>30% of eligible observations), while retaining the ventilatory exposures of interest irrespective of their missingness. Third, the primary models were repeated using only day-0 ventilatory and physiological measurements as a baseline-only analysis, thereby reducing the potential influence of subsequent treatment adjustments and changes in clinical status. Fourth, the primary models were repeated after excluding patients with septic shock at baseline.

As a secondary analysis, potential non-linear relationships between ventilatory parameters and ICU mortality were evaluated within the corresponding multivariable Cox models by including both linear and quadratic terms for each ventilatory variable, (X) and (X2). The *p* value for the quadratic term was used to test deviation from linearity [20]. These models generated the adjusted non-linear exposure–mortality curves. To assess the robustness of the non-linear associations, we performed sensitivity analyses using restricted cubic splines (RCS).

Two sets of analyses were considered exploratory. First, relative distribution analysis was used to explore possible thresholds that separated ICU survivors and non-survivors [21]; these data-derived thresholds were not used to define the primary Cox models. Second, prespecified subgroup analyses were conducted according to viral infection, fungal infection, hypoxemia severity (PaO_2_/FiO_2_ ≥ 200, 100–199, or <100 mmHg), and immunocompromised status. Given their exploratory nature, no formal adjustment for multiple testing was applied. Subgroup analyses used the same model structure as the primary analysis but were not powered for definitive interaction testing; therefore, findings were interpreted according to the direction and precision of effect estimates and biological plausibility rather than isolated *p* values.

All tests were two-sided, and *p* < 0.05 was considered statistically significant. Analyses were performed using R version 4.1.3. A schematic overview of the statistical analysis workflow is provided in Supplementary Figure S1.

## 3. Results

### 3.1. Baseline Characteristics and Outcomes

A total of 2480 patients were included. Baseline characteristics, clinical management, gas exchange variables, and laboratory parameters are summarized in Table 1, and outcomes are presented in Table 2. Mean age was 63.28 (16.98) years, and 1710 patients (68.95%) were male. Immunocompromised status was present in 970 patients (39.11%). Bacterial pathogens were identified in 2230 patients (89.92%), viral pathogens in 686 (27.66%), and fungal pathogens in 1225 (49.40%); mixed infections (detection of two or more pathogen types) were common. The classification of pathogen groups for baseline description, survival analyses, and subgroup analyses differed according to the analytical purpose, as detailed in the Methods. Systemic glucocorticoids were used in 1652 patients (66.61%) and neuromuscular blockers in 627 (25.28%). Baseline illness severity was high, with a mean total SOFA score of 10.39 (3.25), septic shock in 600 patients (24.19%), and a median initial PaO_2_/FiO_2_ ratio of 173.06 (IQR 120.33–243.04).

**Figure 1:**
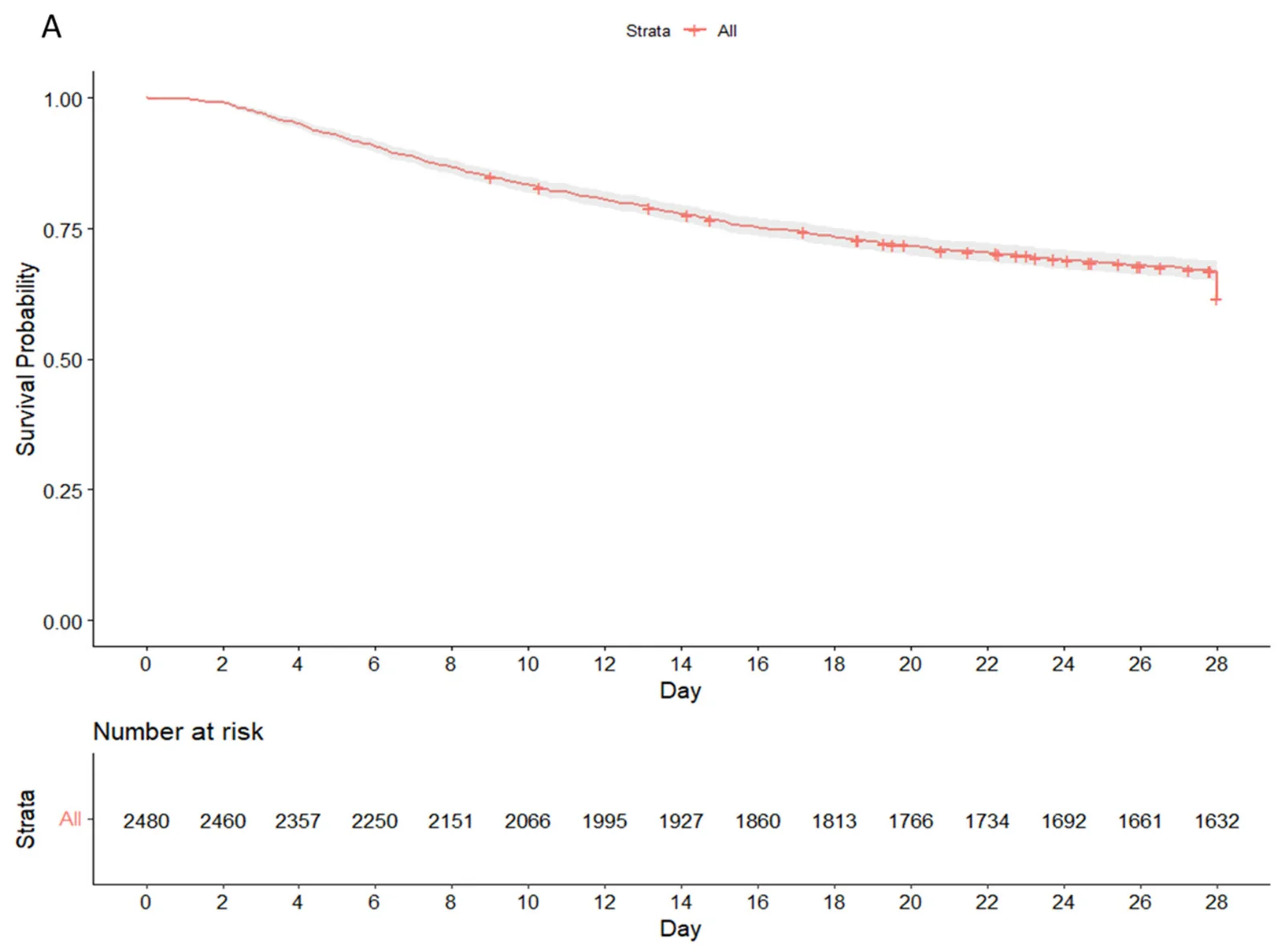

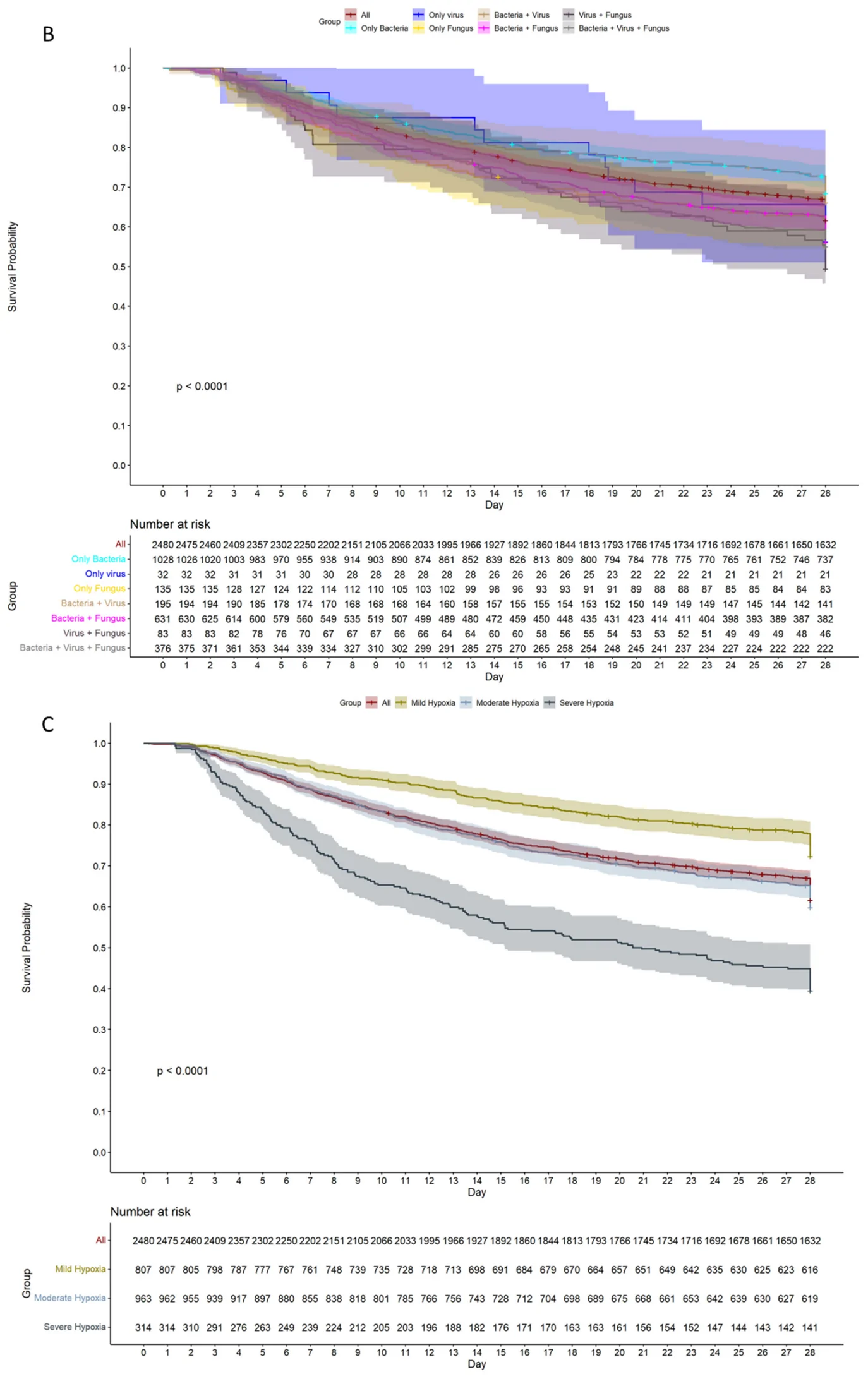

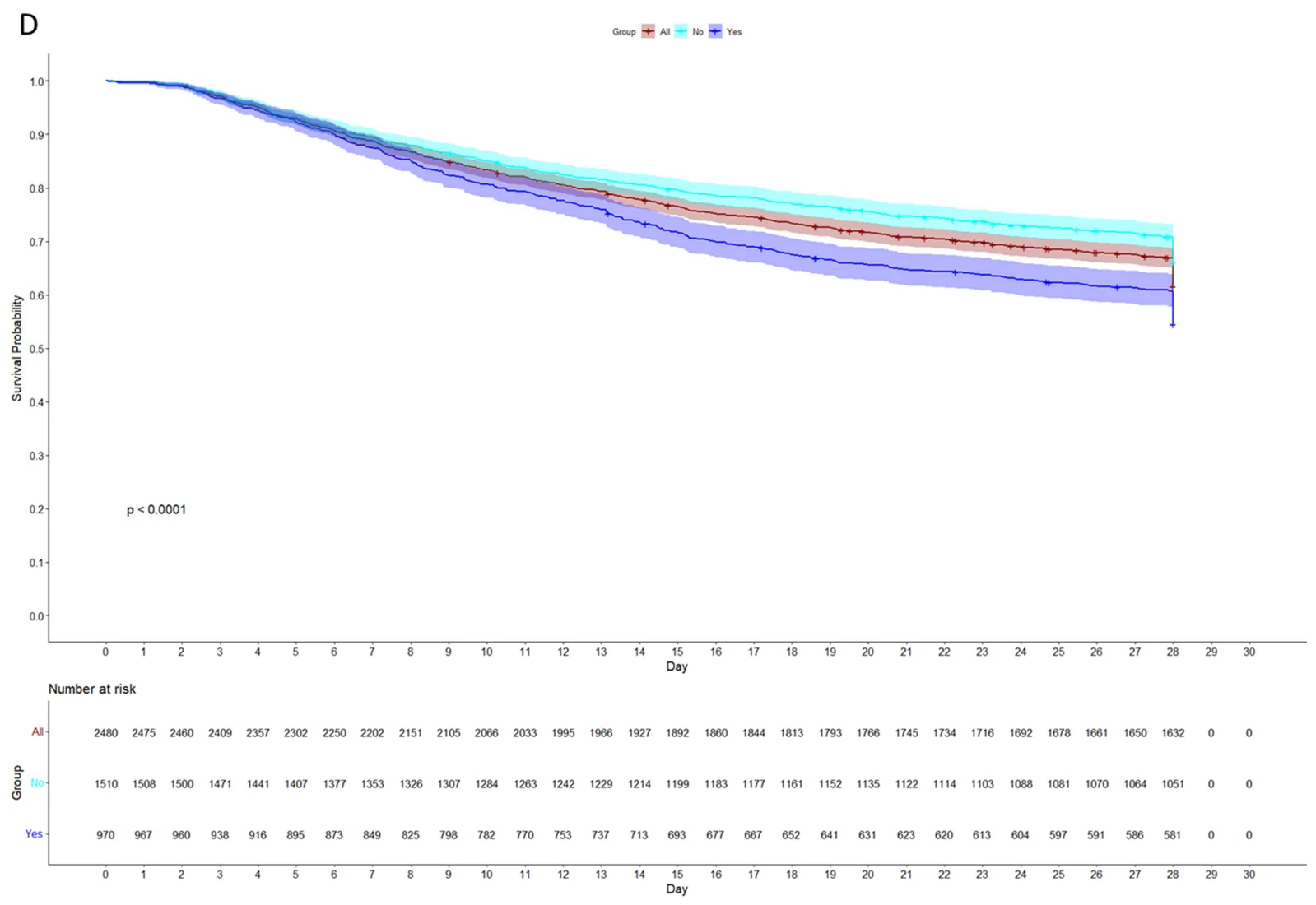
Kaplan–Meier survival curves for 28-day mortality in ICU patients. (**A**) Overall survival in the total cohort (*n* = 2480). (**B**) Survival stratified by pathogen type (bacterial, viral, fungal, and mixed infections). (**C**) Survival stratified by severity of hypoxemia based on PaO_2_/FiO_2_ ratio (mild: ≥200, moderate: 100–200, severe: <100). (**D**) Survival stratified by immunocompromised status (immunocompromised [Yes] vs. non-immunocompromised [No]).

During the ICU stay, 948 patients (38.23%) died; 28-day mortality was 34.19%, and in-hospital mortality was 40.73%. ICU non-survivors were older (65.88 vs. 61.67 years), more often immunocompromised (46.31% vs. 34.66%), and had higher total SOFA scores (11.58 vs. 9.65) and lower initial PaO_2_/FiO_2_ ratios (143.93 vs. 190.33) than survivors. Non-survivors also had more fungal infection, more frequent glucocorticoid exposure (76.37% vs. 60.57%), and more neuromuscular blocker exposure (35.02% vs. 19.26%). For the Kaplan–Meier survival analyses, patients were classified into mutually exclusive groups according to the specific pathogen combination detected: only bacteria, only virus, only fungus, bacteria + virus, bacteria + fungus, virus + fungus, and bacteria + virus + fungus. Kaplan–Meier analyses showed lower survival in patients with mixed infections (Figure 1). In addition, patients with more severe hypoxemia or immunocompromised status also exhibited lower survival (Figure 1). Secondary outcomes were also worse in non-survivors. Successful extubation occurred in 41.29% of the whole cohort but in only 8.23% of ICU non-survivors, and ventilator-free days at day 28 were essentially absent among non-survivors. Barotrauma, septic shock during the ICU course, and acute kidney injury were more frequent in non-survivors. These outcome patterns support the clinical validity of ICU mortality as the primary endpoint and indicate that death was accompanied by persistent respiratory failure and multi-organ complications rather than by a single isolated event. They also underscore that ventilator variables were embedded in a broader trajectory of organ dysfunction.

### 3.2. Ventilatory Parameters and Gas Exchange

**Figure 2:**
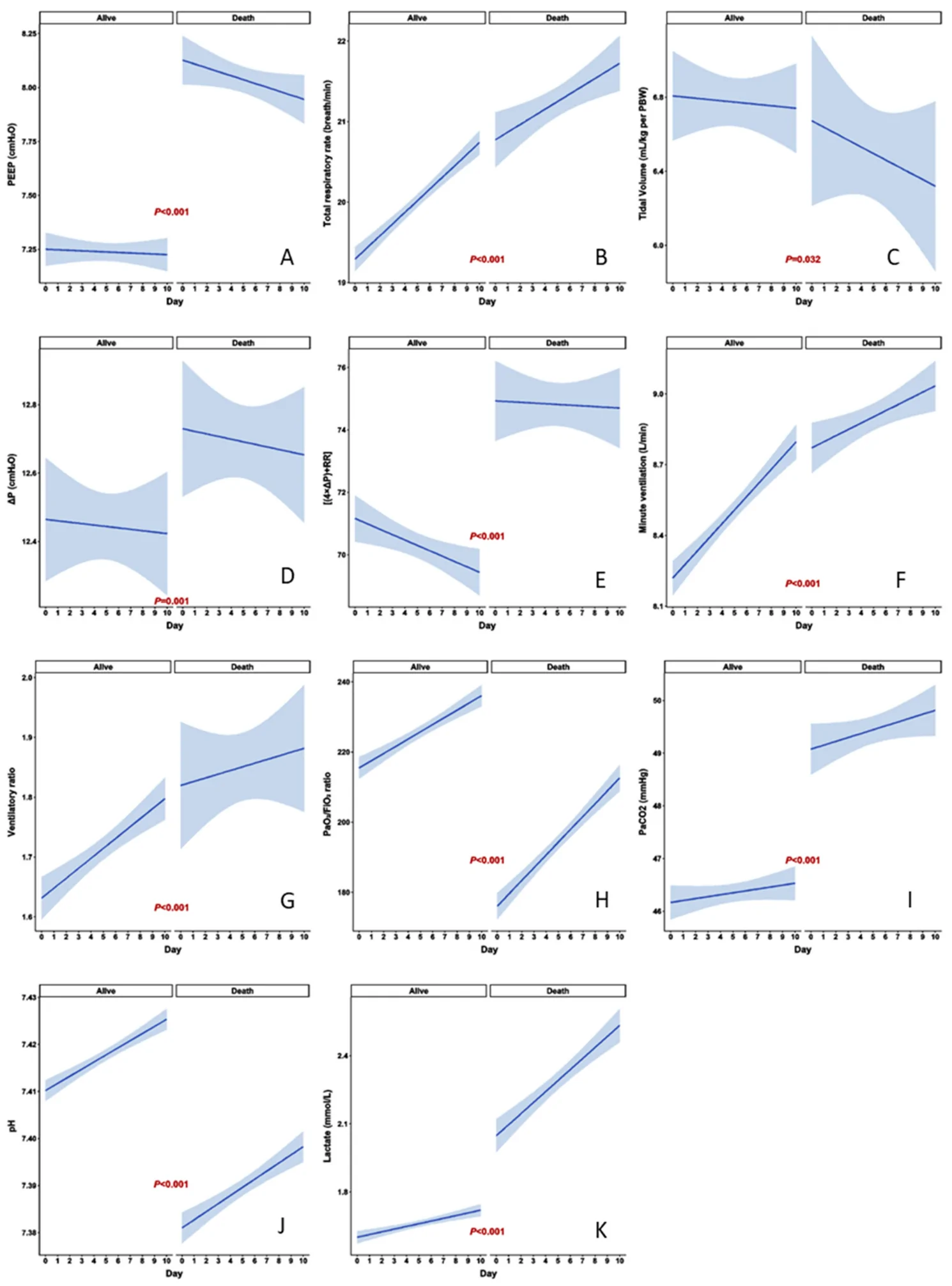
Temporal trends of ventilatory parameters and blood gas variables over the first 10 days in ICU survivors versus non-survivors. Panels: (**A**) PEEP (cmH_2_O), (**B**) respiratory rate (RR, breaths/min), (**C**) tidal volume per body weight (VT/kg, mL/kg), (**D**) driving pressure (ΔP, cmH_2_O), (**E**) (4 × ΔP) + RR, (**F**) minute ventilation (VE, L/min), (**G**) ventilatory ratio (VR), (**H**) PaO_2_/FiO_2_ ratio (P/F ratio), (**I**) partial pressure of carbon dioxide (PaCO_2_, mmHg), (**J**) arterial pH, (**K**) lactate (mmol/L). *p*-values for overall between-group differences over the 10-day period were calculated using mixed-effects linear regression models with random intercepts; *p* < 0.05 was considered statistically significant.

Ventilatory parameters and gas exchange variables over the first 10 days are summarized in Supplementary Table S1. During the first 10 days, ventilatory settings were generally within protective ranges. Median respiratory rate was 20 breaths/min (IQR 16–24), PEEP 8 cmH_2_O (IQR 5–10), VT/kg 7 mL/kg predicted body weight (IQR 6–8), driving pressure 12 cmH_2_O (IQR 10–15), [(4 × driving pressure) + respiratory rate] 70 (IQR 61–78), minute ventilation 9 L/min (IQR 7–10), and ventilatory ratio 1.94 (IQR 1.43–2.37). Median PaO_2_/FiO_2_ ratio was 200 (IQR 143–266), PaCO_2_ was 44 mmHg (IQR 38–53), pH was 7.41 (IQR 7.37–7.44), and lactate was 1.87 mmol/L (IQR 1.31–2.09). Figure 2 presents the longitudinal trends of ventilatory parameters and blood gas variables over the first 10 days in ICU survivors versus non-survivors. Compared with survivors, non-survivors had persistently higher PEEP, respiratory rate, driving pressure, composite mechanical variable, minute ventilation, ventilatory ratio, PaCO_2_, and lactate, and lower VT/kg, PaO_2_/FiO_2_ ratio, and pH.

### 3.3. Multivariable Analyses

None of these interaction terms was statistically significant (all *p* > 0.05), providing no evidence of time-varying effects for the principal exposures (Supplementary Table S4).

**Figure 3:**
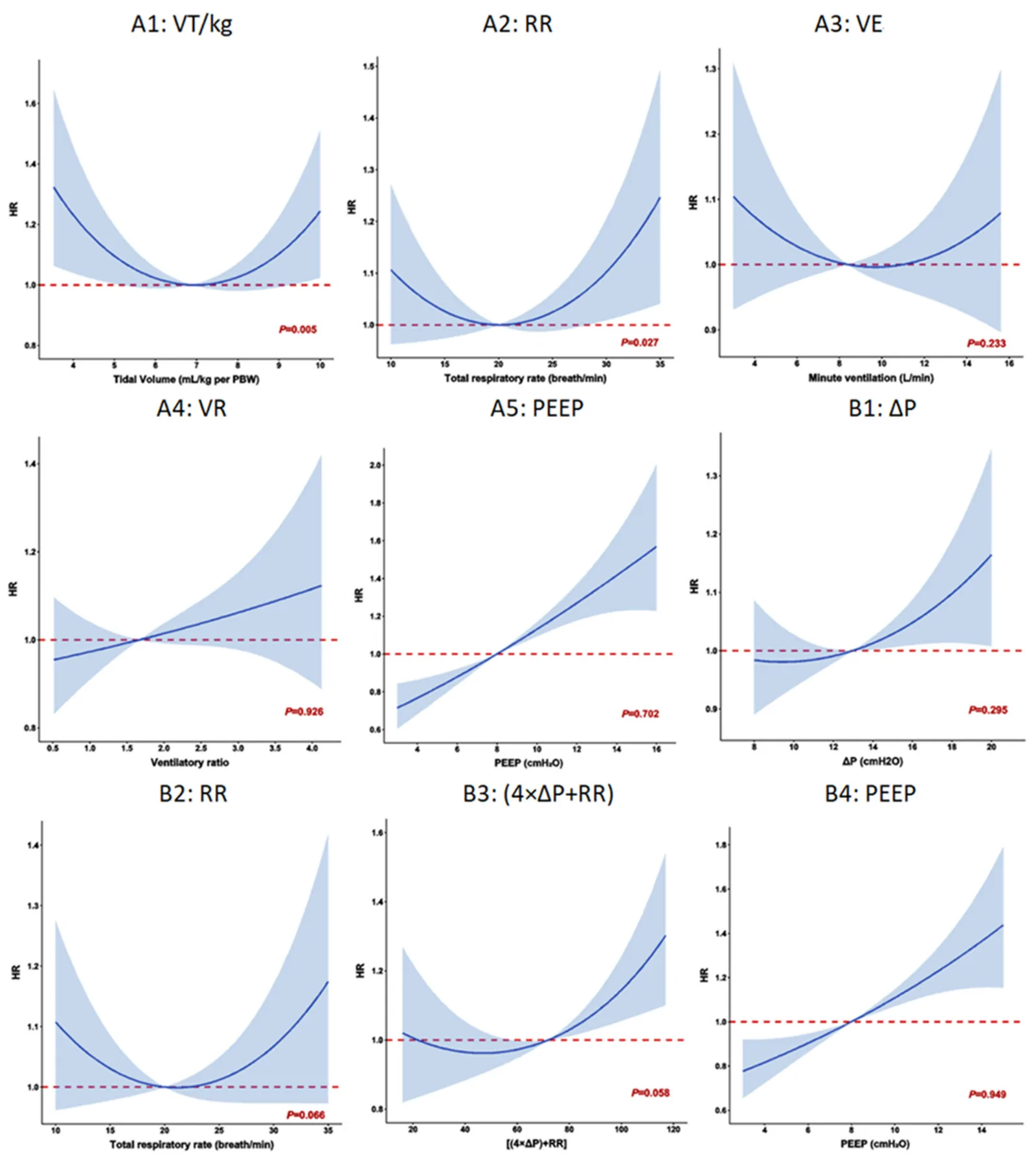
Non-linear associations of ventilatory parameters with ICU mortality using quadratic polynomial models in time-dependent Cox regression. HRs were modeled using quadratic polynomial terms in time-dependent Cox models, adjusted for all covariates listed in Table 3 footnote. Time-dependent covariates were handled using the time-varying Cox regression model with repeated measures. Analyses were stratified by ventilation mode (volume-controlled vs. pressure-controlled). Solid lines = adjusted HRs; shaded areas = 95% CIs; dashed line = HR = 1. *p*-values shown are for the quadratic (non-linear) term, testing deviation from linearity. A significant non-linear *p*-value suggests a non-linear association with ICU mortality; a non-significant non-linear *p*-value does not exclude a linear association (see Table 3). Panels (**A1**–**A5**) show results from the volume-controlled ventilation group: (**A1**) VT/kg from Model 1; (**A2**) RR from Model 1; (**A3**) VE from Model 2; (**A4**) VR from Model 3; and (**A5**) PEEP (representative, from Model 1). Panels (**B1**–**B4**) show results from the pressure-controlled ventilation group: (**B1**) ΔP from Model 4; (**B2**) RR from Model 4; (**B3**) (4 × ΔP + RR) from Model 5; and (**B4**) PEEP (representative, from Model 4). Of note, PEEP was included in all five models, with nearly identical effect estimates across models. For conciseness, PEEP curves are shown only once per ventilation group (Model 1 and Model 4). PEEP curves from Models 2, 3, and 5 are shown in the Supplementary Materials (Supplementary Figures S3–S5). RR was included in Models 1 and 4 only, the corresponding curves are shown in (**A2**,**B2**). VT/kg, tidal volume per kg PBW; VE, minute ventilation; VR, ventilatory ratio; ΔP, driving pressure; RR, respiratory rate; PEEP, positive end-expiratory pressure.

The HRs presented in Table 3 and Table 4 are derived from linear models assuming a linear relationship for each ventilatory variable. Figure 3 displays the non-linear HR curves from the quadratic models. As shown in Table 3 and Table 4, in the primary imputed time-dependent Cox models, PEEP was consistently associated with higher ICU mortality in both VCV and PCV models (HR 1.04 per 1 cmH_2_O, 95% CI 1.02–1.05, *p* < 0.001). Systemic glucocorticoid use was also associated with higher ICU mortality (HR 1.40–1.41, *p* < 0.001), as was neuromuscular blocker use (HR 1.53–1.56, *p* < 0.001) (Table 3 and Table 4). In contrast, respiratory rate, VT/kg, minute ventilation, ventilatory ratio, driving pressure, and [(4 × driving pressure) + respiratory rate] showed no significant linear associations in the corresponding primary models (Table 3 and Table 4). The glucocorticoid and neuromuscular blocker estimates remained consistent across all four sensitivity analyses. Complete-case analyses attenuated the PEEP association, but it remained stable in the other three. Respiratory rate, minute ventilation, and [(4 × ΔP) + RR] became statistically significant after excluding covariates with >30% missingness, while the VT/kg association varied in the day-0 analysis. Overall, these sensitivity analyses support the robustness of the primary findings in terms of direction of effect, while indicating that certain ventilatory parameters may be more susceptible to covariates with higher proportions of missing data. These associations should therefore be interpreted with particular caution, as primary-model associations rather than definitive causal effects (Supplementary Tables S10–S17).

Figure 3 displays the results of the non-linear polynomial regression analyses. Non-linear models showed that VT/kg had a significant U-shaped association with ICU mortality in the VCV group (*p* for non-linearity = 0.005, Figure 3(A1)), with the lowest risk around the commonly protective range of 6–8 mL/kg. From the non-linear HR curves, the HR for VT/kg remained close to 1 across approximately 6–8 mL/kg, with elevated risk below approximately 5 mL/kg and above approximately 9 mL/kg. Respiratory rate showed a significant J-shaped association in the VCV group (*p* for non-linearity = 0.027, Figure 3(A2)), suggesting increased risk at extremes; the HR remained close to 1 between approximately 18 and 26 breaths/min, with increased risk at rates below approximately 14 or above approximately 30 breaths/min. Minute ventilation, ventilatory ratio, and PEEP did not show significant non-linear deviations in VCV. In PCV, [(4 × driving pressure) + respiratory rate] showed a non-linear trend in both quadratic (*p* = 0.058, Figure 3(B3)) and RCS analyses (*p* = 0.026, Supplementary Figure S6), with the latter further supporting this relationship. While respiratory rate, driving pressure, and PEEP were not significantly non-linear in PCV. For conciseness, PEEP curves are shown only once per ventilation group (Model 1 and Model 4). PEEP curves from Models 2, 3, and 5 are presented in the Supplementary Materials (Supplementary Figures S3–S5), confirming the robustness of the PEEP association regardless of the primary respiratory variable included. RCS sensitivity analyses yielded results closely similar to the primary polynomial findings (Supplementary Figure S6).

### 3.4. Exploratory Analyses

**Figure 4:**
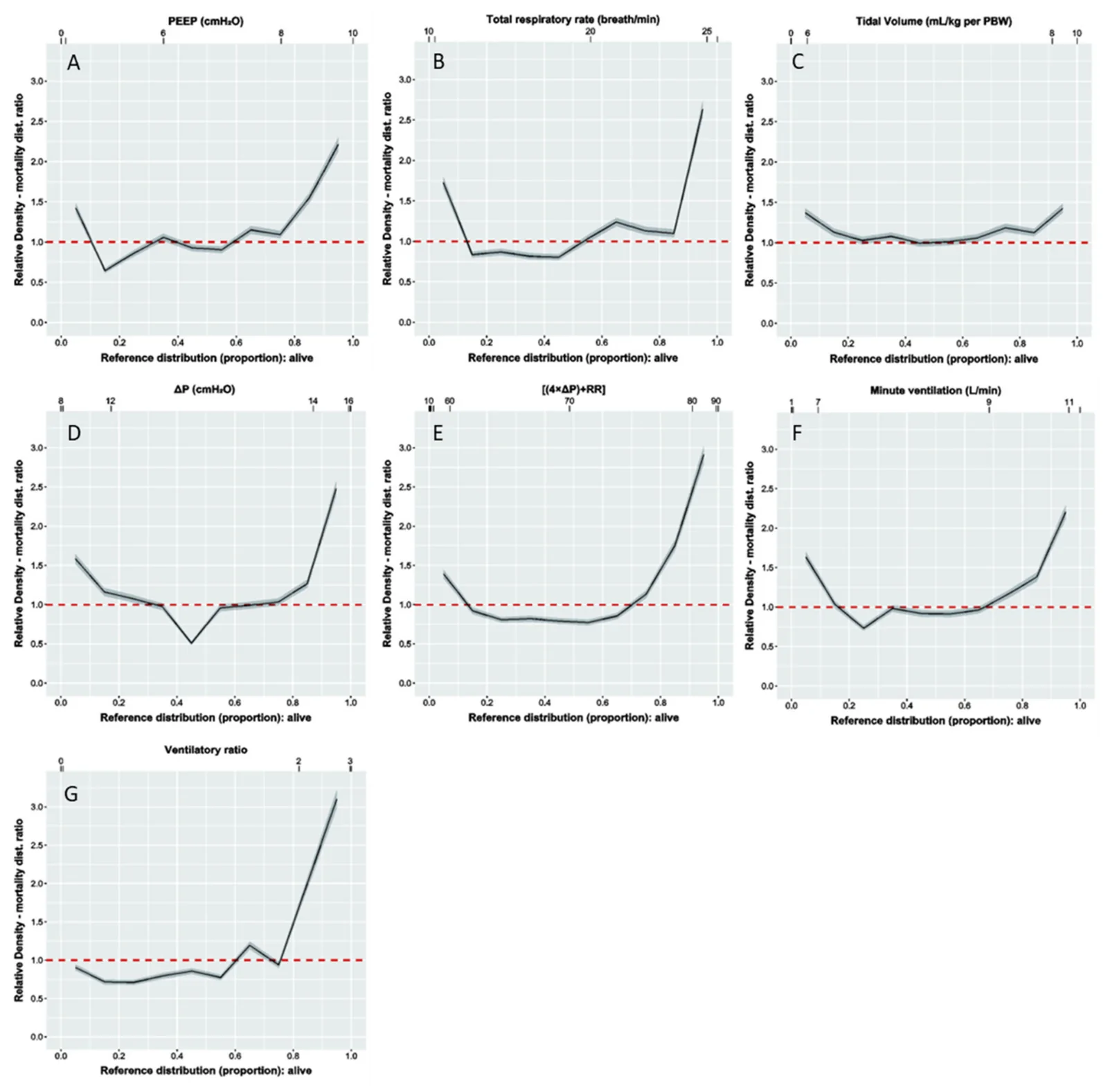
Relative distribution analysis for the identification of exploratory, data-driven thresholds of ventilatory parameters associated with ICU mortality. The cut-off point along the continuum of each marker that separated ICU survivors versus non-survivors was determined by comparing the quantile (proportion) distribution of the marker in survivors (plotted on the x-axis, with corresponding marker values shown at the top) against the relative density (mortality distribution ratio) of the marker in non-survivors (plotted on the y-axis). Panels: (**A**) PEEP (cmH_2_O), (**B**) respiratory rate (RR, breaths/min), (**C**) tidal volume per body weight (VT/kg, mL/kg), (**D**) driving pressure (ΔP, cmH_2_O), (**E**) (4 × ΔP) + RR, (**F**) minute ventilation (VE, L/min), (**G**) ventilatory ratio (VR). The threshold was defined as the marker value corresponding to the crossing point(s) where the relative density ratio crossed the reference line of 1.0, indicating the threshold(s) where the mortality distribution ratio diverged between survivors and non-survivors. For markers exhibiting a U-shaped association with mortality, two crossing points were identified: the lower inflection point (left crossing) defining the lower boundary of the protective range, and the upper inflection point (right crossing) defining the high-risk threshold above which mortality risk increased significantly.

Figure 4 presents the relative distribution analysis for the identification of exploratory, data-driven thresholds of ventilatory parameters associated with ICU mortality. The analysis identified possible thresholds that discriminated ICU survivors from non-survivors. These thresholds were derived from the same study population and were not tested in an independent cohort; therefore, they should be interpreted as exploratory and hypothesis-generating rather than as definitive clinical targets.

**Figure 5:**
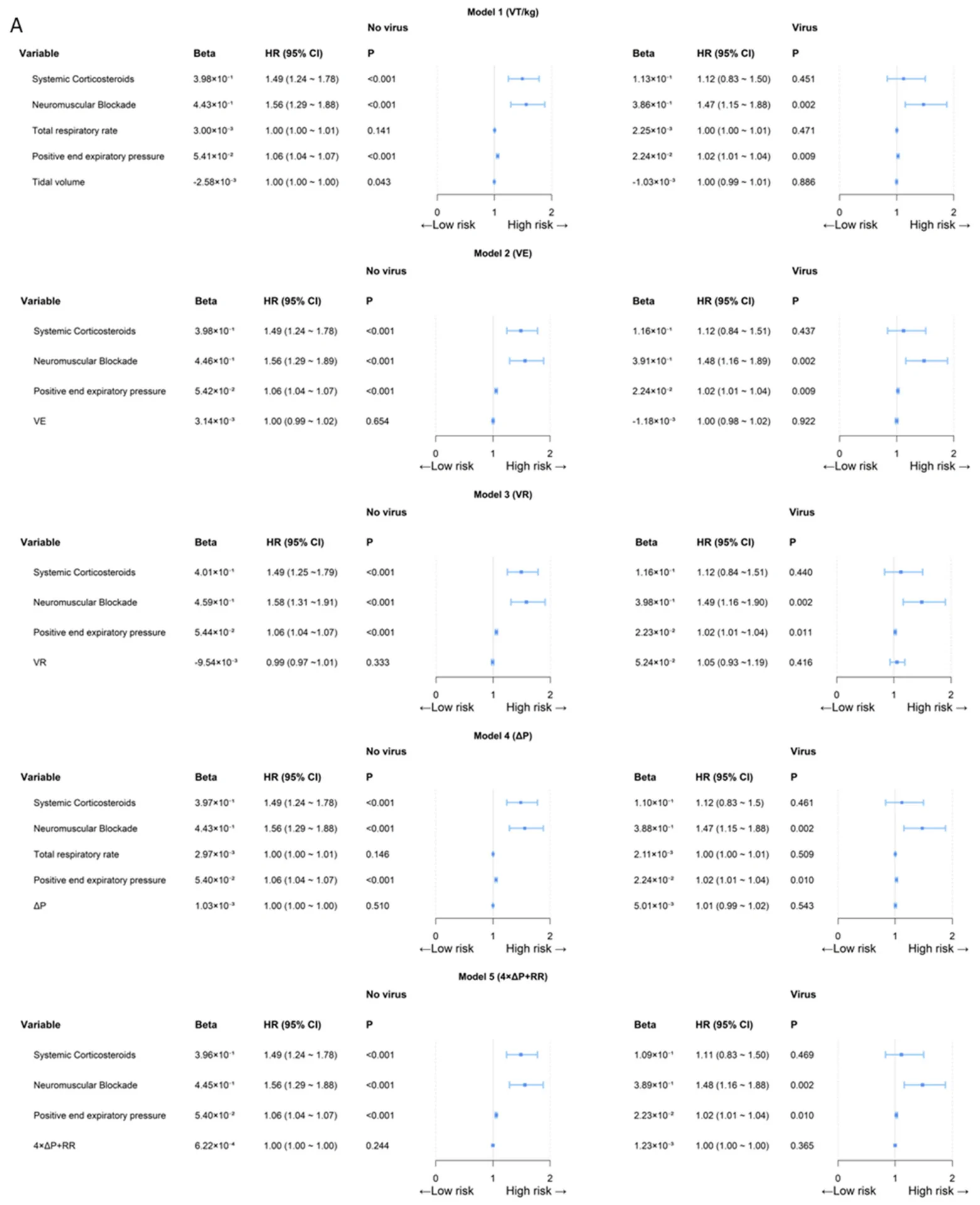

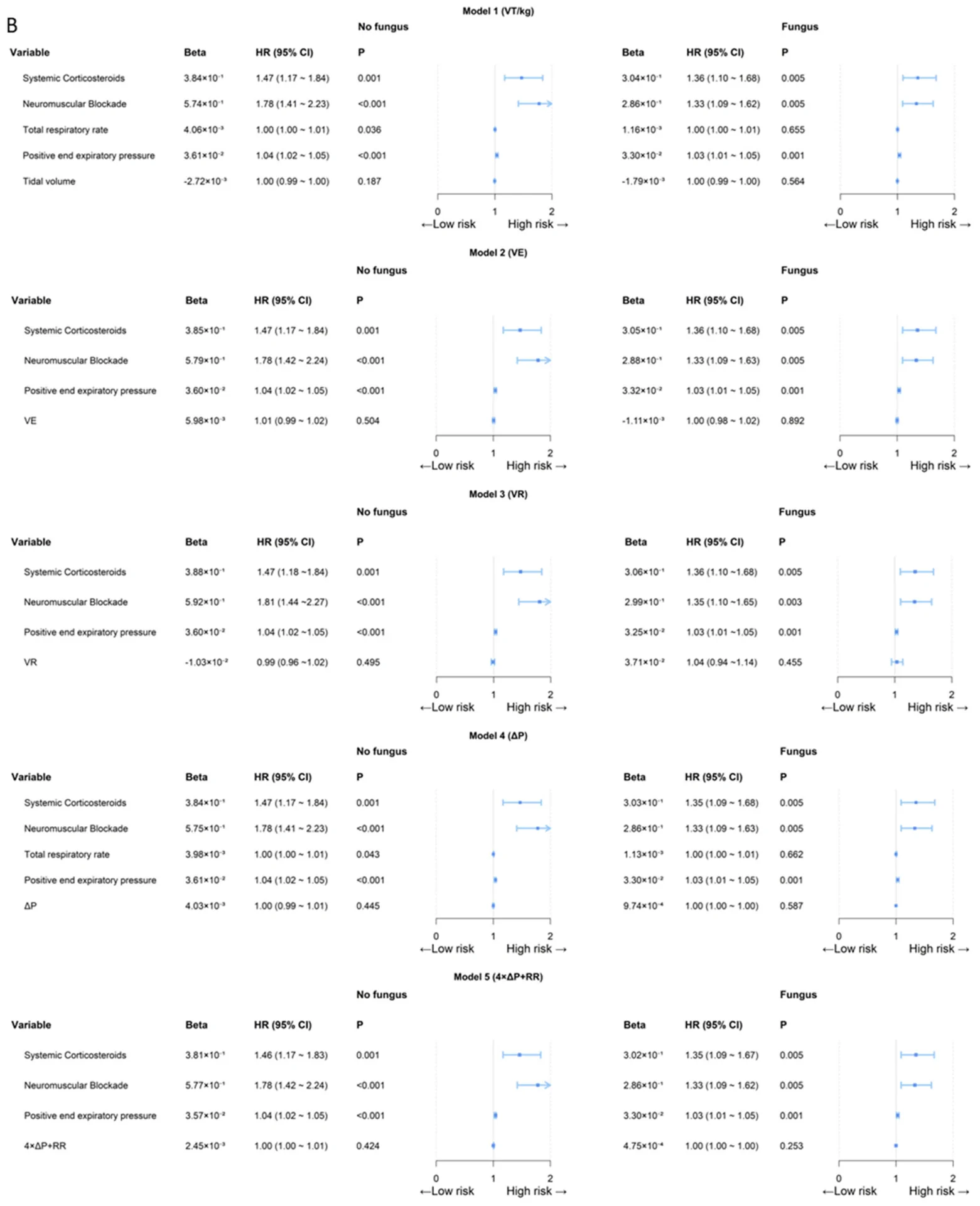

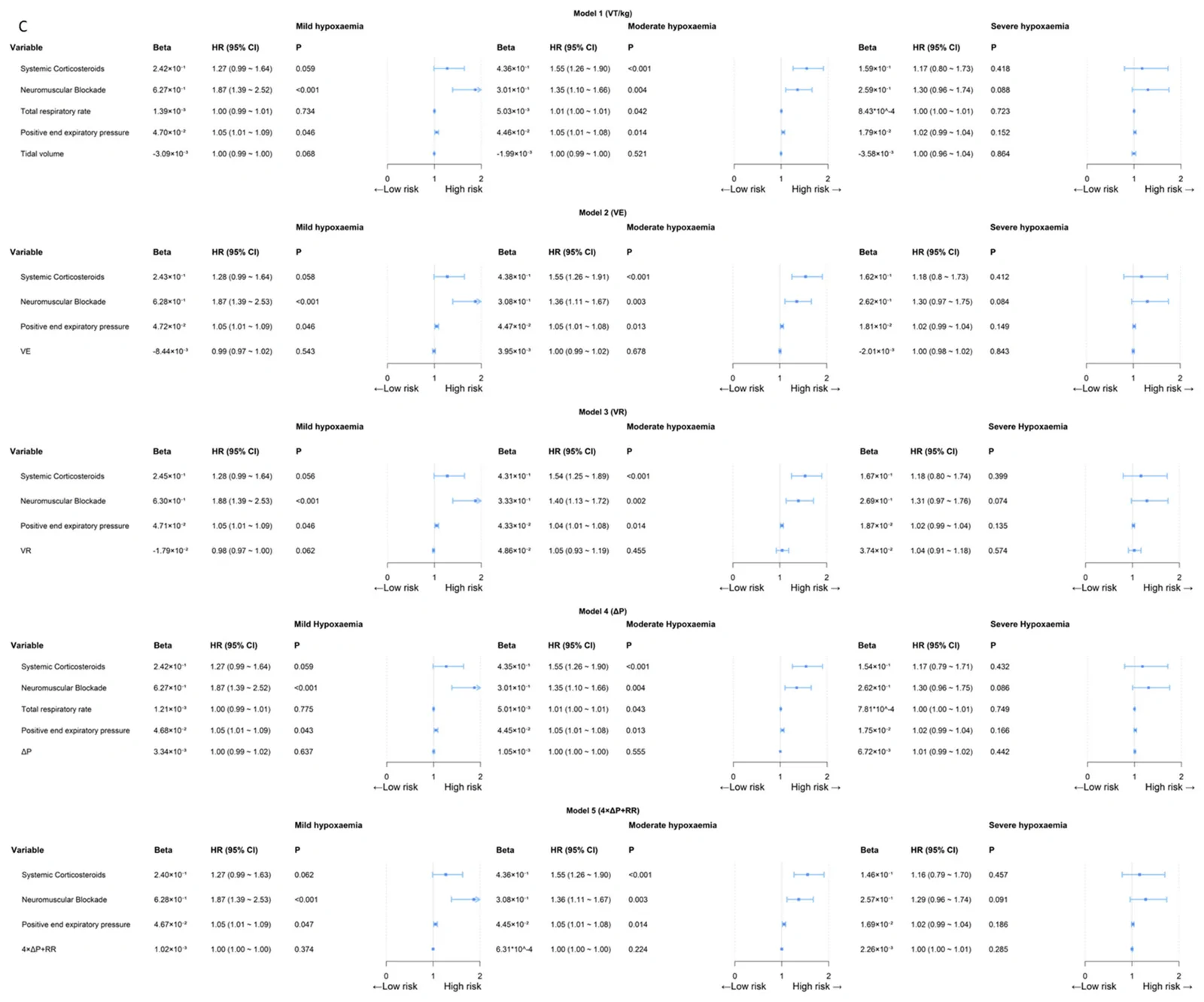

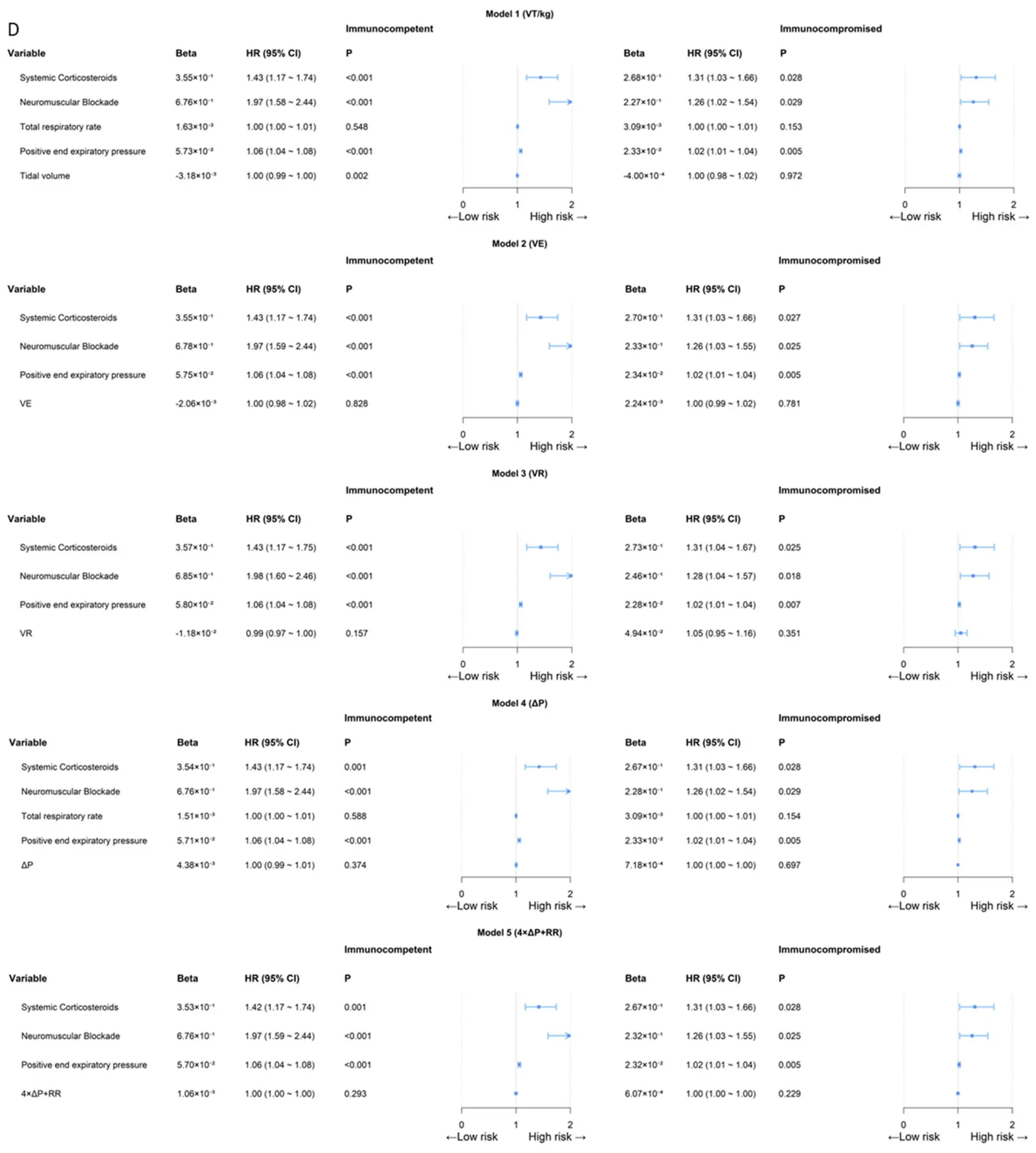
Subgroup analysis of key parameters associated with ICU mortality. (**A**) Subgroup analysis stratified by viral infection status (viral vs. non-viral). (**B**) Subgroup analysis stratified by fungal infection status (fungal vs. non-fungal). (**C**) Subgroup analysis stratified by hypoxemia severity (mild vs. moderate vs. severe). (**D**) Subgroup analysis stratified by immunocompromised status (immunocompetent vs. immunocompromised). Forest plots show adjusted hazard ratios (HRs) and 95% confidence intervals (CIs) for key parameters across prespecified subgroups. HRs were derived from multivariable time-dependent Cox regression models (volume-controlled group: Models 1–3; pressure-controlled group: Models 4–5), adjusted for the same set of covariates as the primary analysis (see Table 3 footnote). The dashed vertical line at HR = 1 indicates no effect. VT/kg, tidal volume per kg predicted body weight; VE, minute ventilation; VR, ventilatory ratio; ΔP, driving pressure; RR, respiratory rate; PEEP, positive end-expiratory pressure. Detailed model outputs for each subgroup are provided in the Supplementary Materials (Tables S18–S37).

Subgroup analyses were exploratory (Figure 5 and Supplementary Tables S18–S37). For these subgroup analyses, patients were grouped by the presence versus absence of each pathogen type (e.g., viral vs. non-viral, fungal vs. non-fungal), regardless of co-infection with other pathogens, because this binary grouping was necessary to maintain mutually exclusive, sufficiently sized strata for model estimation. Glucocorticoid use was no longer statistically significant in the viral infection subgroup, while most other associations were similar to the overall analysis. Respiratory rate was positively associated with mortality in the non-fungal and moderate hypoxemia subgroups. In severe hypoxemia, PEEP, glucocorticoids, and neuromuscular blockers were not significantly associated with ICU mortality. VT/kg appeared protective in non-viral and immunocompetent subgroups, but this finding was not consistent with the primary non-linear analysis and may reflect multiple testing, smaller subgroup size, or residual confounding. The subgroup results therefore were used to generate hypotheses rather than to define treatment targets. Their main value is to show that the SP population is not uniform: the direction and strength of associations may change when patients are grouped by pathogen, oxygenation status, or host immune function. In particular, the absence of significant PEEP, glucocorticoid, and neuromuscular blocker associations in severe hypoxemia should be interpreted cautiously because this subgroup was smaller, had more uniform exposure to rescue therapies, and had mortality strongly driven by baseline disease severity.

## 4. Discussion

In this large cohort of invasively ventilated patients with SP, current ventilatory practice was largely lung protective. The main primary-model findings were that higher PEEP, systemic glucocorticoid exposure, and neuromuscular blocker exposure were associated with higher ICU mortality; VT/kg and respiratory rate showed important non-linear patterns; and driving pressure, minute ventilation, ventilatory ratio, and [(4 × driving pressure) + respiratory rate] were not significantly associated with mortality in the adjusted linear models. The results are consistent with the concept of individualized management, although they do not establish causality. Associations between therapies and mortality should be read as signals from real-world practice, where clinicians preferentially apply higher PEEP, corticosteroids, or paralysis to the sickest patients.

While optimal management of infection and septic shock remains the cornerstone of improving survival in severe pneumonia, minimizing ventilator-induced lung injury represents a distinct and modifiable component of ICU care. In this context, the longitudinal collection of ventilatory and gas-exchange variables over the first 10 days of mechanical ventilation constitutes a key strength of this study. Physiological responses after acute lung injury evolve over time, and measurements obtained only at admission may inadequately represent this dynamic trajectory [22]. Our daily time-dependent approach captures these temporal changes, providing a more accurate assessment of exposure–outcome associations and a real-world snapshot of ventilator management across a heterogeneous SP population. Importantly, we identified specific, quantifiable non-linear thresholds—VT/kg 6–8 mL/kg and respiratory rate 18–26 breaths/min—which offer actionable, data-driven boundaries for bedside titration rather than merely qualitative associations.

The observed ventilation settings are consistent with modern adoption of protective ventilation in ARDS and non-ARDS ICU populations [23,24]. Median VT/kg was 7 mL/kg and median driving pressure was 12 cmH_2_O, lower than values reported in older mechanically ventilated cohorts [25] and close to ARDSNet-derived protective targets [26]. Driving pressure has been strongly associated with ARDS survival [27], and values below approximately 15 cmH_2_O are often regarded as relatively safe [28]. This may explain why driving pressure was not significantly associated with mortality in our adjusted models: most observed values were already in a protective range, and additional reductions may have limited measurable benefit. It also emphasizes a key interpretive point: when a cohort is already managed near accepted safety targets, observational analyses may detect little incremental effect from further lowering a variable, even if the same variable is harmful at extreme values.

PEEP requires balancing recruitment against overdistension and hemodynamic compromise [9,29,30]. In the primary models, higher PEEP was associated with higher mortality. This could reflect a causal effect in patients with focal pneumonia or low recruitability, but confounding by indication in patients with worse oxygenation is at least equally plausible, and the two explanations are not mutually exclusive. The loss or attenuation of statistical significance in severe hypoxemia and complete-case analyses argues against a universal conclusion that higher PEEP is harmful. The attenuation of the PEEP association in complete-case analyses indicates that this finding is sensitive to how missing data were handled, which further supports a cautious, non-causal interpretation. In patients with more recruitable lungs, the benefits of PEEP may offset overdistension, while aggressive recruitment and high-PEEP strategies can also cause harm in unselected ARDS populations [31,32]. These findings highlight the potential value of individualized PEEP titration over routine escalation, although this remains to be tested in prospective studies. Bedside approaches such as careful evaluation of oxygenation response, driving pressure response, hemodynamic tolerance, imaging pattern, and, when available, recruitability assessment may help identify patients in whom PEEP improves lung protection rather than adding stress.

Respiratory rate and VT/kg should be interpreted together. Experimental and clinical data suggest that excessive respiratory rate can increase dynamic strain and mechanical power [18,33,34]. Our J-shaped respiratory-rate result implies that both inadequate and excessive rates may be unfavorable. Low rates may lead to hypoventilation and acidosis, whereas high rates may reflect respiratory distress, increased drive, dead space, or clinician response to hypercapnia. Beyond ventilator settings, respiratory rate is modulated by respiratory drive, hypoxemia, inflammation, and chemoreflex activity [35], and should not be interpreted as an isolated ventilator variable but rather as a reflection of the interplay between ventilator settings, pathophysiology, host response, and clinician interventions. Of note, the positive linear association between respiratory rate and mortality in subgroup analyses was predominantly observed in non-fungal patients, suggesting that in fungal pneumonia mortality is more strongly determined by pathogen and host immune status; alternatively, this may reflect reduced statistical power in the fungal subgroup. This finding should therefore be considered hypothesis-generating. For VT/kg, low tidal volume reduces mortality in ARDS [26], especially when respiratory-system elastance is high [36], but very low VT in non-ARDS or higher-compliance patients may promote hypercapnia, dyspnea, or compensatory tachypnea [37]. The U-shaped VT/kg association therefore supports maintaining a protective middle range rather than pursuing uniformly lower VT for every patient. Additionally, the linear associations for respiratory rate and VT/kg became statistically significant in sensitivity analyses excluding covariates with >30% missingness or using day-0 measurements, respectively, suggesting that these linear associations are more susceptible to analytical choices; the non-linear relationships (J-shaped for respiratory rate, U-shaped for VT/kg) should therefore be the primary focus of interpretation.

Minute ventilation and ventilatory ratio did not independently predict mortality after adjustment. Higher minute ventilation may be a response to metabolic demand, acidosis, fever, or dead space rather than a direct injurious exposure. Ventilatory ratio is a practical surrogate for ventilatory inefficiency and dead space, both prognostic in ARDS [38,39,40], but its independent value in heterogeneous SP may be limited. Prior work also suggests that the association between ventilatory ratio and mortality may be mediated by dyscapnia rather than by ventilatory ratio itself [41]. Thus, ventilatory ratio may remain useful for bedside physiology, but should not be interpreted as a standalone therapeutic target in SP.

The null finding for airway driving pressure should not be interpreted as proof that mechanical stress is unimportant. Airway driving pressure may poorly represent transpulmonary driving pressure when chest-wall elastance or spontaneous breathing effort is substantial [42,43], and clinical trials targeting lower driving pressure have not uniformly improved outcomes [31,44]. Thus, the absence of an independent association for driving pressure in this cohort does not exclude a role for mechanical stress in SP patients, particularly when assessed through more integrative measures. In contrast, the composite parameter [(4 × ΔP) + RR] showed non-linear associations with ICU mortality in both quadratic (*p* = 0.058) and RCS analyses (Supplementary Figure S6), suggesting that this integrative marker of mechanical load may capture risk beyond individual components. Future studies using esophageal pressure, imaging, and recruitability assessment may better identify patients who benefit from individualized pressure and power targets [45].

Notably, the sensitivity analysis excluding covariates with >30% missingness rendered both minute ventilation and [(4 × ΔP) + RR] statistically significant, suggesting that these linear associations may be influenced by adjustment for highly incomplete covariates; their non-significant findings in the primary analysis should therefore be interpreted with this in mind.

Systemic glucocorticoids remain controversial in severe pneumonia. CAPE-COD showed benefit from early hydrocortisone in severe CAP [46], whereas ESCAPe did not show a methylprednisolone mortality benefit [47], and REMAP-CAP raised uncertainty for some hydrocortisone regimens [48]. Meta-analyses suggest possible short-term mortality reduction but with heterogeneity [49,50]. Guidelines increasingly support corticosteroids in selected severe CAP, ARDS, or septic shock contexts, but caution is advised in influenza and fungal pneumonia [51,52,53]. Our positive association with mortality is likely influenced by confounding by indication, mixed infection phenotypes, and unmeasured variation in timing, dose, agent, and duration. These findings therefore highlight the importance of careful patient selection and documentation of indication, rather than supporting a general conclusion about harm or benefit from these agents in all SP patients.

Neuromuscular blockers can improve synchrony and facilitate lung-protective ventilation, yet evidence in ARDS is mixed: ACURASYS suggested benefit with early cisatracurium [54], whereas ROSE found no mortality benefit under lighter sedation [55]. Contemporary guidance discourages routine continuous infusion and favors selective or intermittent use when needed [52,56]. In our cohort, neuromuscular blocker exposure was associated with higher mortality, probably reflecting residual severity, deep sedation, prolonged ventilation, and possible ICU-acquired weakness [57,58]. The attenuation of this association in severe hypoxemia may indicate reduced power, more uniform exposure, or a subgroup in which paralysis facilitates protective ventilation. These findings are consistent with a restrictive strategy: use neuromuscular blockers for clear indications, reassess daily, and minimize duration.

Several clinical implications follow from these findings. First, protective ventilation appears feasible in SP and is consistent with current practice, but the target should not be reduced to a single number. VT, respiratory rate, PEEP, driving pressure, gas exchange, and hemodynamics should be considered together because compensating for one variable may worsen another. Second, PEEP titration may benefit from an individualized approach. Patients with diffuse, recruitable injury and severe hypoxemia may benefit from higher PEEP, whereas those with focal consolidation, low recruitability, or circulatory instability may be harmed by overdistension or reduced venous return. Third, respiratory rate deserves more attention than it often receives. It is frequently adjusted to correct PaCO_2_, but very high rates may increase dynamic mechanical load and worsen air trapping or dyssynchrony. Fourth, adjunctive therapies may be viewed as markers of clinical context as well as interventions. Corticosteroids and neuromuscular blockers may be considered in selected severe CAP, ARDS, or shock phenotypes, but their use is best guided by a documented indication, early reassessment, and careful monitoring for infection, hyperglycemia, weakness, and prolonged ventilation.

Our findings also highlight gaps for future research. Prospective studies should capture corticosteroid agent, dose, timing, and duration; neuromuscular blocker dosing strategy; sedation depth; ventilator asynchrony; prone positioning; and objective measures of recruitability. They should distinguish focal pneumonia, diffuse ARDS-like injury, and mixed infections, and should evaluate whether physiology-guided targets improve outcomes beyond standard protective ventilation. Since SP is not a single phenotype, trials that enroll all SP patients without stratification may dilute benefit in responsive subgroups and obscure harm in others.

Strengths of this study include the large sample, daily time-dependent exposure assessment, adjustment for evolving severity, and subgroup analyses across pathogen, oxygenation, and host immune status. The design also allowed ventilation mode-specific modeling, reducing direct comparison of variables that are not measured equivalently under VCV and PCV. Beyond these methodological strengths, the identification of specific non-linear thresholds for VT/kg and respiratory rate provides clinically interpretable guidance for bedside titration. Collectively, these strengths support the validity of our findings and provide a foundation for future prospective studies evaluating physiology-guided ventilation strategies in severe pneumonia.

Limitations remain substantial. The single-center retrospective design limits generalizability and leaves residual confounding. Treatment details for corticosteroids and neuromuscular blockers were unavailable. VT was unavailable in PCV, plateau pressure in VCV, and uniform application of the inspiratory hold maneuver across PCV recordings could not be confirmed. Polynomial modeling captures only selected non-linear shapes, although RCS sensitivity analyses yielded consistent results (Supplementary Figure S6). Subgroup analyses were underpowered and vulnerable to multiple testing, and mixed infections limited pathogen-specific interpretation; sedation depth, patient-ventilator asynchrony, recruitability, and long-term outcomes were not assessed. We did not distinguish CAP, HAP, or VAP due to retrospective data limitations, but we addressed pathogen-related heterogeneity through stratified subgroup analyses. The fungal detection rate in our cohort (49.4%) was high, reflecting the referral nature of our tertiary center and the high proportion of immunocompromised patients; however, we cannot definitively distinguish colonization from true infection, and the limited subgroup sample sizes precluded non-linear modeling in these subgroups. Accordingly, our findings—particularly for respiratory rate—should be generalized cautiously to settings with lower fungal prevalence. Finally, our cohort was predominantly male (68.95%), which may limit generalizability to female patients; sex differences in chemoreflex sensitivity during recovery from lung injury have been reported [59], and whether our findings apply equally to female patients requires further investigation in sex-balanced cohorts.

## 5. Conclusions

In patients with SP receiving invasive mechanical ventilation, ventilatory settings were generally within protective ranges. In primary time-dependent models, higher PEEP, systemic glucocorticoid use, and neuromuscular blocker use were associated with higher ICU mortality, but these associations were not causal and may reflect confounding by indication, disease severity, and other residual confounders. VT/kg and respiratory rate showed non-linear associations with higher mortality at extremes whereas the lowest risk was observed at VT/kg 6–8 mL/kg and respiratory rate 18–26 breaths/min, and [(4 × ΔP) + RR] showed a non-linear association in sensitivity analyses. Optimizing ventilatory parameters within these identified ranges may help minimize ventilator-induced lung injury, a distinct and modifiable contributor to mortality in SP patients. These findings suggest that physiology-guided ventilation and selective use of adjunctive therapies may be reasonable approaches, but prospective studies are needed to define individualized targets, test protocolized decision pathways, and clarify causal effects across distinct SP phenotypes in multicenter cohorts with standardized protocols.

## Figures and Tables

**Table 1:** Baseline characteristics, clinical management, and laboratory parameters in ICU survivors versus non-survivors.

Variables	Total (*n* = 2480)	Group		Test Statistic (t/χ^2^/U)	*p*-Value
		ICU Survivors (*n* = 1532)	ICU Non-Survivors (*n* = 948)		
Demographic characteristics					
Sex, *n* (%)				0.32	0.571
Male	1710 (68.95)	1050 (68.54)	660 (69.62)		
Female	770 (31.05)	482 (31.46)	288 (30.38)		
Age (years), mean (SD)	63.28 (16.98)	61.67 (17.3)	65.88 (16.14)	−6.14	<0.001
Body weight (kg), mean (SD)	60.31 (12.88)	60.75 (13.33)	59.56 (12.06)	1.83	0.068
Body height (cm), mean (SD)	164.13 (7.87)	164.09 (7.67)	164.19 (8.21)	−0.26	0.792
Admission department, *n* (%)				0.305	0.859
Emergency	866 (34.92)	535 (34.92)	331 (34.92)		
Internal Medicine	1513 (61.01)	932 (60.84)	581 (61.29)		
Surgery	101 (4.07)	65 (4.24)	36 (3.8)		
Comorbidities					
Chronic pulmonary disease, *n* (%)				8.94	0.003
No	1600 (64.52)	1023 (66.78)	577 (60.86)		
Yes	880 (35.48)	509 (33.22)	371 (39.14)		
Hypertension, *n* (%)				0.71	0.398
No	1512 (60.97)	944 (61.62)	568 (59.92)		
Yes	968 (39.03)	588 (38.38)	380 (40.08)		
Cardiac disease, *n* (%)				2.12	0.145
No	2029 (81.81)	1267 (82.7)	762 (80.38)		
Yes	451 (18.19)	265 (17.3)	186 (19.62)		
Neurological disease, *n* (%)				1.72	0.189
No	2024 (81.61)	1238 (80.81)	786 (82.91)		
Yes	456 (18.39)	294 (19.19)	162 (17.09)		
Liver disease, *n* (%)				2.48	0.115
No	2396 (96.61)	1487 (97.06)	909 (95.89)		
Yes	84 (3.39)	45 (2.94)	39 (4.11)		
Chronic kidney disease (CKD), *n* (%)				3.06	0.080
No	2211 (89.15)	1379 (90.01)	832 (87.76)		
Yes	269 (10.85)	153 (9.99)	116 (12.24)		
Diabetes mellitus, *n* (%)				0.16	0.692
No	1807 (72.86)	1112 (72.58)	695 (73.31)		
Yes	673 (27.14)	420 (27.42)	253 (26.69)		
Immunocompromised status, *n* (%)				33.36	<0.001
No	1510 (60.89)	1001 (65.34)	509 (53.69)		
Yes	970 (39.11)	531 (34.66)	439 (46.31)		
Pneumonia pathogen					
Bacterial infection, *n* (%)				7.12	0.008
No	250 (10.08)	135 (8.81)	115 (12.13)		
Yes	2230 (89.92)	1397 (91.19)	833 (87.87)		
Viral infection, *n* (%)				7.06	0.008
No	1794 (72.34)	1137 (74.22)	657 (69.3)		
Yes	686 (27.66)	395 (25.78)	291 (30.7)		
Fungal infection, *n* (%)				40.22	<0.001
No	1255 (50.6)	852 (55.61)	403 (42.51)		
Yes	1225 (49.4)	680 (44.39)	545 (57.49)		
Clinical management					
Systemic glucocorticoid, *n* (%)				65.71	<0.001
No	828 (33.39)	604 (39.43)	224 (23.63)		
Yes	1652 (66.61)	928 (60.57)	724 (76.37)		
Neuromuscular blocker, *n* (%)				77.05	<0.001
No	1853 (74.72)	1237 (80.74)	616 (64.98)		
Yes	627 (25.28)	295 (19.26)	332 (35.02)		
Ventilator settings on admission					
Initial ventilator mode, *n* (%)				15.69	0.003
VCV	1787 (72.52)	1100 (72.32)	687 (72.85)		
PCV	513 (20.82)	299 (19.66)	214 (22.69)		
SIMV	112 (4.55)	85 (5.59)	27 (2.86)		
PSV	41 (1.66)	31 (2.04)	10 (1.06)		
Other	11 (0.45)	6 (0.39)	5 (0.53)		
Initial respiratory rate (RR, breaths/min), mean (SD)	21.75 (6.28)	21.39 (6.19)	22.33 (6.39)	−3.64	<0.001
Initial PEEP (cmH_2_O), median (IQR)	6.00 (5.00, 8.00)	5.00 (5.00, 8.00)	8.00 (5.00, 10.00)	594,074.00	<0.001
Initial FiO_2_ (%), mean (SD)	67.44 (23.89)	63.92 (23)	73.14 (24.22)	−9.38	<0.001
Initial tidal volume per body weight (VT/kg, mL/kg), mean (SD)	6.98 (1.20)	7.02 (1.18)	6.91 (1.24)	1.69	0.091
Initial driving pressure (ΔP, cmH_2_O), mean (SD)	13.43 (3.26)	13.20 (2.82)	13.80 (3.84)	−2.12	0.034
Initial (4 × ΔP) + RR, mean (SD)	75.94 (15.02)	74.85 (12.88)	77.71 (17.85)	−2.18	0.030
Initial minute ventilation (VE, L/min), mean (SD)	9.32 (2.97)	9.16 (2.89)	9.58 (3.07)	−3.01	0.003
Initial ventilatory ratio (VR), mean (SD)	1.86 (0.76)	1.79 (0.71)	1.99 (0.82)	−4.67	<0.001
Severity of illness					
Total SOFA score, mean (SD)	10.39 (3.25)	9.65 (2.93)	11.58 (3.39)	−14.49	<0.001
Non-pulmonary SOFA score, median (IQR)	7.00 (5.00, 9.00)	6.00 (5.00, 8.00)	8.00 (6.00, 10.00)	524,293.50	<0.001
SOFA score-central nervous system, mean (SD)	3.63 (0.70)	3.55 (0.77)	3.76 (0.53)	−8.21	<0.001
SOFA score-cardiovascular, median (IQR)	1.00 (0.00, 4.00)	1.00 (0.00, 4.00)	1.00 (0.00, 4.00)	619,747.00	<0.001
SOFA score-respiration, mean (SD)	3.03 (0.82)	2.89 (0.82)	3.27 (0.75)	−11.67	<0.001
SOFA score-coagulation, median (IQR)	1.00 (0.00, 2.00)	0.00 (0.00, 2.00)	1.00 (0.00, 2.00)	605,767.50	<0.001
SOFA score-liver, median (IQR)	0.00 (0.00, 0.00)	0.00 (0.00, 0.00)	0.00 (0.00, 0.00)	697,753.50	0.101
SOFA score-renal, median (IQR)	0.00 (0.00, 1.00)	0.00 (0.00, 1.00)	0.00 (0.00, 2.00)	627,266.50	<0.001
Septic shock, *n* (%)				9.32	0.002
No	1880 (75.81)	1193 (77.87)	687 (72.47)		
Yes	600 (24.19)	339 (22.13)	261 (27.53)		
Gas exchange					
Initial partial pressure of oxygen (PaO_2_, mmHg), median (IQR)	84.60 (69.70, 108.15)	87.50 (72.30, 114.40)	79.55 (67.68, 101.83)	601,248.50	<0.001
Initial PaO_2_/FiO_2_ ratio (P/F ratio), median (IQR)	173.06 (120.33, 243.04)	190.33 (136.34, 260.38)	143.93 (102.41, 208.89)	664,250.00	<0.001
Initial partial pressure of carbon dioxide (PaCO_2_, mmHg), mean (SD)	46.4 (12.74)	45.01 (11.34)	48.65 (14.43)	−6.08	<0.001
Initial arterial pH, mean (SD)	7.39 (0.08)	7.41 (0.07)	7.37 (0.08)	9.57	<0.001
Initial lactate (mmol/L), median (IQR)	1.40 (1.10, 1.90)	1.30 (1.10, 1.80)	1.60 (1.30, 2.20)	386,107.50	<0.001
Laboratory examinations					
C-reactive protein (CRP), median (IQR)	103.00 (55.45, 175.00)	95.60 (48.95, 164.00)	118.00 (67.30, 191.00)	352,651.50	<0.001
Interleukin-6 (IL-6), median (IQR)	82.19 (35.00, 229.38)	67.43 (29.39, 171.00)	121.00 (43.80, 370.10)	286,351.00	<0.001
Procalcitonin (PCT), median (IQR)	1.06 (0.33, 4.30)	0.86 (0.26, 3.35)	1.48 (0.50, 5.90)	439,937.00	<0.001
White blood cell count (WBC), median (IQR)	10.98 (7.46, 15.61)	10.52 (7.38, 14.87)	11.60 (7.59, 16.75)	581,088.00	<0.001
Absolute neutrophil count (ANC), median (IQR)	9.50 (6.29, 13.94)	9.03 (6.16, 13.16)	10.24 (6.60, 15.20)	566,559.50	<0.001
Absolute lymphocyte count (ALC), median (IQR)	0.56 (0.33, 0.90)	0.61 (0.37, 0.97)	0.49 (0.30, 0.80)	732,588.50	0.711
Hemoglobin (Hb), mean (SD)	95.72 (24.44)	96.4 (24.17)	94.62 (24.85)	1.70	0.089
Platelet count (PLT), median (IQR)	140.00 (84.00, 214.00)	151.50 (94.00, 223.00)	121.00 (67.25, 196.50)	736,168.00	0.564
D-dimer, median (IQR)	7.00 (3.22, 13.67)	6.34 (3.07, 12.49)	8.03 (3.39, 15.79)	498,239.50	<0.001
Albumin, mean (SD)	29.19 (5.18)	29.62 (4.99)	28.51 (5.41)	4.99	<0.001
B-type natriuretic peptide (BNP), median (IQR)	1860.00 (643.50, 5358.00)	1500.00 (533.25, 4398.75)	2519.00 (979.00, 7020.00)	423,972.00	<0.001
Creatinine (Cr), median (IQR)	79.00 (53.00, 138.00)	72.00 (50.00, 124.00)	92.00 (60.25, 178.75)	537,706.50	<0.001
Total bilirubin (TBIL), median (IQR)	10.60 (7.00, 17.50)	10.60 (7.00, 17.15)	10.70 (6.90, 18.10)	654,591.00	<0.001

Data are presented as mean (SD), median (IQR), or *n* (%), as appropriate. For comparisons between ICU survivors and non-survivors, continuous variables with normal distribution were analyzed using the independent two-sample *t*-test; skewed continuous variables were analyzed using the Mann–Whitney U test; categorical variables were analyzed using the chi-square (χ^2^) test. For pathogen variables, “Yes” indicates detection of the specified pathogen type, regardless of co-infection with other pathogens. All tests were two-sided. t denotes Welch’s *t*-test; U denotes Mann–Whitney U statistic.

**Table 2:** Prognostic outcomes and complications in ICU survivors versus non-survivors.

Variables	Total (*n* = 2480)	Group		Test Statistic (χ^2^/U)	*p*-Value
		ICU Survivors (*n* = 1532)	ICU Non-Survivors (*n* = 948)		
28-day mortality, *n* (%)				1858.28	<0.001
No	1632 (65.81)	1503 (98.11)	129 (13.61)		
Yes	848 (34.19)	29 (1.89)	819 (86.39)		
In-hospital mortality, *n* (%)				2233.56	<0.001
No	1470 (59.27)	1470 (95.95)	0 (0)		
Yes	1010 (40.73)	62 (4.05)	948 (100)		
Successful extubation, *n* (%)				692.01	<0.001
No	1456 (58.71)	586 (38.25)	870 (91.77)		
Yes	1024 (41.29)	946 (61.75)	78 (8.23)		
Tracheostomy, *n* (%)				26.99	<0.001
No	1716 (69.19)	1002 (65.4)	714 (75.32)		
Yes	764 (30.81)	530 (34.6)	234 (24.68)		
Length of hospital stay (days), median (IQR)	22.00 (13.00, 35.00)	26 (16, 40)	16.00 (9.75, 28.00)	434,197.00	<0.001
Length of ICU stay (days), median (IQR)	13.78 (7.93, 22.84)	14.70 (8.87, 23.99)	12.55 (6.87, 21.02)	611,541.00	<0.001
Ventilator-free days at day 28 (days), median (IQR)	9.35 (0.00, 21.04)	19.31 (12.54, 23.28)	0.00 (0.00, 0.00)	63,395.50	<0.001
Complication, *n* (%)					
Barotrauma				9.64	0.002
No	2303 (92.86)	1442 (94.13)	861 (90.82)		
Yes	177 (7.14)	90 (5.87)	87 (9.18)		
Septic Shock				264.07	<0.001
No	1921 (77.46)	1351 (88.19)	570 (60.13)		
Yes	559 (22.54)	181 (11.81)	378 (39.87)		
Acute kidney injury (AKI)				95.99	<0.001
No	2114 (85.24)	1390 (90.73)	724 (76.37)		
Yes	366 (14.76)	142 (9.27)	224 (23.63)		

Data are presented as median (IQR) or *n* (%), as appropriate. Categorical variables were analyzed using the chi-square (χ^2^) test; continuous variables were analyzed using the Mann–Whitney U test. All tests were two-sided. U denotes the Mann–Whitney U statistic.

**Table 3:** Multivariable linear time-dependent Cox regression models for ICU mortality—volume-controlled ventilation group.

Variables	Model 1 (VT/kg)			Model 2 (VE)			Model 3 (VR)		
	β	HR (95% CI)	*p*-Value	β	HR (95% CI)	*p*-Value	β	HR (95% CI)	*p*-Value
Systemic glucocorticoid	0.339	1.40 (1.20–1.64)	<0.001	0.340	1.40 (1.20–1.64)	<0.001	0.343	1.41 (1.21–1.64)	<0.001
Neuromuscular blocker	0.427	1.53 (1.32–1.78)	<0.001	0.432	1.54 (1.33–1.79)	<0.001	0.445	1.56 (1.35–1.81)	<0.001
Respiratory rate (RR)	0.002	1.00 (0.99–1.01)	0.183	—			—		
PEEP	0.036	1.04 (1.02–1.05)	<0.001	0.036	1.04 (1.02–1.05)	<0.001	0.036	1.04 (1.02–1.05)	<0.001
VT/kg	−0.003	1.00 (0.99–1.00)	0.082	—			—		
VE	—			0.0003	1.00 (0.99–1.01)	0.957	—		
VR	—			—			−0.003	1.00 (0.97–1.03)	0.843

Data are presented as β coefficient (regression coefficient), *p*-value, and hazard ratio (HR) with 95% confidence interval (CI). HRs were calculated per 1-unit increase for continuous variables unless otherwise specified. All models were adjusted for age, non-pulmonary SOFA score, septic shock, chronic lung disease, heart disease, neurologic disease, liver disease, chronic kidney disease, immunocompromised status, bacterial/viral/fungal infection, mean arterial pressure, heart rate, fluid balance, PaO_2_/FiO_2_ ratio, PaCO_2_, pH, lactate, CRP, IL-6, PCT, BNP, albumin, absolute neutrophil count, absolute lymphocyte count, hemoglobin, D-dimer, creatinine, platelet count, and time (day). Time-dependent covariates were handled using the time-varying Cox regression model with repeated measures. Analyses were stratified by ventilation mode (volume-controlled vs. pressure-controlled). VT/kg, tidal volume per kg predicted body weight; VE, minute ventilation; VR, ventilatory ratio; RR, respiratory rate; PEEP, positive end-expiratory pressure. —, not included in the model.

**Table 4:** Multivariable linear time-dependent Cox regression models for ICU mortality—pressure-controlled ventilation group.

Variables	Model 4 (ΔP)			Model 5 (4 × ΔP + RR)		
	β	HR (95% CI)	*p*-Value	β	HR (95% CI)	*p*-Value
Systemic glucocorticoid	0.338	1.40 (1.20–1.64)	<0.001	0.337	1.40 (1.20–1.63)	<0.001
Neuromuscular blocker	0.428	1.53 (1.32–1.78)	<0.001	0.430	1.54 (1.32–1.78)	<0.001
Respiratory rate (RR)	0.002	1.00 (1.00–1.01)	0.192	—		
PEEP	0.036	1.04 (1.02–1.05)	<0.001	0.036	1.04 (1.02–1.05)	<0.001
ΔP	0.001	1.00 (1.00–1.00)	0.387	—		
(4 × ΔP + RR)	—			0.0006	1.00 (1.00–1.00)	0.148

Data are presented as β coefficient (regression coefficient), *p*-value, and hazard ratio (HR) with 95% confidence interval (CI). HRs were calculated per 1-unit increase for continuous variables unless otherwise specified. All models were adjusted for the same set of covariates as Table 3. Time-dependent covariates were handled using the time-varying Cox regression model with repeated measures. Analyses were stratified by ventilation mode (volume-controlled vs. pressure-controlled). ΔP, driving pressure; RR, respiratory rate; PEEP, positive end-expiratory pressure. —, not included in the model.

## Data Availability

The data that support the findings of this study are available from the corresponding author upon reasonable request due to privacy and ethical restrictions.

## Supplementary Materials

The following supporting information can be downloaded at: https://www.mdpi.com/article/10.3390/jcm15187171/s1, Figure S1: Flowchart of modeling workflow; Figure S2: Temporal trends of 14 physiological and laboratory parameters over the first 30 days in ICU survivors versus non-survivors; Table S1: Summary of ventilatory parameters and gas exchange variables over the first 10 days in ICU survivors versus ICU non-survivors; Table S2: Percentage of missing data; Table S3: Predictor Variables for Multiple Imputation; Table S4: Test of proportional hazards assumption for each ventilatory exposure; Tables S5–S9: Multivariable time-dependent Cox regression models for ICU mortality; Tables S10–S14: complete-case sensitivity analysis without imputation; Table S15: Sensitivity analysis: multivariable linear time-dependent Cox regression model for ICU mortality excluding adjustment covariates with >30% missingness; Table S16: Sensitivity analysis: multivariable Cox regression for ICU mortality using baseline (day 0) data only; Table S17: Sensitivity analysis: multivariable linear time-dependent Cox regression model for ICU mortality excluding patients with septic shock at baseline; Figures S3–S5: Non-linear associations of PEEP with ICU mortality across additional multivariable models; Figure S6: Non-linear associations between ventilatory parameters and ICU mortality via restricted cubic splines (RCS) in time-dependent Cox models; Tables S18–S37: Complete results of subgroup analyses for the association between parameters and ICU mortality—multivariable time-dependent Cox regression models stratified by viral infection, fungal infection, hypoxemia severity, and immunocompromised status.

## References

[1] Mandell L.A., Wunderink R.G., Anzueto A., Bartlett J.G., Campbell G.D., Dean N.C., Dowell S.F., File T.M., Musher D.M., Niederman M.S. (2007). Infectious Diseases Society of America/American Thoracic Society consensus guidelines on the management of community-acquired pneumonia in adults. Clin. Infect. Dis..

[2] Su X., He B.C. (2025). Rapid and accurate diagnosis of severe pneumonia: Similarities and differences between SCAP and HAP/VAP. Zhonghua Jie He He Hu Xi Za Zhi.

[3] Martin-Loeches I., Torres A., Nagavci B., Aliberti S., Antonelli M., Bassetti M., Bos L.D., Chalmers J.D., Derde L., de Waele J. (2023). ERS/ESICM/ESCMID/ALAT guidelines for the management of severe community-acquired pneumonia. Intensive Care Med..

[4] Japanese Respiratory Society (2024). The JRS guidelines for the management of community-acquired pneumonia in adults 2024. Respir. Investig..

[5] (2020). GBD 2019 Diseases and Injuries Collaborators. Global burden of 369 diseases and injuries in 204 countries and territories, 1990–2019: A systematic analysis for the Global Burden of Disease Study 2019. Lancet.

[6] Ramirez J.A., Wiemken T.L., Peyrani P., Arnold F.W., Kelley R., Mattingly W.A., Nakamatsu R., Pena S., Guinn B.E., Furmanek S.P. (2017). Adults hospitalized with pneumonia in the United States: Incidence, epidemiology, and mortality. Clin. Infect. Dis..

[7] Cavallazzi R., Furmanek S., Arnold F.W., Beavin L.A., Wunderink R.G., Niederman M.S., Ramirez J.A. (2020). The burden of community-acquired pneumonia requiring admission to ICU in the United States. Chest.

[8] Cavallazzi R., Ramirez J.A. (2024). Definition, epidemiology, and pathogenesis of severe community-acquired pneumonia. Semin Respir. Crit. Care Med..

[9] Slutsky A.S., Ranieri V.M. (2013). Ventilator-induced lung injury. N. Engl. J. Med..

[10] Cao Y., Xie L.X., Xiao K. (2026). Spatiotemporal immune evolution in severe pneumonia and its implications for precision intervention. Guoji Huxi Zazhi.

[11] Weng J.Z., Li Y.M. (2025). Immunomodulatory therapy for severe pneumonia: A double-edged sword determining success or failure. Zhonghua Jie He He Hu Xi Za Zhi.

[12] Gattinoni L., Coppola S., Cressoni M., Busana M., Rossi S., Chiumello D. (2020). COVID-19 does not lead to a “typical” acute respiratory distress syndrome. Am. J. Respir. Crit. Care Med..

[13] Chinese Thoracic Society, Chinese Association for Lung Cancer and Respiratory Diseases (2026). Chinese Expert consensus on the diagnosis and treatment of severe pneumonia in immunocompromised hosts. Zhonghua Jie He He Hu Xi Za Zhi.

[14] Di Pasquale M.F., Sotgiu G., Gramegna A., Radovanovic D., Terraneo S., Reyes L.F., Rupp J., González Del Castillo J., Blasi F., Aliberti S. (2019). Prevalence and etiology of community-acquired pneumonia in immunocompromised patients. Clin. Infect. Dis..

[15] Ranieri V.M., Rubenfeld G.D., Thompson B.T., Ferguson N.D., Caldwell E., Fan E., Camporota L., Slutsky A.S. (2012). Acute respiratory distress syndrome: The Berlin Definition. JAMA.

[16] Torres A., Cilloniz C., Niederman M.S., Menéndez R., Chalmers J.D., Wunderink R.G., van der Poll T. (2021). Pneumonia. Nat. Rev. Dis. Primers.

[17] Sinha P., Calfee C.S., Beitler J.R., Soni N., Ho K., Matthay M.A., Kallet R.H. (2019). Physiologic analysis and clinical performance of the ventilatory ratio in acute respiratory distress syndrome. Am. J. Respir. Crit. Care Med..

[18] Costa E.L.V., Slutsky A.S., Brochard L.J., Brower R., Serpa-Neto A., Cavalcanti A.B., Mercat A., Meade M., Morais C.C.A., Goligher E. (2021). Ventilatory variables and mechanical power in patients with acute respiratory distress syndrome. Am. J. Respir. Crit. Care Med..

[19] Peduzzi P., Concato J., Feinstein A.R., Holford T.R. (1995). Importance of events per independent variable in proportional hazards regression analysis. II. Accuracy and precision of regression estimates. J. Clin. Epidemiol..

[20] Royston P., Sauerbrei W. (2008). Multivariable Model-Building: A Pragmatic Approach to Regression Analysis Based on Fractional Polynomials for Modelling Continuous Variables.

[21] Jann B. (2021). Relative distribution analysis in Stata. Stata J..

[22] Kamra K., Karpuk N., Adam R., Zucker I.H., Schultz H.D., Wang H.-J. (2022). Time-dependent alteration in the chemoreflex post-acute lung injury. Front. Physiol..

[23] Neto A.S., Barbas C.S.V., Simonis F.D., Artigas-Raventós A., Canet J., Determann R.M., Anstey J., Hedenstierna G., Hemmes S.N.T., Hermans G. (2016). Epidemiological characteristics, practice of ventilation, and clinical outcome in patients at risk of acute respiratory distress syndrome in intensive care units from 16 countries (PRoVENT): An international, multicentre, prospective study. Lancet Respir. Med..

[24] Hochberg C.H., Emetu S.A., Psoter K.J., Sahetya S.K., Kerlin M.P., Iwashyna T.J., Hager D.N., Eakin M.N. (2026). ICU Implementation Determinants and Delivery of Lung Protective Ventilation: A Multihospital Survey and Cohort Study. CHEST Crit. Care.

[25] Esteban A., Frutos-Vivar F., Muriel A., Ferguson N.D., Peñuelas O., Abraira V., Raymondos K., Rios F., Nin N., Apezteguía C. (2013). Evolution of mortality over time in patients receiving mechanical ventilation. Am. J. Respir. Crit. Care Med..

[26] Brower R.G., Matthay M.A., Morris A., Schoenfeld D., Thompson B.T., Wheeler A. (2000). Ventilation with lower tidal volumes as compared with traditional tidal volumes for acute lung injury and the acute respiratory distress syndrome. N. Engl. J. Med..

[27] Amato M.B.P., Meade M.O., Slutsky A.S., Brochard L., Costa E.L., Schoenfeld D.A., Stewart T.E., Briel M., Talmor D., Mercat A. (2015). Driving pressure and survival in the acute respiratory distress syndrome. N. Engl. J. Med..

[28] Delamarre L., Marini J.J., Guerin C. (2025). Driving pressure as a target for lung protective strategies: From ARDS critically-ill patients to the operating room—Lessons from the IMPROVE-2 Trial. Intensive Care Med..

[29] Gattinoni L., Carlesso E., Cadringher P., Valenza F., Vagginelli F., Chiumello D. (2003). Physical and biological triggers of ventilator-induced lung injury and its prevention. Eur. Respir. J. Suppl..

[30] Gattinoni L., Caironi P., Cressoni M., Chiumello D., Ranieri V.M., Quintel M., Russo S., Patroniti N., Cornejo R., Bugedo G. (2006). Lung recruitment in patients with the acute respiratory distress syndrome. N. Engl. J. Med..

[31] Cavalcanti A.B., Suzumura E.A., Laranjeira L.N., Paisani D.M., Damiani L.P., Guimarães H.P., Romano E.R., Regenga M.M., Taniguchi L.N.T., Teixeira C. (2017). Effect of lung recruitment and titrated positive end-expiratory pressure (PEEP) vs low PEEP on mortality in patients with acute respiratory distress syndrome: A randomized clinical trial. JAMA.

[32] Guerin C. (2011). The preventive role of higher PEEP in treating severely hypoxemic ARDS. Minerva Anestesiol..

[33] Hotchkiss J.R., Blanch L., Murias G., Adams A.B., Olson D.A., Wangensteen O.D., Leo P.H., Marini J.J. (2000). Effects of decreased respiratory frequency on ventilator-induced lung injury. Am. J. Respir. Crit. Care Med..

[34] Vaporidi K., Akoumianaki E., Telias I., Goligher E.C., Brochard L., Georgopoulos D. (2020). Respiratory drive in critically ill patients: Pathophysiology and clinical implications. Am. J. Respir. Crit. Care Med..

[35] Kamra K., Xia Z., Zucker I.H., Schultz H., Wang H. (2025). Chemoreflex function in pulmonary diseases—A review. J. Physiol..

[36] Goligher E.C., Costa E.L.V., Yarnell C.J., Brochard L.J., Stewart T.E., Tomlinson G., Brower R.G., Slutsky A.S., Amato M.P.B. (2021). Effect of lowering VT on mortality in acute respiratory distress syndrome varies with respiratory system elastance. Am. J. Respir. Crit. Care Med..

[37] Simonis F.D., Serpa Neto A., Binnekade J.M., Braber A., Bruin K.C.M., Determann R.M., Goekoop G.-J., Heidt J., Horn J., Innemee G. (2018). Effect of a low vs intermediate tidal volume strategy on ventilator-free days in intensive care unit patients without ARDS: A randomized clinical trial. JAMA.

[38] Nuckton T.J., Alonso J.A., Kallet R.H., Daniel B.M., Pittet J.-F., Eisner M.D., Matthay M.A. (2002). Pulmonary dead-space fraction as a risk factor for death in the acute respiratory distress syndrome. N. Engl. J. Med..

[39] Parada-Gereda H.M., Peña-López L.A., Muñoz-Escudero D.F., Molano-Franco D., Parrilla- Gómez F.J., Grillo C.F., Rodriguez I.J., Masclans J.R. (2026). Ventilatory ratio as a predictor of mortality in acute respiratory distress syndrome: A systematic review and Bayesian meta-analysis of observational studies. Respir. Med..

[40] Jiang L., Chen H., Chang W., Sun Q., Yuan X., Wu Z., Xie J., Liu L., Yang Y. (2025). Time-varying intensity of ventilatory inefficiency and mortality in patients with acute respiratory distress syndrome. Ann. Intensive Care.

[41] Braunsteiner J., Castro L., Wiessner C., Grensemann J., Schroeder M., Burdelski C., Sensen B., Kluge S., Fischer M. (2024). Association Between Dyscapnia, Ventilatory Variables, and Mortality in Patients with Acute Respiratory Distress Syndrome—A Retrospective Cohort Study. J. Intensive Care Med..

[42] Stamatopoulou V., Akoumianaki E., Vaporidi K., Stamatopoulos E., Kondili E., Georgopoulos D. (2024). Driving pressure of respiratory system and lung stress in mechanically ventilated patients with active breathing. Crit. Care.

[43] Yoshida T., Amato M.B.P., Grieco D.L., Chen L., Lima C.A.S., Roldan R., Morais C.C.A., Gomes S., Costa E.L.V., Cardoso P.F.G. (2018). Esophageal manometry and regional transpulmonary pressure in lung injury. Am. J. Respir. Crit. Care Med..

[44] McNamee J.J., Gillies M.A., Barrett N.A., Perkins G.D., Tunnicliffe W., Young D., Bentley A., Harrison D.A., Brodie D., Boyle A.J. (2021). Effect of lower tidal volume ventilation facilitated by extracorporeal carbon dioxide removal vs standard care ventilation on 90-day mortality in patients with acute hypoxemic respiratory failure: The REST randomized clinical trial. JAMA.

[45] Futier E., De Jong A., Cirenei C., Godet T., Jabaudon M., Constantin J.-M., Grillot N., Bouzat P., Henry L., Margetis D. (2025). Personalized driving pressure-guided positive end-expiratory pressure in patients at risk of postoperative respiratory failure (IMPROVE-2): A multicenter, pragmatic, randomized clinical trial. Intensive Care Med..

[46] Dequin P.F., Meziani F., Quenot J.P., Kamel T., Ricard J.-D., Badie J., Reignier J., Heming N., Plantefève G., Souweine B. (2023). Hydrocortisone in severe community-acquired pneumonia. N. Engl. J. Med..

[47] Meduri G.U., Shih M.C., Bridges L., Martin T.J., El-Solh A., Seam N., Davis-Karim A., Umberger R., Anzueto A., Sriram P. (2022). Low-dose methylprednisolone treatment in critically ill patients with severe community-acquired pneumonia. Intensive Care Med..

[48] Angus D.C., REMAP-CAP Investigators (2025). Effect of hydrocortisone on mortality in patients with severe community-acquired pneumonia: The REMAP-CAP Corticosteroid Domain Randomized Clinical Trial. Intensive Care Med..

[49] Pitre T., Pauley E., Chaudhuri D., Saha R., Rudd K.E., Villar J., Berry L.R., Lorenzi E., Hills T., Nichol A. (2025). Corticosteroids for adult patients hospitalised with non-viral community-acquired pneumonia: A systematic review and meta-analysis. Intensive Care Med..

[50] Cheema H.A., Musheer A., Ejaz A., Paracha A.A., Shahid A., Rehman M.E.U., Hermis A.H., Singh H., Duric N., Ahmad F. (2024). Efficacy and safety of corticosteroids for the treatment of community-acquired pneumonia: A systematic review and meta-analysis of randomized controlled trials. J. Crit. Care.

[51] Chaudhuri D., Nei A.M., Rochwerg B., Balk R.A., Asehnoune K., Cadena R., Carcillo J.A., Correa R., Drover K., Esper A.M. (2024). 2024 Focused Update: Guidelines on Use of Corticosteroids in Sepsis, Acute Respiratory Distress Syndrome, and Community-Acquired Pneumonia. Crit. Care Med..

[52] Qadir N., Sahetya S., Munshi L., Summers C., Abrams D., Beitler J., Bellani G., Brower R.G., Burry L., Chen J.-T. (2024). An Update on Management of Adult Patients with Acute Respiratory Distress Syndrome: An Official American Thoracic Society Clinical Practice Guideline. Am. J. Respir. Crit. Care Med..

[53] Fujishima S. (2025). Current corticosteroid therapeutic strategy for community-acquired pneumonia in adults: Indications, dosage, and timing. J. Intensive Care.

[54] Papazian L., Forel J.M., Gacouin A., Penot-Ragon C., Perrin G., Loundou A., Jaber S., Arnal J.-M., Perez D., Seghboyan J.-M. (2010). Neuromuscular blockers in early acute respiratory distress syndrome. N. Engl. J. Med..

[55] Moss M., Huang D.T., Brower R.G., Ferguson N.D., Ginde A.A., Gong M.N., Grissom C.K., Gundel S., Hayden D., Hite R.D. (2019). Early neuromuscular blockade in the acute respiratory distress syndrome. N. Engl. J. Med..

[56] Grasselli G., Calfee C.S., Camporota L., Poole D., Amato M.B.P., Antonelli M., Arabi Y.M., Baroncelli F., Beitler J.R., Bellani G. (2023). ESICM guidelines on acute respiratory distress syndrome: Definition, phenotyping and respiratory support strategies. Intensive Care Med..

[57] Balzer F., Weiß B., Kumpf O., Treskatsch S., Spies C., Wernecke K.-D., Krannich A., Kastrup M. (2015). Early deep sedation is associated with decreased in-hospital and two-year follow-up survival. Crit. Care.

[58] Price D.R., Mikkelsen M.E., Umscheid C.A., Armstrong E.J. (2016). Neuromuscular blocking agents and neuromuscular dysfunction acquired in critical illness: A systematic review and meta-analysis. Crit. Care Med..

[59] Kamra K., Zucker I.H., Schultz H.D., Wang H.-J. (2024). Chemoreflex sensitization occurs in both male and female rats during recovery from acute lung injury. Front. Physiol..

