# Supplementary material for: Ventilatory Parameters and Adjunctive Therapies Associated with ICU Mortality in Severe Pneumonia: A Large Retrospective Cohort Study
Source: J Clin Med. 2026 Sep 15;15(18):7171. doi: 10.3390/jcm15187171 (PMC13607185; doi:10.3390/jcm15187171)
Supplement: Supplementary file 1 [file jcm-15-07171-s001.zip › jcm-4459292-supplementary.pdf]

Supplementary Figure S1. Flowchart of modeling workflow

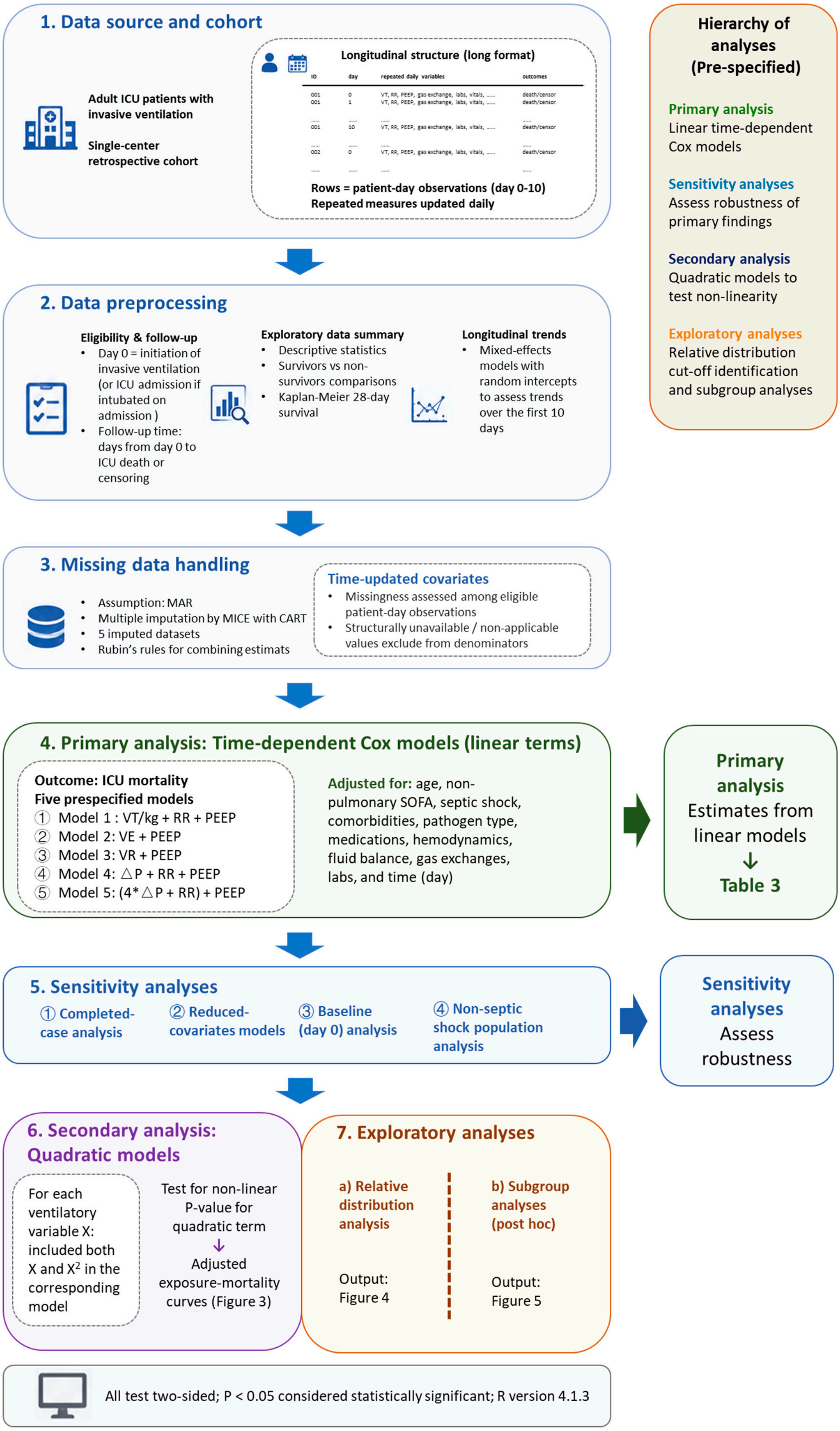

Supplementary Figure S2. Temporal trends of 14 physiological and laboratory parameters over the first 30 days in ICU survivors versus non-survivors.

Data are presented as mean (SD). Panels: (A) MAP (mmHg), (B) HR (beats/min), (C) RR (breaths/min), (D) PEEP (cmH_2O), (E) FiO_2 (%), (F) VT/kg (mL/kg), (G) ΔP (cmH_2O), (H) (4×ΔP+RR), (I) VE (L/min), (J) VR, (K) P/F ratio, (L) $PaCO_2$ (mmHg), (M) arterial pH, (N) lactate (mmol/L). ICU survivors: n=1532; ICU non-survivors: n=948.

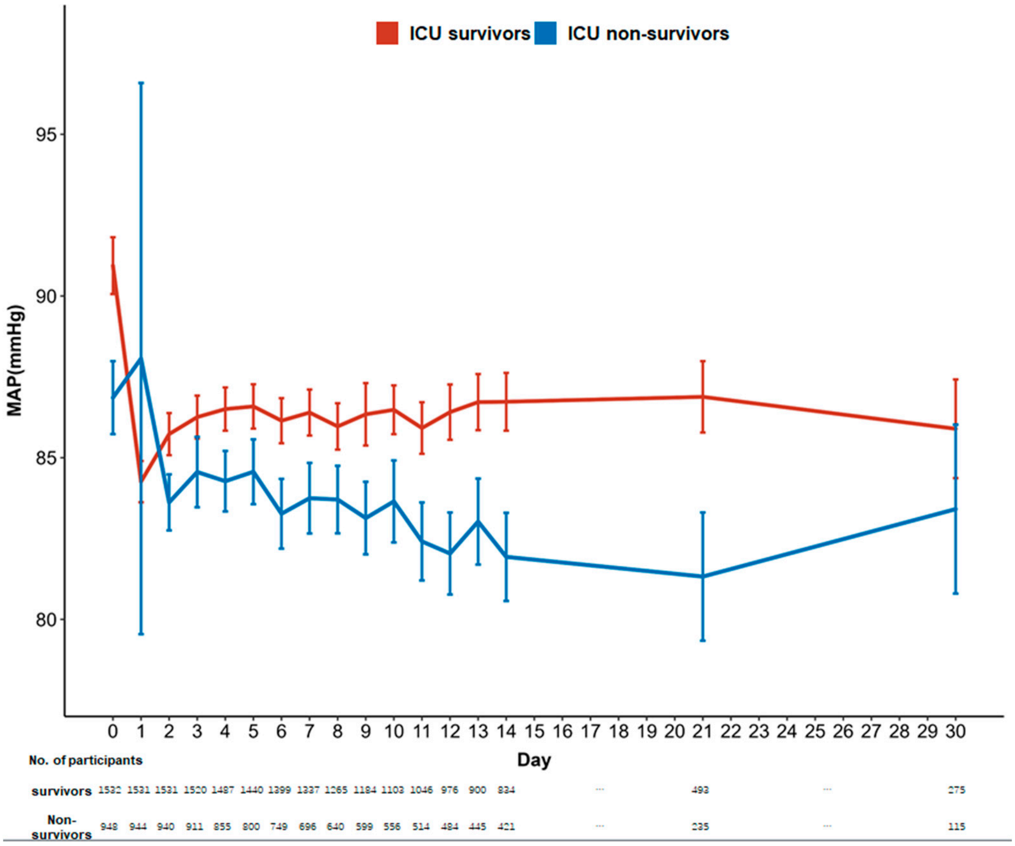

Figure S2A

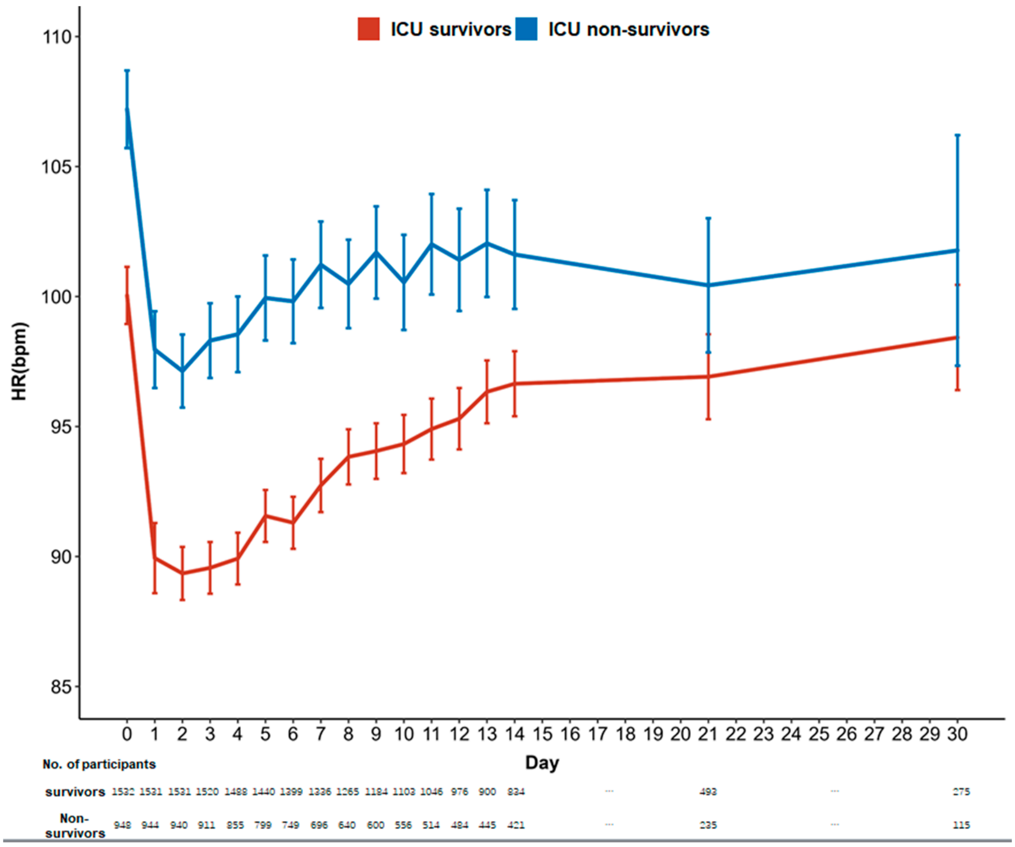

Figure S2B

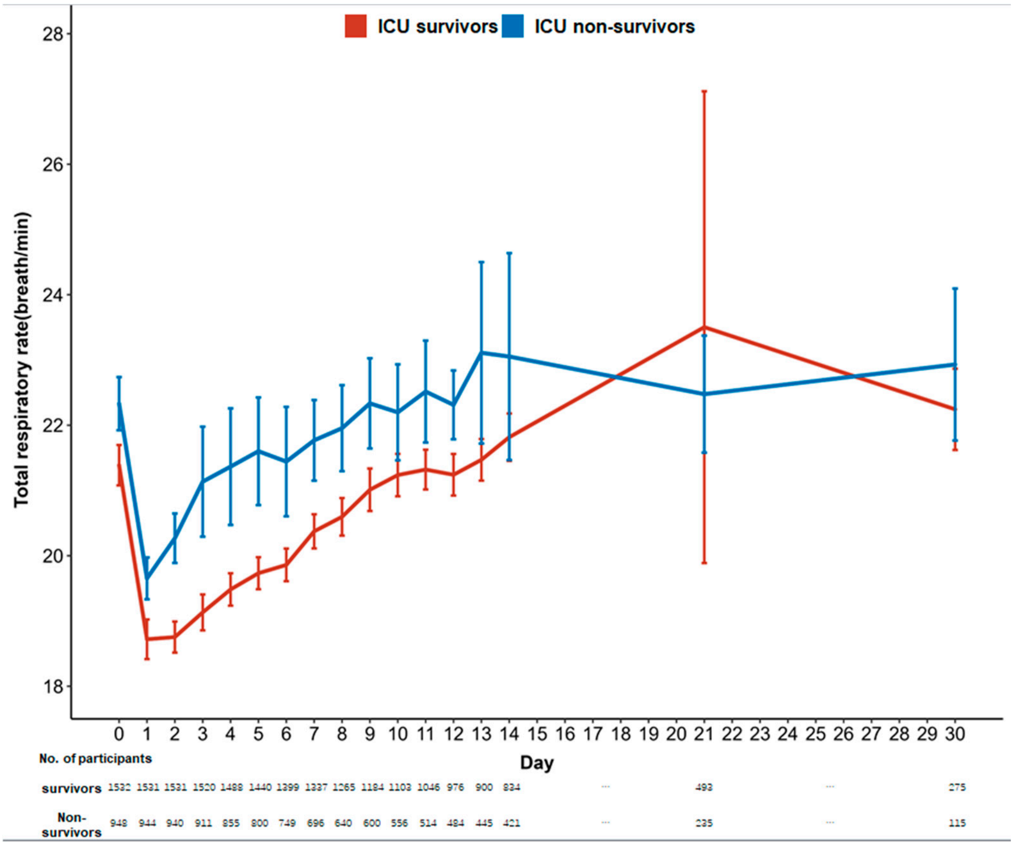

Figure S2C

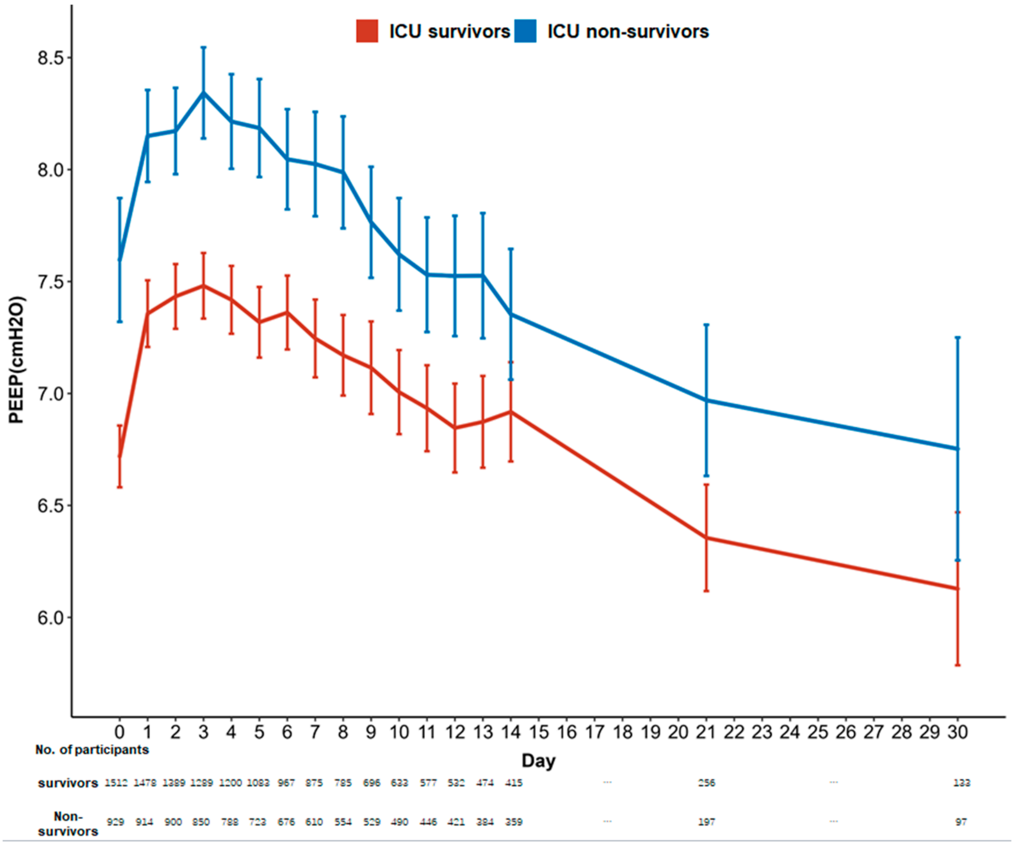

Figure S2D

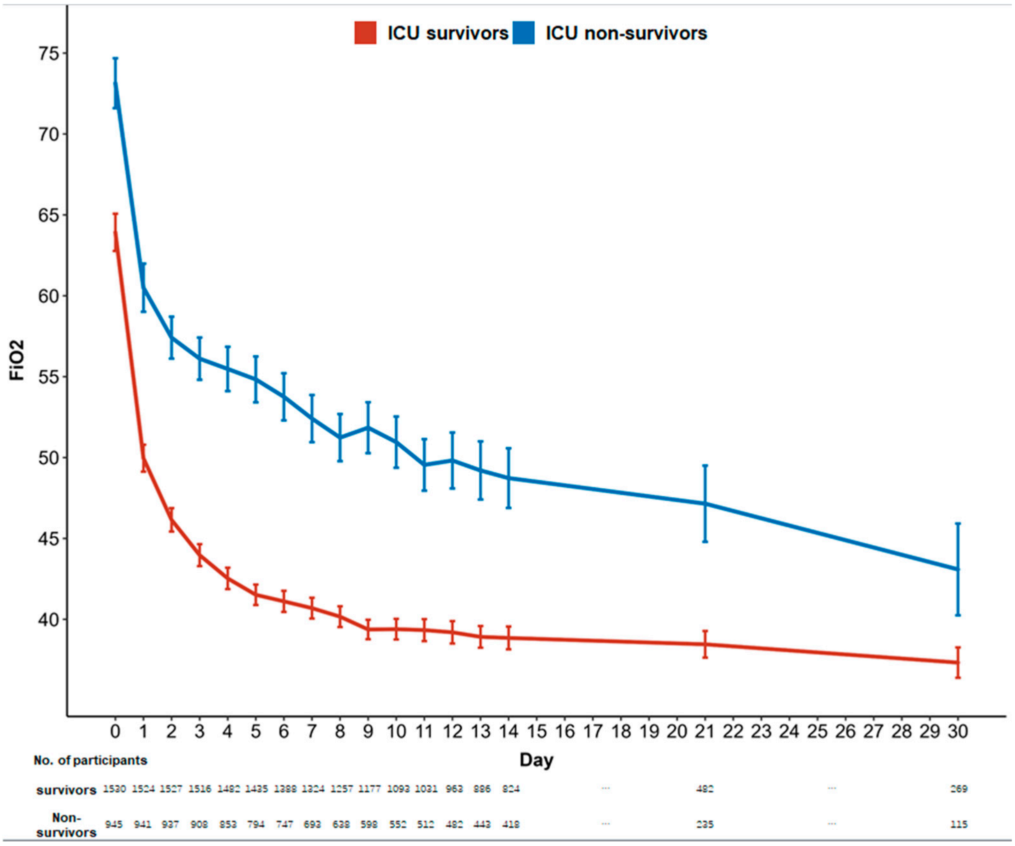

Figure S2E

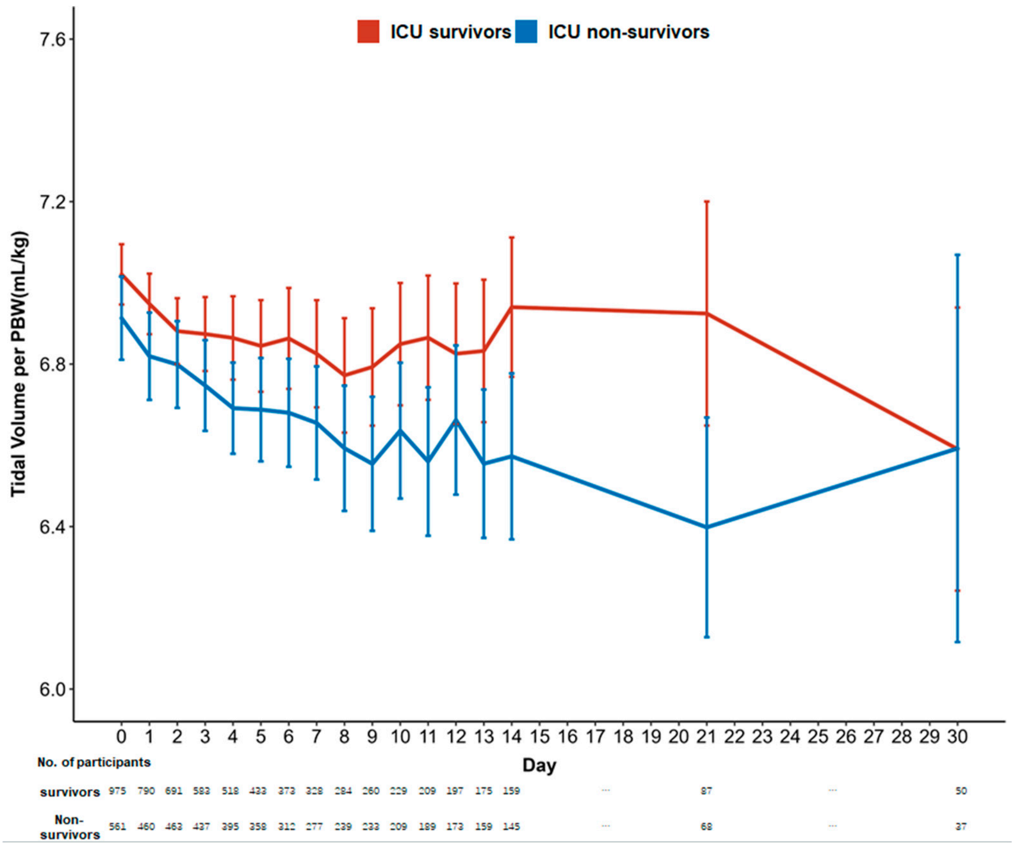

Figure S2F

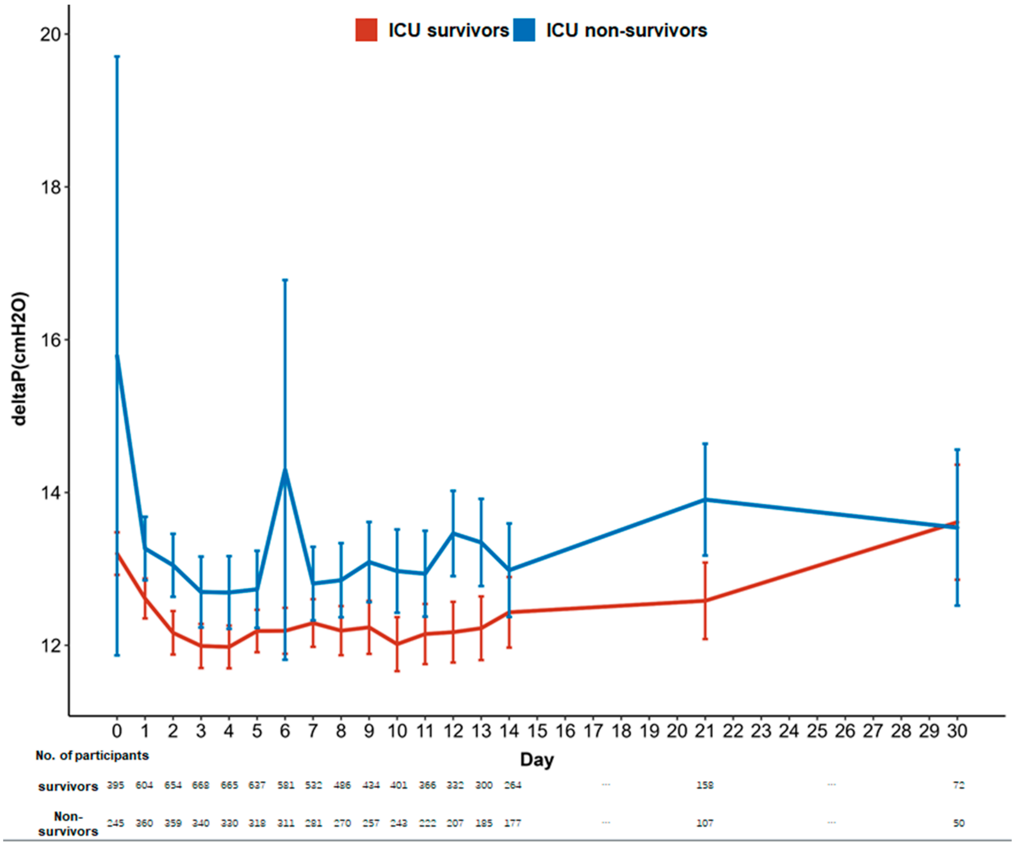

Figure S2G

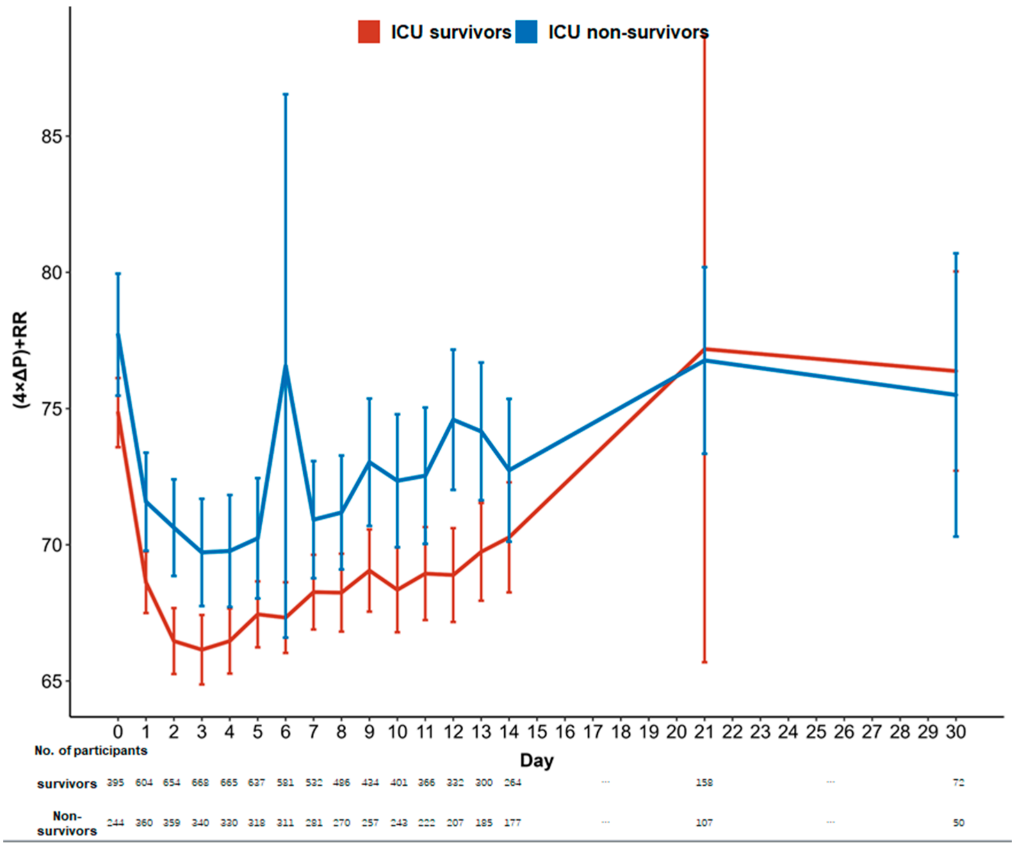

Figure S2H

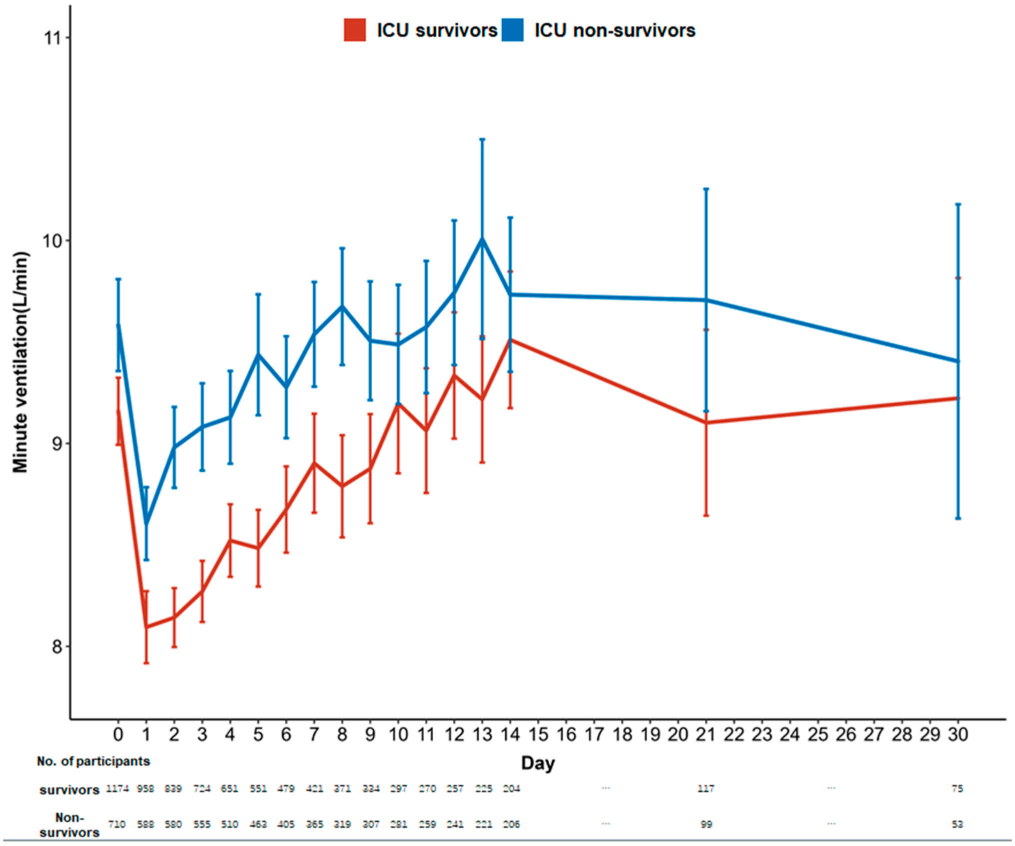

Figure S2I

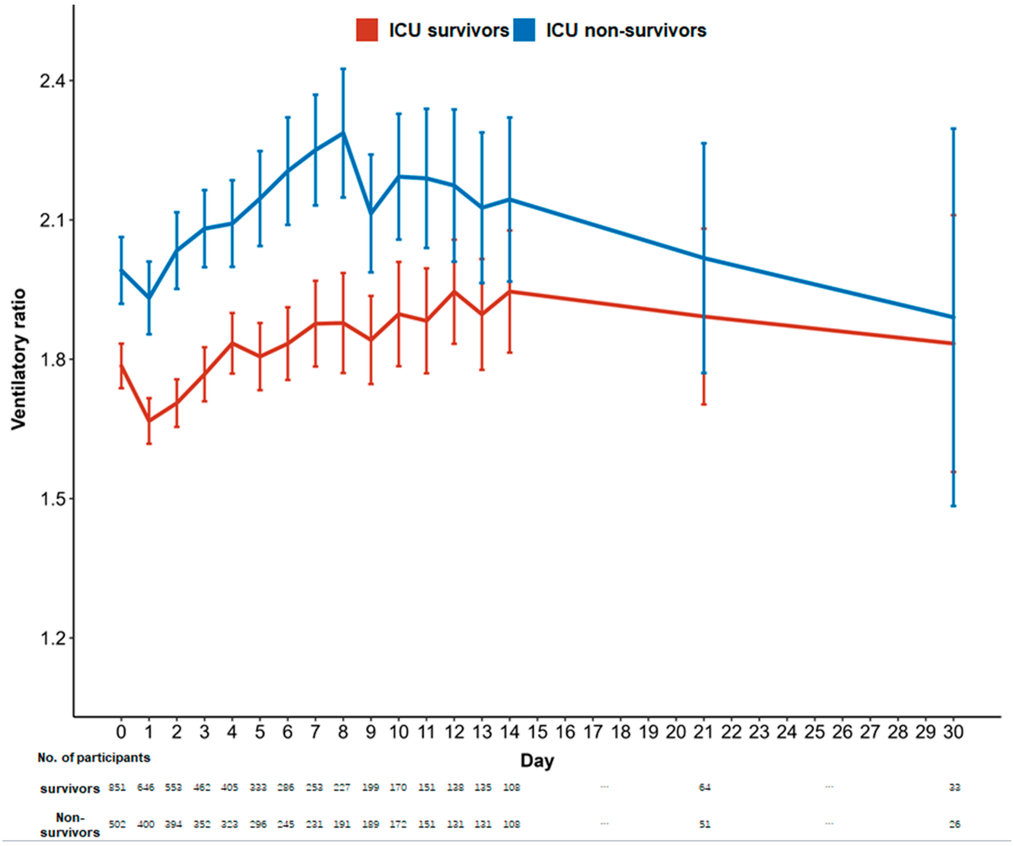

Figure S2J

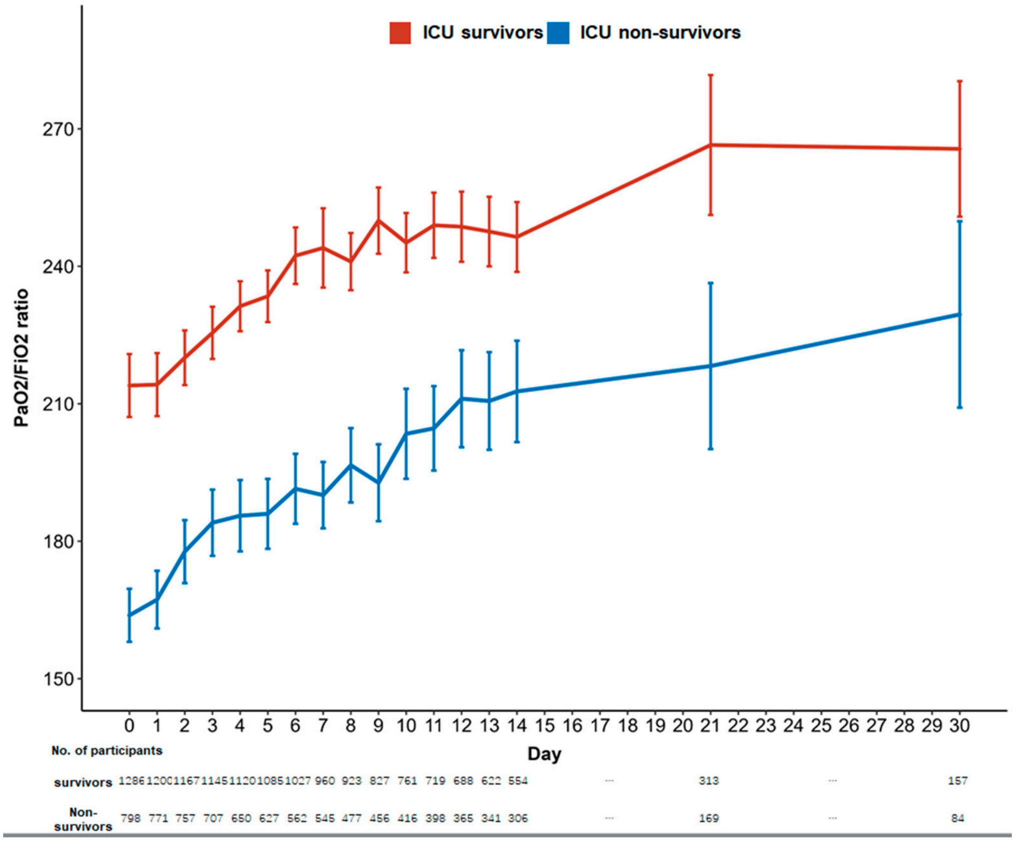

Figure S2K

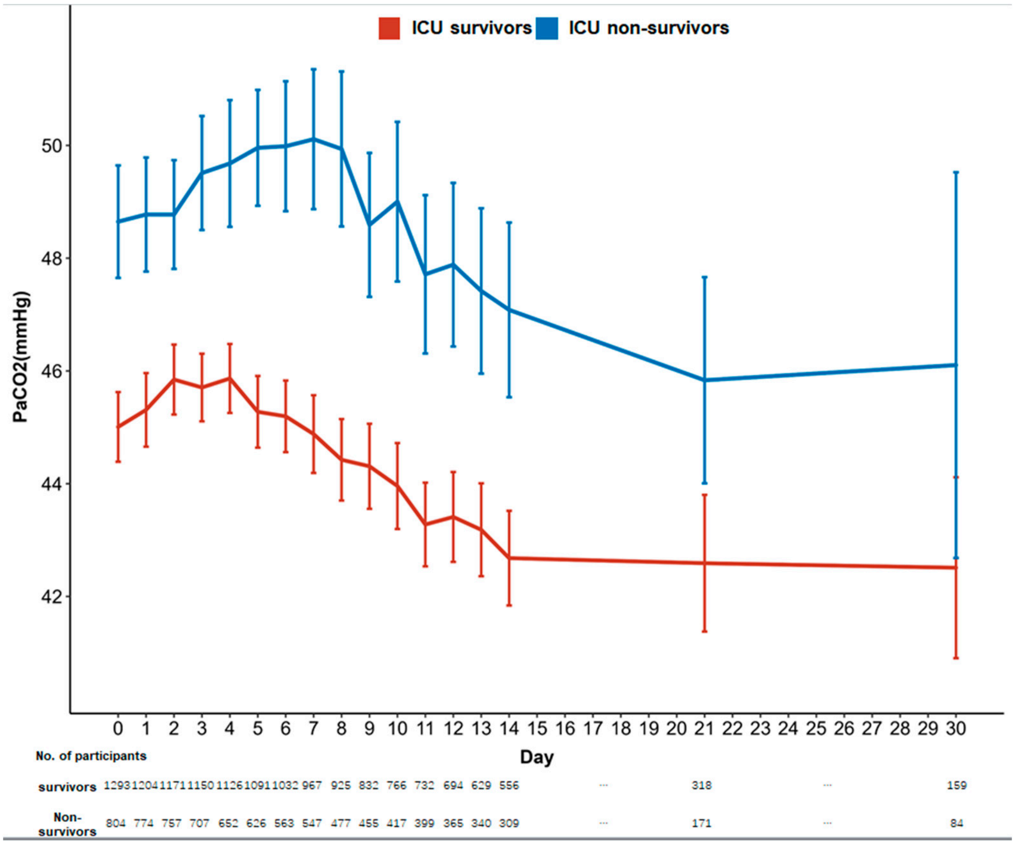

Figure S2L

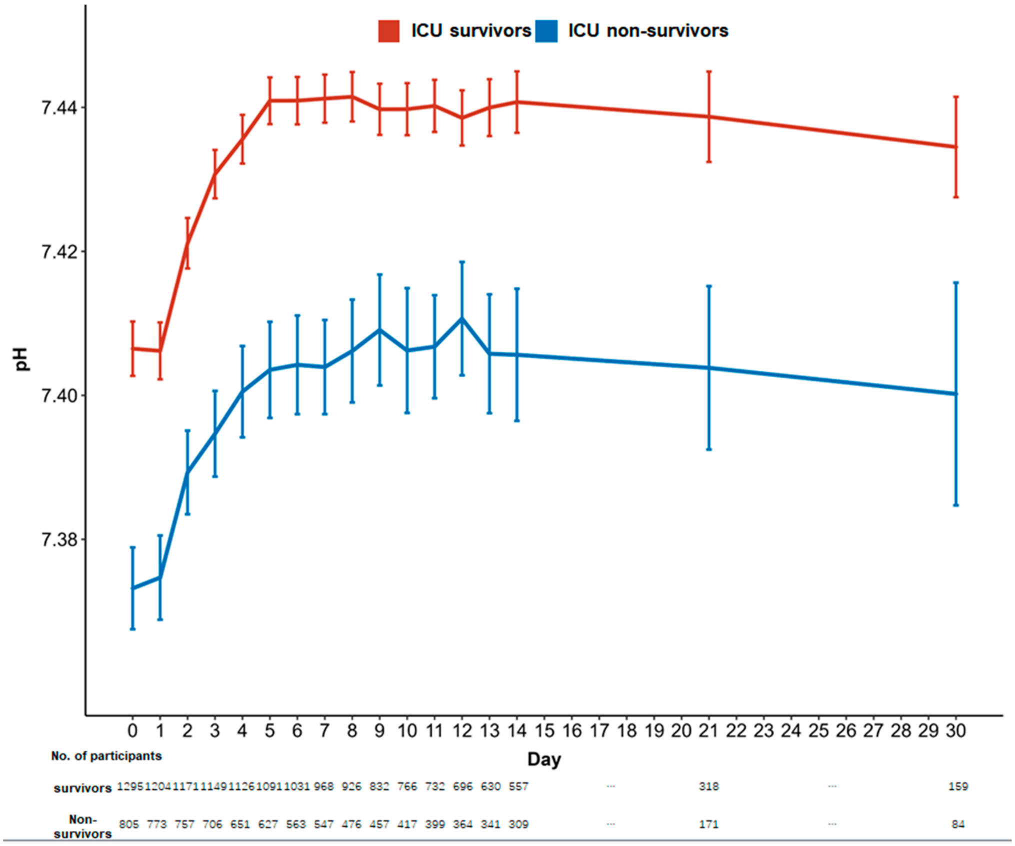

Figure S2M

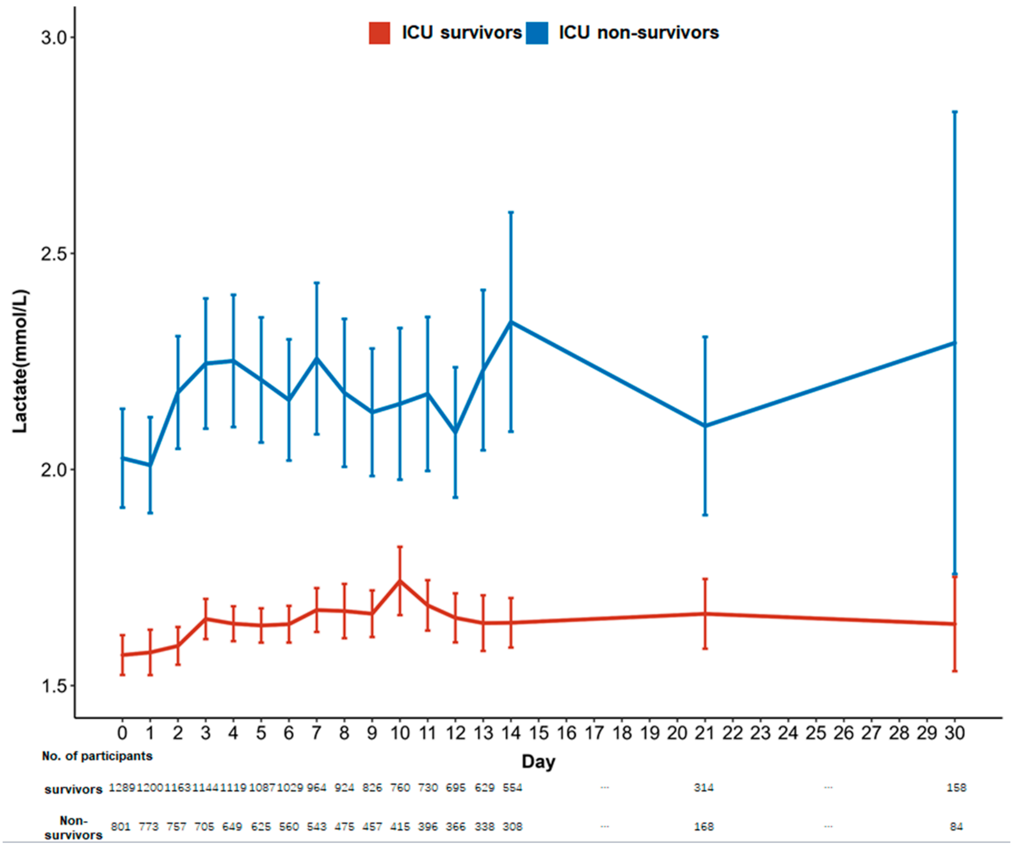

ICU survivors
ICU non-survivors
Lactate(mmol/L)
3.0
2.5
2.0
1.5
0 1 2 3 4 5 6 7 8 9 10 11 12 13 14 15 16 17 18 19 20 21 22 23 24 25 26 27 28 29 30
Day
No. of participants
survivors 1289 1200 1163 1144 1119 1087 1029 964 924 826 760 730 695 629 554 ··· 314 ··· 158
Non-survivors 801 773 757 705 649 625 560 543 475 457 415 396 366 338 308 ··· 168 ··· 84

Figure S2N

Supplemental Tables S1. Summary of ventilatory parameters and gas exchange variables over the first 10 days in ICU survivors versus ICU non-survivors

Variables	Total (n=2480)	Group		Test statistic : U	P-value
		ICU survivors (n=1532)	ICU non-survivors (n=948)		
RR, mean (SD)	20 (16-24)	20 (16-23)	20 (17-25)	58022052.50	<0.001
PEEP, mean (SD)	8 (5-10)	7 (5-8)	8 (5-10)	39998212.50	<0.001
FiO2, mean (SD)	40 (40-60)	40 (35-50)	50 (40-70)	41980333.50	<0.001
VT/kg, mean (SD)	7 (6-8)	7 (6-8)	7 (6-7)	9843221.50	<0.001
△P, mean (SD)	12 (10-15)	12 (10-15)	14 (12-15)	8615150.00	<0.001
(4×ΔP+RR) , mean (SD)	70 (61-78)	68 (60-77)	72 (63-81)	8651775.50	<0.001
VE, mean (SD)	9 (7-10)	8 (7-10)	9 (8-11)	14289638.50	<0.001
VR, mean (SD)	1.94 (1.43-2.37)	1.81 (1.37-2.26)	2.15 (1.74-2.61)	5592287.00	<0.001
PaO_2, mean (SD)	84 (70-105)	86 (72-107)	81 (68-102)	43423737.00	<0.001
PaO_2/FiO_2, mean (SD)	200 (143-266)	216 (165-281)	167 (114-233)	26871713.50	<0.001
$PaCO_2$, mean (SD)	44 (38-53)	43 (38-51)	47 (39-57)	32494350.00	<0.001
pH, mean (SD)	7.41 (7.37-7.44)	7.43 (7.40-7.45)	7.39 (7.35-7.41)	28639529.00	<0.001
Lactate, mean (SD)	1.87 (1.31-2.09)	1.47 (1.24-1.81)	2.20 (1.49-2.45)	29298613.50	<0.001

Footnotes: Data are presented as median (IQR). The "Total" column is descriptive only and was not included in group comparisons. Group comparisons were performed using the Mann-Whitney U test; all tests were two-sided. U denotes the Mann-Whitney U statistic. Abbreviations: RR, respiratory rate; PEEP, positive end-expiratory pressure; FiO_2, fraction of inspired oxygen; VT/kg, tidal volume per predicted body weight; △P, driving pressure; VE, minute ventilation; VR, ventilatory ratio; PaO_2, partial pressure of arterial oxygen; $PaCO_2$, partial pressure of arterial carbon dioxide; pH, arterial pH.

Supplementary Table S2. Percentage of missing data

Variables	Percentage of missing data (%)	ICU survivors percentage of missing data (%)	ICU non-survivors percentage of missing data (%)
Variables selected for imputations			
Mean arterial pressure (MAP)	12.14	9.03	17.17
Heart rate (HR)	12.14	9.04	17.16
Respiratory rate (RR)	10.34	9.14	12.24
PEEP	27.16	29.34	23.64
Tidal volume per body weight (VT/kg)	17.09	17.90	15.89
Minute ventilation (VE)	17.92	18.52	16.96
Ventilatory ratio (VR)	34.71	34.42	35.16
[(4×ΔP)+RR]	18.07	17.15	19.53
Fluid balance (input − output)	12.34	9.22	17.38
PaO_2/FiO_2 ratio	33.04	31.75	35.12
$PaCO_2$	32.79	31.42	34.99
pH	32.78	31.41	34.99
Lactate	33.05	31.73	35.17
C-reactive protein (CRP)	21.99	21.21	23.25
Interleukin-6 (IL-6)	22.10	21.22	23.25
Procalcitonin (PCT)	28.71	27.45	30.75
B-type natriuretic peptide (BNP)	37.59	37.37	37.95
Absolute neutrophil count (ANC)	20.71	18.75	23.87
Absolute lymphocyte count (ALC)	20.63	18.63	23.86
Hemoglobin (Hb)	20.58	18.70	23.62

Variables	Percentage of missing data (%)	ICU survivors percentage of missing data (%)	ICU non-survivors percentage of missing data (%)
D-dimer	30.31	30.10	30.65
Creatinine (Cr)	19.00	17.52	21.39
Platelet count (PLT)	20.65	18.81	23.63
Variables not requiring imputation			
Body temperature	12.18	9.09	17.17
FiO_2	12.54	9.49	17.47
Tidal volume (VT)	16.10	16.91	14.90
Driving pressure (ΔP)	15.65	14.06	18.23

Supplementary Table S3. Predictor Variables for Multiple Imputation

Variables selected for imputations	Predictor variables
Mean arterial pressure (MAP)	Septic shock, Cardiac disease, SOFA score-cardiovascular, BNP, Cardiac troponin T (cTnT), MAP (lag-1 and lag+1 values)
Heart rate (HR)	Septic shock, Cardiac disease, SOFA score-cardiovascular, BNP, Cardiac troponin T (cTnT), HR (lag-1 and lag+1 values)
Respiratory rate (RR)	MAP, HR, Body temperature, RR (lag-1 and lag+1 values)
PEEP	FiO_2, PEEP (lag-1 and lag+1 values)
Tidal volume per body weight (VT/kg)	VT, Age, Sex, VT/kg (lag-1 and lag+1 values)
Minute ventilation (VE)	VT, RR, VE (lag-1 and lag+1 values)
Ventilatory ratio (VR)	Minute ventilation (VE), $PaCO_2$, Age, Sex, VR (lag-1 and lag+1 values)
[(4×ΔP)+RR]	Driving pressure (ΔP), RR, [(4×ΔP)+RR] (lag-1 and lag+1 values)
Fluid balance (input − output)	MAP, HR, Fluid balance (lag-1 and lag+1 values)
PaO_2/FiO_2 ratio	SpO_2/FiO_2 ratio, PaO_2/FiO_2 ratio (lag-1 and lag+1 values)
$PaCO_2$	RR, VE, $PaCO_2$ (lag-1 and lag+1 values)
pH	$PaCO_2$, HCO_3^-, Lactate, pH (lag-1 and lag+1 values)
Lactate	MAP, HR, Septic shock, Lactate (lag-1 and lag+1 values)
C-reactive protein (CRP)	ANC, White blood cell count (WBC), CRP (lag-1 and lag+1 values)
Interleukin-6 (IL-6)	ANC, White blood cell count (WBC), IL-6 (lag-1 and lag+1 values)
Procalcitonin (PCT)	ANC, White blood cell count (WBC), PCT (lag-1 and lag+1 values)
B-type natriuretic peptide (BNP)	Myoglobin (MYO), Creatine kinase-MB (CK-MB), Troponin T (cTnT), Cardiac disease, BNP (lag-1 and lag+1 values)
Absolute neutrophil count (ANC)	ANC (lag-1 and lag+1 values), WBC
Absolute lymphocyte count (ALC)	ALC (lag-1 and lag+1 values), WBC
Hemoglobin (Hb)	Hb (lag-1 and lag+1 values) , Red blood cell count (RBC)
D-dimer	D-dimer (lag-1 and lag+1 values), PLT

Variables selected for imputations	Predictor variables
Creatinine (Cr)	Cr (lag-1 and lag+1 values), Urea (BUN)
Platelet count (PLT)	PLT (lag-1 and lag+1 values), D-dimer

Supplementary Table S4. Test of proportional hazards assumption for each ventilatory exposure.

Footnote: P values are from the exposure-by-time interaction term in each multivariable Cox model; P > 0.05 indicates no evidence of time-varying effect and thus no violation of the proportional hazards assumption.

Model	Exposure-by-time interaction term	P value
Model 1	VT/kg * day	0.976
Model 2	VE * day	0.402
Model 3	VR * day	0.792
Model 4	△P * day	0.634
Model 5	4×ΔP + RR * day	0.478

Supplemental Tables S5–S14. Multivariable time-dependent Cox regression models for ICU mortality

Note to Tables S5–S14:

Data are presented as β coefficient (regression coefficient), standard error (Se), p-value, and hazard ratio (HR) with 95% confidence interval (CI). HRs were calculated per 1-unit increase for continuous variables unless otherwise specified. All models were adjusted for age, non-pulmonary SOFA score, septic shock, chronic lung disease, heart disease, neurologic disease, liver disease, chronic kidney disease, immunocompromised status, bacterial/viral/fungal infection, corticosteroid therapy, neuromuscular blocker therapy, mean arterial pressure, heart rate, set respiratory rate, PEEP, fluid balance, PaO_2/FiO_2 ratio, $PaCO_2$, pH, lactate, CRP, IL-6, PCT, BNP, albumin, absolute neutrophil count, absolute lymphocyte count, hemoglobin, D-dimer, creatinine, platelet count, and time (day). Time-dependent covariates were handled using the time-varying Cox regression model with repeated measures. Analyses were stratified by ventilation mode (volume-controlled vs. pressure-controlled).

Multiple imputation by chained equations (MICE) was applied for missing data in Tables S5–S9; Tables S10–S14 present the complete-case sensitivity analysis without imputation.

Abbreviations: ICU, intensive care unit; PBW, predicted body weight; VT, tidal volume; VE, minute ventilation; VR, ventilatory ratio; ΔP, driving pressure; RR, respiratory rate; PEEP, positive end-expiratory pressure; SOFA, sequential organ failure assessment; MAP, mean arterial pressure; CRP, C-reactive protein; IL-6, interleukin-6; PCT, procalcitonin; BNP, B-type natriuretic peptide.

Table S5. Multivariable time-dependent Cox regression model for ICU mortality — volume-controlled ventilation group, Model 1 (tidal volume per kg PBW)

Variables	*β*	*Se*	*P*	*HR* (95% *CI*)
Age	$1.97*10^{-2}$	$2.37*10^{-3}$	<0.001	1.02 (1.02 ~ 1.02)
Non-pulmonary SOFA score	$8.40*10^{-2}$	$1.18*10^{-2}$	<0.001	1.09 (1.06 ~ 1.11)
Septic shock	$-3.92*10^{-2}$	$7.92*10^{-2}$	0.620	0.96 (0.82 ~ 1.12)
Chronic lung disease	$6.23*10^{-2}$	$7.22*10^{-2}$	0.388	1.06 (0.92 ~ 1.23)
Cardiac disease	$5.23*10^{-2}$	$8.42*10^{-2}$	0.534	1.05 (0.89 ~ 1.24)
Neurologic disease	$-4.41*10^{-2}$	$8.20*10^{-2}$	0.591	0.96 (0.81 ~ 1.12)
Liver disease	$2.22*10^{-1}$	$1.60*10^{-1}$	0.165	1.25 (0.91 ~ 1.71)
Chronic kidney disease	$-2.05*10^{-1}$	$1.05*10^{-1}$	0.052	0.81 (0.66 ~ 1)
Immunocompromised status	$1.81*10^{-1}$	$6.79*10^{-2}$	0.008	1.2 (1.05 ~ 1.37)
Bacterial infection	$-1.50*10^{-1}$	$1.01*10^{-1}$	0.138	0.86 (0.71 ~ 1.05)
Viral infection	$1.53*10^{-2}$	$7.58*10^{-2}$	0.84	1.02 (0.88 ~ 1.18)
Fungal infection	$1.86*10^{-1}$	$6.86*10^{-2}$	0.007	1.2 (1.05 ~ 1.38)

Variables	*β*	*Se*	*P*	*HR* (95% *CI*)
Systemic glucocorticoid	$3.39*10^{-1}$	$7.86*10^{-2}$	<0.001	1.4 (1.2 ~ 1.64)
Neuromuscular blocker	$4.27*10^{-1}$	$7.65*10^{-2}$	<0.001	1.53 (1.32 ~ 1.78)
Mean arterial pressure (MAP)	$4.36*10^{-9}$	$5.29*10^{-10}$	<0.001	1 (1 ~ 1)
Heart rate (HR)	$7.44*10^{-3}$	$9.25*10^{-4}$	<0.001	1.01 (1.01 ~ 1.01)
Respiratory rate (RR)	$2.38*10^{-3}$	$1.77*10^{-3}$	0.183	1 (1 ~ 1.01)
PEEP	$3.61*10^{-2}$	$6.52*10^{-3}$	<0.001	1.04 (1.02 ~ 1.05)
Tidal volume per body weight (VT/kg)	$-2.55*10^{-3}$	$1.34*10^{-3}$	0.082	1 (0.99 ~ 1)
Fluid balance (input − output)	$1.36*10^{-11}$	$9.91*10^{-12}$	0.240	1 (1 ~ 1)
PaO_2/FiO_2 ratio	$-1.19*10^{-3}$	$1.98*10^{-4}$	<0.001	1 (1 ~ 1)
PaCO2	$4.55*10^{-3}$	$2.14*10^{-3}$	0.038	1 (1 ~ 1.01)
pH	$-6.64*10^{-1}$	$2.83*10^{-1}$	0.027	0.51 (0.3 ~ 0.9)
Lactate	$1.15*10^{-1}$	$1.49*10^{-2}$	<0.001	1.12 (1.09 ~ 1.16)
C-reactive protein (CRP)	$1.01*10^{-3}$	$2.58*10^{-4}$	0.001	1 (1 ~ 1)
Interleukin-6 (IL-6)	$1.31*10^{-4}$	$1.98*10^{-5}$	<0.001	1 (1 ~ 1)
Procalcitonin (PCT)	$-1.84*10^{-3}$	$1.55*10^{-3}$	0.235	1 (1 ~ 1)
B-type natriuretic peptide (BNP)	$9.57*10^{-6}$	$2.81*10^{-6}$	0.001	1 (1 ~ 1)
Albumin	$-9.53*10^{-3}$	$3.77*10^{-3}$	0.012	0.99 (0.98 ~ 1)
Absolute neutrophil count (ANC)	$1.89*10^{-2}$	$3.51*10^{-3}$	<0.001	1.02 (1.01 ~ 1.03)
Absolute lymphocyte count (ALC)	$-7.21*10^{-2}$	$3.53*10^{-2}$	0.041	0.93 (0.87 ~ 1)
Hemoglobin (Hb)	$-9.16*10^{-4}$	$1.12*10^{-3}$	0.414	1 (1 ~ 1)
D-dimer	$2.27*10^{-5}$	$1.27*10^{-4}$	0.86	1 (1 ~ 1)
Creatinine (Cr)	$7.18*10^{-4}$	$2.47*10^{-4}$	0.004	1 (1 ~ 1)

**Variables**	***β***	***Se***	***P***	***HR* (95% *CI*)**
Platelet count (PLT)	$-1.34*10^{-3}$	$2.65*10^{-4}$	<0.001	1 (1 ~ 1)
Time (day)	$2.07*10^{-2}$	$2.70*10^{-3}$	<0.001	1.02 (1.02 ~ 1.03)

For covariate definitions and abbreviations, see the note to Tables S2–S11.

Table S6. Multivariable time-dependent Cox regression model for ICU mortality — volume-controlled ventilation group, Model 2 (minute ventilation)

Variables	*β*	*Se*	*P*	*HR* (95% *CI*)
Age	$1.98*10^{-2}$	$2.37*10^{-3}$	<0.001	1.02 (1.02 ~ 1.02)
Non-pulmonary SOFA score	$8.39*10^{-2}$	$1.19*10^{-2}$	<0.001	1.09 (1.06 ~ 1.11)
Septic shock	$-3.94*10^{-2}$	$7.93*10^{-2}$	0.619	0.96 (0.82 ~ 1.12)
Chronic lung disease	$6.31*10^{-2}$	$7.22*10^{-2}$	0.382	1.07 (0.92 ~ 1.23)
Cardiac disease	$5.18*10^{-2}$	$8.42*10^{-2}$	0.538	1.05 (0.89 ~ 1.24)
Neurologic disease	$-4.42*10^{-2}$	$8.21*10^{-2}$	0.590	0.96 (0.81 ~ 1.12)
Liver disease	$2.23*10^{-1}$	$1.60*10^{-1}$	0.163	1.25 (0.91 ~ 1.71)
Chronic kidney disease	$-2.04*10^{-1}$	$1.06*10^{-1}$	0.053	0.82 (0.66 ~ 1)
Immunocompromised status	$1.82*10^{-1}$	$6.79*10^{-2}$	0.007	1.2 (1.05 ~ 1.37)
Bacterial infection	$-1.48*10^{-1}$	$1.01*10^{-1}$	0.145	0.86 (0.71 ~ 1.05)
Viral infection	$1.61*10^{-2}$	$7.58*10^{-2}$	0.832	1.02 (0.88 ~ 1.18)
Fungal infection	$1.86*10^{-1}$	$6.86*10^{-2}$	0.007	1.2 (1.05 ~ 1.38)
Systemic glucocorticoid	$3.40*10^{-1}$	$7.86*10^{-2}$	<0.001	1.4 (1.2 ~ 1.64)
Neuromuscular blocker	$4.32*10^{-1}$	$7.64*10^{-2}$	<0.001	1.54 (1.33 ~ 1.79)
Mean arterial pressure (MAP)	$4.36*10^{-9}$	$5.29*10^{-10}$	<0.001	1 (1 ~ 1)
Heart rate (HR)	$7.51*10^{-3}$	$9.37*10^{-4}$	<0.001	1.01 (1.01 ~ 1.01)
PEEP	$3.62*10^{-2}$	$6.53*10^{-3}$	<0.001	1.04 (1.02 ~ 1.05)
Minute ventilation (VE)	$3.32*10^{-4}$	$6.13*10^{-3}$	0.957	1 (0.99 ~ 1.01)
Fluid balance (input − output)	$1.36*10^{-11}$	$9.91*10^{-12}$	0.240	1 (1 ~ 1)
PaO_2/FiO_2 ratio	$-1.21*10^{-3}$	$1.98*10^{-4}$	<0.001	1 (1 ~ 1)
PaCO2	$4.60*10^{-3}$	$2.14*10^{-3}$	0.036	1 (1 ~ 1.01)

Variables	*β*	*Se*	*P*	*HR* (95% *CI*)
pH	$-6.69*10^{-1}$	$2.82*10^{-1}$	0.026	0.51 (0.29 ~ 0.89)
Lactate	$1.15*10^{-1}$	$1.49*10^{-2}$	<0.001	1.12 (1.09 ~ 1.16)
C-reactive protein (CRP)	$1.01*10^{-3}$	$2.58*10^{-4}$	0.001	1 (1 ~ 1)
Interleukin-6 (IL-6)	$1.32*10^{-4}$	$1.98*10^{-5}$	<0.001	1 (1 ~ 1)
Procalcitonin (PCT)	$-1.84*10^{-3}$	$1.55*10^{-3}$	0.236	1 (1 ~ 1)
B-type natriuretic peptide (BNP)	$9.56*10^{-6}$	$2.81*10^{-6}$	0.001	1 (1 ~ 1)
Albumin	$-9.52*10^{-3}$	$3.77*10^{-3}$	0.012	0.99 (0.98 ~ 1)
Absolute neutrophil count (ANC)	$1.89*10^{-2}$	$3.51*10^{-3}$	<0.001	1.02 (1.01 ~ 1.03)
Absolute lymphocyte count (ALC)	$-7.27*10^{-2}$	$3.53*10^{-2}$	0.040	0.93 (0.87 ~ 1)
Hemoglobin (Hb)	$-9.35*10^{-4}$	$1.12*10^{-3}$	0.404	1 (1 ~ 1)
D-dimer	$2.34*10^{-5}$	$1.27*10^{-4}$	0.856	1 (1 ~ 1)
Creatinine (Cr)	$7.16*10^{-4}$	$2.46*10^{-4}$	0.004	1 (1 ~ 1)
Platelet count (PLT)	$-1.34*10^{-3}$	$2.64*10^{-4}$	<0.001	1 (1 ~ 1)
Time (day)	$2.11*10^{-2}$	$2.71*10^{-3}$	<0.001	1.02 (1.02 ~ 1.03)

For covariate definitions and abbreviations, see the note to Tables S2–S11.

Table S7. Multivariable time-dependent Cox regression model for ICU mortality — volume-controlled ventilation group, Model 3 (ventilatory ratio)

Variables	*β*	*Se*	*P*	*HR* (95% *CI*)
Age	$1.99*10^{-2}$	$2.37*10^{-3}$	<0.001	1.02 (1.02 ~ 1.02)
Non-pulmonary SOFA score	$8.29*10^{-2}$	$1.18*10^{-2}$	<0.001	1.09 (1.06 ~ 1.11)
Septic shock	$-3.91*10^{-2}$	$7.93*10^{-2}$	0.621	0.96 (0.82 ~ 1.12)
Chronic lung disease	$7.79*10^{-2}$	$7.17*10^{-2}$	0.278	1.08 (0.94 ~ 1.24)
Cardiac disease	$4.92*10^{-2}$	$8.42*10^{-2}$	0.559	1.05 (0.89 ~ 1.24)
Neurologic disease	$-4.62*10^{-2}$	$8.21*10^{-2}$	0.573	0.95 (0.81 ~ 1.12)
Liver disease	$2.34*10^{-1}$	$1.57*10^{-1}$	0.135	1.26 (0.93 ~ 1.72)
Chronic kidney disease	$-2.11*10^{-1}$	$1.05*10^{-1}$	0.046	0.81 (0.66 ~ 1)
Immunocompromised status	$1.83*10^{-1}$	$6.79*10^{-2}$	0.007	1.2 (1.05 ~ 1.37)
Bacterial infection	$-1.46*10^{-1}$	$1.01*10^{-1}$	0.147	0.86 (0.71 ~ 1.05)
Viral infection	$1.92*10^{-2}$	$7.59*10^{-2}$	0.800	1.02 (0.88 ~ 1.18)
Fungal infection	$1.89*10^{-1}$	$6.85*10^{-2}$	0.006	1.21 (1.06 ~ 1.38)
Systemic glucocorticoid	$3.43*10^{-1}$	$7.85*10^{-2}$	<0.001	1.41 (1.21 ~ 1.64)
Neuromuscular blocker	$4.45*10^{-1}$	$7.57*10^{-2}$	<0.001	1.56 (1.35 ~ 1.81)
Mean arterial pressure (MAP)	$4.35*10^{-9}$	$5.29*10^{-10}$	<0.001	1 (1 ~ 1)
Heart rate (HR)	$7.61*10^{-3}$	$9.20*10^{-4}$	<0.001	1.01 (1.01 ~ 1.01)
PEEP	$3.61*10^{-2}$	$6.55*10^{-3}$	<0.001	1.04 (1.02 ~ 1.05)
Ventilatory ratio (VR)	$-3.15*10^{-3}$	$1.59*10^{-2}$	0.843	1 (0.97 ~ 1.03)
Fluid balance (input − output)	$1.35*10^{-11}$	$9.90*10^{-12}$	0.240	1 (1 ~ 1)
PaO_2/FiO_2 ratio	$-1.32*10^{-3}$	$2.02*10^{-4}$	<0.001	1 (1 ~ 1)
pH	-1.04*10+00	$1.94*10^{-1}$	<0.001	0.35 (0.24 ~ 0.52)

Variables	*β*	*Se*	*P*	*HR* (95% *CI*)
Lactate	$1.12*10^{-1}$	$1.35*10^{-2}$	<0.001	1.12 (1.09 ~ 1.15)
C-reactive protein (CRP)	$1.02*10^{-3}$	$2.60*10^{-4}$	0.001	1 (1 ~ 1)
Interleukin-6 (IL-6)	$1.31*10^{-4}$	$1.97*10^{-5}$	<0.001	1 (1 ~ 1)
Procalcitonin (PCT)	$-1.99*10^{-3}$	$1.55*10^{-3}$	0.202	1 (0.99 ~ 1)
B-type natriuretic peptide (BNP)	$9.76*10^{-6}$	$2.82*10^{-6}$	0.001	1 (1 ~ 1)
Albumin	$-9.07*10^{-3}$	$3.77*10^{-3}$	0.016	0.99 (0.98 ~ 1)
Absolute neutrophil count (ANC)	$1.94*10^{-2}$	$3.51*10^{-3}$	<0.001	1.02 (1.01 ~ 1.03)
Absolute lymphocyte count (ALC)	$-7.53*10^{-2}$	$3.53*10^{-2}$	0.033	0.93 (0.87 ~ 0.99)
Hemoglobin (Hb)	$-8.46*10^{-4}$	$1.12*10^{-3}$	0.450	1 (1 ~ 1)
D-dimer	$2.23*10^{-5}$	$1.28*10^{-4}$	0.862	1 (1 ~ 1)
Creatinine (Cr)	$6.59*10^{-4}$	$2.45*10^{-4}$	0.008	1 (1 ~ 1)
Platelet count (PLT)	$-1.35*10^{-3}$	$2.65*10^{-4}$	<0.001	1 (1 ~ 1)
Time (day)	$2.20*10^{-2}$	$2.71*10^{-3}$	<0.001	1.02 (1.02 ~ 1.03)

For covariate definitions and abbreviations, see the note to Tables S2–S11.

Table S8. Multivariable time-dependent Cox regression model for ICU mortality — pressure-controlled ventilation group, Model 4 (driving pressure)

**Variables**	***β***	***Se***	***P***	***HR* (95% *CI*)**
Age	$1.97*10^{-2}$	$2.37*10^{-3}$	<0.001	1.02 (1.02 ~ 1.02)
Non-pulmonary SOFA score	$8.40*10^{-2}$	$1.18*10^{-2}$	<0.001	1.09 (1.06 ~ 1.11)
Septic shock	$-3.92*10^{-2}$	$7.92*10^{-2}$	0.620	0.96 (0.82 ~ 1.12)
Chronic lung disease	$6.25*10^{-2}$	$7.22*10^{-2}$	0.387	1.06 (0.92 ~ 1.23)
Cardiac disease	$5.16*10^{-2}$	$8.42*10^{-2}$	0.540	1.05 (0.89 ~ 1.24)
Neurologic disease	$-4.46*10^{-2}$	$8.21*10^{-2}$	0.587	0.96 (0.81 ~ 1.12)
Liver disease	$2.22*10^{-1}$	$1.60*10^{-1}$	0.164	1.25 (0.91 ~ 1.71)
Chronic kidney disease	$-2.04*10^{-1}$	$1.05*10^{-1}$	0.053	0.82 (0.66 ~ 1)
Immunocompromised status	$1.81*10^{-1}$	$6.79*10^{-2}$	0.008	1.2 (1.05 ~ 1.37)
Bacterial infection	$-1.50*10^{-1}$	$1.01*10^{-1}$	0.139	0.86 (0.71 ~ 1.05)
Viral infection	$1.57*10^{-2}$	$7.58*10^{-2}$	0.835	1.02 (0.88 ~ 1.18)
Fungal infection	$1.86*10^{-1}$	$6.86*10^{-2}$	0.007	1.2 (1.05 ~ 1.38)
Systemic glucocorticoid	$3.38*10^{-1}$	$7.86*10^{-2}$	<0.001	1.4 (1.2 ~ 1.64)
Neuromuscular blocker	$4.28*10^{-1}$	$7.65*10^{-2}$	<0.001	1.53 (1.32 ~ 1.78)
Mean arterial pressure (MAP)	$4.36*10^{-9}$	$5.29*10^{-10}$	<0.001	1 (1 ~ 1)
Heart rate (HR)	$7.44*10^{-3}$	$9.24*10^{-4}$	<0.001	1.01 (1.01 ~ 1.01)
Respiratory rate (RR)	$2.33*10^{-3}$	$1.78*10^{-3}$	0.192	1 (1 ~ 1.01)
PEEP	$3.61*10^{-2}$	$6.52*10^{-3}$	<0.001	1.04 (1.02 ~ 1.05)
Driving pressure (ΔP)	$1.35*10^{-3}$	$1.55*10^{-3}$	0.387	1 (1 ~ 1)
Fluid balance (input − output)	$1.36*10^{-11}$	$9.91*10^{-12}$	0.240	1 (1 ~ 1)
PaO_2/FiO_2 ratio	$-1.19*10^{-3}$	$1.98*10^{-4}$	<0.001	1 (1 ~ 1)

Variables	β	*Se*	*P*	*HR* (95% *CI*)
PaCO2	$4.53*10^{-3}$	$2.14*10^{-3}$	0.039	1 (1 ~ 1.01)
pH	$-6.61*10^{-1}$	$2.81*10^{-1}$	0.027	0.52 (0.3 ~ 0.9)
Lactate	$1.16*10^{-1}$	$1.49*10^{-2}$	<0.001	1.12 (1.09 ~ 1.16)
C-reactive protein (CRP)	$1.01*10^{-3}$	$2.58*10^{-4}$	0.001	1 (1 ~ 1)
Interleukin-6 (IL-6)	$1.31*10^{-4}$	$1.98*10^{-5}$	<0.001	1 (1 ~ 1)
Procalcitonin (PCT)	$-1.85*10^{-3}$	$1.56*10^{-3}$	0.234	1 (1 ~ 1)
B-type natriuretic peptide (BNP)	$9.53*10^{-6}$	$2.81*10^{-6}$	0.001	1 (1 ~ 1)
Albumin	$-9.50*10^{-3}$	$3.76*10^{-3}$	0.012	0.99 (0.98 ~ 1)
Absolute neutrophil count (ANC)	$1.89*10^{-2}$	$3.51*10^{-3}$	<0.001	1.02 (1.01 ~ 1.03)
Absolute lymphocyte count (ALC)	$-7.21*10^{-2}$	$3.52*10^{-2}$	0.041	0.93 (0.87 ~ 1)
Hemoglobin (Hb)	$-9.23*10^{-4}$	$1.12*10^{-3}$	0.411	1 (1 ~ 1)
D-dimer	$2.33*10^{-5}$	$1.27*10^{-4}$	0.856	1 (1 ~ 1)
Creatinine (Cr)	$7.20*10^{-4}$	$2.47*10^{-4}$	0.004	1 (1 ~ 1)
Platelet count (PLT)	$-1.34*10^{-3}$	$2.64*10^{-4}$	<0.001	1 (1 ~ 1)
Time (day)	$2.07*10^{-2}$	$2.69*10^{-3}$	<0.001	1.02 (1.02 ~ 1.03)

For covariate definitions and abbreviations, see the note to Tables S2–S11.

Table S9. Multivariable time-dependent Cox regression model for ICU mortality — pressure-controlled ventilation group, Model 5 (4×ΔP + RR)

Variables	*β*	*Se*	*P*	*HR* (95% *CI*)
Age	$1.97*10^{-2}$	$2.38*10^{-3}$	<0.001	1.02 (1.02 ~ 1.02)
Non-pulmonary SOFA score	$8.40*10^{-2}$	$1.19*10^{-2}$	<0.001	1.09 (1.06 ~ 1.11)
Septic shock	$-3.89*10^{-2}$	$7.92*10^{-2}$	0.624	0.96 (0.82 ~ 1.12)
Chronic lung disease	$6.17*10^{-2}$	$7.22*10^{-2}$	0.392	1.06 (0.92 ~ 1.23)
Cardiac disease	$4.98*10^{-2}$	$8.42*10^{-2}$	0.555	1.05 (0.89 ~ 1.24)
Neurologic disease	$-4.60*10^{-2}$	$8.22*10^{-2}$	0.576	0.96 (0.81 ~ 1.12)
Liver disease	$2.22*10^{-1}$	$1.60*10^{-1}$	0.166	1.25 (0.91 ~ 1.71)
Chronic kidney disease	$-2.04*10^{-1}$	$1.06*10^{-1}$	0.053	0.82 (0.66 ~ 1)
Immunocompromised status	$1.81*10^{-1}$	$6.79*10^{-2}$	0.008	1.2 (1.05 ~ 1.37)
Bacterial infection	$-1.49*10^{-1}$	$1.01*10^{-1}$	0.141	0.86 (0.71 ~ 1.05)
Viral infection	$1.68*10^{-2}$	$7.58*10^{-2}$	0.825	1.02 (0.88 ~ 1.18)
Fungal infection	$1.85*10^{-1}$	$6.86*10^{-2}$	0.007	1.2 (1.05 ~ 1.38)
Systemic glucocorticoid	$3.37*10^{-1}$	$7.87*10^{-2}$	<0.001	1.4 (1.2 ~ 1.63)
Neuromuscular blocker	$4.30*10^{-1}$	$7.63*10^{-2}$	<0.001	1.54 (1.32 ~ 1.78)
Mean arterial pressure (MAP)	$4.36*10^{-9}$	$5.29*10^{-10}$	<0.001	1 (1 ~ 1)
Heart rate (HR)	$7.48*10^{-3}$	$9.20*10^{-4}$	<0.001	1.01 (1.01 ~ 1.01)
PEEP	$3.60*10^{-2}$	$6.53*10^{-3}$	<0.001	1.04 (1.02 ~ 1.05)
[(4×ΔP)+RR]	$6.40*10^{-4}$	$4.16*10^{-4}$	0.148	1 (1 ~ 1)
Fluid balance (input − output)	$1.36*10^{-11}$	$9.93*10^{-12}$	0.240	1 (1 ~ 1)
PaO_2/FiO_2 ratio	$-1.20*10^{-3}$	$1.98*10^{-4}$	<0.001	1 (1 ~ 1)
PaCO2	$4.49*10^{-3}$	$2.15*10^{-3}$	0.041	1 (1 ~ 1.01)

Variables	*β*	*Se*	*P*	*HR* (95% *CI*)
pH	$-6.65*10^{-1}$	$2.83*10^{-1}$	0.027	0.51 (0.3 ~ 0.9)
Lactate	$1.15*10^{-1}$	$1.49*10^{-2}$	<0.001	1.12 (1.09 ~ 1.16)
C-reactive protein (CRP)	$1.02*10^{-3}$	$2.58*10^{-4}$	0.001	1 (1 ~ 1)
Interleukin-6 (IL-6)	$1.31*10^{-4}$	$1.98*10^{-5}$	<0.001	1 (1 ~ 1)
Procalcitonin (PCT)	$-1.87*10^{-3}$	$1.55*10^{-3}$	0.228	1 (1 ~ 1)
B-type natriuretic peptide (BNP)	$9.53*10^{-6}$	$2.82*10^{-6}$	0.001	1 (1 ~ 1)
Albumin	$-9.43*10^{-3}$	$3.76*10^{-3}$	0.012	0.99 (0.98 ~ 1)
Absolute neutrophil count (ANC)	$1.89*10^{-2}$	$3.51*10^{-3}$	<0.001	1.02 (1.01 ~ 1.03)
Absolute lymphocyte count (ALC)	$-7.25*10^{-2}$	$3.53*10^{-2}$	0.040	0.93 (0.87 ~ 1)
Hemoglobin (Hb)	$-9.55*10^{-4}$	$1.12*10^{-3}$	0.394	1 (1 ~ 1)
D-dimer	$2.29*10^{-5}$	$1.27*10^{-4}$	0.859	1 (1 ~ 1)
Creatinine (Cr)	$7.21*10^{-4}$	$2.47*10^{-4}$	0.004	1 (1 ~ 1)
Platelet count (PLT)	$-1.34*10^{-3}$	$2.65*10^{-4}$	<0.001	1 (1 ~ 1)
Time (day)	$2.10*10^{-2}$	$2.69*10^{-3}$	<0.001	1.02 (1.02 ~ 1.03)

For covariate definitions and abbreviations, see the note to Tables S2–S11.

Table S10. Sensitivity analysis: multivariable time-dependent Cox regression model for ICU mortality without multiple imputation — volume-controlled ventilation group, Model 1 (tidal volume per kg PBW)

Variables	*β*	*Se*	*P*	*HR* (95% *CI*)
Age	$2.21*10^{-2}$	$3.66*10^{-3}$	<0.001	1.02 (1.02 ~ 1.03)
Non-pulmonary SOFA score	$4.98*10^{-2}$	$2.05*10^{-2}$	0.015	1.05 (1.01 ~ 1.09)
Septic shock	$2.81*10^{-2}$	$1.21*10^{-1}$	0.816	1.03 (0.81 ~ 1.30)
Chronic lung disease	$-2.08*10^{-1}$	$1.25*10^{-1}$	0.096	0.81 (0.64 ~ 1.04)
Cardiac disease	$1.17*10^{-1}$	$1.35*10^{-1}$	0.383	1.12 (0.86 ~ 1.46)
Neurologic disease	$-2.56*10^{-2}$	$1.28*10^{-1}$	0.841	0.97 (0.76 ~ 1.25)
Liver disease	$2.38*10^{-1}$	$2.65*10^{-1}$	0.369	1.27 (0.75 ~ 2.13)
Chronic kidney disease	$-2.94*10^{-1}$	$1.78*10^{-1}$	0.100	0.75 (0.53 ~ 1.06)
Immunocompromised status	$2.01*10^{-1}$	$1.14*10^{-1}$	0.078	1.22 (0.98 ~ 1.53)
Bacterial infection	$-1.60*10^{-1}$	$1.63*10^{-1}$	0.326	0.85 (0.62 ~ 1.17)
Viral infection	$8.55*10^{-3}$	$1.21*10^{-1}$	0.944	1.01 (0.80 ~ 1.28)
Fungal infection	$6.95*10^{-2}$	$1.11*10^{-1}$	0.529	1.07 (0.86 ~ 1.33)
Systemic glucocorticoid	$3.50*10^{-1}$	$1.31*10^{-1}$	0.008	1.42 (1.10 ~ 1.83)
Neuromuscular blocker	$3.80*10^{-1}$	$1.28*10^{-1}$	0.003	1.46 (1.14 ~ 1.88)
Mean arterial pressure (MAP)	$1.82*10^{-4}$	$1.31*10^{-4}$	0.163	1.00 (1.00 ~ 1.00)
Heart rate (HR)	$5.97*10^{-3}$	$1.69*10^{-3}$	<0.001	1.01 (1.00 ~ 1.01)
Respiratory rate (RR)	$5.62*10^{-3}$	$7.43*10^{-3}$	0.449	1.01 (0.99 ~ 1.02)
PEEP	$4.08*10^{-3}$	$1.81*10^{-2}$	0.821	1.00 (0.97 ~ 1.04)
Tidal volume per body weight (VT/kg)	$-2.71*10^{-3}$	$1.08*10^{-3}$	0.012	1.00 (1.00 ~ 1.00)
Fluid balance (input − output)	$8.60*10^{-5}$	$2.68*10^{-5}$	0.001	1.00 (1.00 ~ 1.00)
PaO_2/FiO_2 ratio	$-2.04*10^{-3}$	$5.31*10^{-4}$	<0.001	1.00 (1.00 ~ 1.00)

**Variables**	***β***	***Se***	***P***	***HR* (95% *CI*)**
PaCO2	$1.84*10^{-3}$	$4.57*10^{-3}$	0.687	1.00 (0.99 ~ 1.01)
pH	$-4.40*10^{-1}$	$7.21*10^{-1}$	0.542	0.64 (0.16 ~ 2.65)
Lactate	$1.35*10^{-1}$	$1.99*10^{-2}$	<0.001	1.14 (1.10 ~ 1.19)
C-reactive protein (CRP)	$1.01*10^{-4}$	$4.85*10^{-4}$	0.836	1.00 (1.00 ~ 1.00)
Interleukin-6 (IL-6)	$9.00*10^{-5}$	$4.58*10^{-5}$	0.049	1.00 (1.00 ~ 1.00)
Procalcitonin (PCT)	$-1.39*10^{-3}$	$3.37*10^{-3}$	0.681	1.00 (0.99 ~ 1.01)
B-type natriuretic peptide (BNP)	$1.15*10^{-5}$	$6.09*10^{-6}$	0.059	1.00 (1.00 ~ 1.00)
Albumin	$-6.36*10^{-3}$	$8.07*10^{-3}$	0.430	0.99 (0.98 ~ 1.01)
Absolute neutrophil count (ANC)	$2.88*10^{-2}$	$7.06*10^{-3}$	<0.001	1.03 (1.02 ~ 1.04)
Absolute lymphocyte count (ALC)	$-1.10*10^{-1}$	$6.11*10^{-2}$	0.073	0.90 (0.80 ~ 1.01)
Hemoglobin (Hb)	$-1.79*10^{-3}$	$2.40*10^{-3}$	0.456	1.00 (0.99 ~ 1.00)
D-dimer	$1.14*10^{-5}$	$1.25*10^{-4}$	0.928	1.00 (1.00 ~ 1.00)
Creatinine (Cr)	$-1.83*10^{-4}$	$5.41*10^{-4}$	0.735	1.00 (1.00 ~ 1.00)
Platelet count (PLT)	$-2.30*10^{-3}$	$6.40*10^{-4}$	<0.001	1.00 (1.00 ~ 1.00)
Time (day)	$2.70*10^{-2}$	$1.07*10^{-2}$	0.011	1.03 (1.01 ~ 1.05)

For covariate definitions and abbreviations, see the note to Tables S2–S11.

Table S11. Sensitivity analysis: multivariable time-dependent Cox regression model for ICU mortality without multiple imputation — volume-controlled ventilation group, Model 2 (minute ventilation)

Variables	*β*	*Se*	*P*	*HR* (95% *CI*)
Age	$2.20*10^{-2}$	$3.47*10^{-3}$	<0.001	1.02 (1.02 ~ 1.03)
Non-pulmonary SOFA score	$4.97*10^{-2}$	$1.91*10^{-2}$	0.009	1.05 (1.01 ~ 1.09)
Septic shock	$6.03*10^{-3}$	$1.16*10^{-1}$	0.959	1.01 (0.80 ~ 1.26)
Chronic lung disease	$-1.47*10^{-1}$	$1.16*10^{-1}$	0.203	0.86 (0.69 ~ 1.08)
Cardiac disease	$1.18*10^{-1}$	$1.24*10^{-1}$	0.345	1.12 (0.88 ~ 1.44)
Neurologic disease	$-2.22*10^{-2}$	$1.19*10^{-1}$	0.852	0.98 (0.77 ~ 1.23)
Liver disease	$2.19*10^{-1}$	$2.80*10^{-1}$	0.433	1.25 (0.72 ~ 2.16)
Chronic kidney disease	$-2.46*10^{-1}$	$1.66*10^{-1}$	0.139	0.78 (0.56 ~ 1.08)
Immunocompromised status	$2.08*10^{-1}$	$1.04*10^{-1}$	0.046	1.23 (1.00 ~ 1.51)
Bacterial infection	$-2.31*10^{-1}$	$1.51*10^{-1}$	0.126	0.79 (0.59 ~ 1.07)
Viral infection	$-1.89*10^{-2}$	$1.13*10^{-1}$	0.868	0.98 (0.79 ~ 1.23)
Fungal infection	$7.50*10^{-2}$	$1.02*10^{-1}$	0.462	1.08 (0.88 ~ 1.32)
Systemic glucocorticoid	$2.92*10^{-1}$	$1.22*10^{-1}$	0.016	1.34 (1.05 ~ 1.70)
Neuromuscular blocker	$3.85*10^{-1}$	$1.20*10^{-1}$	0.001	1.47 (1.16 ~ 1.86)
Mean arterial pressure (MAP)	$2.05*10^{-4}$	$1.03*10^{-4}$	0.047	1.00 (1.00 ~ 1.00)
Heart rate (HR)	$7.28*10^{-3}$	$1.62*10^{-3}$	<0.001	1.01 (1.00 ~ 1.01)
PEEP	$7.80*10^{-3}$	$1.65*10^{-2}$	0.637	1.01 (0.98 ~ 1.04)
Minute ventilation (VE)	$-1.45*10^{-2}$	$1.66*10^{-2}$	0.381	0.99 (0.95 ~ 1.02)
Fluid balance (input − output)	$9.00*10^{-5}$	$2.49*10^{-5}$	<0.001	1.00 (1.00 ~ 1.00)
PaO_2/FiO_2 ratio	$-1.92*10^{-3}$	$4.89*10^{-4}$	<0.001	1.00 (1.00 ~ 1.00)
PaCO2	$4.73*10^{-3}$	$4.03*10^{-3}$	0.241	1.00 (1.00 ~ 1.01)

Variables	β	*Se*	*P*	*HR* (95% *CI*)
pH	$-2.08*10^{-1}$	$6.68*10^{-1}$	0.756	0.81 (0.22 ~ 3.01)
Lactate	$1.38*10^{-1}$	$1.81*10^{-2}$	<0.001	1.15 (1.11 ~ 1.19)
C-reactive protein (CRP)	$2.96*10^{-4}$	$4.54*10^{-4}$	0.514	1.00 (1.00 ~ 1.00)
Interleukin-6 (IL-6)	$9.17*10^{-5}$	$4.46*10^{-5}$	0.040	1.00 (1.00 ~ 1.00)
Procalcitonin (PCT)	$-1.84*10^{-3}$	$3.27*10^{-3}$	0.573	1.00 (0.99 ~ 1.00)
B-type natriuretic peptide (BNP)	$1.03*10^{-5}$	$5.53*10^{-6}$	0.062	1.00 (1.00 ~ 1.00)
Albumin	$-4.91*10^{-3}$	$7.56*10^{-3}$	0.516	1.00 (0.98 ~ 1.01)
Absolute neutrophil count (ANC)	$2.95*10^{-2}$	$6.42*10^{-3}$	<0.001	1.03 (1.02 ~ 1.04)
Absolute lymphocyte count (ALC)	$-1.00*10^{-1}$	$5.82*10^{-2}$	0.084	0.90 (0.81 ~ 1.01)
Hemoglobin (Hb)	$-1.03*10^{-3}$	$2.23*10^{-3}$	0.646	1.00 (0.99 ~ 1.00)
D-dimer	$1.45*10^{-6}$	$1.26*10^{-4}$	0.991	1.00 (1.00 ~ 1.00)
Creatinine (Cr)	$-9.90*10^{-5}$	$4.79*10^{-4}$	0.836	1.00 (1.00 ~ 1.00)
Platelet count (PLT)	$-2.46*10^{-3}$	$5.94*10^{-4}$	<0.001	1.00 (1.00 ~ 1.00)
Time (day)	$2.64*10^{-2}$	$9.91*10^{-3}$	0.008	1.03 (1.01 ~ 1.05)

For covariate definitions and abbreviations, see the note to Tables S2–S11.

Table S12. Sensitivity analysis: multivariable time-dependent Cox regression model for ICU mortality without multiple imputation — volume-controlled ventilation group, Model 3 (ventilatory ratio)

Variables	*β*	*Se*	*P*	*HR* (95% *CI*)
Age	$2.22*10^{-2}$	$3.64*10^{-3}$	<0.001	1.02 (1.02 ~ 1.03)
Non-pulmonary SOFA score	$4.85*10^{-2}$	$2.05*10^{-2}$	0.018	1.05 (1.01 ~ 1.09)
Septic shock	$3.65*10^{-2}$	$1.20*10^{-1}$	0.762	1.04 (0.82 ~ 1.31)
Chronic lung disease	$-2.00*10^{-1}$	$1.22*10^{-1}$	0.100	0.82 (0.64 ~ 1.04)
Cardiac disease	$1.12*10^{-1}$	$1.34*10^{-1}$	0.401	1.12 (0.86 ~ 1.45)
Neurologic disease	$-3.08*10^{-2}$	$1.27*10^{-1}$	0.809	0.97 (0.76 ~ 1.24)
Liver disease	$2.58*10^{-1}$	$2.61*10^{-1}$	0.322	1.29 (0.78 ~ 2.16)
Chronic kidney disease	$-3.02*10^{-1}$	$1.77*10^{-1}$	0.087	0.74 (0.52 ~ 1.04)
Immunocompromised status	$2.02*10^{-1}$	$1.13*10^{-1}$	0.074	1.22 (0.98 ~ 1.53)
Bacterial infection	$-1.54*10^{-1}$	$1.62*10^{-1}$	0.342	0.86 (0.62 ~ 1.18)
Viral infection	$1.17*10^{-2}$	$1.21*10^{-1}$	0.923	1.01 (0.80 ~ 1.28)
Fungal infection	$7.10*10^{-2}$	$1.10*10^{-1}$	0.519	1.07 (0.87 ~ 1.33)
Systemic glucocorticoid	$3.56*10^{-1}$	$1.31*10^{-1}$	0.007	1.43 (1.10 ~ 1.85)
Neuromuscular blocker	$4.02*10^{-1}$	$1.25*10^{-1}$	0.001	1.50 (1.17 ~ 1.91)
Mean arterial pressure (MAP)	$1.81*10^{-4}$	$1.27*10^{-4}$	0.154	1.00 (1.00 ~ 1.00)
Heart rate (HR)	$6.37*10^{-3}$	$1.66*10^{-3}$	<0.001	1.01 (1.00 ~ 1.01)
PEEP	$3.43*10^{-3}$	$1.80*10^{-2}$	0.849	1.00 (0.97 ~ 1.04)
Ventilatory ratio (VR)	$-1.04*10^{-2}$	$1.59*10^{-2}$	0.515	0.99 (0.96 ~ 1.02)
Fluid balance (input − output)	$8.43*10^{-5}$	$2.66*10^{-5}$	0.002	1.00 (1.00 ~ 1.00)
PaO_2/FiO_2 ratio	$-2.19*10^{-3}$	$5.29*10^{-4}$	<0.001	1.00 (1.00 ~ 1.00)
pH	$-6.58*10^{-1}$	$5.75*10^{-1}$	0.253	0.52 (0.17 ~ 1.6)

Variables	*β*	*Se*	*P*	*HR* (95% *CI*)
Lactate	$1.32*10^{-1}$	$1.90*10^{-2}$	<0.001	1.14 (1.10 ~ 1.18)
C-reactive protein (CRP)	$1.16*10^{-4}$	$4.84*10^{-4}$	0.811	1.00 (1.00 ~ 1.00)
Interleukin-6 (IL-6)	$8.94*10^{-5}$	$4.56*10^{-5}$	0.050	1.00 (1.00 ~ 1.00)
Procalcitonin (PCT)	$-1.55*10^{-3}$	$3.37*10^{-3}$	0.646	1.00 (0.99 ~ 1.01)
B-type natriuretic peptide (BNP)	$1.17*10^{-5}$	$6.07*10^{-6}$	0.055	1.00 (1.00 ~ 1.00)
Albumin	$-6.47*10^{-3}$	$8.06*10^{-3}$	0.423	0.99 (0.98 ~ 1.01)
Absolute neutrophil count (ANC)	$2.92*10^{-2}$	$7.06*10^{-3}$	<0.001	1.03 (1.02 ~ 1.04)
Absolute lymphocyte count (ALC)	$-1.13*10^{-1}$	$6.22*10^{-2}$	0.07	0.89 (0.79 ~ 1.01)
Hemoglobin (Hb)	$-1.84*10^{-3}$	$2.38*10^{-3}$	0.439	1.00 (0.99 ~ 1.00)
D-dimer	$1.43*10^{-5}$	$1.25*10^{-4}$	0.909	1.00 (1.00 ~ 1.00)
Creatinine (Cr)	$-2.21*10^{-4}$	$5.31*10^{-4}$	0.678	1.00 (1.00 ~ 1.00)
Platelet count (PLT)	$-2.30*10^{-3}$	$6.41*10^{-4}$	<0.001	1.00 (1.00 ~ 1.00)
Time (day)	$2.96*10^{-2}$	$1.04*10^{-2}$	0.004	1.03 (1.01 ~ 1.05)

For covariate definitions and abbreviations, see the note to Tables S2–S11.

Table S13. Sensitivity analysis: multivariable time-dependent Cox regression model for ICU mortality without multiple imputation — pressure-controlled ventilation group, Model 4 (driving pressure)

Variables	*β*	*Se*	*P*	*HR* (95% *CI*)
Age	$2.45*10^{-2}$	$4.67*10^{-3}$	<0.001	1.02 (1.02 ~ 1.03)
Non-pulmonary SOFA score	$3.96*10^{-2}$	$2.41*10^{-2}$	0.100	1.04 (0.99 ~ 1.09)
Septic shock	$-1.23*10^{-1}$	$1.48*10^{-1}$	0.405	0.88 (0.66 ~ 1.18)
Chronic lung disease	$-1.22*10^{-2}$	$1.37*10^{-1}$	0.929	0.99 (0.76 ~ 1.29)
Cardiac disease	$1.99*10^{-1}$	$1.47*10^{-1}$	0.174	1.22 (0.92 ~ 1.63)
Neurologic disease	$-1.83*10^{-1}$	$1.61*10^{-1}$	0.256	0.83 (0.61 ~ 1.14)
Liver disease	$1.17*10^{-1}$	$2.71*10^{-1}$	0.666	1.12 (0.66 ~ 1.91)
Chronic kidney disease	$-3.27*10^{-1}$	$2.26*10^{-1}$	0.148	0.72 (0.46 ~ 1.12)
Immunocompromised status	$2.61*10^{-1}$	$1.21*10^{-1}$	0.031	1.30 (1.02 ~ 1.64)
Bacterial infection	$1.19*10^{-1}$	$2.00*10^{-1}$	0.552	1.13 (0.76 ~ 1.67)
Viral infection	$-1.08*10^{-1}$	$1.43*10^{-1}$	0.451	0.90 (0.68 ~ 1.19)
Fungal infection	$1.84*10^{-1}$	$1.28*10^{-1}$	0.15	1.20 (0.94 ~ 1.55)
Systemic glucocorticoid	$1.99*10^{-1}$	$1.36*10^{-1}$	0.145	1.22 (0.93 ~ 1.59)
Neuromuscular blocker	$4.47*10^{-1}$	$1.51*10^{-1}$	0.003	1.56 (1.16 ~ 2.10)
Mean arterial pressure (MAP)	$-2.10*10^{-3}$	$2.79*10^{-3}$	0.452	1.00 (0.99 ~ 1.00)
Heart rate (HR)	$4.38*10^{-3}$	$1.35*10^{-3}$	0.001	1.00 (1.00 ~ 1.01)
Respiratory rate (RR)	$-5.55*10^{-3}$	$7.29*10^{-3}$	0.446	0.99 (0.98 ~ 1.01)
PEEP	$3.92*10^{-2}$	$2.02*10^{-2}$	0.052	1.04 (1.00 ~ 1.08)
Driving pressure (ΔP)	$2.35*10^{-2}$	$1.74*10^{-2}$	0.177	1.02 (0.99 ~ 1.06)
Fluid balance (input − output)	$5.78*10^{-5}$	$3.46*10^{-5}$	0.095	1.00 (1.00 ~ 1.00)
PaO_2/FiO_2 ratio	$-2.23*10^{-3}$	$6.40*10^{-4}$	<0.001	1.00 (1.00 ~ 1.00)

Variables	β	*Se*	*P*	*HR* (95% *CI*)
PaCO2	$-4.35*10^{-3}$	$5.61*10^{-3}$	0.439	1.00 (0.98 ~ 1.01)
pH	-2.61	$8.70*10^{-1}$	0.003	0.07 (0.01 ~ 0.40)
Lactate	$1.36*10^{-1}$	$2.91*10^{-2}$	<0.001	1.15 (1.08 ~ 1.21)
C-reactive protein (CRP)	$8.41*10^{-4}$	$6.83*10^{-4}$	0.218	1.00 (1.00 ~ 1.00)
Interleukin-6 (IL-6)	$5.78*10^{-5}$	$5.78*10^{-5}$	0.318	1.00 (1.00 ~ 1.00)
Procalcitonin (PCT)	$-1.19*10^{-2}$	$4.89*10^{-3}$	0.015	0.99 (0.98 ~ 1.00)
B-type natriuretic peptide (BNP)	$2.73*10^{-5}$	$6.82*10^{-6}$	<0.001	1.00 (1.00 ~ 1.00)
Albumin	$-1.01*10^{-2}$	$1.00*10^{-2}$	0.311	0.99 (0.97 ~ 1.01)
Absolute neutrophil count (ANC)	$1.46*10^{-2}$	$9.39*10^{-3}$	0.120	1.01 (1.00 ~ 1.03)
Absolute lymphocyte count (ALC)	$-5.50*10^{-2}$	$6.71*10^{-2}$	0.412	0.95 (0.83 ~ 1.08)
Hemoglobin (Hb)	$-3.32*10^{-3}$	$2.90*10^{-3}$	0.252	1.00 (0.99 ~ 1.00)
D-dimer	$-2.01*10^{-4}$	$1.74*10^{-4}$	0.247	1.00 (1.00 ~ 1.00)
Creatinine (Cr)	$1.82*10^{-4}$	$5.50*10^{-4}$	0.740	1.00 (1.00 ~ 1.00)
Platelet count (PLT)	$-1.81*10^{-3}$	$7.02*10^{-4}$	0.010	1.00 (1.00 ~ 1.00)
Time (day)	$1.12*10^{-3}$	$1.29*10^{-2}$	0.931	1.00 (0.98 ~ 1.03)

For covariate definitions and abbreviations, see the note to Tables S2–S11.

Table S14. Sensitivity analysis: multivariable time-dependent Cox regression model for ICU mortality without multiple imputation — pressure-controlled ventilation group, Model 5 (4×ΔP + RR)

Variables	*β*	*Se*	*P*	*HR* (95% *CI*)
Age	$2.44*10^{-2}$	$4.67*10^{-3}$	<0.001	1.02 (1.02 ~ 1.03)
Non-pulmonary SOFA score	$4.05*10^{-2}$	$2.40*10^{-2}$	0.091	1.04 (0.99 ~ 1.09)
Septic shock	$-1.27*10^{-1}$	$1.48*10^{-1}$	0.390	0.88 (0.66 ~ 1.18)
Chronic lung disease	$-5.34*10^{-3}$	$1.37*10^{-1}$	0.969	0.99 (0.76 ~ 1.30)
Cardiac disease	$2.00*10^{-1}$	$1.47*10^{-1}$	0.172	1.22 (0.92 ~ 1.63)
Neurologic disease	$-1.81*10^{-1}$	$1.61*10^{-1}$	0.259	0.83 (0.61 ~ 1.14)
Liver disease	$1.01*10^{-1}$	$2.73*10^{-1}$	0.712	1.11 (0.65 ~ 1.89)
Chronic kidney disease	$-3.24*10^{-1}$	$2.25*10^{-1}$	0.151	0.72 (0.47 ~ 1.12)
Immunocompromised status	$2.61*10^{-1}$	$1.21*10^{-1}$	0.030	1.30 (1.03 ~ 1.64)
Bacterial infection	$1.17*10^{-1}$	$2.01*10^{-1}$	0.561	1.12 (0.76 ~ 1.67)
Viral infection	$-1.13*10^{-1}$	$1.43*10^{-1}$	0.429	0.89 (0.67 ~ 1.18)
Fungal infection	$1.84*10^{-1}$	$1.28*10^{-1}$	0.151	1.20 (0.93 ~ 1.55)
Systemic glucocorticoid	$1.99*10^{-1}$	$1.36*10^{-1}$	0.144	1.22 (0.93 ~ 1.59)
Neuromuscular blocker	$4.48*10^{-1}$	$1.51*10^{-1}$	0.003	1.56 (1.16 ~ 2.11)
Mean arterial pressure (MAP)	$-2.46*10^{-3}$	$2.78*10^{-3}$	0.376	1.00 (0.99 ~ 1.00)
Heart rate (HR)	$4.28*10^{-3}$	$1.34*10^{-3}$	0.001	1.00 (1.00 ~ 1.01)
PEEP	$3.98*10^{-2}$	$2.00*10^{-2}$	0.047	1.04 (1.00 ~ 1.08)
[(4×ΔP)+RR]	$2.81*10^{-3}$	$3.33*10^{-3}$	0.400	1.00 (1.00 ~ 1.01)
Fluid balance (input − output)	$5.75*10^{-5}$	$3.46*10^{-5}$	0.096	1.00 (1.00 ~ 1.00)
PaO_2/FiO_2 ratio	$-2.21*10^{-3}$	$6.39*10^{-4}$	0.001	1.00 (1.00 ~ 1.00)
PaCO2	$-3.89*10^{-3}$	$5.59*10^{-3}$	0.487	1.00 (0.99 ~ 1.01)

Variables	*β*	*Se*	*P*	*HR* (95% *CI*)
pH	-2.59	$8.65*10^{-1}$	0.003	0.08 (0.01 ~ 0.41)
Lactate	$1.36*10^{-1}$	$2.91*10^{-2}$	<0.001	1.15 (1.08 ~ 1.21)
C-reactive protein (CRP)	$8.17*10^{-4}$	$6.83*10^{-4}$	0.232	1.00 (1.00 ~ 1.00)
Interleukin-6 (IL-6)	$5.59*10^{-5}$	$5.74*10^{-5}$	0.33	1.00 (1.00 ~ 1.00)
Procalcitonin (PCT)	$-1.18*10^{-2}$	$4.86*10^{-3}$	0.015	0.99 (0.98 ~ 1.00)
B-type natriuretic peptide (BNP)	$2.77*10^{-5}$	$6.79*10^{-6}$	<0.001	1.00 (1.00 ~ 1.00)
Albumin	$-1.03*10^{-2}$	$9.89*10^{-3}$	0.300	0.99 (0.97 ~ 1.01)
Absolute neutrophil count (ANC)	$1.48*10^{-2}$	$9.39*10^{-3}$	0.114	1.01 (1.00 ~ 1.03)
Absolute lymphocyte count (ALC)	$-5.51*10^{-2}$	$6.60*10^{-2}$	0.404	0.95 (0.83 ~ 1.08)
Hemoglobin (Hb)	$-3.27*10^{-3}$	$2.90*10^{-3}$	0.260	1.00 (0.99 ~ 1.00)
D-dimer	$-2.18*10^{-4}$	$1.77*10^{-4}$	0.219	1.00 (1.00 ~ 1.00)
Creatinine (Cr)	$1.50*10^{-4}$	$5.48*10^{-4}$	0.785	1.00 (1.00 ~ 1.00)
Platelet count (PLT)	$-1.82*10^{-3}$	$7.00*10^{-4}$	0.009	1.00 (1.00 ~ 1.00)
Time (day)	$7.71*10^{-4}$	$1.28*10^{-2}$	0.952	1.00 (0.98 ~ 1.03)

For covariate definitions and abbreviations, see the note to Tables S2–S11.

Supplementary Table S15. Sensitivity analysis: multivariable linear time-dependent Cox regression model for ICU mortality excluding adjustment covariates with >30% missingness.

Footnote: Data are presented as β coefficient, p-value, and hazard ratio (HR) with 95% confidence interval (CI). HRs were calculated per 1-unit increase for continuous variables unless otherwise specified. All models were adjusted for age, non-pulmonary SOFA score, septic shock, chronic lung disease, heart disease, neurologic disease, liver disease, chronic kidney disease, immunocompromised status, bacterial/viral/fungal infection, mean arterial pressure, heart rate, fluid balance, PaO_2/FiO_2 ratio, $PaCO_2$, pH, lactate, CRP, IL-6, PCT, BNP, albumin, absolute neutrophil count, absolute lymphocyte count, hemoglobin, D-dimer, creatinine, platelet count, and time (day). Time-dependent covariates were handled using the time-varying Cox regression model with repeated measures. Analyses were stratified by ventilation mode (volume-controlled vs. pressure-controlled). VT/kg, tidal volume per kg predicted body weight; VE, minute ventilation; VR, ventilatory ratio; ΔP, driving pressure; RR, respiratory rate; PEEP, positive end-expiratory pressure. —, not included in the model.

Sensitivity analysis: In the analyses after excluding covariates with >30% missing data, the results remained broadly consistent with the primary analysis: the associations for VT/kg, VR, ΔP, systemic glucocorticoid, neuromuscular blocker, and PEEP remained unchanged. Notably, both RR, VE and [(4×ΔP)+RR] became statistically significant in the sensitivity analysis, suggesting that these variables were more susceptible to covariates with higher proportions of missing data; however, their directions of effect were consistent with the primary analysis. Overall, these findings support the robustness of the primary model.

Supplementary Table S15. A. Sensitivity analysis: multivariable linear time-dependent Cox regression model for ICU mortality excluding adjustment covariates with >30% missingness — volume-controlled ventilation group.

Variables	Model 1 (VT/kg)			Model 2 (VE)			Model 3 (VR)		
	β	HR (95% CI)	P-value	β	HR (95% CI)	P-value	β	HR (95% CI)	P-value
Systemic glucocorticoid	0.399	1.49 (1.28–1.73)	<0.001	0.400	1.49 (1.28–1.74)	<0.001	0.398	1.49 (1.28–1.73)	<0.001
Neuromuscular blocker	0.414	1.51 (1.30–1.76)	<0.001	0.421	1.52 (1.31–1.77)	<0.001	0.409	1.51 (1.29–1.76)	<0.001
Respiratory rate (RR)	0.009	1.01 (1.01–1.01)	<0.001		—			—	
PEEP	0.046	1.05 (1.03–1.06)	<0.001	0.046	1.05 (1.03–1.06)	<0.001	0.046	1.05 (1.03–1.06)	<0.001
VT/kg	−0.001	1.00 (1.00–1.00)	0.444		—			—	
VE		—		0.023	1.02 (1.01–1.04)	0.003		—	
VR		—			—		0.089	1.09 (0.95–1.25)	0.210

Supplementary Table S15. B. Sensitivity analysis: multivariable linear time-dependent Cox regression model for ICU mortality excluding adjustment covariates with >30% missingness — pressure-controlled ventilation group.

Variables	Model 4 (ΔP)			Model 5 (4×ΔP + RR)		
	β	HR (95% CI)	P-value	β	HR (95% CI)	P-value
Systemic glucocorticoid	0.398	1.49 (1.28–1.73)	< 0.001	0.403	1.50 (1.29–1.74)	< 0.001
Neuromuscular blocker	0.414	1.51 (1.30–1.76)	< 0.001	0.432	1.54 (1.32–1.79)	< 0.001
Respiratory rate (RR)	0.009	1.01 (1.01–1.01)	< 0.001		—	
PEEP	0.046	1.05 (1.03–1.06)	< 0.001	0.047	1.05 (1.03–1.06)	< 0.001
ΔP	0.003	1.00 (1.00–1.01)	0.105		—	
(4×ΔP + RR)		—		0.001	1.00 (1.00–1.00)	0.027

Supplementary Table S16. Sensitivity analysis: multivariable Cox regression for ICU mortality using baseline (day 0) data only.

Footnote: Data are presented as β coefficient, p-value, and hazard ratio (HR) with 95% confidence interval (CI). HRs were calculated per 1-unit increase for continuous variables unless otherwise specified. All models were adjusted for age, non-pulmonary SOFA score, septic shock, chronic lung disease, heart disease, neurologic disease, liver disease, chronic kidney disease, immunocompromised status, bacterial/viral/fungal infection, mean arterial pressure, heart rate, fluid balance, PaO_2/FiO_2 ratio, $PaCO_2$, pH, lactate, CRP, IL-6, PCT, BNP, albumin, absolute neutrophil count, absolute lymphocyte count, hemoglobin, D-dimer, creatinine, and platelet count. Analyses were stratified by ventilation mode (volume-controlled vs. pressure-controlled). VT/kg, tidal volume per kg predicted body weight; VE, minute ventilation; VR, ventilatory ratio; ΔP, driving pressure; RR, respiratory rate; PEEP, positive end-expiratory pressure. —, not included in the model.

Sensitivity analysis: In the analyses restricted to day-0 baseline measurements (to account for potential treatment adaptation and evolving clinical status), the results remained broadly consistent with the primary longitudinal analysis: the associations for Models 2–5 (VE, VR, ΔP, and [(4×ΔP)+RR]) remained unchanged. Notably, the association for VT/kg (Model 1) changed in statistical significance in the day-0 analysis, with the P value approaching the conventional 0.05 threshold; however, the effect estimate remained in the same direction, suggesting that this variable may be more susceptible to time-varying clinical factors. Overall, these findings support the general robustness of the primary model, while indicating that the VT/kg association should be interpreted with particular caution.

Supplementary Table S16. A. Sensitivity analysis: multivariable Cox regression for ICU mortality using baseline (day 0) data only — volume-controlled ventilation group.

Variables	Model 1 (VT/kg)			Model 2 (VE)			Model 3 (VR)		
	β	HR (95% CI)	P-value	β	HR (95% CI)	P-value	β	HR (95% CI)	P-value
Systemic glucocorticoid	0.323	1.38 (1.18–1.62)	<0.001	0.325	1.38 (1.18–1.63)	<0.001	0.329	1.39 (1.18–1.63)	<0.001
Neuromuscular blocker	0.327	1.39 (1.18–1.62)	<0.001	0.332	1.39 (1.19–1.63)	<0.001	0.346	1.41 (1.21–1.65)	<0.001
Respiratory rate (RR)	0.006	1.01 (1.00–1.02)	0.245		—			—	
PEEP	0.020	1.02 (1.00–1.04)	0.011	0.020	1.02 (1.00–1.04)	0.01	0.020	1.02 (1.01–1.04)	0.008
VT/kg	−0.005	0.99 (0.99–1.00)	0.047		—			—	
VE		—		0.013	1.01 (0.99–1.04)	0.267		—	
VR		—			—		0.015	0.99 (0.97–1.00)	0.101

Supplementary Table S16. B. Sensitivity analysis: multivariable Cox regression for ICU mortality using baseline (day 0) data only — pressure-controlled ventilation group.

Variables	Model 4 (ΔP)			Model 5 (4×ΔP + RR)		
	β	HR (95% CI)	P-value	β	HR (95% CI)	P-value
Systemic glucocorticoid	0.322	1.38 (1.17–1.62)	< 0.001	0.324	1.38 (1.18–1.63)	< 0.001
Neuromuscular blocker	0.328	1.39 (1.19–1.63)	< 0.001	0.330	1.39 (1.19–1.63)	< 0.001
Respiratory rate (RR)	0.006	1.01 (1.00–1.02)	0.254		—	
PEEP	0.020	1.02 (1.00–1.04)	0.012	0.020	1.02 (1.00–1.04)	0.01
ΔP	0.002	1.00 (0.98–1.03)	0.853		—	
(4×ΔP + RR)		—		0.002	1.00 (1.00–1.01)	0.62

Supplementary Table S17. Sensitivity analysis: multivariable linear time-dependent Cox regression model for ICU mortality excluding patients with septic shock at baseline.

Footnote: Data are presented as β coefficient, p-value, and hazard ratio (HR) with 95% confidence interval (CI). HRs were calculated per 1-unit increase for continuous variables unless otherwise specified. All models were adjusted for age, non-pulmonary SOFA score, septic shock, chronic lung disease, heart disease, neurologic disease, liver disease, chronic kidney disease, immunocompromised status, bacterial/viral/fungal infection, mean arterial pressure, heart rate, fluid balance, PaO_2/FiO_2 ratio, $PaCO_2$, pH, lactate, CRP, IL-6, PCT, BNP, albumin, absolute neutrophil count, absolute lymphocyte count, hemoglobin, D-dimer, creatinine, platelet count, and time (day). Time-dependent covariates were handled using the time-varying Cox regression model with repeated measures. Analyses were stratified by ventilation mode (volume-controlled vs. pressure-controlled). VT/kg, tidal volume per kg predicted body weight; VE, minute ventilation; VR, ventilatory ratio; ΔP, driving pressure; RR, respiratory rate; PEEP, positive end-expiratory pressure. —, not included in the model.

Sensitivity analysis: In the analyses after excluding patients with septic shock at baseline, the effect estimates and statistical significance for VT/kg, VR, ΔP, RR, VE, [(4×ΔP)+RR], systemic glucocorticoid, neuromuscular blocker, and PEEP were unchanged compared with the primary analysis, indicating that the main findings were not materially driven by patients presenting with baseline septic shock, supporting the robustness of our primary model.

Supplementary Table S17. A. Sensitivity analysis: multivariable linear time-dependent Cox regression model for ICU mortality excluding patients with septic shock at baseline — volume-controlled ventilation group.

Variables	Model 1 (VT/kg)			Model 2 (VE)			Model 3 (VR)		
	β	HR (95% CI)	P-value	β	HR (95% CI)	P-value	β	HR (95% CI)	P-value
Systemic glucocorticoid	0.344	1.41 (1.15–1.74)	0.001	0.345	1.41 (1.15–1.74)	0.001	0.349	1.42 (1.15–1.74)	0.001
Neuromuscular blocker	0.534	1.71 (1.40–2.08)	<0.001	0.538	1.71 (1.41–2.09)	<0.001	0.554	1.74 (1.43–2.11)	<0.001
Respiratory rate (RR)	0.004	1.00 (1.00–1.01)	0.131		—			—	
PEEP	0.037	1.04 (1.02–1.05)	<0.001	0.037	1.04 (1.02–1.05)	<0.001	0.036	1.04 (1.02–1.05)	<0.001
VT/kg	−0.002	1.00 (0.99–1.00)	0.371		—			—	
VE		—		0.006	1.01 (0.99–1.03)	0.544		—	
VR		—			—		0.013	1.01 (0.91–1.13)	0.804

Supplementary Table S17. B. Sensitivity analysis: multivariable linear time-dependent Cox regression model for ICU mortality excluding patients with septic shock at baseline — pressure-controlled ventilation group.

Variables	Model 4 (ΔP)			Model 5 (4×ΔP + RR)		
	β	HR (95% CI)	P-value	β	HR (95% CI)	P-value
Systemic glucocorticoid	0.344	1.41 (1.15–1.74)	0.001	0.344	1.41 (1.15–1.74)	0.001
Neuromuscular blocker	0.535	1.71 (1.40–2.08)	< 0.001	0.539	1.71 (1.41–2.09)	< 0.001
Respiratory rate (RR)	0.004	1.00 (1.00–1.01)	0.145		—	
PEEP	0.037	1.04 (1.02–1.05)	< 0.001	0.037	1.04 (1.02–1.05)	< 0.001
ΔP	0.004	1.00 (1.00–1.01)	0.316		—	
(4×ΔP + RR)		—		0.002	1.00 (1.00–1.00)	0.239

Supplemental Figures S3–S5. Non-linear associations of PEEP with ICU mortality across additional multivariable models

Figure legend: PEEP curves from Model 2 (S3), Model 3 (S4), and Model 5 (S5). HRs were modeled using quadratic polynomial terms in time-dependent Cox models, adjusted for the same covariates as the main text (see Figure 4 legend). Solid lines = adjusted HRs; shaded areas = 95% CIs; dashed line = HR = 1. P-values shown are for the quadratic (non-linear) term, testing deviation from linearity. A non-significant non-linear P-value does not exclude a linear association (see Table 3).The curves are nearly identical to those presented in the main text (Figure 4A5 and 4B4), confirming the robustness of the PEEP association regardless of the primary respiratory variable included. VE, minute ventilation; VR, ventilatory ratio; ΔP, driving pressure; RR, respiratory rate; PEEP, positive end-expiratory pressure.

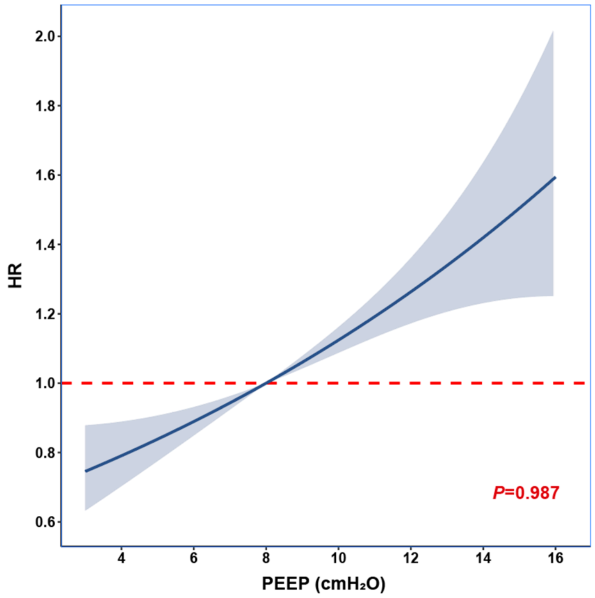

Supplemental Figure S3. Non-linear association of PEEP with ICU mortality — volume-controlled ventilation group, Model 2 (VE model)

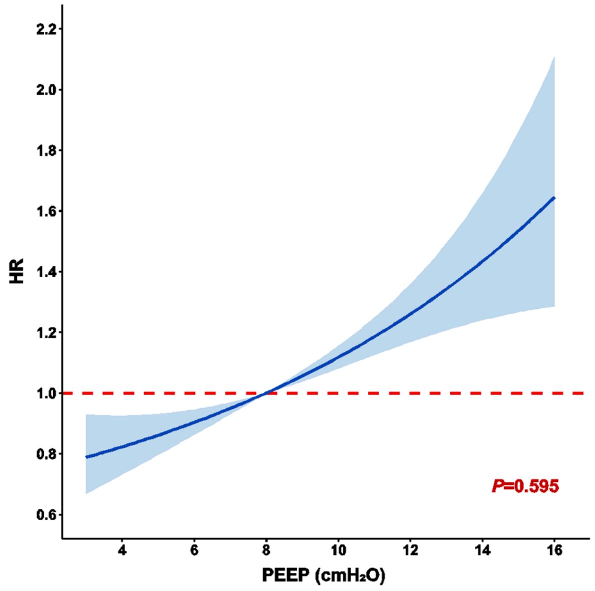

Supplemental Figure S4. Non-linear association of PEEP with ICU mortality — volume-controlled ventilation group, Model 3 (VR model)

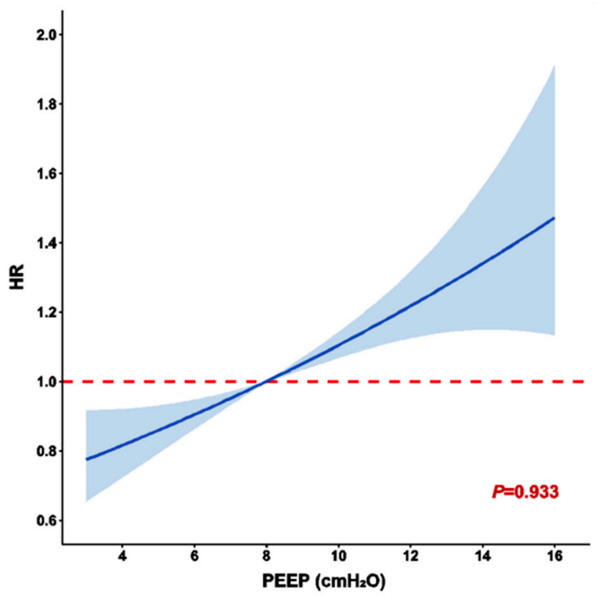

Supplemental Figure S5. Non-linear association of PEEP with ICU mortality — pressure-controlled ventilation group, Model 5 ((4×ΔP+RR) model)

Supplemental Figure S6. Non-linear associations between ventilatory parameters and ICU mortality via restricted cubic splines (RCS) in time-dependent Cox models

Figure legend: HRs were modeled using restricted cubic splines (RCS) in time-dependent Cox models, adjusted for all covariates listed in the Table 3 footnotes. Analyses were stratified by ventilation mode (volume-controlled vs. pressure-controlled). Solid lines = adjusted HRs; shaded areas = 95% CIs; dashed line = HR = 1. P-values shown are for the quadratic (non-linear) term, testing deviation from linearity. A significant non-linear P-value suggests a non-linear association with ICU mortality; a non-significant non-linear P-value does not exclude a linear association.

Panels A1–A7 show results from the volume-controlled ventilation group: A1 VT/kg from Model 1; A2 RR from Model 1; A3 VE from Model 2; A4 VR from Model 3; A5 PEEP from Model 1; A6 PEEP from Model 2; and A7 PEEP from Model 3. Panels B1–B5 show results from the pressure-controlled ventilation group: B1 ΔP from Model 4; B2 RR from Model 4; B3 (4×ΔP + RR) from Model 5; B4 PEEP from Model 4; and B5 PEEP from Model 5.

VT/kg, tidal volume per kg PBW; VE, minute ventilation; VR, ventilatory ratio; ΔP, driving pressure; RR, respiratory rate; PEEP, positive end-expiratory pressure.

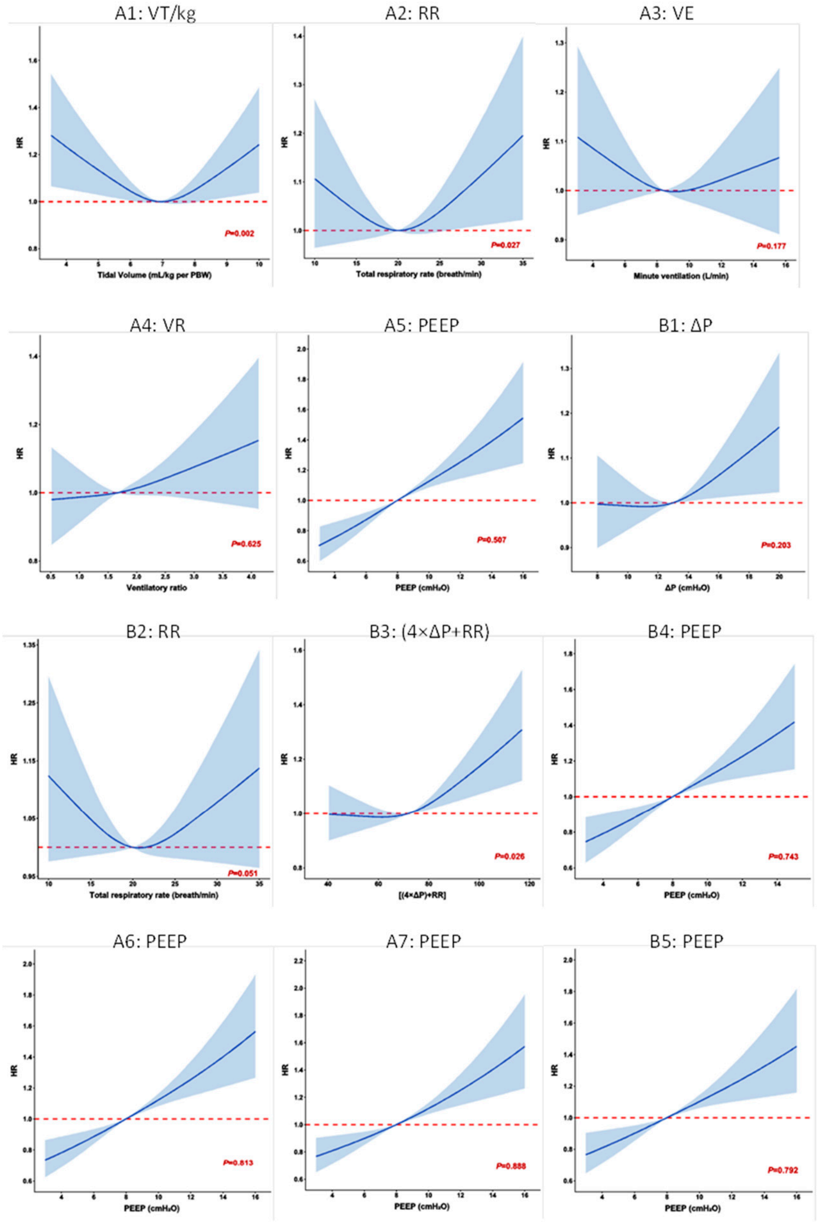

Supplemental Tables S18–S37. Complete results of subgroup analyses for the association between parameters and ICU mortality — multivariable time-dependent Cox regression models stratified by viral infection, fungal infection, hypoxemia severity, and immunocompromised status

Footnote: Data are presented as β coefficient, hazard ratio (HR) with 95% confidence interval (CI), and P-value. HRs were calculated per 1-unit increase for continuous variables. All models were adjusted for the same set of covariates as the primary analysis (see footnote to Table 3A/B in the main text). Time-dependent covariates were handled using the time-varying Cox regression model with repeated measures. VT/kg, tidal volume per kg predicted body weight; VE, minute ventilation; VR, ventilatory ratio; ΔP, driving pressure; RR, respiratory rate; PEEP, positive end-expiratory pressure.

Table S18. Subgroup analysis stratified by viral infection status (viral vs. non-viral), Model 1 (VT/kg)

	No virus					Virus			
Variables	*β*	*Se*	*P*	*HR* (95% *CI*)		*β*	*Se*	*P*	*HR* (95% *CI*)
Age	$2.36*10^{-2}$	$3.00*10^{-3}$	<0.001	1.02 (1.02 ~ 1.03)		$1.37*10^{-2}$	$4.11*10^{-3}$	0.001	1.01 (1.01 ~ 1.02)
Non-pulmonary SOFA score	$8.04*10^{-2}$	$1.41*10^{-2}$	<0.001	1.08 (1.05 ~ 1.11)		$8.87*10^{-2}$	$2.39*10^{-2}$	<0.001	1.09 (1.04 ~ 1.15)
Septic shock	$-5.35*10^{-2}$	$9.77*10^{-2}$	0.584	0.95 (0.78 ~ 1.15)		$1.34*10^{-2}$	$1.39*10^{-1}$	0.923	1.01 (0.77 ~ 1.33)
Chronic lung disease	$1.06*10^{-1}$	$8.64*10^{-2}$	0.218	1.11 (0.94 ~ 1.32)		$-3.60*10^{-2}$	$1.38*10^{-1}$	0.795	0.96 (0.74 ~ 1.26)
Cardiac disease	$2.40*10^{-2}$	$9.75*10^{-2}$	0.805	1.02 (0.85 ~ 1.24)		$6.55*10^{-2}$	$1.72*10^{-1}$	0.704	1.07 (0.76 ~ 1.50)
Neurologic disease	$-1.58*10^{-1}$	$9.80*10^{-2}$	0.108	0.85 (0.70 ~ 1.03)		$2.44*10^{-1}$	$1.52*10^{-1}$	0.110	1.28 (0.95 ~ 1.72)
Liver disease	$2.08*10^{-1}$	$1.75*10^{-1}$	0.235	1.23 (0.87 ~ 1.73)		$2.87*10^{-1}$	$3.47*10^{-1}$	0.409	1.33 (0.68 ~ 2.63)
Chronic kidney disease	$-2.31*10^{-1}$	$1.33*10^{-1}$	0.082	0.79 (0.61 ~ 1.03)		$-1.56*10^{-1}$	$1.84*10^{-1}$	0.396	0.86 (0.60 ~ 1.23)
Immunocompromised status	$1.69*10^{-1}$	$8.17*10^{-2}$	0.038	1.18 (1.01 ~ 1.39)		$1.87*10^{-1}$	$1.24*10^{-1}$	0.132	1.21 (0.95 ~ 1.54)
Bacterial infection	$-9.16*10^{-2}$	$1.41*10^{-1}$	0.514	0.91 (0.69 ~ 1.20)		$-2.10*10^{-1}$	$1.48*10^{-1}$	0.155	0.81 (0.61 ~ 1.08)
Fungal infection	$1.78*10^{-1}$	$8.28*10^{-2}$	0.032	1.19 (1.02 ~ 1.41)		$2.20*10^{-1}$	$1.33*10^{-1}$	0.099	1.25 (0.96 ~ 1.62)
Systemic glucocorticoid	$3.98*10^{-1}$	$9.16*10^{-2}$	<0.001	1.49 (1.24 ~ 1.78)		$1.13*10^{-1}$	$1.50*10^{-1}$	0.451	1.12 (0.83 ~ 1.50)
Neuromuscular blocker	$4.43*10^{-1}$	$9.66*10^{-2}$	<0.001	1.56 (1.29 ~ 1.88)		$3.86*10^{-1}$	$1.25*10^{-1}$	0.002	1.47 (1.15 ~ 1.88)
Mean arterial pressure (MAP)	$4.42*10^{-9}$	$5.07*10^{-10}$	<0.001	1.00 (1.00 ~ 1.00)		$1.64*10^{-4}$	$1.67*10^{-4}$	0.331	1.00 (1.00 ~ 1.00)
Heart rate (HR)	$7.02*10^{-3}$	$9.89*10^{-4}$	<0.001	1.01 (1.01 ~ 1.01)		$9.61*10^{-3}$	$1.59*10^{-3}$	<0.001	1.01 (1.01 ~ 1.01)
Respiratory rate (RR)	$3.00*10^{-3}$	$2.02*10^{-3}$	0.141	1.00 (1.00 ~ 1.01)		$2.25*10^{-3}$	$3.11*10^{-3}$	0.471	1.00 (1.00 ~ 1.01)
PEEP	$5.41*10^{-2}$	$8.87*10^{-3}$	<0.001	1.06 (1.04 ~ 1.07)		$2.24*10^{-2}$	$8.57*10^{-3}$	0.009	1.02 (1.01 ~ 1.04)

	No virus					Virus			
**Variables**	***β***	***Se***	***P***	***HR* (95% *CI*)**		***β***	***Se***	***P***	***HR* (95% *CI*)**
Tidal volume per body weight (VT/kg)	$-2.58*10^{-3}$	$1.21*10^{-3}$	0.043	1.00 (1.00 ~ 1.00)		$-1.03*10^{-3}$	$7.05*10^{-3}$	0.886	1.00 (0.99 ~ 1.01)
Fluid balance (input − output)	$1.41*10^{-11}$	$1.02*10^{-11}$	0.237	1.00 (1.00 ~ 1.00)		$3.29*10^{-5}$	$2.41*10^{-5}$	0.172	1.00 (1.00 ~ 1.00)
PaO_2/FiO_2 ratio	$-1.24*10^{-3}$	$2.27*10^{-4}$	<0.001	1.00 (1.00 ~ 1.00)		$-1.08*10^{-3}$	$3.76*10^{-4}$	0.004	1.00 (1.00 ~ 1.00)
PaCO2	$4.73*10^{-3}$	$2.50*10^{-3}$	0.062	1.00 (1.00 ~ 1.01)		$6.01*10^{-3}$	$3.54*10^{-3}$	0.092	1.01 (1.00 ~ 1.01)
pH	$-7.41*10^{-1}$	$2.79*10^{-1}$	0.009	0.48 (0.28 ~ 0.82)		$-8.46*10^{-2}$	$5.77*10^{-1}$	0.885	0.92 (0.30 ~ 2.85)
Lactate	$1.16*10^{-1}$	$1.58*10^{-2}$	<0.001	1.12 (1.09 ~ 1.16)		$1.18*10^{-1}$	$2.23*10^{-2}$	<0.001	1.12 (1.08 ~ 1.18)
C-reactive protein (CRP)	$1.22*10^{-3}$	$3.19*10^{-4}$	0.001	1.00 (1.00 ~ 1.00)		$4.30*10^{-4}$	$3.67*10^{-4}$	0.242	1.00 (1.00 ~ 1.00)
Interleukin-6 (IL-6)	$1.06*10^{-4}$	$2.66*10^{-5}$	<0.001	1.00 (1.00 ~ 1.00)		$1.78*10^{-4}$	$3.79*10^{-5}$	<0.001	1.00 (1.00 ~ 1.00)
Procalcitonin (PCT)	$-2.48*10^{-3}$	$1.96*10^{-3}$	0.210	1.00 (0.99 ~ 1.00)		$-9.08*10^{-5}$	$2.94*10^{-3}$	0.975	1.00 (0.99 ~ 1.01)
B-type natriuretic peptide (BNP)	$9.68*10^{-6}$	$3.22*10^{-6}$	0.003	1.00 (1.00 ~ 1.00)		$8.19*10^{-6}$	$5.41*10^{-6}$	0.133	1.00 (1.00 ~ 1.00)
Albumin	$-1.18*10^{-2}$	$4.50*10^{-3}$	0.009	0.99 (0.98 ~ 1.00)		$-2.55*10^{-3}$	$6.90*10^{-3}$	0.713	1.00 (0.98 ~ 1.01)
Absolute neutrophil count (ANC)	$1.88*10^{-2}$	$3.97*10^{-3}$	<0.001	1.02 (1.01 ~ 1.03)		$1.82*10^{-2}$	$7.27*10^{-3}$	0.013	1.02 (1.00 ~ 1.03)
Absolute lymphocyte count (ALC)	$-7.78*10^{-2}$	$4.59*10^{-2}$	0.091	0.93 (0.85 ~ 1.01)		$-5.69*10^{-2}$	$5.24*10^{-2}$	0.278	0.94 (0.85 ~ 1.05)
Hemoglobin (Hb)	$-9.67*10^{-4}$	$1.43*10^{-3}$	0.501	1.00 (1.00 ~ 1.00)		$-8.99*10^{-4}$	$2.11*10^{-3}$	0.671	1.00 (0.99 ~ 1.00)
D-dimer	$1.36*10^{-3}$	$3.30*10^{-3}$	0.695	1.00 (0.99 ~ 1.01)		$-2.55*10^{-4}$	$8.49*10^{-4}$	0.764	1.00 (1.00 ~ 1.00)
Creatinine (Cr)	$8.54*10^{-4}$	$2.92*10^{-4}$	0.004	1.00 (1.00 ~ 1.00)		$3.31*10^{-4}$	$4.66*10^{-4}$	0.478	1.00 (1.00 ~ 1.00)
Platelet count (PLT)	$-1.37*10^{-3}$	$3.16*10^{-4}$	<0.001	1.00 (1.00 ~ 1.00)		$-1.16*10^{-3}$	$5.26*10^{-4}$	0.029	1.00 (1.00 ~ 1.00)
Time (day)	$2.11*10^{-2}$	$3.12*10^{-3}$	<0.001	1.02 (1.02 ~ 1.03)		$1.37*10^{-2}$	$5.26*10^{-3}$	0.010	1.01 (1.00 ~ 1.02)

For covariate definitions and abbreviations, see the note to Tables S18–S37.

Table S19. Subgroup analysis stratified by viral infection status (viral vs. non-viral), Model 2 (VE)

	No virus				Virus			
**Variables**	***β***	***Se***	***P***	***HR* (95% *CI*)**	***β***	***Se***	***P***	***HR* (95% *CI*)**
Age	$2.36*10^{-2}$	$3.00*10^{-3}$	<0.001	1.02 (1.02 ~ 1.03)	$1.38*10^{-2}$	$4.11*10^{-3}$	0.001	1.01 (1.01 ~ 1.02)
Non-pulmonary SOFA score	$8.03*10^{-2}$	$1.41*10^{-2}$	<0.001	1.08 (1.05 ~ 1.11)	$8.83*10^{-2}$	$2.40*10^{-2}$	<0.001	1.09 (1.04 ~ 1.14)
Septic shock	$-5.36*10^{-2}$	$9.78*10^{-2}$	0.584	0.95 (0.78 ~ 1.15)	$1.29*10^{-2}$	$1.39*10^{-1}$	0.926	1.01 (0.77 ~ 1.33)
Chronic lung disease	$1.08*10^{-1}$	$8.63*10^{-2}$	0.212	1.11 (0.94 ~ 1.32)	$-3.65*10^{-2}$	$1.38*10^{-1}$	0.791	0.96 (0.74 ~ 1.26)
Cardiac disease	$2.38*10^{-2}$	$9.76*10^{-2}$	0.807	1.02 (0.85 ~ 1.24)	$6.50*10^{-2}$	$1.72*10^{-1}$	0.706	1.07 (0.76 ~ 1.49)
Neurologic disease	$-1.58*10^{-1}$	$9.81*10^{-2}$	0.108	0.85 (0.70 ~ 1.04)	$2.42*10^{-1}$	$1.53*10^{-1}$	0.113	1.27 (0.94 ~ 1.72)
Liver disease	$2.09*10^{-1}$	$1.75*10^{-1}$	0.234	1.23 (0.87 ~ 1.74)	$2.88*10^{-1}$	$3.46*10^{-1}$	0.406	1.33 (0.68 ~ 2.63)
Chronic kidney disease	$-2.30*10^{-1}$	$1.33*10^{-1}$	0.083	0.79 (0.61 ~ 1.03)	$-1.57*10^{-1}$	$1.84*10^{-1}$	0.394	0.85 (0.60 ~ 1.23)
Immunocompromised status	$1.70*10^{-1}$	$8.17*10^{-2}$	0.037	1.19 (1.01 ~ 1.39)	$1.87*10^{-1}$	$1.24*10^{-1}$	0.133	1.21 (0.94 ~ 1.54)
Bacterial infection	$-9.01*10^{-2}$	$1.41*10^{-1}$	0.522	0.91 (0.69 ~ 1.20)	$-2.07*10^{-1}$	$1.48*10^{-1}$	0.164	0.81 (0.61 ~ 1.09)
Fungal infection	$1.77*10^{-1}$	$8.28*10^{-2}$	0.033	1.19 (1.01 ~ 1.40)	$2.20*10^{-1}$	$1.34*10^{-1}$	0.100	1.25 (0.96 ~ 1.62)
Systemic glucocorticoid	$3.98*10^{-1}$	$9.16*10^{-2}$	<0.001	1.49 (1.24 ~ 1.78)	$1.16*10^{-1}$	$1.50*10^{-1}$	0.437	1.12 (0.84 ~ 1.51)
Neuromuscular blocker	$4.46*10^{-1}$	$9.65*10^{-2}$	<0.001	1.56 (1.29 ~ 1.89)	$3.91*10^{-1}$	$1.25*10^{-1}$	0.002	1.48 (1.16 ~ 1.89)
Mean arterial pressure (MAP)	$4.42*10^{-9}$	$5.07*10^{-10}$	<0.001	1.00 (1.00 ~ 1.00)	$1.65*10^{-4}$	$1.64*10^{-4}$	0.318	1.00 (1.00 ~ 1.00)
Heart rate (HR)	$7.07*10^{-3}$	$9.95*10^{-4}$	<0.001	1.01 (1.01 ~ 1.01)	$9.75*10^{-3}$	$1.62*10^{-3}$	<0.001	1.01 (1.01 ~ 1.01)
PEEP	$5.42*10^{-2}$	$8.86*10^{-3}$	<0.001	1.06 (1.04 ~ 1.07)	$2.24*10^{-2}$	$8.57*10^{-3}$	0.009	1.02 (1.01 ~ 1.04)
Minute ventilation (VE)	$3.14*10^{-3}$	$7.00*10^{-3}$	0.654	1.00 (0.99 ~ 1.02)	$-1.18*10^{-3}$	$1.19*10^{-2}$	0.922	1.00 (0.98 ~ 1.02)
Fluid balance (input − output)	$1.41*10^{-11}$	$1.02*10^{-11}$	0.237	1.00 (1.00 ~ 1.00)	$3.32*10^{-5}$	$2.40*10^{-5}$	0.167	1.00 (1.00 ~ 1.00)
PaO_2/FiO_2 ratio	$-1.25*10^{-3}$	$2.28*10^{-4}$	<0.001	1.00 (1.00 ~ 1.00)	$-1.09*10^{-3}$	$3.78*10^{-4}$	0.004	1.00 (1.00 ~ 1.00)
PaCO2	$4.76*10^{-3}$	$2.50*10^{-3}$	0.061	1.00 (1.00 ~ 1.01)	$6.12*10^{-3}$	$3.51*10^{-3}$	0.084	1.01 (1.00 ~ 1.01)
pH	$-7.47*10^{-1}$	$2.78*10^{-1}$	0.008	0.47 (0.27 ~ 0.82)	$-8.95*10^{-2}$	$5.75*10^{-1}$	0.878	0.91 (0.30 ~ 2.82)

	No virus					Virus			
**Variables**	***β***	***Se***	***P***	***HR* (95% *CI*)**		***β***	***Se***	***P***	***HR* (95% *CI*)**
Lactate	$1.16*10^{-1}$	$1.57*10^{-2}$	<0.001	1.12 (1.09 ~ 1.16)		$1.17*10^{-1}$	$2.25*10^{-2}$	<0.001	1.12 (1.08 ~ 1.18)
C-reactive protein (CRP)	$1.22*10^{-3}$	$3.22*10^{-4}$	0.001	1.00 (1.00 ~ 1.00)		$4.33*10^{-4}$	$3.68*10^{-4}$	0.240	1.00 (1.00 ~ 1.00)
Interleukin-6 (IL-6)	$1.07*10^{-4}$	$2.67*10^{-5}$	<0.001	1.00 (1.00 ~ 1.00)		$1.79*10^{-4}$	$3.81*10^{-5}$	<0.001	1.00 (1.00 ~ 1.00)
Procalcitonin (PCT)	$-2.49*10^{-3}$	$1.96*10^{-3}$	0.208	1.00 (0.99 ~ 1.00)		$-1.10*10^{-4}$	$2.94*10^{-3}$	0.970	1.00 (0.99 ~ 1.01)
B-type natriuretic peptide (BNP)	$9.67*10^{-6}$	$3.23*10^{-6}$	0.003	1.00 (1.00 ~ 1.00)		$8.29*10^{-6}$	$5.40*10^{-6}$	0.127	1.00 (1.00 ~ 1.00)
Albumin	$-1.18*10^{-2}$	$4.49*10^{-3}$	0.009	0.99 (0.98 ~ 1.00)		$-2.58*10^{-3}$	$6.90*10^{-3}$	0.709	1.00 (0.98 ~ 1.01)
Absolute neutrophil count (ANC)	$1.88*10^{-2}$	$3.96*10^{-3}$	<0.001	1.02 (1.01 ~ 1.03)		$1.82*10^{-2}$	$7.24*10^{-3}$	0.013	1.02 (1.00 ~ 1.03)
Absolute lymphocyte count (ALC)	$-7.85*10^{-2}$	$4.60*10^{-2}$	0.089	0.92 (0.84 ~ 1.01)		$-5.74*10^{-2}$	$5.24*10^{-2}$	0.274	0.94 (0.85 ~ 1.05)
Hemoglobin (Hb)	$-9.86*10^{-4}$	$1.43*10^{-3}$	0.491	1.00 (1.00 ~ 1.00)		$-9.24*10^{-4}$	$2.11*10^{-3}$	0.662	1.00 (0.99 ~ 1.00)
D-dimer	$1.36*10^{-3}$	$3.30*10^{-3}$	0.695	1.00 (0.99 ~ 1.01)		$-2.61*10^{-4}$	$1.01*10^{-3}$	0.796	1.00 (1.00 ~ 1.00)
Creatinine (Cr)	$8.50*10^{-4}$	$2.92*10^{-4}$	0.004	1.00 (1.00 ~ 1.00)		$3.29*10^{-4}$	$4.66*10^{-4}$	0.481	1.00 (1.00 ~ 1.00)
Platelet count (PLT)	$-1.36*10^{-3}$	$3.16*10^{-4}$	<0.001	1.00 (1.00 ~ 1.00)		$-1.16*10^{-3}$	$5.25*10^{-4}$	0.029	1.00 (1.00 ~ 1.00)
Time (day)	$2.14*10^{-2}$	$3.11*10^{-3}$	<0.001	1.02 (1.02 ~ 1.03)		$1.41*10^{-2}$	$5.31*10^{-3}$	0.009	1.01 (1.00 ~ 1.02)

For covariate definitions and abbreviations, see the note to Tables S18–S37.

Table S20. Subgroup analysis stratified by viral infection status (viral vs. non-viral), Model 3 (VR)

	No virus				Virus			
**Variables**	***β***	***Se***	***P***	***HR* (95% *CI*)**	***β***	***Se***	***P***	***HR* (95% *CI*)**
Age	$2.36*10^{-2}$	$2.99*10^{-3}$	<0.001	1.02 (1.02 ~1.03)	$1.41*10^{-2}$	$4.13*10^{-3}$	0.001	1.01 (1.01 ~1.02)
Non-pulmonary SOFA score	$7.91*10^{-2}$	$1.41*10^{-2}$	<0.001	1.08 (1.05 ~1.11)	$8.82*10^{-2}$	$2.40*10^{-2}$	<0.001	1.09 (1.04 ~1.14)
Septic shock	$-5.10*10^{-2}$	$9.78*10^{-2}$	0.602	0.95 (0.78 ~1.15)	$6.95*10^{-3}$	$1.38*10^{-1}$	0.960	1.01 (0.77 ~1.32)
Chronic lung disease	$1.26*10^{-1}$	$8.55*10^{-2}$	0.141	1.13 (0.96 ~1.34)	$-2.80*10^{-2}$	$1.38*10^{-1}$	0.839	0.97 (0.74 ~1.27)
Cardiac disease	$2.38*10^{-2}$	$9.76*10^{-2}$	0.808	1.02 (0.85 ~1.24)	$6.03*10^{-2}$	$1.72*10^{-1}$	0.726	1.06 (0.76 ~1.49)
Neurologic disease	$-1.60*10^{-1}$	$9.79*10^{-2}$	0.103	0.85 (0.70 ~1.03)	$2.45*10^{-1}$	$1.53*10^{-1}$	0.109	1.28 (0.95 ~1.72)
Liver disease	$2.09*10^{-1}$	$1.74*10^{-1}$	0.23	1.23 (0.88 ~1.73)	$3.11*10^{-1}$	$3.40*10^{-1}$	0.360	1.37 (0.70 ~2.66)
Chronic kidney disease	$-2.32*10^{-1}$	$1.33*10^{-1}$	0.081	0.79 (0.61 ~1.03)	$-1.64*10^{-1}$	$1.84*10^{-1}$	0.373	0.85 (0.59 ~1.22)
Immunocompromised status	$1.72*10^{-1}$	$8.17*10^{-2}$	0.035	1.19 (1.01 ~1.39)	$1.87*10^{-1}$	$1.24*10^{-1}$	0.134	1.21 (0.94 ~1.54)
Bacterial infection	$-8.53*10^{-2}$	$1.41*10^{-1}$	0.545	0.92 (0.70 ~1.21)	$-2.12*10^{-1}$	$1.47*10^{-1}$	0.150	0.81 (0.61 ~1.08)
Fungal infection	$1.83*10^{-1}$	$8.27*10^{-2}$	0.027	1.20 (1.02 ~1.41)	$2.20*10^{-1}$	$1.34*10^{-1}$	0.100	1.25 (0.96 ~1.62)
Systemic glucocorticoid	$4.01*10^{-1}$	$9.15*10^{-2}$	<0.001	1.49 (1.25 ~1.79)	$1.16*10^{-1}$	$1.50*10^{-1}$	0.440	1.12 (0.84 ~1.51)
Neuromuscular blocker	$4.59*10^{-1}$	$9.58*10^{-2}$	<0.001	1.58 (1.31 ~1.91)	$3.98*10^{-1}$	$1.25*10^{-1}$	0.002	1.49 (1.16 ~1.90)
Mean arterial pressure (MAP)	$4.41*10^{-9}$	$5.08*10^{-10}$	<0.001	1.00 (1.00 ~ 1.00)	$1.61*10^{-4}$	$1.69*10^{-4}$	0.344	1.00 (1.00 ~ 1.00)
Heart rate (HR)	$7.21*10^{-3}$	$9.90*10^{-4}$	<0.001	1.01 (1.01 ~1.01)	$9.57*10^{-3}$	$1.61*10^{-3}$	<0.001	1.01 (1.01 ~1.01)
PEEP	$5.44*10^{-2}$	$8.86*10^{-3}$	<0.001	1.06 (1.04 ~1.07)	$2.23*10^{-2}$	$8.70*10^{-3}$	0.011	1.02 (1.01 ~1.04)
Ventilatory ratio (VR)	$-9.54*10^{-3}$	$9.82*10^{-3}$	0.333	0.99 (0.97 ~1.01)	$5.24*10^{-2}$	$6.27*10^{-2}$	0.416	1.05 (0.93 ~1.19)
Fluid balance (input − output)	$1.41*10^{-11}$	$1.02*10^{-11}$	0.237	1.00 (1.00 ~ 1.00)	$2.93*10^{-5}$	$2.39*10^{-5}$	0.221	1.00 (1.00 ~ 1.00)
PaO_2/FiO_2 ratio	$-1.37*10^{-3}$	$2.36*10^{-4}$	<0.001	1.00 (1.00 ~ 1.00)	$-1.18*10^{-3}$	$3.79*10^{-4}$	0.002	1.00 (1.00 ~ 1.00)
pH	-1.15	$2.01*10^{-1}$	<0.001	0.32 (0.21 ~ 0.47)	$-4.47*10^{-1}$	$5.23*10^{-1}$	0.412	0.64 (0.23 ~1.78)
Lactate	$1.12*10^{-1}$	$1.46*10^{-2}$	<0.001	1.12 (1.09 ~ 1.15)	$1.15*10^{-1}$	$2.15*10^{-2}$	<0.001	1.12 (1.08 ~1.17)

	No virus					Virus			
Variables	*β*	*Se*	*P*	*HR* (95% *CI*)		*β*	*Se*	*P*	*HR* (95% *CI*)
C-reactive protein (CRP)	$1.23*10^{-3}$	$3.22*10^{-4}$	0.001	1.00 (1.00 ~ 1.00)		$4.26*10^{-4}$	$3.67*10^{-4}$	0.247	1.00 (1.00 ~ 1.00)
Interleukin-6 (IL-6)	$1.07*10^{-4}$	$2.65*10^{-5}$	<0.001	1.00 (1.00 ~ 1.00)		$1.77*10^{-4}$	$3.81*10^{-5}$	<0.001	1.00 (1.00 ~ 1.00)
Procalcitonin (PCT)	$-2.68*10^{-3}$	$1.98*10^{-3}$	0.178	1.00 (0.99 ~ 1.00)		$-7.35*10^{-5}$	$2.91*10^{-3}$	0.980	1.00 (0.99 ~1.01)
B-type natriuretic peptide (BNP)	$9.76*10^{-6}$	$3.24*10^{-6}$	0.003	1.00 (1.00 ~ 1.00)		$8.40*10^{-6}$	$5.50*10^{-6}$	0.130	1.00 (1.00 ~ 1.00)
Albumin	$-1.12*10^{-2}$	$4.48*10^{-3}$	0.012	0.99 (0.98 ~ 1.00)		$-2.29*10^{-3}$	$6.90*10^{-3}$	0.741	1.00 (0.98 ~1.01)
Absolute neutrophil count (ANC)	$1.92*10^{-2}$	$3.98*10^{-3}$	<0.001	1.02 (1.01 ~ 1.03)		$1.91*10^{-2}$	$7.31*10^{-3}$	0.010	1.02 (1.00 ~1.03)
Absolute lymphocyte count (ALC)	$-8.13*10^{-2}$	$4.60*10^{-2}$	0.078	0.92 (0.84 ~ 1.01)		$-5.94*10^{-2}$	$5.32*10^{-2}$	0.265	0.94 (0.85 ~1.05)
Hemoglobin (Hb)	$-9.11*10^{-4}$	$1.43*10^{-3}$	0.525	1.00 (1.00 ~ 1.00)		$-8.27*10^{-4}$	$2.12*10^{-3}$	0.697	1.00 (1.00 ~ 1.00)
D-dimer	$1.36*10^{-3}$	$3.31*10^{-3}$	0.695	1.00 (0.99 ~1.01)		$-2.47*10^{-4}$	$7.01*10^{-4}$	0.725	1.00 (1.00 ~ 1.00)
Creatinine (Cr)	$7.92*10^{-4}$	$2.88*10^{-4}$	0.006	1.00 (1.00 ~ 1.00)		$2.71*10^{-4}$	$4.71*10^{-4}$	0.566	1.00 (1.00 ~ 1.00)
Platelet count (PLT)	$-1.37*10^{-3}$	$3.16*10^{-4}$	<0.001	1.00 (1.00 ~ 1.00)		$-1.18*10^{-3}$	$5.28*10^{-4}$	0.028	1.00 (1.00 ~ 1.00)
Time (day)	$2.28*10^{-2}$	$3.12*10^{-3}$	<0.001	1.02 (1.02 ~1.03)		$1.42*10^{-2}$	$5.30*10^{-3}$	0.008	1.01 (1.00 ~1.02)

For covariate definitions and abbreviations, see the note to Tables S18–S37.

Table S21. Subgroup analysis stratified by viral infection status (viral vs. non-viral), Model 4 (ΔP)

	No virus					Virus			
**Variables**	***β***	***Se***	***P***	***HR* (95% *CI*)**		***β***	***Se***	***P***	***HR* (95% *CI*)**
Age	$2.36*10^{-2}$	$3.00*10^{-3}$	<0.001	1.02 (1.02 ~ 1.03)		$1.36*10^{-2}$	$4.11*10^{-3}$	0.001	1.01 (1.01 ~ 1.02)
Non-pulmonary SOFA score	$8.04*10^{-2}$	$1.41*10^{-2}$	<0.001	1.08 (1.05 ~ 1.11)		$8.85*10^{-2}$	$2.40*10^{-2}$	<0.001	1.09 (1.04 ~ 1.14)
Septic shock	$-5.34*10^{-2}$	$9.77*10^{-2}$	0.584	0.95 (0.78 ~ 1.15)		$1.39*10^{-2}$	$1.39*10^{-1}$	0.921	1.01 (0.77 ~ 1.33)
Chronic lung disease	$1.07*10^{-1}$	$8.63*10^{-2}$	0.216	1.11 (0.94 ~ 1.32)		$-3.76*10^{-2}$	$1.38*10^{-1}$	0.785	0.96 (0.73 ~ 1.26)
Cardiac disease	$2.32*10^{-2}$	$9.75*10^{-2}$	0.812	1.02 (0.85 ~ 1.24)		$6.57*10^{-2}$	$1.72*10^{-1}$	0.703	1.07 (0.76 ~ 1.50)
Neurologic disease	$-1.58*10^{-1}$	$9.81*10^{-2}$	0.107	0.85 (0.70 ~ 1.03)		$2.43*10^{-1}$	$1.52*10^{-1}$	0.111	1.27 (0.95 ~ 1.72)
Liver disease	$2.09*10^{-1}$	$1.75*10^{-1}$	0.233	1.23 (0.87 ~ 1.74)		$2.87*10^{-1}$	$3.46*10^{-1}$	0.408	1.33 (0.68 ~ 2.63)
Chronic kidney disease	$-2.30*10^{-1}$	$1.33*10^{-1}$	0.084	0.79 (0.61 ~ 1.03)		$-1.60*10^{-1}$	$1.84*10^{-1}$	0.384	0.85 (0.59 ~ 1.22)
Immunocompromised status	$1.69*10^{-1}$	$8.17*10^{-2}$	0.038	1.18 (1.01 ~ 1.39)		$1.84*10^{-1}$	$1.25*10^{-1}$	0.139	1.20 (0.94 ~ 1.54)
Bacterial infection	$-9.13*10^{-2}$	$1.41*10^{-1}$	0.516	0.91 (0.69 ~ 1.20)		$-2.11*10^{-1}$	$1.48*10^{-1}$	0.154	0.81 (0.61 ~ 1.08)
Fungal infection	$1.77*10^{-1}$	$8.29*10^{-2}$	0.032	1.19 (1.01 ~ 1.40)		$2.21*10^{-1}$	$1.34*10^{-1}$	0.099	1.25 (0.96 ~ 1.62)
Systemic glucocorticoid	$3.97*10^{-1}$	$9.16*10^{-2}$	<0.001	1.49 (1.24 ~ 1.78)		$1.10*10^{-1}$	$1.50*10^{-1}$	0.461	1.12 (0.83 ~ 1.5)
Neuromuscular blocker	$4.43*10^{-1}$	$9.66*10^{-2}$	<0.001	1.56 (1.29 ~ 1.88)		$3.88*10^{-1}$	$1.25*10^{-1}$	0.002	1.47 (1.15 ~ 1.88)
Mean arterial pressure (MAP)	$4.42*10^{-9}$	$5.07*10^{-10}$	<0.001	1.00 (1.00 ~ 1.00)		$1.71*10^{-4}$	$1.66*10^{-4}$	0.305	1.00 (1.00 ~ 1.00)
Heart rate (HR)	$7.02*10^{-3}$	$9.88*10^{-4}$	<0.001	1.01 (1.01 ~ 1.01)		$9.61*10^{-3}$	$1.60*10^{-3}$	<0.001	1.01 (1.01 ~ 1.01)
Respiratory rate (RR)	$2.97*10^{-3}$	$2.02*10^{-3}$	0.146	1.00 (1.00 ~ 1.01)		$2.11*10^{-3}$	$3.18*10^{-3}$	0.509	1.00 (1.00 ~ 1.01)
PEEP	$5.40*10^{-2}$	$8.87*10^{-3}$	<0.001	1.06 (1.04 ~ 1.07)		$2.24*10^{-2}$	$8.62*10^{-3}$	0.010	1.02 (1.01 ~ 1.04)
Driving pressure (ΔP)	$1.03*10^{-3}$	$1.56*10^{-3}$	0.510	1.00 (1.00 ~ 1.00)		$5.01*10^{-3}$	$8.14*10^{-3}$	0.543	1.01 (0.99 ~ 1.02)
Fluid balance (input − output)	$1.41*10^{-11}$	$1.02*10^{-11}$	0.237	1.00 (1.00 ~ 1.00)		$3.32*10^{-5}$	$2.40*10^{-5}$	0.166	1.00 (1.00 ~ 1.00)
PaO_2/FiO_2 ratio	$-1.23*10^{-3}$	$2.28*10^{-4}$	<0.001	1.00 (1.00 ~ 1.00)		$-1.07*10^{-3}$	$3.76*10^{-4}$	0.005	1.00 (1.00 ~ 1.00)
PaCO2	$4.70*10^{-3}$	$2.50*10^{-3}$	0.063	1.00 (1.00 ~ 1.01)		$5.97*10^{-3}$	$3.54*10^{-3}$	0.095	1.01 (1.00 ~ 1.01)

	No virus				Virus			
**Variables**	***β***	***Se***	***P***	***HR* (95% *CI*)**	***β***	***Se***	***P***	***HR* (95% *CI*)**
pH	$-7.39*10^{-1}$	$2.79*10^{-1}$	0.009	0.48 (0.28 ~ 0.82)	$-8.22*10^{-2}$	$5.70*10^{-1}$	0.887	0.92 (0.30 ~ 2.82)
Lactate	$1.16*10^{-1}$	$1.57*10^{-2}$	<0.001	1.12 (1.09 ~ 1.16)	$1.18*10^{-1}$	$2.24*10^{-2}$	<0.001	1.12 (1.08 ~ 1.18)
C-reactive protein (CRP)	$1.22*10^{-3}$	$3.21*10^{-4}$	0.001	1.00 (1.00 ~ 1.00)	$4.39*10^{-4}$	$3.67*10^{-4}$	0.232	1.00 (1.00 ~ 1.00)
Interleukin-6 (IL-6)	$1.07*10^{-4}$	$2.67*10^{-5}$	<0.001	1.00 (1.00 ~ 1.00)	$1.79*10^{-4}$	$3.77*10^{-5}$	<0.001	1.00 (1.00 ~ 1.00)
Procalcitonin (PCT)	$-2.49*10^{-3}$	$1.97*10^{-3}$	0.21	1.00 (0.99 ~ 1.00)	$-1.12*10^{-4}$	$2.93*10^{-3}$	0.969	1.00 (0.99 ~ 1.01)
B-type natriuretic peptide (BNP)	$9.64*10^{-6}$	$3.22*10^{-6}$	0.003	1.00 (1.00 ~ 1.00)	$8.17*10^{-6}$	$5.38*10^{-6}$	0.132	1.00 (1.00 ~ 1.00)
Albumin	$-1.18*10^{-2}$	$4.49*10^{-3}$	0.009	0.99 (0.98 ~ 1.00)	$-2.43*10^{-3}$	$6.88*10^{-3}$	0.725	1.00 (0.98 ~ 1.01)
Absolute neutrophil count (ANC)	$1.88*10^{-2}$	$3.96*10^{-3}$	<0.001	1.02 (1.01 ~ 1.03)	$1.82*10^{-2}$	$7.23*10^{-3}$	0.013	1.02 (1.00 ~ 1.03)
Absolute lymphocyte count (ALC)	$-7.79*10^{-2}$	$4.58*10^{-2}$	0.09	0.93 (0.85 ~ 1.01)	$-5.74*10^{-2}$	$5.24*10^{-2}$	0.274	0.94 (0.85 ~ 1.05)
Hemoglobin (Hb)	$-9.67*10^{-4}$	$1.43*10^{-3}$	0.501	1.00 (1.00 ~ 1.00)	$-9.35*10^{-4}$	$2.11*10^{-3}$	0.657	1.00 (0.99 ~ 1.00)
D-dimer	$1.36*10^{-3}$	$3.30*10^{-3}$	0.695	1.00 (0.99 ~ 1.01)	$-2.55*10^{-4}$	$9.26*10^{-4}$	0.783	1.00 (1.00 ~ 1.00)
Creatinine (Cr)	$8.56*10^{-4}$	$2.92*10^{-4}$	0.004	1.00 (1.00 ~ 1.00)	$3.38*10^{-4}$	$4.65*10^{-4}$	0.468	1.00 (1.00 ~ 1.00)
Platelet count (PLT)	$-1.37*10^{-3}$	$3.16*10^{-4}$	<0.001	1.00 (1.00 ~ 1.00)	$-1.16*10^{-3}$	$5.25*10^{-4}$	0.029	1.00 (1.00 ~ 1.00)
Time (day)	$2.11*10^{-2}$	$3.11*10^{-3}$	<0.001	1.02 (1.02 ~ 1.03)	$1.38*10^{-2}$	$5.25*10^{-3}$	0.009	1.01 (1.00 ~ 1.02)

For covariate definitions and abbreviations, see the note to Tables S18–S37.

Table S22. Subgroup analysis stratified by viral infection status (viral vs. non-viral), Model 5 (4×ΔP+RR)

	No virus					Virus			
**Variables**	***β***	***Se***	***P***	***HR* (95% *CI*)**		***β***	***Se***	***P***	***HR* (95% *CI*)**
Age	$2.36*10^{-2}$	$3.00*10^{-3}$	<0.001	1.02 (1.02 ~ 1.03)		$1.36*10^{-2}$	$4.09*10^{-3}$	0.001	1.01 (1.01 ~ 1.02)
Non-pulmonary SOFA score	$8.05*10^{-2}$	$1.41*10^{-2}$	<0.001	1.08 (1.05 ~ 1.11)		$8.85*10^{-2}$	$2.40*10^{-2}$	<0.001	1.09 (1.04 ~ 1.15)
Septic shock	$-5.29*10^{-2}$	$9.77*10^{-2}$	0.588	0.95 (0.78 ~ 1.15)		$1.30*10^{-2}$	$1.39*10^{-1}$	0.925	1.01 (0.77 ~ 1.33)
Chronic lung disease	$1.07*10^{-1}$	$8.64*10^{-2}$	0.216	1.11 (0.94 ~ 1.32)		$-4.08*10^{-2}$	$1.38*10^{-1}$	0.768	0.96 (0.73 ~ 1.26)
Cardiac disease	$2.17*10^{-2}$	$9.76*10^{-2}$	0.824	1.02 (0.84 ~ 1.24)		$6.40*10^{-2}$	$1.72*10^{-1}$	0.710	1.07 (0.76 ~ 1.49)
Neurologic disease	$-1.60*10^{-1}$	$9.83*10^{-2}$	0.104	0.85 (0.70 ~ 1.03)		$2.44*10^{-1}$	$1.52*10^{-1}$	0.110	1.28 (0.95 ~ 1.72)
Liver disease	$2.08*10^{-1}$	$1.75*10^{-1}$	0.237	1.23 (0.87 ~ 1.74)		$2.85*10^{-1}$	$3.46*10^{-1}$	0.410	1.33 (0.67 ~ 2.62)
Chronic kidney disease	$-2.30*10^{-1}$	$1.33*10^{-1}$	0.084	0.79 (0.61 ~ 1.03)		$-1.61*10^{-1}$	$1.84*10^{-1}$	0.382	0.85 (0.59 ~ 1.22)
Immunocompromised status	$1.70*10^{-1}$	$8.17*10^{-2}$	0.038	1.18 (1.01 ~ 1.39)		$1.84*10^{-1}$	$1.24*10^{-1}$	0.139	1.20 (0.94 ~ 1.53)
Bacterial infection	$-9.12*10^{-2}$	$1.41*10^{-1}$	0.517	0.91 (0.69 ~ 1.20)		$-2.10*10^{-1}$	$1.47*10^{-1}$	0.153	0.81 (0.61 ~ 1.08)
Fungal infection	$1.76*10^{-1}$	$8.28*10^{-2}$	0.034	1.19 (1.01 ~ 1.40)		$2.20*10^{-1}$	$1.33*10^{-1}$	0.100	1.25 (0.96 ~ 1.62)
Systemic glucocorticoid	$3.96*10^{-1}$	$9.17*10^{-2}$	<0.001	1.49 (1.24 ~ 1.78)		$1.09*10^{-1}$	$1.50*10^{-1}$	0.469	1.11 (0.83 ~ 1.50)
Neuromuscular blocker	$4.45*10^{-1}$	$9.65*10^{-2}$	<0.001	1.56 (1.29 ~ 1.88)		$3.89*10^{-1}$	$1.24*10^{-1}$	0.002	1.48 (1.16 ~ 1.88)
Mean arterial pressure (MAP)	$4.42*10^{-9}$	$5.06*10^{-10}$	<0.001	1.00 (1.00 ~ 1.00)		$1.64*10^{-4}$	$1.64*10^{-4}$	0.319	1.00 (1.00 ~ 1.00)
Heart rate (HR)	$7.07*10^{-3}$	$9.89*10^{-4}$	<0.001	1.01 (1.01 ~ 1.01)		$9.64*10^{-3}$	$1.59*10^{-3}$	<0.001	1.01 (1.01 ~ 1.01)
PEEP	$5.40*10^{-2}$	$8.89*10^{-3}$	<0.001	1.06 (1.04 ~ 1.07)		$2.23*10^{-2}$	$8.60*10^{-3}$	0.010	1.02 (1.01 ~ 1.04)
[(4×ΔP)+RR]	$6.22*10^{-4}$	$5.10*10^{-4}$	0.244	1.00 (1.00 ~ 1.00)		$1.23*10^{-3}$	$1.31*10^{-3}$	0.365	1.00 (1.00 ~ 1.00)
Fluid balance (input − output)	$1.41*10^{-11}$	$1.03*10^{-11}$	0.237	1.00 (1.00 ~ 1.00)		$3.31*10^{-5}$	$2.40*10^{-5}$	0.169	1.00 (1.00 ~ 1.00)
PaO_2/FiO_2 ratio	$-1.25*10^{-3}$	$2.30*10^{-4}$	<0.001	1.00 (1.00 ~ 1.00)		$-1.07*10^{-3}$	$3.78*10^{-4}$	0.005	1.00 (1.00 ~ 1.00)
PaCO2	$4.67*10^{-3}$	$2.51*10^{-3}$	0.066	1.00 (1.00 ~ 1.01)		$5.77*10^{-3}$	$3.51*10^{-3}$	0.103	1.01 (1.00 ~ 1.01)
pH	$-7.42*10^{-1}$	$2.79*10^{-1}$	0.009	0.48 (0.28 ~ 0.82)		$-9.79*10^{-2}$	$5.75*10^{-1}$	0.867	0.91 (0.29 ~ 2.80)

	No virus				Virus			
**Variables**	***β***	***Se***	***P***	***HR* (95% *CI*)**	***β***	***Se***	***P***	***HR* (95% *CI*)**
Lactate	$1.16*10^{-1}$	$1.57*10^{-2}$	<0.001	1.12 (1.09 ~ 1.16)	$1.17*10^{-1}$	$2.23*10^{-2}$	<0.001	1.12 (1.08 ~ 1.17)
C-reactive protein (CRP)	$1.23*10^{-3}$	$3.22*10^{-4}$	0.001	1.00 (1.00 ~ 1.00)	$4.45*10^{-4}$	$3.68*10^{-4}$	0.227	1.00 (1.00 ~ 1.00)
Interleukin-6 (IL-6)	$1.07*10^{-4}$	$2.69*10^{-5}$	<0.001	1.00 (1.00 ~ 1.00)	$1.78*10^{-4}$	$3.81*10^{-5}$	<0.001	1.00 (1.00 ~ 1.00)
Procalcitonin (PCT)	$-2.50*10^{-3}$	$1.96*10^{-3}$	0.204	1.00 (0.99 ~ 1.00)	$-1.58*10^{-4}$	$2.95*10^{-3}$	0.957	1.00 (0.99 ~ 1.01)
B-type natriuretic peptide (BNP)	$9.66*10^{-6}$	$3.23*10^{-6}$	0.003	1.00 (1.00 ~ 1.00)	$8.13*10^{-6}$	$5.37*10^{-6}$	0.132	1.00 (1.00 ~ 1.00)
Albumin	$-1.17*10^{-2}$	$4.49*10^{-3}$	0.009	0.99 (0.98 ~ 1.00)	$-2.43*10^{-3}$	$6.91*10^{-3}$	0.726	1.00 (0.98 ~ 1.01)
Absolute neutrophil count (ANC)	$1.89*10^{-2}$	$3.96*10^{-3}$	<0.001	1.02 (1.01 ~ 1.03)	$1.82*10^{-2}$	$7.27*10^{-3}$	0.014	1.02 (1.00 ~ 1.03)
Absolute lymphocyte count (ALC)	$-7.85*10^{-2}$	$4.60*10^{-2}$	0.089	0.92 (0.84 ~ 1.01)	$-5.71*10^{-2}$	$5.24*10^{-2}$	0.276	0.94 (0.85 ~ 1.05)
Hemoglobin (Hb)	$-1.01*10^{-3}$	$1.43*10^{-3}$	0.483	1.00 (1.00 ~ 1.00)	$-9.50*10^{-4}$	$2.12*10^{-3}$	0.654	1.00 (0.99 ~ 1.00)
D-dimer	$1.35*10^{-3}$	$3.27*10^{-3}$	0.695	1.00 (0.99 ~ 1.01)	$-2.42*10^{-4}$	$7.24*10^{-4}$	0.739	1.00 (1.00 ~ 1.00)
Creatinine (Cr)	$8.55*10^{-4}$	$2.92*10^{-4}$	0.004	1.00 (1.00 ~ 1.00)	$3.37*10^{-4}$	$4.66*10^{-4}$	0.469	1.00 (1.00 ~ 1.00)
Platelet count (PLT)	$-1.36*10^{-3}$	$3.17*10^{-4}$	<0.001	1.00 (1.00 ~ 1.00)	$-1.16*10^{-3}$	$5.24*10^{-4}$	0.029	1.00 (1.00 ~ 1.00)
Time (day)	$2.15*10^{-2}$	$3.13*10^{-3}$	<0.001	1.02 (1.02 ~ 1.03)	$1.40*10^{-2}$	$5.18*10^{-3}$	0.007	1.01 (1.00 ~ 1.02)

For covariate definitions and abbreviations, see the note to Tables S18–S37.

Table S23. Subgroup analysis stratified by fungal infection status (fungal vs. non-fungal), Model 1 (VT/kg)

	No fungus				Fungus			
**Variables**	***β***	***Se***	***P***	***HR* (95% *CI*)**	***β***	***Se***	***P***	***HR* (95% *CI*)**
Age	$2.62*10^{-2}$	$3.60*10^{-3}$	<0.001	1.03 (1.02 ~ 1.03)	$1.36*10^{-2}$	$3.23*10^{-3}$	<0.001	1.01 (1.01 ~ 1.02)
Non-pulmonary SOFA score	$7.12*10^{-2}$	$1.91*10^{-2}$	<0.001	1.07 (1.03 ~ 1.11)	$9.14*10^{-2}$	$1.54*10^{-2}$	<0.001	1.10 (1.06 ~ 1.13)
Septic shock	$-2.80*10^{-2}$	$1.21*10^{-1}$	0.817	0.97 (0.77 ~ 1.23)	$-4.98*10^{-2}$	$1.05*10^{-1}$	0.636	0.95 (0.77 ~ 1.17)
Chronic lung disease	$6.24*10^{-3}$	$1.12*10^{-1}$	0.956	1.01 (0.81 ~ 1.25)	$1.01*10^{-1}$	$9.60*10^{-2}$	0.293	1.11 (0.92 ~ 1.34)
Cardiac disease	$7.85*10^{-2}$	$1.20*10^{-1}$	0.511	1.08 (0.86 ~ 1.37)	$2.35*10^{-2}$	$1.19*10^{-1}$	0.844	1.02 (0.81 ~ 1.29)
Neurologic disease	$-1.18*10^{-1}$	$1.19*10^{-1}$	0.323	0.89 (0.70 ~ 1.12)	$5.64*10^{-3}$	$1.15*10^{-1}$	0.961	1.01 (0.80 ~ 1.26)
Liver disease	$4.86*10^{-2}$	$2.44*10^{-1}$	0.842	1.05 (0.65 ~ 1.69)	$3.14*10^{-1}$	$2.20*10^{-1}$	0.153	1.37 (0.89 ~ 2.11)
Chronic kidney disease	$-9.37*10^{-2}$	$1.77*10^{-1}$	0.597	0.91 (0.64 ~ 1.29)	$-2.87*10^{-1}$	$1.34*10^{-1}$	0.033	0.75 (0.58 ~ 0.98)
Immunocompromised status	$2.33*10^{-1}$	$1.05*10^{-1}$	0.026	1.26 (1.03 ~ 1.55)	$1.48*10^{-1}$	$8.98*10^{-2}$	0.099	1.16 (0.97 ~ 1.38)
Bacterial infection	$-4.40*10^{-1}$	$2.54*10^{-1}$	0.083	0.64 (0.39 ~ 1.06)	$-1.17*10^{-1}$	$1.10*10^{-1}$	0.289	0.89 (0.72 ~ 1.10)
Viral infection	$-3.96*10^{-2}$	$1.40*10^{-1}$	0.778	0.96 (0.73 ~ 1.27)	$1.23*10^{-2}$	$9.45*10^{-2}$	0.896	1.01 (0.84 ~ 1.22)
Systemic glucocorticoid	$3.84*10^{-1}$	$1.15*10^{-1}$	0.001	1.47 (1.17 ~ 1.84)	$3.04*10^{-1}$	$1.09*10^{-1}$	0.005	1.36 (1.10 ~ 1.68)
Neuromuscular blocker	$5.74*10^{-1}$	$1.17*10^{-1}$	<0.001	1.78 (1.41 ~ 2.23)	$2.86*10^{-1}$	$1.02*10^{-1}$	0.005	1.33 (1.09 ~ 1.62)
Mean arterial pressure (MAP)	$4.64*10^{-9}$	$5.08*10^{-10}$	<0.001	1.00 (1.00 ~ 1.00)	$-2.95*10^{-4}$	$1.65*10^{-3}$	0.858	1.00 (1.00 ~ 1.00)
Heart rate (HR)	$7.65*10^{-3}$	$1.26*10^{-3}$	<0.001	1.01 (1.01 ~ 1.01)	$7.04*10^{-3}$	$1.16*10^{-3}$	<0.001	1.01 (1.00 ~ 1.01)
Respiratory rate (RR)	$4.06*10^{-3}$	$1.84*10^{-3}$	0.036	1.00 (1.00 ~ 1.01)	$1.16*10^{-3}$	$2.59*10^{-3}$	0.655	1.00 (1.00 ~ 1.01)
PEEP	$3.61*10^{-2}$	$8.50*10^{-3}$	<0.001	1.04 (1.02 ~ 1.05)	$3.30*10^{-2}$	$9.97*10^{-3}$	0.001	1.03 (1.01 ~ 1.05)
Tidal volume per body weight (VT/kg)	$-2.72*10^{-3}$	$1.93*10^{-3}$	0.187	1.00 (0.99 ~ 1.00)	$-1.79*10^{-3}$	$3.00*10^{-3}$	0.564	1.00 (0.99 ~ 1.00)
Fluid balance (input − output)	$1.44*10^{-11}$	$1.04*10^{-11}$	0.237	1.00 (1.00 ~ 1.00)	$4.78*10^{-5}$	$1.81*10^{-5}$	0.009	1.00 (1.00 ~ 1.00)
PaO_2/FiO_2 ratio	$-1.22*10^{-3}$	$2.99*10^{-4}$	<0.001	1.00 (1.00 ~ 1.00)	$-1.15*10^{-3}$	$2.64*10^{-4}$	<0.001	1.00 (1.00 ~ 1.00)
PaCO2	$3.45*10^{-3}$	$2.95*10^{-3}$	0.244	1.00 (1.00 ~ 1.01)	$6.51*10^{-3}$	$2.79*10^{-3}$	0.022	1.01 (1.00 ~ 1.01)

Variables	No fungus					Fungus			
	β	*Se*	*P*	*HR* (95% *CI*)		*β*	*Se*	*P*	*HR* (95% *CI*)
pH	$-8.39*10^{-1}$	$3.76*10^{-1}$	0.029	0.43 (0.21 ~ 0.90)		$-3.40*10^{-1}$	$3.55*10^{-1}$	0.344	0.71 (0.36 ~ 1.43)
Lactate	$1.20*10^{-1}$	$2.00*10^{-2}$	<0.001	1.13 (1.08 ~ 1.17)		$1.07*10^{-1}$	$1.56*10^{-2}$	<0.001	1.11 (1.08 ~ 1.15)
C-reactive protein (CRP)	$1.05*10^{-3}$	$3.95*10^{-4}$	0.012	1.00 (1.00 ~ 1.00)		$8.97*10^{-4}$	$2.72*10^{-4}$	0.001	1.00 (1.00 ~ 1.00)
Interleukin-6 (IL-6)	$1.26*10^{-4}$	$2.80*10^{-5}$	<0.001	1.00 (1.00 ~ 1.00)		$1.26*10^{-4}$	$2.88*10^{-5}$	<0.001	1.00 (1.00 ~ 1.00)
Procalcitonin (PCT)	$-7.89*10^{-4}$	$2.19*10^{-3}$	0.718	1.00 (0.99 ~ 1.00)		$-3.20*10^{-3}$	$2.25*10^{-3}$	0.155	1.00 (0.99 ~ 1.00)
B-type natriuretic peptide (BNP)	$7.81*10^{-6}$	$3.98*10^{-6}$	0.052	1.00 (1.00 ~ 1.00)		$1.04*10^{-5}$	$3.82*10^{-6}$	0.006	1.00 (1.00 ~ 1.00)
Albumin	$-2.81*10^{-3}$	$5.92*10^{-3}$	0.635	1.00 (0.99 ~ 1.01)		$-1.36*10^{-2}$	$4.81*10^{-3}$	0.005	0.99 (0.98 ~ 1.00)
Absolute neutrophil count (ANC)	$1.89*10^{-2}$	$5.62*10^{-3}$	0.001	1.02 (1.01 ~ 1.03)		$1.85*10^{-2}$	$5.03*10^{-3}$	<0.001	1.02 (1.01 ~ 1.03)
Absolute lymphocyte count (ALC)	$-7.27*10^{-2}$	$5.35*10^{-2}$	0.174	0.93 (0.84 ~ 1.03)		$-7.19*10^{-2}$	$4.25*10^{-2}$	0.092	0.93 (0.86 ~ 1.01)
Hemoglobin (Hb)	$-2.10*10^{-3}$	$1.82*10^{-3}$	0.250	1.00 (0.99 ~ 1.00)		$3.47*10^{-4}$	$1.50*10^{-3}$	0.817	1.00 (1.00 ~ 1.00)
D-dimer	$1.22*10^{-3}$	$3.06*10^{-3}$	0.701	1.00 (1.00 ~ 1.01)		$-9.15*10^{-5}$	$1.58*10^{-4}$	0.566	1.00 (1.00 ~ 1.00)
Creatinine (Cr)	$1.12*10^{-3}$	$3.46*10^{-4}$	0.002	1.00 (1.00 ~ 1.00)		$4.77*10^{-4}$	$3.17*10^{-4}$	0.133	1.00 (1.00 ~ 1.00)
Platelet count (PLT)	$-1.52*10^{-3}$	$4.06*10^{-4}$	<0.001	1.00 (1.00 ~ 1.00)		$-1.11*10^{-3}$	$3.50*10^{-4}$	0.002	1.00 (1.00 ~ 1.00)
Time (day)	$1.93*10^{-2}$	$4.18*10^{-3}$	<0.001	1.02 (1.01 ~ 1.03)		$2.15*10^{-2}$	$3.68*10^{-3}$	<0.001	1.02 (1.01 ~ 1.03)

For covariate definitions and abbreviations, see the note to Tables S18–S37.

Table S24. Subgroup analysis stratified by fungal infection status (fungal vs. non-fungal), Model 2 (VE)

	**No fungus**				**Fungus**			
**Variables**	***β***	***Se***	***P***	***HR* (95% *CI*)**	***β***	***Se***	***P***	***HR* (95% *CI*)**
Age	$2.61*10^{-2}$	$3.60*10^{-3}$	<0.001	1.03 (1.02 ~ 1.03)	$1.37*10^{-2}$	$3.23*10^{-3}$	<0.001	1.01 (1.01 ~ 1.02)
Non-pulmonary SOFA score	$7.14*10^{-2}$	$1.91*10^{-2}$	<0.001	1.07 (1.03 ~ 1.12)	$9.12*10^{-2}$	$1.55*10^{-2}$	<0.001	1.10 (1.06 ~ 1.13)
Septic shock	$-3.10*10^{-2}$	$1.21*10^{-1}$	0.798	0.97 (0.76 ~ 1.23)	$-4.90*10^{-2}$	$1.05*10^{-1}$	0.642	0.95 (0.77 ~ 1.17)
Chronic lung disease	$8.70*10^{-3}$	$1.12*10^{-1}$	0.938	1.01 (0.81 ~ 1.26)	$1.01*10^{-1}$	$9.60*10^{-2}$	0.294	1.11 (0.92 ~ 1.33)
Cardiac disease	$7.88*10^{-2}$	$1.20*10^{-1}$	0.511	1.08 (0.86 ~ 1.37)	$2.34*10^{-2}$	$1.19*10^{-1}$	0.844	1.02 (0.81 ~ 1.29)
Neurologic disease	$-1.19*10^{-1}$	$1.19*10^{-1}$	0.320	0.89 (0.70 ~ 1.12)	$5.54*10^{-3}$	$1.15*10^{-1}$	0.962	1.01 (0.80 ~ 1.26)
Liver disease	$4.97*10^{-2}$	$2.44*10^{-1}$	0.838	1.05 (0.65 ~ 1.70)	$3.15*10^{-1}$	$2.19*10^{-1}$	0.152	1.37 (0.89 ~ 2.11)
Chronic kidney disease	$-9.38*10^{-2}$	$1.77*10^{-1}$	0.596	0.91 (0.64 ~ 1.29)	$-2.86*10^{-1}$	$1.34*10^{-1}$	0.033	0.75 (0.58 ~ 0.98)
Immunocompromised status	$2.33*10^{-1}$	$1.05*10^{-1}$	0.026	1.26 (1.03 ~ 1.55)	$1.48*10^{-1}$	$8.97*10^{-2}$	0.098	1.16 (0.97 ~ 1.38)
Bacterial infection	$-4.36*10^{-1}$	$2.55*10^{-1}$	0.087	0.65 (0.39 ~ 1.07)	$-1.16*10^{-1}$	$1.10*10^{-1}$	0.294	0.89 (0.72 ~ 1.11)
Viral infection	$-3.92*10^{-2}$	$1.41*10^{-1}$	0.781	0.96 (0.73 ~ 1.27)	$1.28*10^{-2}$	$9.45*10^{-2}$	0.892	1.01 (0.84 ~ 1.22)
Systemic glucocorticoid	$3.85*10^{-1}$	$1.15*10^{-1}$	0.001	1.47 (1.17 ~ 1.84)	$3.05*10^{-1}$	$1.09*10^{-1}$	0.005	1.36 (1.10 ~ 1.68)
Neuromuscular blocker	$5.79*10^{-1}$	$1.16*10^{-1}$	<0.001	1.78 (1.42 ~ 2.24)	$2.88*10^{-1}$	$1.02*10^{-1}$	0.005	1.33 (1.09 ~ 1.63)
Mean arterial pressure (MAP)	$4.63*10^{-9}$	$5.08*10^{-10}$	<0.001	1.00 (1.00 ~ 1.00)	$-2.86*10^{-4}$	$1.60*10^{-3}$	0.858	1.00 (1.00 ~ 1.00)
Heart rate (HR)	$7.69*10^{-3}$	$1.27*10^{-3}$	<0.001	1.01 (1.01 ~ 1.01)	$7.13*10^{-3}$	$1.18*10^{-3}$	<0.001	1.01 (1.00 ~ 1.01)
PEEP	$3.60*10^{-2}$	$8.49*10^{-3}$	<0.001	1.04 (1.02 ~ 1.05)	$3.32*10^{-2}$	$9.99*10^{-3}$	0.001	1.03 (1.01 ~ 1.05)
Minute ventilation (VE)	$5.98*10^{-3}$	$8.94*10^{-3}$	0.504	1.01 (0.99 ~ 1.02)	$-1.11*10^{-3}$	$8.23*10^{-3}$	0.892	1．00 (0.98 ~ 1.02)
Fluid balance (input − output)	$1.44*10^{-11}$	$1.04*10^{-11}$	0.237	1.00 (1.00 ~ 1.00)	$4.78*10^{-5}$	$1.81*10^{-5}$	0.009	1.00 (1.00 ~ 1.00)
PaO_2/FiO_2 ratio	$-1.23*10^{-3}$	$2.98*10^{-4}$	<0.001	1.00 (1.00 ~ 1.00)	$-1.16*10^{-3}$	$2.64*10^{-4}$	<0.001	1.00 (1.00 ~ 1.00)
PaCO2	$3.51*10^{-3}$	$2.95*10^{-3}$	0.236	1.00 (1.00 ~ 1.01)	$6.55*10^{-3}$	$2.79*10^{-3}$	0.021	1.01 (1.00 ~ 1.01)
pH	$-8.49*10^{-1}$	$3.74*10^{-1}$	0.026	0.43 (0.21 ~ 0.89)	$-3.42*10^{-1}$	$3.55*10^{-1}$	0.341	0.71 (0.35 ~ 1.42)

	No fungus					Fungus			
**Variables**	***β***	***Se***	***P***	***HR* (95% *CI*)**		***β***	***Se***	***P***	***HR* (95% *CI*)**
Lactate	$1.21*10^{-1}$	$1.98*10^{-2}$	<0.001	1.13 (1.09 ~ 1.17)		$1.07*10^{-1}$	$1.57*10^{-2}$	<0.001	1.11 (1.08 ~ 1.15)
C-reactive protein (CRP)	$1.05*10^{-3}$	$3.95*10^{-4}$	0.012	1.00 (1.00 ~ 1.00)		$9.00*10^{-4}$	$2.73*10^{-4}$	0.001	1.00 (1.00 ~ 1.00)
Interleukin-6 (IL-6)	$1.26*10^{-4}$	$2.79*10^{-5}$	<0.001	1.00 (1.00 ~ 1.00)		$1.26*10^{-4}$	$2.88*10^{-5}$	<0.001	1.00 (1.00 ~ 1.00)
Procalcitonin (PCT)	$-8.05*10^{-4}$	$2.18*10^{-3}$	0.712	1.00 (0.99 ~ 1.00)		$-3.20*10^{-3}$	$2.25*10^{-3}$	0.156	1.00 (0.99 ~ 1.00)
B-type natriuretic peptide (BNP)	$7.78*10^{-6}$	$3.99*10^{-6}$	0.053	1.00 (1.00 ~ 1.00)		$1.04*10^{-5}$	$3.82*10^{-6}$	0.006	1.00 (1.00 ~ 1.00)
Albumin	$-2.73*10^{-3}$	$5.91*10^{-3}$	0.645	1.00 (0.99 ~ 1.01)		$-1.36*10^{-2}$	$4.81*10^{-3}$	0.005	0.99 (0.98 ~ 1.00)
Absolute neutrophil count (ANC)	$1.90*10^{-2}$	$5.63*10^{-3}$	0.001	1.02 (1.01 ~ 1.03)		$1.85*10^{-2}$	$5.03*10^{-3}$	<0.001	1.02 (1.01 ~ 1.03)
Absolute lymphocyte count (ALC)	$-7.34*10^{-2}$	$5.37*10^{-2}$	0.172	0.93 (0.84 ~ 1.03)		$-7.23*10^{-2}$	$4.25*10^{-2}$	0.09	0.93 (0.86 ~ 1.01)
Hemoglobin (Hb)	$-2.14*10^{-3}$	$1.81*10^{-3}$	0.238	1.00 (0.99 ~ 1.00)		$3.45*10^{-4}$	$1.50*10^{-3}$	0.818	1.00 (1.00 ~ 1.00)
D-dimer	$1.22*10^{-3}$	$3.07*10^{-3}$	0.701	1.00 (1.00 ~ 1.01)		$-9.11*10^{-5}$	$1.58*10^{-4}$	0.567	1.00 (1.00 ~ 1.00)
Creatinine (Cr)	$1.12*10^{-3}$	$3.45*10^{-4}$	0.002	1.00 (1.00 ~ 1.00)		$4.77*10^{-4}$	$3.16*10^{-4}$	0.132	1.00 (1.00 ~ 1.00)
Platelet count (PLT)	$-1.52*10^{-3}$	$4.05*10^{-4}$	<0.001	1.00 (1.00 ~ 1.00)		$-1.10*10^{-3}$	$3.50*10^{-4}$	0.002	1.00 (1.00 ~ 1.00)
Time (day)	$1.96*10^{-2}$	$4.15*10^{-3}$	<0.001	1.02 (1.01 ~ 1.03)		$2.18*10^{-2}$	$3.69*10^{-3}$	<0.001	1.02 (1.01 ~ 1.03)

For covariate definitions and abbreviations, see the note to Tables S18–S37.

Table S25. Subgroup analysis stratified by fungal infection status (fungal vs. non-fungal), Model 3 (VR)

	No fungus					Fungus			
**Variables**	***β***	***Se***	***P***	***HR* (95% *CI*)**		***β***	***Se***	***P***	***HR* (95% *CI*)**
Age	$2.61*10^{-2}$	$3.59*10^{-3}$	<0.001	1.03 (1.02 ~1.03)		$1.39*10^{-2}$	$3.21*10^{-3}$	<0.001	1.01 (1.01 ~1.02)
Non-pulmonary SOFA score	$7.03*10^{-2}$	$1.91*10^{-2}$	<0.001	1.07 (1.03 ~1.11)		$9.09*10^{-2}$	$1.55*10^{-2}$	<0.001	1.10 (1.06 ~1.13)
Septic shock	$-2.89*10^{-2}$	$1.21*10^{-1}$	0.811	0.97 (0.77 ~1.23)		$-4.92*10^{-2}$	$1.05*10^{-1}$	0.640	0.95 (0.77 ~1.17)
Chronic lung disease	$2.45*10^{-2}$	$1.12*10^{-1}$	0.826	1.02 (0.82 ~1.28)		$1.14*10^{-1}$	$9.55*10^{-2}$	0.233	1.12 (0.93 ~1.35)
Cardiac disease	$7.59*10^{-2}$	$1.20*10^{-1}$	0.527	1.08 (0.85 ~1.36)		$2.19*10^{-2}$	$1.19*10^{-1}$	0.854	1.02 (0.81 ~1.29)
Neurologic disease	$-1.23*10^{-1}$	$1.19*10^{-1}$	0.303	0.88 (0.70 ~1.12)		$4.36*10^{-3}$	$1.15*10^{-1}$	0.970	1.00 (0.80 ~1.26)
Liver disease	$5.45*10^{-2}$	$2.43*10^{-1}$	0.823	1.06 (0.66 ~1.70)		$3.31*10^{-1}$	$2.15*10^{-1}$	0.123	1.39 (0.91 ~2.12)
Chronic kidney disease	$-9.93*10^{-2}$	$1.77*10^{-1}$	0.574	0.91 (0.64 ~1.28)		$-2.90*10^{-1}$	$1.34*10^{-1}$	0.03	0.75 (0.58 ~0.97)
Immunocompromised status	$2.36*10^{-1}$	$1.05*10^{-1}$	0.024	1.27 (1.03 ~1.55)		$1.49*10^{-1}$	$8.95*10^{-2}$	0.097	1.16 (0.97 ~1.38)
Bacterial infection	$-4.21*10^{-1}$	$2.53*10^{-1}$	0.096	0.66 (0.40 ~1.08)		$-1.17*10^{-1}$	$1.10*10^{-1}$	0.284	0.89 (0.72 ~1.10)
Viral infection	$-3.10*10^{-2}$	$1.40*10^{-1}$	0.826	0.97 (0.74 ~1.28)		$1.45*10^{-2}$	$9.46*10^{-2}$	0.879	1.01 (0.84 ~1.22)
Systemic glucocorticoid	$3.88*10^{-1}$	$1.14*10^{-1}$	0.001	1.47 (1.18 ~1.84)		$3.06*10^{-1}$	$1.09*10^{-1}$	0.005	1.36 (1.10 ~1.68)
Neuromuscular blocker	$5.92*10^{-1}$	$1.15*10^{-1}$	<0.001	1.81 (1.44 ~2.27)		$2.99*10^{-1}$	$1.02*10^{-1}$	0.003	1.35 (1.10 ~1.65)
Mean arterial pressure (MAP)	$4.63*10^{-9}$	$5.09*10^{-10}$	<0.001	1.00 (1.00 ~ 1.00)		$-3.39*10^{-4}$	$1.84*10^{-3}$	0.854	1.00 (1.00 ~ 1.00)
Heart rate (HR)	$7.78*10^{-3}$	$1.27*10^{-3}$	<0.001	1.01 (1.01 ~1.01)		$7.06*10^{-3}$	$1.20*10^{-3}$	<0.001	1.01 (1.00 ~1.01)
PEEP	$3.60*10^{-2}$	$8.51*10^{-3}$	<0.001	1.04 (1.02 ~1.05)		$3.25*10^{-2}$	$1.00*10^{-2}$	0.001	1.03 (1.01 ~1.05)
Ventilatory ratio (VR)	$-1.03*10^{-2}$	$1.50*10^{-2}$	0.495	0.99 (0.96 ~1.02)		$3.71*10^{-2}$	$4.88*10^{-2}$	0.455	1.04 (0.94 ~1.14)
Fluid balance (input − output)	$1.43*10^{-11}$	$1.04*10^{-11}$	0.237	1.00 (1.00 ~ 1.00)		$4.32*10^{-5}$	$1.80*10^{-5}$	0.017	1.00 (1.00 ~ 1.00)
PaO_2/FiO_2 ratio	$-1.34*10^{-3}$	$3.04*10^{-4}$	<0.001	1.00 (1.00 ~ 1.00)		$-1.26*10^{-3}$	$2.69*10^{-4}$	<0.001	1.00 (1.00 ~ 1.00)
pH	-1.16	$2.74*10^{-1}$	<0.001	0.31 (0.18 ~0.54)		$-7.88*10^{-1}$	$2.96*10^{-1}$	0.013	0.45 (0.25 ~0.81)
Lactate	$1.18*10^{-1}$	$1.88*10^{-2}$	<0.001	1.13 (1.08 ~1.17)		$1.03*10^{-1}$	$1.45*10^{-2}$	<0.001	1.11 (1.08 ~1.14)

	No fungus					Fungus			
**Variables**	***β***	***Se***	***P***	***HR* (95% *CI*)**		***β***	***Se***	***P***	***HR* (95% *CI*)**
C-reactive protein (CRP)	$1.05*10^{-3}$	$3.96*10^{-4}$	0.012	1.00 (1.00 ~ 1.00)		$9.09*10^{-4}$	$2.75*10^{-4}$	0.001	1.00 (1.00 ~ 1.00)
Interleukin-6 (IL-6)	$1.27*10^{-4}$	$2.76*10^{-5}$	<0.001	1.00 (1.00 ~ 1.00)		$1.25*10^{-4}$	$2.88*10^{-5}$	<0.001	1.00 (1.00 ~ 1.00)
Procalcitonin (PCT)	$-8.51*10^{-4}$	$2.17*10^{-3}$	0.695	1.00 (0.99 ~1.00)		$-3.38*10^{-3}$	$2.27*10^{-3}$	0.136	1.00 (0.99 ~1.00)
B-type natriuretic peptide (BNP)	$7.98*10^{-6}$	$3.98*10^{-6}$	0.047	1.00 (1.00 ~ 1.00)		$1.06*10^{-5}$	$3.83*10^{-6}$	0.006	1.00 (1.00 ~ 1.00)
Albumin	$-2.37*10^{-3}$	$5.93*10^{-3}$	0.689	1.00 (0.99 ~1.01)		$-1.31*10^{-2}$	$4.83*10^{-3}$	0.007	0.99 (0.98 ~1.00)
Absolute neutrophil count (ANC)	$1.93*10^{-2}$	$5.59*10^{-3}$	0.001	1.02 (1.01 ~1.03)		$1.93*10^{-2}$	$5.00*10^{-3}$	<0.001	1.02 (1.01 ~1.03)
Absolute lymphocyte count (ALC)	$-7.50*10^{-2}$	$5.34*10^{-2}$	0.160	0.93 (0.84 ~1.03)		$-7.44*10^{-2}$	$4.25*10^{-2}$	0.081	0.93 (0.85 ~1.01)
Hemoglobin (Hb)	$-2.10*10^{-3}$	$1.81*10^{-3}$	0.249	1.00 (0.99 ~1.00)		$4.75*10^{-4}$	$1.50*10^{-3}$	0.752	1.00 (1.00 ~ 1.00)
D-dimer	$1.25*10^{-3}$	$3.12*10^{-3}$	0.701	1.00 (1.00 ~1.01)		$-9.49*10^{-5}$	$1.59*10^{-4}$	0.555	1.00 (1.00 ~ 1.00)
Creatinine (Cr)	$1.08*10^{-3}$	$3.50*10^{-4}$	0.003	1.00 (1.00 ~ 1.00)		$4.04*10^{-4}$	$3.11*10^{-4}$	0.195	1.00 (1.00 ~ 1.00)
Platelet count (PLT)	$-1.51*10^{-3}$	$4.06*10^{-4}$	<0.001	1.00 (1.00 ~ 1.00)		$-1.12*10^{-3}$	$3.49*10^{-4}$	0.001	1.00 (1.00 ~ 1.00)
Time (day)	$2.08*10^{-2}$	$4.13*10^{-3}$	<0.001	1.02 (1.01 ~1.03)		$2.21*10^{-2}$	$3.58*10^{-3}$	<0.001	1.02 (1.02 ~1.03)

For covariate definitions and abbreviations, see the note to Tables S18–S37.

Table S26. Subgroup analysis stratified by fungal infection status (fungal vs. non-fungal), Model 4 (ΔP)

	No fungus				Fungus			
**Variables**	***β***	***Se***	***P***	***HR* (95% *CI*)**	***β***	***Se***	***P***	***HR* (95% *CI*)**
Age	$2.62*10^{-2}$	$3.60*10^{-3}$	<0.001	1.03 (1.02 ~ 1.03)	$1.36*10^{-2}$	$3.23*10^{-3}$	<0.001	1.01 (1.01 ~ 1.02)
Non-pulmonary SOFA score	$7.13*10^{-2}$	$1.91*10^{-2}$	<0.001	1.07 (1.03 ~ 1.11)	$9.14*10^{-2}$	$1.54*10^{-2}$	<0.001	1.10 (1.06 ~ 1.13)
Septic shock	$-2.93*10^{-2}$	$1.21*10^{-1}$	0.809	0.97 (0.77 ~ 1.23)	$-4.93*10^{-2}$	$1.05*10^{-1}$	0.640	0.95 (0.77 ~ 1.17)
Chronic lung disease	$6.45*10^{-3}$	$1.12*10^{-1}$	0.954	1.01 (0.81 ~ 1.25)	$1.01*10^{-1}$	$9.60*10^{-2}$	0.294	1.11 (0.92 ~ 1.33)
Cardiac disease	$7.70*10^{-2}$	$1.20*10^{-1}$	0.520	1.08 (0.85 ~ 1.37)	$2.34*10^{-2}$	$1.19*10^{-1}$	0.845	1.02 (0.81 ~ 1.29)
Neurologic disease	$-1.18*10^{-1}$	$1.19*10^{-1}$	0.322	0.89 (0.70 ~ 1.12)	$5.08*10^{-3}$	$1.15*10^{-1}$	0.965	1.01 (0.80 ~ 1.26)
Liver disease	$5.11*10^{-2}$	$2.44*10^{-1}$	0.834	1.05 (0.65 ~ 1.7)	$3.14*10^{-1}$	$2.20*10^{-1}$	0.153	1.37 (0.89 ~ 2.10)
Chronic kidney disease	$-9.36*10^{-2}$	$1.77*10^{-1}$	0.597	0.91 (0.64 ~ 1.29)	$-2.86*10^{-1}$	$1.34*10^{-1}$	0.033	0.75 (0.58 ~ 0.98)
Immunocompromised status	$2.32*10^{-1}$	$1.05*10^{-1}$	0.026	1.26 (1.03 ~ 1.55)	$1.47*10^{-1}$	$8.98*10^{-2}$	0.101	1.16 (0.97 ~ 1.38)
Bacterial infection	$-4.40*10^{-1}$	$2.54*10^{-1}$	0.083	0.64 (0.39 ~ 1.06)	$-1.17*10^{-1}$	$1.10*10^{-1}$	0.289	0.89 (0.72 ~ 1.10)
Viral infection	$-3.87*10^{-2}$	$1.41*10^{-1}$	0.783	0.96 (0.73 ~ 1.27)	$1.27*10^{-2}$	$9.45*10^{-2}$	0.893	1.01 (0.84 ~ 1.22)
Systemic glucocorticoid	$3.84*10^{-1}$	$1.15*10^{-1}$	0.001	1.47 (1.17 ~ 1.84)	$3.03*10^{-1}$	$1.09*10^{-1}$	0.005	1.35 (1.09 ~ 1.68)
Neuromuscular blocker	$5.75*10^{-1}$	$1.17*10^{-1}$	<0.001	1.78 (1.41 ~ 2.23)	$2.86*10^{-1}$	$1.02*10^{-1}$	0.005	1.33 (1.09 ~ 1.63)
Mean arterial pressure (MAP)	$4.64*10^{-9}$	$5.08*10^{-10}$	<0.001	1.00 (1.00 ~ 1.00)	$-2.91*10^{-4}$	$1.63*10^{-3}$	0.859	1.00 (1.00 ~ 1.00)
Heart rate (HR)	$7.66*10^{-3}$	$1.26*10^{-3}$	<0.001	1.01 (1.01 ~ 1.01)	$7.04*10^{-3}$	$1.16*10^{-3}$	<0.001	1.01 (1.00 ~ 1.01)
Respiratory rate (RR)	$3.98*10^{-3}$	$1.88*10^{-3}$	0.043	1.00 (1.00 ~ 1.01)	$1.13*10^{-3}$	$2.59*10^{-3}$	0.662	1.00 (1.00 ~ 1.01)
PEEP	$3.61*10^{-2}$	$8.47*10^{-3}$	<0.001	1.04 (1.02 ~ 1.05)	$3.30*10^{-2}$	$9.97*10^{-3}$	0.001	1.03 (1.01 ~ 1.05)
Driving pressure (ΔP)	$4.03*10^{-3}$	$5.14*10^{-3}$	0.445	1.00 (0.99 ~ 1.01)	$9.74*10^{-4}$	$1.79*10^{-3}$	0.587	1.00 (1.00 ~ 1.00)
Fluid balance (input − output)	$1.44*10^{-11}$	$1.04*10^{-11}$	0.237	1.00 (1.00 ~ 1.00)	$4.79*10^{-5}$	$1.81*10^{-5}$	0.009	1.00 (1.00 ~ 1.00)
PaO_2/FiO_2 ratio	$-1.21*10^{-3}$	$2.99*10^{-4}$	<0.001	1.00 (1.00 ~ 1.00)	$-1.15*10^{-3}$	$2.63*10^{-4}$	<0.001	1.00 (1.00 ~ 1.00)
PaCO2	$3.39*10^{-3}$	$2.95*10^{-3}$	0.252	1.00 (1.00 ~ 1.01)	$6.49*10^{-3}$	$2.79*10^{-3}$	0.022	1.01 (1.00 ~ 1.01)

	No fungus				Fungus			
**Variables**	***β***	***Se***	***P***	***HR* (95% *CI*)**	***β***	***Se***	***P***	***HR* (95% *CI*)**
pH	$-8.39*10^{-1}$	$3.76*10^{-1}$	0.029	0.43 (0.21 ~ 0.90)	$-3.37*10^{-1}$	$3.53*10^{-1}$	0.345	0.71 (0.36 ~ 1.43)
Lactate	$1.20*10^{-1}$	$1.98*10^{-2}$	<0.001	1.13 (1.09 ~ 1.17)	$1.07*10^{-1}$	$1.56*10^{-2}$	<0.001	1.11 (1.08 ~ 1.15)
C-reactive protein (CRP)	$1.05*10^{-3}$	$3.96*10^{-4}$	0.012	1.00 (1.00 ~ 1.00)	$9.00*10^{-4}$	$2.73*10^{-4}$	0.001	1.00 (1.00 ~ 1.00)
Interleukin-6 (IL-6)	$1.26*10^{-4}$	$2.79*10^{-5}$	<0.001	1.00 (1.00 ~ 1.00)	$1.26*10^{-4}$	$2.88*10^{-5}$	<0.001	1.00 (1.00 ~ 1.00)
Procalcitonin (PCT)	$-8.27*10^{-4}$	$2.20*10^{-3}$	0.707	1.00 (0.99 ~ 1.00)	$-3.20*10^{-3}$	$2.25*10^{-3}$	0.155	1.00 (0.99 ~ 1.00)
B-type natriuretic peptide (BNP)	$7.72*10^{-6}$	$3.99*10^{-6}$	0.054	1.00 (1.00 ~ 1.00)	$1.04*10^{-5}$	$3.81*10^{-6}$	0.006	1.00 (1.00 ~ 1.00)
Albumin	$-2.75*10^{-3}$	$5.92*10^{-3}$	0.642	1.00 (0.99 ~ 1.01)	$-1.36*10^{-2}$	$4.81*10^{-3}$	0.005	0.99 (0.98 ~ 1.00)
Absolute neutrophil count (ANC)	$1.89*10^{-2}$	$5.62*10^{-3}$	0.001	1.02 (1.01 ~ 1.03)	$1.85*10^{-2}$	$5.03*10^{-3}$	<0.001	1.02 (1.01 ~ 1.03)
Absolute lymphocyte count (ALC)	$-7.31*10^{-2}$	$5.35*10^{-2}$	0.172	0.93 (0.84 ~ 1.03)	$-7.18*10^{-2}$	$4.24*10^{-2}$	0.091	0.93 (0.86 ~ 1.01)
Hemoglobin (Hb)	$-2.11*10^{-3}$	$1.81*10^{-3}$	0.245	1.00 (0.99 ~ 1.00)	$3.44*10^{-4}$	$1.50*10^{-3}$	0.819	1.00 (1.00 ~ 1.00)
D-dimer	$1.23*10^{-3}$	$3.08*10^{-3}$	0.701	1.00 (1.00 ~ 1.01)	$-9.13*10^{-5}$	$1.58*10^{-4}$	0.568	1.00 (1.00 ~ 1.00)
Creatinine (Cr)	$1.13*10^{-3}$	$3.44*10^{-4}$	0.001	1.00 (1.00 ~ 1.00)	$4.78*10^{-4}$	$3.16*10^{-4}$	0.131	1.00 (1.00 ~ 1.00)
Platelet count (PLT)	$-1.52*10^{-3}$	$4.05*10^{-4}$	<0.001	1.00 (1.00 ~ 1.00)	$-1.10*10^{-3}$	$3.50*10^{-4}$	0.002	1.00 (1.00 ~ 1.00)
Time (day)	$1.93*10^{-2}$	$4.18*10^{-3}$	<0.001	1.02 (1.01 ~ 1.03)	$2.15*10^{-2}$	$3.66*10^{-3}$	<0.001	1.02 (1.01 ~ 1.03)

For covariate definitions and abbreviations, see the note to Tables S18–S37.

Table S27. Subgroup analysis stratified by fungal infection status (fungal vs. non-fungal), Model 5 (4×ΔP+RR)

	No fungus				Fungus			
**Variables**	***β***	***Se***	***P***	***HR* (95% *CI*)**	***β***	***Se***	***P***	***HR* (95% *CI*)**
Age	$2.60*10^{-2}$	$3.60*10^{-3}$	<0.001	1.03 (1.02 ~ 1.03)	$1.36*10^{-2}$	$3.22*10^{-3}$	<0.001	1.01 (1.01 ~ 1.02)
Non-pulmonary SOFA score	$7.18*10^{-2}$	$1.91*10^{-2}$	<0.001	1.07 (1.03 ~ 1.12)	$9.13*10^{-2}$	$1.54*10^{-2}$	<0.001	1.10 (1.06 ~ 1.13)
Septic shock	$-3.09*10^{-2}$	$1.21*10^{-1}$	0.798	0.97 (0.76 ~ 1.23)	$-4.87*10^{-2}$	$1.05*10^{-1}$	0.644	0.95 (0.77 ~ 1.17)
Chronic lung disease	$1.07*10^{-3}$	$1.13*10^{-1}$	0.992	1.00 (0.8 ~ 1.25)	$9.99*10^{-2}$	$9.59*10^{-2}$	0.298	1.11 (0.92 ~ 1.33)
Cardiac disease	$7.73*10^{-2}$	$1.20*10^{-1}$	0.518	1.08 (0.85 ~ 1.37)	$2.17*10^{-2}$	$1.19*10^{-1}$	0.856	1.02 (0.81 ~ 1.29)
Neurologic disease	$-1.18*10^{-1}$	$1.19*10^{-1}$	0.324	0.89 (0.70 ~ 1.12)	$3.80*10^{-3}$	$1.15*10^{-1}$	0.974	1.00 (0.80 ~ 1.26)
Liver disease	$5.23*10^{-2}$	$2.47*10^{-1}$	0.832	1.05 (0.65 ~ 1.71)	$3.14*10^{-1}$	$2.20*10^{-1}$	0.154	1.37 (0.89 ~ 2.10)
Chronic kidney disease	$-9.59*10^{-2}$	$1.77*10^{-1}$	0.589	0.91 (0.64 ~ 1.29)	$-2.87*10^{-1}$	$1.34*10^{-1}$	0.033	0.75 (0.58 ~ 0.98)
Immunocompromised status	$2.30*10^{-1}$	$1.05*10^{-1}$	0.028	1.26 (1.03 ~ 1.55)	$1.47*10^{-1}$	$8.98*10^{-2}$	0.102	1.16 (0.97 ~ 1.38)
Bacterial infection	$-4.46*10^{-1}$	$2.55*10^{-1}$	0.08	0.64 (0.39 ~ 1.05)	$-1.17*10^{-1}$	$1.10*10^{-1}$	0.289	0.89 (0.72 ~ 1.10)
Viral infection	$-4.09*10^{-2}$	$1.41*10^{-1}$	0.772	0.96 (0.73 ~ 1.27)	$1.35*10^{-2}$	$9.44*10^{-2}$	0.886	1.01 (0.84 ~ 1.22)
Systemic glucocorticoid	$3.81*10^{-1}$	$1.15*10^{-1}$	0.001	1.46 (1.17 ~ 1.83)	$3.02*10^{-1}$	$1.09*10^{-1}$	0.005	1.35 (1.09 ~ 1.67)
Neuromuscular blocker	$5.77*10^{-1}$	$1.17*10^{-1}$	<0.001	1.78 (1.42 ~ 2.24)	$2.86*10^{-1}$	$1.01*10^{-1}$	0.005	1.33 (1.09 ~ 1.62)
Mean arterial pressure (MAP)	$4.65*10^{-9}$	$5.03*10^{-10}$	<0.001	1.00 (1.00 ~ 1.00)	$-2.79*10^{-4}$	$1.57*10^{-3}$	0.859	1.00 (1.00 ~ 1.00)
Heart rate (HR)	$7.65*10^{-3}$	$1.27*10^{-3}$	<0.001	1.01 (1.01 ~ 1.01)	$7.06*10^{-3}$	$1.15*10^{-3}$	<0.001	1.01 (1.00 ~ 1.01)
PEEP	$3.57*10^{-2}$	$8.54*10^{-3}$	<0.001	1.04 (1.02 ~ 1.05)	$3.30*10^{-2}$	$9.97*10^{-3}$	0.001	1.03 (1.01 ~ 1.05)
[(4×ΔP)+RR]	$2.45*10^{-3}$	$2.83*10^{-3}$	0.424	1.00 (1.00 ~ 1.01)	$4.75*10^{-4}$	$4.01*10^{-4}$	0.253	1.00 (1.00 ~ 1.00)
Fluid balance (input − output)	$1.44*10^{-11}$	$1.05*10^{-11}$	0.240	1.00 (1.00 ~ 1.00)	$4.77*10^{-5}$	$1.82*10^{-5}$	0.009	1.00 (1.00 ~ 1.00)
PaO_2/FiO_2 ratio	$-1.20*10^{-3}$	$3.06*10^{-4}$	<0.001	1.00 (1.00 ~ 1.00)	$-1.15*10^{-3}$	$2.63*10^{-4}$	<0.001	1.00 (1.00 ~ 1.00)
PaCO2	$3.11*10^{-3}$	$2.91*10^{-3}$	0.287	1.00 (1.00 ~ 1.01)	$6.44*10^{-3}$	$2.79*10^{-3}$	0.023	1.01 (1.00 ~ 1.01)
pH	$-8.59*10^{-1}$	$3.75*10^{-1}$	0.025	0.42 (0.20 ~ 0.88)	$-3.38*10^{-1}$	$3.56*10^{-1}$	0.348	0.71 (0.35 ~ 1.43)

	No fungus				Fungus			
**Variables**	***β***	***Se***	***P***	***HR* (95% *CI*)**	***β***	***Se***	***P***	***HR* (95% *CI*)**
Lactate	$1.20*10^{-1}$	$1.97*10^{-2}$	<0.001	1.13 (1.08 ~ 1.17)	$1.07*10^{-1}$	$1.56*10^{-2}$	<0.001	1.11 (1.08 ~ 1.15)
C-reactive protein (CRP)	$1.05*10^{-3}$	$3.96*10^{-4}$	0.012	1.00 (1.00 ~ 1.00)	$9.05*10^{-4}$	$2.71*10^{-4}$	0.001	1.00 (1.00 ~ 1.00)
Interleukin-6 (IL-6)	$1.26*10^{-4}$	$2.78*10^{-5}$	<0.001	1.00 (1.00 ~ 1.00)	$1.26*10^{-4}$	$2.87*10^{-5}$	<0.001	1.00 (1.00 ~ 1.00)
Procalcitonin (PCT)	$-8.22*10^{-4}$	$2.20*10^{-3}$	0.709	1.00 (0.99 ~ 1.00)	$-3.22*10^{-3}$	$2.25*10^{-3}$	0.153	1.00 (0.99 ~ 1.00)
B-type natriuretic peptide (BNP)	$7.74*10^{-6}$	$3.98*10^{-6}$	0.054	1.00 (1.00 ~ 1.00)	$1.04*10^{-5}$	$3.81*10^{-6}$	0.006	1.00 (1.00 ~ 1.00)
Albumin	$-2.70*10^{-3}$	$5.94*10^{-3}$	0.649	1.00 (0.99 ~ 1.01)	$-1.35*10^{-2}$	$4.80*10^{-3}$	0.005	0.99 (0.98 ~ 1.00)
Absolute neutrophil count (ANC)	$1.88*10^{-2}$	$5.67*10^{-3}$	0.001	1.02 (1.01 ~ 1.03)	$1.85*10^{-2}$	$5.04*10^{-3}$	0.001	1.02 (1.01 ~ 1.03)
Absolute lymphocyte count (ALC)	$-7.39*10^{-2}$	$5.37*10^{-2}$	0.169	0.93 (0.84 ~ 1.03)	$-7.19*10^{-2}$	$4.24*10^{-2}$	0.091	0.93 (0.86 ~ 1.01)
Hemoglobin (Hb)	$-2.14*10^{-3}$	$1.80*10^{-3}$	0.235	1.00 (0.99 ~ 1.00)	$3.17*10^{-4}$	$1.50*10^{-3}$	0.833	1.00 (1.00 ~ 1.00)
D-dimer	$1.19*10^{-3}$	$3.00*10^{-3}$	0.702	1.00 (1.00 ~ 1.01)	$-9.22*10^{-5}$	$1.59*10^{-4}$	0.564	1.00 (1.00 ~ 1.00)
Creatinine (Cr)	$1.12*10^{-3}$	$3.44*10^{-4}$	0.001	1.00 (1.00 ~ 1.00)	$4.80*10^{-4}$	$3.17*10^{-4}$	0.131	1.00 (1.00 ~ 1.00)
Platelet count (PLT)	$-1.52*10^{-3}$	$4.03*10^{-4}$	<0.001	1.00 (1.00 ~ 1.00)	$-1.10*10^{-3}$	$3.50*10^{-4}$	0.002	1.00 (1.00 ~ 1.00)
Time (day)	$1.98*10^{-2}$	$4.29*10^{-3}$	<0.001	1.02 (1.01 ~ 1.03)	$2.16*10^{-2}$	$3.62*10^{-3}$	<0.001	1.02 (1.01 ~ 1.03)

For covariate definitions and abbreviations, see the note to Tables S18–S37.

Table S28. Subgroup analysis stratified by hypoxemia severity (mild vs. moderate vs. severe), Model 1 (VT/kg)

	Mild hypoxemia				Moderate hypoxemia				Severe hypoxemia			
**Variables**	***β***	***Se***	***P***	***HR* (95% *CI*)**	***β***	***Se***	***P***	***HR* (95% *CI*)**	***β***	***Se***	***P***	***HR* (95% *CI*)**
Age	$2.77*10^{-2}$	$4.36*10^{-3}$	<0.001	1.03 (1.02 ~ 1.04)	$2.13*10^{-2}$	$3.33*10^{-3}$	<0.001	1.02 (1.01 ~ 1.03)	$9.03*10^{-3}$	$4.61*10^{-3}$	0.050	1.01 (1.00 ~ 1.02)
Non-pulmonary SOFA score	$8.74*10^{-2}$	$2.20*10^{-2}$	<0.001	1.09 (1.05 ~ 1.14)	$9.47*10^{-2}$	$1.69*10^{-2}$	<0.001	1.10 (1.06 ~ 1.14)	$4.96*10^{-2}$	$2.19*10^{-2}$	0.024	1.05 (1.01 ~ 1.10)
Septic shock	$-1.34*10^{-1}$	$1.39*10^{-1}$	0.336	0.87 (0.67 ~ 1.15)	$-6.38*10^{-2}$	$1.15*10^{-1}$	0.577	0.94 (0.75 ~ 1.17)	$1.88*10^{-1}$	$1.60*10^{-1}$	0.242	1.21 (0.88 ~ 1.65)
Chronic lung disease	$-1.52*10^{-1}$	$1.35*10^{-1}$	0.258	0.86 (0.66 ~ 1.12)	$7.67*10^{-2}$	$1.01*10^{-1}$	0.446	1.08 (0.89 ~ 1.31)	$2.97*10^{-1}$	$1.39*10^{-1}$	0.032	1.35 (1.03 ~ 1.77)
Cardiac disease	$2.40*10^{-1}$	$1.39*10^{-1}$	0.084	1.27 (0.97 ~ 1.67)	$-9.70*10^{-2}$	$1.25*10^{-1}$	0.439	0.91 (0.71 ~ 1.16)	$1.27*10^{-1}$	$1.71*10^{-1}$	0.459	1.14 (0.81 ~ 1.59)
Neurologic disease	$-1.16*10^{-2}$	$1.41*10^{-1}$	0.934	0.99 (0.75 ~ 1.30)	$-6.14*10^{-2}$	$1.18*10^{-1}$	0.603	0.94 (0.75 ~ 1.19)	$-4.11*10^{-2}$	$1.72*10^{-1}$	0.811	0.96 (0.69 ~ 1.34)
Liver disease	$3.46*10^{-1}$	$2.25*10^{-1}$	0.125	1.41 (0.91 ~ 2.20)	$1.80*10^{-1}$	$2.41*10^{-1}$	0.454	1.20 (0.75 ~ 1.92)	$3.45*10^{-1}$	$3.62*10^{-1}$	0.341	1.41 (0.69 ~ 2.87)
Chronic kidney disease	$-3.41*10^{-1}$	$1.85*10^{-1}$	0.065	0.71 (0.49 ~ 1.02)	$-1.54*10^{-1}$	$1.46*10^{-1}$	0.292	0.86 (0.64 ~ 1.14)	$-8.67*10^{-2}$	$2.25*10^{-1}$	0.700	0.92 (0.59 ~ 1.43)
Immunocompromised status	$2.43*10^{-1}$	$1.18*10^{-1}$	0.040	1.27 (1.01 ~ 1.61)	$1.16*10^{-1}$	$9.50*10^{-2}$	0.223	1.12 (0.93 ~ 1.35)	$2.74*10^{-1}$	$1.34*10^{-1}$	0.041	1.31 (1.01 ~ 1.71)
Bacterial infection	$-1.95*10^{-1}$	$1.73*10^{-1}$	0.260	0.82 (0.59 ~ 1.15)	$-1.65*10^{-1}$	$1.35*10^{-1}$	0.223	0.85 (0.65 ~ 1.11)	$2.70*10^{-2}$	$2.20*10^{-1}$	0.903	1.03 (0.67 ~ 1.58)
Viral infection	$2.27*10^{-1}$	$1.37*10^{-1}$	0.098	1.25 (0.96 ~ 1.64)	$5.00*10^{-2}$	$1.06*10^{-1}$	0.638	1.05 (0.85 ~ 1.30)	$-3.20*10^{-1}$	$1.42*10^{-1}$	0.024	0.73 (0.55 ~ 0.96)
Fungal infection	$3.77*10^{-1}$	$1.23*10^{-1}$	0.002	1.46 (1.15 ~ 1.86)	$5.65*10^{-2}$	$9.60*10^{-2}$	0.556	1.06 (0.88 ~ 1.28)	$2.06*10^{-1}$	$1.41*10^{-1}$	0.145	1.23 (0.93 ~ 1.62)
Systemic glucocorticoid	$2.42*10^{-1}$	$1.28*10^{-1}$	0.059	1.27 (0.99 ~ 1.64)	$4.36*10^{-1}$	$1.06*10^{-1}$	<0.001	1.55 (1.26 ~ 1.90)	$1.59*10^{-1}$	$1.97*10^{-1}$	0.418	1.17 (0.80 ~ 1.73)
Neuromuscular blocker	$6.27*10^{-1}$	$1.52*10^{-1}$	<0.001	1.87 (1.39 ~ 2.52)	$3.01*10^{-1}$	$1.05*10^{-1}$	0.004	1.35 (1.10 ~ 1.66)	$2.59*10^{-1}$	$1.52*10^{-1}$	0.088	1.30 (0.96 ~ 1.74)
Mean arterial pressure (MAP)	$-2.53*10^{-3}$	$2.10*10^{-3}$	0.228	1.00 (0.99 ~ 1.00)	$4.70*10^{-9}$	$4.70*10^{-10}$	<0.001	1.00 (1.00 ~ 1.00)	$1.33*10^{-4}$	$2.09*10^{-4}$	0.526	1.00 (1.00 ~ 1.00)
Heart rate (HR)	$6.85*10^{-3}$	$1.23*10^{-3}$	<0.001	1.01 (1.00 ~ 1.01)	$8.96*10^{-3}$	$1.26*10^{-3}$	<0.001	1.01 (1.01 ~ 1.01)	$6.92*10^{-3}$	$1.85*10^{-3}$	<0.001	1.01 (1.00 ~ 1.01)
Respiratory rate (RR)	$1.39*10^{-3}$	$4.08*10^{-3}$	0.734	1.00 (0.99 ~ 1.01)	$5.03*10^{-3}$	$2.46*10^{-3}$	0.042	1.01 (1.00 ~ 1.01)	$8.43*10^{-4}$	$2.37*10^{-3}$	0.723	1.00 (1.00 ~ 1.01)
PEEP	$4.70*10^{-2}$	$1.97*10^{-2}$	0.046	1.05 (1.01 ~ 1.09)	$4.46*10^{-2}$	$1.56*10^{-2}$	0.014	1.05 (1.01 ~ 1.08)	$1.79*10^{-2}$	$1.25*10^{-2}$	0.152	1.02 (0.99 ~ 1.04)
Tidal volume per body weight (VT/kg)	$-3.09*10^{-3}$	$1.58*10^{-3}$	0.068	1.00 (0.99 ~ 1.00)	$-1.99*10^{-3}$	$3.00*10^{-3}$	0.521	1.00 (0.99 ~ 1.00)	$-3.58*10^{-3}$	$2.09*10^{-2}$	0.864	1.00 (0.96 ~ 1.04)
Fluid balance (input − output)	$4.51*10^{-5}$	$4.99*10^{-5}$	0.405	1.00 (1.00 ~ 1.00)	$1.94*10^{-5}$	$3.16*10^{-5}$	0.565	1.00 (1.00 ~ 1.00)	$1.84*10^{-5}$	$2.62*10^{-5}$	0.49	1.00 (1.00 ~ 1.00)
PaCO2	$6.50*10^{-4}$	$4.55*10^{-3}$	0.887	1.00 (0.99 ~ 1.01)	$1.13*10^{-2}$	$2.74*10^{-3}$	<0.001	1.01 (1.01 ~ 1.02)	$5.36*10^{-3}$	$3.28*10^{-3}$	0.103	1.01 (1.00 ~ 1.01)

Variables	Mild hypoxemia β	Mild hypoxemia Se	Mild hypoxemia P	Mild hypoxemia HR (95% CI)	Moderate hypoxemia β	Moderate hypoxemia Se	Moderate hypoxemia P	Moderate hypoxemia HR (95% CI)	Severe hypoxemia β	Severe hypoxemia Se	Severe hypoxemia P	Severe hypoxemia HR (95% CI)
pH	-1.32	$5.49*10^{-1}$	0.027	0.27 (0.09 ~ 0.78)	$3.37*10^{-1}$	$3.96*10^{-1}$	0.401	1.40 (0.64 ~ 3.05)	$-6.90*10^{-1}$	$4.86*10^{-1}$	0.163	0.50 (0.19 ~ 1.30)
Lactate	$1.25*10^{-1}$	$1.88*10^{-2}$	<0.001	1.13 (1.09 ~ 1.18)	$1.10*10^{-1}$	$2.38*10^{-2}$	0.002	1.12 (1.07 ~ 1.17)	$1.07*10^{-1}$	$2.29*10^{-2}$	<0.001	1.11 (1.06 ~ 1.16)
C-reactive protein (CRP)	$1.14*10^{-3}$	$5.37*10^{-4}$	0.050	1.00 (1.00 ~ 1.00)	$1.13*10^{-3}$	$3.33*10^{-4}$	0.001	1.00 (1.00 ~ 1.00)	$4.90*10^{-4}$	$3.62*10^{-4}$	0.176	1.00 (1.00 ~ 1.00)
Interleukin-6 (IL-6)	$1.22*10^{-4}$	$4.38*10^{-5}$	0.010	1.00 (1.00 ~ 1.00)	$1.26*10^{-4}$	$2.86*10^{-5}$	<0.001	1.00 (1.00 ~ 1.00)	$1.59*10^{-4}$	$4.47*10^{-5}$	0.002	1.00 (1.00 ~ 1.00)
Procalcitonin (PCT)	$-1.66*10^{-3}$	$3.20*10^{-3}$	0.605	1.00 (0.99 ~ 1.00)	$-1.68*10^{-3}$	$2.09*10^{-3}$	0.422	1.00 (0.99 ~ 1.00)	$-2.00*10^{-3}$	$3.02*10^{-3}$	0.508	1.00 (0.99 ~ 1.00)
B-type natriuretic peptide (BNP)	$1.04*10^{-5}$	$5.09*10^{-6}$	0.043	1.00 (1.00 ~ 1.00)	$1.03*10^{-5}$	$4.11*10^{-6}$	0.014	1.00 (1.00 ~ 1.00)	$8.36*10^{-6}$	$4.84*10^{-6}$	0.085	1.00 (1.00 ~ 1.00)
Albumin	$-5.25*10^{-3}$	$6.49*10^{-3}$	0.419	0.99 (0.98 ~ 1.01)	$-6.71*10^{-3}$	$5.34*10^{-3}$	0.210	0.99 (0.98 ~ 1.00)	$-1.56*10^{-2}$	$7.88*10^{-3}$	0.052	0.98 (0.97 ~ 1.00)
Absolute neutrophil count (ANC)	$2.02*10^{-2}$	$6.09*10^{-3}$	0.001	1.02 (1.01 ~ 1.03)	$2.35*10^{-2}$	$5.14*10^{-3}$	<0.001	1.02 (1.01 ~ 1.03)	$2.72*10^{-3}$	$7.33*10^{-3}$	0.712	1.00 (0.99 ~ 1.02)
Absolute lymphocyte count (ALC)	$-7.53*10^{-2}$	$7.41*10^{-2}$	0.315	0.93 (0.8 ~ 1.07)	$-7.81*10^{-2}$	$5.00*10^{-2}$	0.119	0.92 (0.84 ~ 1.02)	$-3.35*10^{-2}$	$6.67*10^{-2}$	0.618	0.97 (0.85 ~ 1.10)
Hemoglobin (Hb)	$-8.22*10^{-4}$	$2.19*10^{-3}$	0.707	1.00 (0.99 ~ 1.00)	$-1.57*10^{-3}$	$1.56*10^{-3}$	0.315	1.00 (1.00 ~ 1.00)	$1.60*10^{-3}$	$2.24*10^{-3}$	0.477	1.00 (1.00 ~ 1.01)
D-dimer	$3.36*10^{-3}$	$4.67*10^{-3}$	0.481	1.00 (0.99 ~ 1.01)	$-8.24*10^{-5}$	$2.05*10^{-4}$	0.696	1.00 (1.00 ~ 1.00)	$3.73*10^{-3}$	$4.60*10^{-3}$	0.427	1.00 (0.99 ~ 1.01)
Creatinine (Cr)	$5.85*10^{-4}$	$4.25*10^{-4}$	0.168	1.00 (1.00 ~ 1.00)	$8.49*10^{-4}$	$2.93*10^{-4}$	0.004	1.00 (1.00 ~ 1.00)	$5.36*10^{-4}$	$5.25*10^{-4}$	0.313	1.00 (1.00 ~ 1.00)
Platelet count (PLT)	$-1.40*10^{-3}$	$5.10*10^{-4}$	0.006	1.00 (1.00 ~ 1.00)	$-1.50*10^{-3}$	$3.87*10^{-4}$	<0.001	1.00 (1.00 ~ 1.00)	$-6.74*10^{-4}$	$5.55*10^{-4}$	0.229	1.00 (1.00 ~ 1.00)
Time (day)	$1.63*10^{-2}$	$5.20*10^{-3}$	0.002	1.02 (1.01 ~ 1.03)	$1.49*10^{-2}$	$3.99*10^{-3}$	<0.001	1.02 (1.01 ~ 1.02)	$2.51*10^{-2}$	$7.89*10^{-3}$	0.004	1.03 (1.01 ~ 1.04)

For covariate definitions and abbreviations, see the note to Tables S18–S37.

Table S29. Subgroup analysis stratified by hypoxemia severity (mild vs. moderate vs. severe), Model 2 (VE)

	Mild hypoxemia				Moderate hypoxemia				Severe hypoxemia			
**Variables**	***β***	***Se***	***P***	***HR* (95% *CI*)**	***β***	***Se***	***P***	***HR* (95% *CI*)**	***β***	***Se***	***P***	***HR* (95% *CI*)**
Age	$2.77*10^{-2}$	$4.36*10^{-3}$	<0.001	1.03 (1.02 ~ 1.04)	$2.13*10^{-2}$	$3.33*10^{-3}$	<0.001	1.02 (1.01 ~ 1.03)	$9.11*10^{-3}$	$4.60*10^{-3}$	0.048	1.01 (1.00 ~ 1.02)
Non-pulmonary SOFA score	$8.70*10^{-2}$	$2.21*10^{-2}$	<0.001	1.09 (1.04 ~ 1.14)	$9.45*10^{-2}$	$1.70*10^{-2}$	<0.001	1.10 (1.06 ~ 1.14)	$4.96*10^{-2}$	$2.19*10^{-2}$	0.024	1.05 (1.01 ~ 1.10)
Septic shock	$-1.34*10^{-1}$	$1.39*10^{-1}$	0.335	0.87 (0.67 ~ 1.15)	$-6.42*10^{-2}$	$1.15*10^{-1}$	0.575	0.94 (0.75 ~ 1.17)	$1.89*10^{-1}$	$1.60*10^{-1}$	0.24	1.21 (0.88 ~ 1.65)
Chronic lung disease	$-1.50*10^{-1}$	$1.35*10^{-1}$	0.266	0.86 (0.66 ~ 1.12)	$7.71*10^{-2}$	$1.01*10^{-1}$	0.443	1.08 (0.89 ~ 1.32)	$2.98*10^{-1}$	$1.38*10^{-1}$	0.031	1.35 (1.03 ~ 1.77)
Cardiac disease	$2.38*10^{-1}$	$1.39*10^{-1}$	0.086	1.27 (0.97 ~ 1.67)	$-9.57*10^{-2}$	$1.26*10^{-1}$	0.446	0.91 (0.71 ~ 1.16)	$1.26*10^{-1}$	$1.71*10^{-1}$	0.463	1.13 (0.81 ~ 1.59)
Neurologic disease	$-1.18*10^{-2}$	$1.40*10^{-1}$	0.933	0.99 (0.75 ~ 1.30)	$-6.21*10^{-2}$	$1.18*10^{-1}$	0.600	0.94 (0.75 ~ 1.19)	$-3.82*10^{-2}$	$1.71*10^{-1}$	0.823	0.96 (0.69 ~ 1.35)
Liver disease	$3.43*10^{-1}$	$2.26*10^{-1}$	0.128	1.41 (0.91 ~ 2.19)	$1.82*10^{-1}$	$2.41*10^{-1}$	0.449	1.20 (0.75 ~ 1.93)	$3.48*10^{-1}$	$3.61*10^{-1}$	0.335	1.42 (0.70 ~ 2.87)
Chronic kidney disease	$-3.43*10^{-1}$	$1.84*10^{-1}$	0.063	0.71 (0.49 ~ 1.02)	$-1.52*10^{-1}$	$1.46*10^{-1}$	0.300	0.86 (0.65 ~ 1.14)	$-8.54*10^{-2}$	$2.25*10^{-1}$	0.705	0.92 (0.59 ~ 1.43)
Immunocompromised status	$2.41*10^{-1}$	$1.18*10^{-1}$	0.041	1.27 (1.01 ~ 1.60)	$1.18*10^{-1}$	$9.50*10^{-2}$	0.216	1.12 (0.93 ~ 1.35)	$2.74*10^{-1}$	$1.34*10^{-1}$	0.041	1.32 (1.01 ~ 1.71)
Bacterial infection	$-1.91*10^{-1}$	$1.73*10^{-1}$	0.271	0.83 (0.59 ~ 1.16)	$-1.63*10^{-1}$	$1.35*10^{-1}$	0.228	0.85 (0.65 ~ 1.11)	$2.97*10^{-2}$	$2.21*10^{-1}$	0.893	1.03 (0.67 ~ 1.59)
Viral infection	$2.28*10^{-1}$	$1.37*10^{-1}$	0.096	1.26 (0.96 ~ 1.64)	$5.06*10^{-2}$	$1.07*10^{-1}$	0.635	1.05 (0.85 ~ 1.30)	$-3.20*10^{-1}$	$1.42*10^{-1}$	0.024	0.73 (0.55 ~ 0.96)
Fungal infection	$3.78*10^{-1}$	$1.23*10^{-1}$	0.002	1.46 (1.15 ~ 1.86)	$5.38*10^{-2}$	$9.59*10^{-2}$	0.575	1.06 (0.87 ~ 1.27)	$2.06*10^{-1}$	$1.41*10^{-1}$	0.145	1.23 (0.93 ~ 1.62)
Systemic glucocorticoid	$2.43*10^{-1}$	$1.28*10^{-1}$	0.058	1.28 (0.99 ~ 1.64)	$4.38*10^{-1}$	$1.06*10^{-1}$	<0.001	1.55 (1.26 ~ 1.91)	$1.62*10^{-1}$	$1.97*10^{-1}$	0.412	1.18 (0.8 ~ 1.73)
Neuromuscular blocker	$6.28*10^{-1}$	$1.52*10^{-1}$	<0.001	1.87 (1.39 ~ 2.53)	$3.08*10^{-1}$	$1.05*10^{-1}$	0.003	1.36 (1.11 ~ 1.67)	$2.62*10^{-1}$	$1.52*10^{-1}$	0.084	1.30 (0.97 ~ 1.75)
Mean arterial pressure (MAP)	$-2.51*10^{-3}$	$2.09*10^{-3}$	0.231	1.00 (0.99 ~ 1.00)	$4.70*10^{-9}$	$4.70*10^{-10}$	<0.001	1.00 (1.00 ~ 1.00)	$1.33*10^{-4}$	$2.08*10^{-4}$	0.523	1.00 (1.00 ~ 1.00)
Heart rate (HR)	$6.97*10^{-3}$	$1.25*10^{-3}$	<0.001	1.01 (1.00 ~ 1.01)	$9.13*10^{-3}$	$1.27*10^{-3}$	<0.001	1.01 (1.01 ~ 1.01)	$7.04*10^{-3}$	$1.85*10^{-3}$	<0.001	1.01 (1.00 ~ 1.01)
PEEP	$4.72*10^{-2}$	$1.97*10^{-2}$	0.046	1.05 (1.01 ~ 1.09)	$4.47*10^{-2}$	$1.55*10^{-2}$	0.013	1.05 (1.01 ~ 1.08)	$1.81*10^{-2}$	$1.25*10^{-2}$	0.149	1.02 (0.99 ~ 1.04)
Minute ventilation (VE)	$-8.44*10^{-3}$	$1.37*10^{-2}$	0.543	0.99 (0.97 ~ 1.02)	$3.95*10^{-3}$	$9.50*10^{-3}$	0.678	1.00 (0.99 ~ 1.02)	$-2.01*10^{-3}$	$1.01*10^{-2}$	0.843	1.00 (0.98 ~ 1.02)
Fluid balance (input − output)	$4.57*10^{-5}$	$5.06*10^{-5}$	0.405	1.00 (1.00 ~ 1.00)	$1.95*10^{-5}$	$3.17*10^{-5}$	0.564	1.00 (1.00 ~ 1.00)	$1.83*10^{-5}$	$2.64*10^{-5}$	0.495	1.00 (1.00 ~ 1.00)
PaCO2	$6.26*10^{-4}$	$4.56*10^{-3}$	0.892	1.00 (0.99 ~ 1.01)	$1.14*10^{-2}$	$2.74*10^{-3}$	<0.001	1.01 (1.01 ~ 1.02)	$5.39*10^{-3}$	$3.26*10^{-3}$	0.099	1.01 (1.00 ~ 1.01)
pH	-1.33	$5.47*10^{-1}$	0.026	0.26 (0.09 ~ 0.77)	$3.28*10^{-1}$	$3.95*10^{-1}$	0.412	1.39 (0.64 ~ 3.01)	$-6.99*10^{-1}$	$4.86*10^{-1}$	0.159	0.50 (0.19 ~ 1.29)

	Mild hypoxemia				Moderate hypoxemia				Severe hypoxemia			
**Variables**	***β***	***Se***	***P***	***HR* (95% *CI*)**	***β***	***Se***	***P***	***HR* (95% *CI*)**	***β***	***Se***	***P***	***HR* (95% *CI*)**
Lactate	$1.25*10^{-1}$	$1.86*10^{-2}$	<0.001	1.13 (1.09 ~ 1.18)	$1.10*10^{-1}$	$2.38*10^{-2}$	0.002	1.12 (1.07 ~ 1.17)	$1.07*10^{-1}$	$2.27*10^{-2}$	<0.001	1.11 (1.06 ~ 1.16)
C-reactive protein (CRP)	$1.15*10^{-3}$	$5.43*10^{-4}$	0.052	1.00 (1.00 ~ 1.00)	$1.14*10^{-3}$	$3.31*10^{-4}$	0.001	1.00 (1.00 ~ 1.00)	$4.94*10^{-4}$	$3.63*10^{-4}$	0.173	1.00 (1.00 ~ 1.00)
Interleukin-6 (IL-6)	$1.24*10^{-4}$	$4.40*10^{-5}$	0.010	1.00 (1.00 ~ 1.00)	$1.27*10^{-4}$	$2.84*10^{-5}$	<0.001	1.00 (1.00 ~ 1.00)	$1.60*10^{-4}$	$4.44*10^{-5}$	0.001	1.00 (1.00 ~ 1.00)
Procalcitonin (PCT)	$-1.61*10^{-3}$	$3.18*10^{-3}$	0.613	1.00 (0.99 ~ 1.00)	$-1.70*10^{-3}$	$2.10*10^{-3}$	0.417	1.00 (0.99 ~ 1.00)	$-2.01*10^{-3}$	$3.02*10^{-3}$	0.507	1.00 (0.99 ~ 1.00)
B-type natriuretic peptide (BNP)	$1.03*10^{-5}$	$5.08*10^{-6}$	0.045	1.00 (1.00 ~ 1.00)	$1.04*10^{-5}$	$4.11*10^{-6}$	0.014	1.00 (1.00 ~ 1.00)	$8.32*10^{-6}$	$4.85*10^{-6}$	0.087	1.00 (1.00 ~ 1.00)
Albumin	$-5.37*10^{-3}$	$6.48*10^{-3}$	0.407	0.99 (0.98 ~ 1.01)	$-6.65*10^{-3}$	$5.36*10^{-3}$	0.216	0.99 (0.98 ~ 1.00)	$-1.57*10^{-2}$	$7.89*10^{-3}$	0.052	0.98 (0.97 ~ 1.00)
Absolute neutrophil count (ANC)	$2.02*10^{-2}$	$6.09*10^{-3}$	0.001	1.02 (1.01 ~ 1.03)	$2.35*10^{-2}$	$5.14*10^{-3}$	<0.001	1.02 (1.01 ~ 1.03)	$2.80*10^{-3}$	$7.30*10^{-3}$	0.703	1.00 (0.99 ~ 1.02)
Absolute lymphocyte count (ALC)	$-7.57*10^{-2}$	$7.47*10^{-2}$	0.316	0.93 (0.80 ~ 1.07)	$-7.92*10^{-2}$	$5.04*10^{-2}$	0.116	0.92 (0.84 ~ 1.02)	$-3.45*10^{-2}$	$6.68*10^{-2}$	0.607	0.97 (0.85 ~ 1.10)
Hemoglobin (Hb)	$-8.38*10^{-4}$	$2.20*10^{-3}$	0.703	1.00 (0.99 ~ 1.00)	$-1.61*10^{-3}$	$1.56*10^{-3}$	0.303	1.00 (1.00 ~ 1.00)	$1.61*10^{-3}$	$2.25*10^{-3}$	0.475	1.00 (1.00 ~ 1.01)
D-dimer	$3.35*10^{-3}$	$4.66*10^{-3}$	0.482	1.00 (0.99 ~ 1.01)	$-8.02*10^{-5}$	$2.06*10^{-4}$	0.704	1.00 (1.00 ~ 1.00)	$3.75*10^{-3}$	$4.62*10^{-3}$	0.426	1.00 (0.99 ~ 1.01)
Creatinine (Cr)	$5.87*10^{-4}$	$4.24*10^{-4}$	0.167	1.00 (1.00 ~ 1.00)	$8.43*10^{-4}$	$2.94*10^{-4}$	0.004	1.00 (1.00 ~ 1.00)	$5.40*10^{-4}$	$5.18*10^{-4}$	0.302	1.00 (1.00 ~ 1.00)
Platelet count (PLT)	$-1.39*10^{-3}$	$5.09*10^{-4}$	0.006	1.00 (1.00 ~ 1.00)	$-1.49*10^{-3}$	$3.86*10^{-4}$	<0.001	1.00 (1.00 ~ 1.00)	$-6.74*10^{-4}$	$5.53*10^{-4}$	0.227	1.00 (1.00 ~ 1.00)
Time (day)	$1.73*10^{-2}$	$5.13*10^{-3}$	0.001	1.02 (1.01 ~ 1.03)	$1.53*10^{-2}$	$3.95*10^{-3}$	<0.001	1.02 (1.01 ~ 1.02)	$2.53*10^{-2}$	$7.92*10^{-3}$	0.004	1.03 (1.01 ~ 1.04)

For covariate definitions and abbreviations, see the note to Tables S18–S37.

Table S30. Subgroup analysis stratified by hypoxemia severity (mild vs. moderate vs. severe), Model 3 (VR)

	Mild hypoxemia				Moderate hypoxemia				Severe hypoxemia			
Variables	*β*	*Se*	*P*	*HR* (95% *CI*)	*β*	*Se*	*P*	*HR* (95% *CI*)	*β*	*Se*	*P*	*HR* (95% *CI*)
Age	$2.77*10^{-2}$	$4.36*10^{-3}$	<0.001	1.03 (1.02 ~ 1.04)	$2.13*10^{-2}$	$3.33*10^{-3}$	<0.001	1.02 (1.01 ~ 1.03)	$9.46*10^{-3}$	$4.57*10^{-3}$	0.038	1.01 (1.00 ~ 1.02)
Non-pulmonary SOFA score	$8.72*10^{-2}$	$2.23*10^{-2}$	<0.001	1.09 (1.04 ~ 1.14)	$9.24*10^{-2}$	$1.69*10^{-2}$	<0.001	1.10 (1.06 ~ 1.13)	$4.90*10^{-2}$	$2.20*10^{-2}$	0.026	1.05 (1.01 ~ 1.10)
Septic shock	$-1.34*10^{-1}$	$1.39*10^{-1}$	0.336	0.87 (0.67 ~ 1.15)	$-6.75*10^{-2}$	$1.15*10^{-1}$	0.557	0.93 (0.75 ~ 1.17)	$1.87*10^{-1}$	$1.61*10^{-1}$	0.246	1.21 (0.88 ~ 1.65)
Chronic lung disease	$-1.47*10^{-1}$	$1.35*10^{-1}$	0.277	0.86 (0.66 ~ 1.13)	$1.11*10^{-1}$	$1.01*10^{-1}$	0.272	1.12 (0.92 ~ 1.36)	$3.03*10^{-1}$	$1.38*10^{-1}$	0.029	1.35 (1.03 ~ 1.78)
Cardiac disease	$2.39*10^{-1}$	$1.38*10^{-1}$	0.085	1.27 (0.97 ~ 1.66)	$-9.38*10^{-2}$	$1.26*10^{-1}$	0.456	0.91 (0.71 ~ 1.17)	$1.18*10^{-1}$	$1.72*10^{-1}$	0.491	1.13 (0.80 ~ 1.58)
Neurologic disease	$-1.25*10^{-2}$	$1.41*10^{-1}$	0.929	0.99 (0.75 ~ 1.30)	$-6.86*10^{-2}$	$1.18*10^{-1}$	0.560	0.93 (0.74 ~ 1.18)	$-3.14*10^{-2}$	$1.71*10^{-1}$	0.854	0.97 (0.69 ~ 1.35)
Liver disease	$3.45*10^{-1}$	$2.25*10^{-1}$	0.126	1.41 (0.91 ~ 2.20)	$1.85*10^{-1}$	$2.40*10^{-1}$	0.441	1.20 (0.75 ~ 1.93)	$3.71*10^{-1}$	$3.56*10^{-1}$	0.297	1.45 (0.72 ~ 2.91)
Chronic kidney disease	$-3.42*10^{-1}$	$1.85*10^{-1}$	0.064	0.71 (0.49 ~ 1.02)	$-1.66*10^{-1}$	$1.45*10^{-1}$	0.254	0.85 (0.64 ~ 1.13)	$-9.69*10^{-2}$	$2.24*10^{-1}$	0.666	0.91 (0.58 ~ 1.41)
Immunocompromised status	$2.43*10^{-1}$	$1.18*10^{-1}$	0.040	1.27 (1.01 ~ 1.61)	$1.14*10^{-1}$	$9.52*10^{-2}$	0.233	1.12 (0.93 ~ 1.35)	$2.77*10^{-1}$	$1.35*10^{-1}$	0.040	1.32 (1.01 ~ 1.72)
Bacterial infection	$-1.92*10^{-1}$	$1.73*10^{-1}$	0.269	0.83 (0.59 ~ 1.16)	$-1.59*10^{-1}$	$1.36*10^{-1}$	0.242	0.85 (0.65 ~ 1.11)	$1.93*10^{-2}$	$2.20*10^{-1}$	0.930	1.02 (0.66 ~ 1.57)
Viral infection	$2.28*10^{-1}$	$1.37*10^{-1}$	0.096	1.26 (0.96 ~ 1.64)	$5.43*10^{-2}$	$1.07*10^{-1}$	0.613	1.06 (0.86 ~ 1.30)	$-3.12*10^{-1}$	$1.41*10^{-1}$	0.027	0.73 (0.56 ~ 0.97)
Fungal infection	$3.79*10^{-1}$	$1.23*10^{-1}$	0.002	1.46 (1.15 ~ 1.86)	$6.19*10^{-2}$	$9.63*10^{-2}$	0.521	1.06 (0.88 ~ 1.28)	$2.06*10^{-1}$	$1.41*10^{-1}$	0.145	1.23 (0.93 ~ 1.62)
Systemic glucocorticoid	$2.45*10^{-1}$	$1.28*10^{-1}$	0.056	1.28 (0.99 ~ 1.64)	$4.31*10^{-1}$	$1.06*10^{-1}$	<0.001	1.54 (1.25 ~ 1.89)	$1.67*10^{-1}$	$1.98*10^{-1}$	0.399	1.18 (0.80 ~ 1.74)
Neuromuscular blocker	$6.30*10^{-1}$	$1.52*10^{-1}$	<0.001	1.88 (1.39 ~ 2.53)	$3.33*10^{-1}$	$1.06*10^{-1}$	0.002	1.40 (1.13 ~ 1.72)	$2.69*10^{-1}$	$1.50*10^{-1}$	0.074	1.31 (0.97 ~ 1.76)
Mean arterial pressure (MAP)	$-2.53*10^{-3}$	$2.10*10^{-3}$	0.228	1.00 (0.99 ~ 1.00)	$4.74*10^{-9}$	$4.70*10^{-10}$	<0.001	1.00 (1.00 ~ 1.00)	$1.26*10^{-4}$	$2.15*10^{-4}$	0.559	1.00 (1.00 ~ 1.00)
Heart rate (HR)	$6.92*10^{-3}$	$1.22*10^{-3}$	<0.001	1.01.00 (1.00 ~ 1.01)	$9.38*10^{-3}$	$1.33*10^{-3}$	<0.001	1.01 (1.01 ~ 1.01)	$6.93*10^{-3}$	$1.94*10^{-3}$	0.001	1.01 (1.00 ~ 1.01)
PEEP	$4.71*10^{-2}$	$1.97*10^{-2}$	0.046	1.05 (1.01 ~ 1.09)	$4.33*10^{-2}$	$1.54*10^{-2}$	0.014	1.04 (1.01 ~ 1.08)	$1.87*10^{-2}$	$1.25*10^{-2}$	0.135	1.02 (0.99 ~ 1.04)
Ventilatory ratio (VR)	$-1.79*10^{-2}$	$8.76*10^{-3}$	0.062	0.98 (0.97 ~ 1.00)	$4.86*10^{-2}$	$6.41*10^{-2}$	0.455	1.05 (0.93 ~ 1.19)	$3.74*10^{-2}$	$6.55*10^{-2}$	0.574	1.04 (0.91 ~ 1.18)
Fluid balance (input − output)	$4.41*10^{-5}$	$4.91*10^{-5}$	0.406	1.00 (1.00 ~ 1.00)	$1.58*10^{-5}$	$2.67*10^{-5}$	0.577	1.00 (1.00 ~ 1.00)	$1.37*10^{-5}$	$2.58*10^{-5}$	0.600	1.00 (1.00 ~ 1.00)
pH	-1.41	$3.83*10^{-1}$	0.001	0.24 (0.11 ~ 0.52)	$-4.91*10^{-1}$	$3.22*10^{-1}$	0.134	0.61 (0.33 ~ 1.15)	-1.06	$4.33*10^{-1}$	0.023	0.34 (0.15 ~ 0.81)
Lactate	$1.24*10^{-1}$	$1.85*10^{-2}$	<0.001	1.13 (1.09 ~ 1.17)	$1.06*10^{-1}$	$2.20*10^{-2}$	0.002	1.11 (1.06 ~ 1.16)	$1.04*10^{-1}$	$2.17*10^{-2}$	<0.001	1.11 (1.06 ~ 1.16)

	Mild hypoxemia				Moderate hypoxemia				Severe hypoxemia			
**Variables**	***β***	***Se***	***P***	***HR* (95% *CI*)**	***β***	***Se***	***P***	***HR* (95% *CI*)**	***β***	***Se***	***P***	***HR* (95% *CI*)**
C-reactive protein (CRP)	$1.14*10^{-3}$	$5.39*10^{-4}$	0.050	1.00 (1.00 ~ 1.00)	$1.14*10^{-3}$	$3.37*10^{-4}$	0.001	1.00 (1.00 ~ 1.00)	$4.97*10^{-4}$	$3.65*10^{-4}$	0.173	1.00 (1.00 ~ 1.00)
Interleukin-6 (IL-6)	$1.25*10^{-4}$	$4.42*10^{-5}$	0.010	1.00 (1.00 ~ 1.00)	$1.24*10^{-4}$	$2.80*10^{-5}$	<0.001	1.00 (1.00 ~ 1.00)	$1.58*10^{-4}$	$4.40*10^{-5}$	0.002	1.00 (1.00 ~ 1.00)
Procalcitonin (PCT)	$-1.67*10^{-3}$	$3.20*10^{-3}$	0.602	1.00 (0.99 ~ 1.00)	$-1.90*10^{-3}$	$2.11*10^{-3}$	0.366	1.00 (0.99 ~ 1.00)	$-2.08*10^{-3}$	$2.97*10^{-3}$	0.484	1.00 (0.99 ~ 1.00)
B-type natriuretic peptide (BNP)	$1.04*10^{-5}$	$5.09*10^{-6}$	0.043	1.00 (1.00 ~ 1.00)	$1.04*10^{-5}$	$4.07*10^{-6}$	0.013	1.00 (1.00 ~ 1.00)	$8.43*10^{-6}$	$4.77*10^{-6}$	0.078	1.00 (1.00 ~ 1.00)
Albumin	$-5.19*10^{-3}$	$6.48*10^{-3}$	0.423	0.99 (0.98 ~ 1.01)	$-6.19*10^{-3}$	$5.37*10^{-3}$	0.250	0.99 (0.98 ~ 1.00)	$-1.50*10^{-2}$	$7.94*10^{-3}$	0.064	0.99 (0.97 ~ 1.00)
Absolute neutrophil count (ANC)	$2.03*10^{-2}$	$6.07*10^{-3}$	0.001	1.02 (1.01 ~ 1.03)	$2.39*10^{-2}$	$5.11*10^{-3}$	<0.001	1.02 (1.01 ~ 1.03)	$3.80*10^{-3}$	$7.36*10^{-3}$	0.607	1.00 (0.99 ~ 1.02)
Absolute lymphocyte count (ALC)	$-7.62*10^{-2}$	$7.39*10^{-2}$	0.307	0.93 (0.80 ~ 1.07)	$-8.18*10^{-2}$	$4.95*10^{-2}$	0.099	0.92 (0.84 ~ 1.02)	$-3.53*10^{-2}$	$6.78*10^{-2}$	0.606	0.97 (0.85 ~ 1.10)
Hemoglobin (Hb)	$-8.00*10^{-4}$	$2.23*10^{-3}$	0.720	1.00 (0.99 ~ 1.00)	$-1.44*10^{-3}$	$1.57*10^{-3}$	0.359	1.00 (1.00 ~ 1.00)	$1.62*10^{-3}$	$2.23*10^{-3}$	0.467	1.00 (1.00 ~ 1.01)
D-dimer	$3.38*10^{-3}$	$4.68*10^{-3}$	0.480	1.00 (0.99 ~ 1.01)	$-8.88*10^{-5}$	$2.09*10^{-4}$	0.680	1.00 (1.00 ~ 1.00)	$3.81*10^{-3}$	$4.61*10^{-3}$	0.418	1.00 (0.99 ~ 1.01)
Creatinine (Cr)	$5.73*10^{-4}$	$4.23*10^{-4}$	0.176	1.00 (1.00 ~ 1.00)	$7.10*10^{-4}$	$3.01*10^{-4}$	0.019	1.00 (1.00 ~ 1.00)	$5.00*10^{-4}$	$5.32*10^{-4}$	0.353	1.00 (1.00 ~ 1.00)
Platelet count (PLT)	$-1.40*10^{-3}$	$5.11*10^{-4}$	0.007	1.00 (1.00 ~ 1.00)	$-1.48*10^{-3}$	$3.79*10^{-4}$	<0.001	1.00 (1.00 ~ 1.00)	$-6.71*10^{-4}$	$5.56*10^{-4}$	0.232	1.00 (1.00 ~ 1.00)
Time (day)	$1.73*10^{-2}$	$5.19*10^{-3}$	0.001	1.02 (1.01 ~ 1.03)	$1.61*10^{-2}$	$4.20*10^{-3}$	<0.001	1.02 (1.01 ~ 1.02)	$2.50*10^{-2}$	$7.76*10^{-3}$	0.003	1.03 (1.01 ~ 1.04)

For covariate definitions and abbreviations, see the note to Tables S18–S37.

Table S31. Subgroup analysis stratified by hypoxemia severity (mild vs. moderate vs. severe), Model 4 (ΔP)

	Mild hypoxemia				Moderate hypoxemia				Severe hypoxemia			
**Variables**	***β***	***Se***	***P***	***HR* (95% *CI*)**	***β***	***Se***	***P***	***HR* (95% *CI*)**	***β***	***Se***	***P***	***HR* (95% *CI*)**
Age	$2.76*10^{-2}$	$4.35*10^{-3}$	<0.001	1.03 (1.02 ~ 1.04)	$2.13*10^{-2}$	$3.33*10^{-3}$	<0.001	1.02 (1.01 ~ 1.03)	$9.00*10^{-3}$	$4.60*10^{-3}$	0.051	1.01 (1.00 ~ 1.02)
Non-pulmonary SOFA score	$8.73*10^{-2}$	$2.21*10^{-2}$	<0.001	1.09 (1.04 ~ 1.14)	$9.47*10^{-2}$	$1.70*10^{-2}$	<0.001	1.10 (1.06 ~ 1.14)	$4.98*10^{-2}$	$2.19*10^{-2}$	0.023	1.05 (1.01 ~ 1.10)
Septic shock	$-1.35*10^{-1}$	$1.39*10^{-1}$	0.332	0.87 (0.67 ~ 1.15)	$-6.39*10^{-2}$	$1.15*10^{-1}$	0.577	0.94 (0.75 ~ 1.17)	$1.89*10^{-1}$	$1.60*10^{-1}$	0.237	1.21 (0.88 ~ 1.65)
Chronic lung disease	$-1.52*10^{-1}$	$1.35*10^{-1}$	0.259	0.86 (0.66 ~ 1.12)	$7.65*10^{-2}$	$1.01*10^{-1}$	0.447	1.08 (0.89 ~ 1.31)	$2.94*10^{-1}$	$1.38*10^{-1}$	0.033	1.34 (1.02 ~ 1.76)
Cardiac disease	$2.39*10^{-1}$	$1.39*10^{-1}$	0.086	1.27 (0.97 ~ 1.67)	$-9.73*10^{-2}$	$1.25*10^{-1}$	0.438	0.91 (0.71 ~ 1.16)	$1.27*10^{-1}$	$1.72*10^{-1}$	0.459	1.14 (0.81 ~ 1.59)
Neurologic disease	$-1.12*10^{-2}$	$1.41*10^{-1}$	0.936	0.99 (0.75 ~ 1.30)	$-6.20*10^{-2}$	$1.18*10^{-1}$	0.600	0.94 (0.75 ~ 1.19)	$-4.17*10^{-2}$	$1.72*10^{-1}$	0.808	0.96 (0.69 ~ 1.34)
Liver disease	$3.46*10^{-1}$	$2.25*10^{-1}$	0.125	1.41 (0.91 ~ 2.20)	$1.80*10^{-1}$	$2.41*10^{-1}$	0.455	1.20 (0.75 ~ 1.92)	$3.47*10^{-1}$	$3.62*10^{-1}$	0.337	1.42 (0.70 ~ 2.87)
Chronic kidney disease	$-3.41*10^{-1}$	$1.85*10^{-1}$	0.065	0.71 (0.50 ~ 1.02)	$-1.54*10^{-1}$	$1.46*10^{-1}$	0.291	0.86 (0.64 ~ 1.14)	$-8.81*10^{-2}$	$2.25*10^{-1}$	0.696	0.92 (0.59 ~ 1.42)
Immunocompromised status	$2.42*10^{-1}$	$1.18*10^{-1}$	0.040	1.27 (1.01 ~ 1.60)	$1.16*10^{-1}$	$9.50*10^{-2}$	0.224	1.12 (0.93 ~ 1.35)	$2.72*10^{-1}$	$1.34*10^{-1}$	0.043	1.31 (1.01 ~ 1.71)
Bacterial infection	$-1.93*10^{-1}$	$1.73*10^{-1}$	0.263	0.82 (0.59 ~ 1.16)	$-1.65*10^{-1}$	$1.35*10^{-1}$	0.224	0.85 (0.65 ~ 1.11)	$2.37*10^{-2}$	$2.20*10^{-1}$	0.914	1.02 (0.67 ~ 1.58)
Viral infection	$2.27*10^{-1}$	$1.37*10^{-1}$	0.098	1.26 (0.96 ~ 1.64)	$5.08*10^{-2}$	$1.07*10^{-1}$	0.633	1.05 (0.85 ~ 1.30)	$-3.19*10^{-1}$	$1.41*10^{-1}$	0.024	0.73 (0.55 ~ 0.96)
Fungal infection	$3.76*10^{-1}$	$1.23*10^{-1}$	0.002	1.46 (1.14 ~ 1.85)	$5.59*10^{-2}$	$9.60*10^{-2}$	0.561	1.06 (0.88 ~ 1.28)	$2.08*10^{-1}$	$1.41*10^{-1}$	0.142	1.23 (0.93 ~ 1.62)
Systemic glucocorticoid	$2.42*10^{-1}$	$1.28*10^{-1}$	0.059	1.27 (0.99 ~ 1.64)	$4.35*10^{-1}$	$1.06*10^{-1}$	<0.001	1.55 (1.26 ~ 1.90)	$1.54*10^{-1}$	$1.96*10^{-1}$	0.432	1.17 (0.79 ~ 1.71)
Neuromuscular blocker	$6.27*10^{-1}$	$1.52*10^{-1}$	<0.001	1.87 (1.39 ~ 2.52)	$3.01*10^{-1}$	$1.05*10^{-1}$	0.004	1.35 (1.10 ~ 1.66)	$2.62*10^{-1}$	$1.52*10^{-1}$	0.086	1.30 (0.96 ~ 1.75)
Mean arterial pressure (MAP)	$-2.53*10^{-3}$	$2.09*10^{-3}$	0.227	1.00 (0.99 ~ 1.00)	$4.70*10^{-9}$	$4.70*10^{-10}$	<0.001	1.00 (1.00 ~ 1.00)	$1.48*10^{-4}$	$2.06*10^{-4}$	0.474	1.00 (1.00 ~ 1.00)
Heart rate (HR)	$6.85*10^{-3}$	$1.23*10^{-3}$	<0.001	1.01 (1.00 ~ 1.01)	$8.96*10^{-3}$	$1.26*10^{-3}$	<0.001	1.01 (1.01 ~ 1.01)	$6.90*10^{-3}$	$1.85*10^{-3}$	<0.001	1.01 (1.00 ~ 1.01)
Respiratory rate (RR)	$1.21*10^{-3}$	$4.20*10^{-3}$	0.775	1.00 (0.99 ~ 1.01)	$5.01*10^{-3}$	$2.47*10^{-3}$	0.043	1.01 (1.00 ~ 1.01)	$7.81*10^{-4}$	$2.42*10^{-3}$	0.749	1.00 (1.00 ~ 1.01)
PEEP	$4.68*10^{-2}$	$1.94*10^{-2}$	0.043	1.05 (1.01 ~ 1.09)	$4.45*10^{-2}$	$1.55*10^{-2}$	0.013	1.05 (1.01 ~ 1.08)	$1.75*10^{-2}$	$1.26*10^{-2}$	0.166	1.02 (0.99 ~ 1.04)
Driving pressure (ΔP)	$3.34*10^{-3}$	$6.98*10^{-3}$	0.637	1.00 (0.99 ~ 1.02)	$1.05*10^{-3}$	$1.78*10^{-3}$	0.555	1.00 (1.00 ~ 1.00)	$6.72*10^{-3}$	$8.61*10^{-3}$	0.442	1.01 (0.99 ~ 1.02)
Fluid balance (input − output)	$4.60*10^{-5}$	$5.09*10^{-5}$	0.405	1.00 (1.00 ~ 1.00)	$1.95*10^{-5}$	$3.17*10^{-5}$	0.564	1.00 (1.00 ~ 1.00)	$1.81*10^{-5}$	$2.64*10^{-5}$	0.499	1.00 (1.00 ~ 1.00)
PaCO2	$5.98*10^{-4}$	$4.55*10^{-3}$	0.896	1.00 (0.99 ~ 1.01)	$1.13*10^{-2}$	$2.75*10^{-3}$	<0.001	1.01 (1.01 ~ 1.02)	$5.19*10^{-3}$	$3.26*10^{-3}$	0.112	1.01 (1.00 ~ 1.01)

	Mild hypoxemia				Moderate hypoxemia				Severe hypoxemia			
**Variables**	***β***	***Se***	***P***	***HR* (95% *CI*)**	***β***	***Se***	***P***	***HR* (95% *CI*)**	***β***	***Se***	***P***	***HR* (95% *CI*)**
pH	-1.32	$5.45*10^{-1}$	0.026	0.27 (0.09 ~ 0.78)	$3.38*10^{-1}$	$3.95*10^{-1}$	0.398	1.40 (0.65 ~ 3.04)	$-6.95*10^{-1}$	$4.85*10^{-1}$	0.159	0.50 (0.19 ~ 1.29)
Lactate	$1.25*10^{-1}$	$1.86*10^{-2}$	<0.001	1.13 (1.09 ~ 1.18)	$1.10*10^{-1}$	$2.38*10^{-2}$	0.002	1.12 (1.07 ~ 1.17)	$1.07*10^{-1}$	$2.32*10^{-2}$	<0.001	1.11 (1.06 ~ 1.17)
C-reactive protein (CRP)	$1.14*10^{-3}$	$5.43*10^{-4}$	0.053	1.00 (1.00 ~ 1.00)	$1.13*10^{-3}$	$3.32*10^{-4}$	0.001	1.00 (1.00 ~ 1.00)	$4.98*10^{-4}$	$3.62*10^{-4}$	0.168	1.00 (1.00 ~ 1.00)
Interleukin-6 (IL-6)	$1.23*10^{-4}$	$4.38*10^{-5}$	0.009	1.00 (1.00 ~ 1.00)	$1.27*10^{-4}$	$2.86*10^{-5}$	<0.001	1.00 (1.00 ~ 1.00)	$1.59*10^{-4}$	$4.46*10^{-5}$	0.002	1.00 (1.00 ~ 1.00)
Procalcitonin (PCT)	$-1.69*10^{-3}$	$3.23*10^{-3}$	0.603	1.00 (0.99 ~ 1.00)	$-1.68*10^{-3}$	$2.09*10^{-3}$	0.421	1.00 (0.99 ~ 1.00)	$-2.01*10^{-3}$	$3.01*10^{-3}$	0.505	1.00 (0.99 ~ 1.00)
B-type natriuretic peptide (BNP)	$1.03*10^{-5}$	$5.09*10^{-6}$	0.045	1.00 (1.00 ~ 1.00)	$1.03*10^{-5}$	$4.10*10^{-6}$	0.014	1.00 (1.00 ~ 1.00)	$8.24*10^{-6}$	$4.84*10^{-6}$	0.089	1.00 (1.00 ~ 1.00)
Albumin	$-5.31*10^{-3}$	$6.50*10^{-3}$	0.414	0.99 (0.98 ~ 1.01)	$-6.62*10^{-3}$	$5.35*10^{-3}$	0.217	0.99 (0.98 ~ 1.00)	$-1.56*10^{-2}$	$7.88*10^{-3}$	0.053	0.98 (0.97 ~ 1.00)
Absolute neutrophil count (ANC)	$2.01*10^{-2}$	$6.10*10^{-3}$	0.001	1.02 (1.01 ~ 1.03)	$2.35*10^{-2}$	$5.15*10^{-3}$	<0.001	1.02 (1.01 ~ 1.03)	$2.71*10^{-3}$	$7.31*10^{-3}$	0.712	1.00 (0.99 ~ 1.02)
Absolute lymphocyte count (ALC)	$-7.49*10^{-2}$	$7.43*10^{-2}$	0.319	0.93 (0.80 ~ 1.07)	$-7.82*10^{-2}$	$5.00*10^{-2}$	0.118	0.92 (0.84 ~ 1.02)	$-3.30*10^{-2}$	$6.66*10^{-2}$	0.622	0.97 (0.85 ~ 1.10)
Hemoglobin (Hb)	$-8.21*10^{-4}$	$2.21*10^{-3}$	0.710	1.00 (0.99 ~ 1.00)	$-1.59*10^{-3}$	$1.56*10^{-3}$	0.310	1.00 (1.00 ~ 1.00)	$1.59*10^{-3}$	$2.26*10^{-3}$	0.484	1.00 (1.00 ~ 1.01)
D-dimer	$3.37*10^{-3}$	$4.67*10^{-3}$	0.480	1.00 (0.99 ~ 1.01)	$-8.18*10^{-5}$	$2.06*10^{-4}$	0.698	1.00 (1.00 ~ 1.00)	$3.85*10^{-3}$	$4.66*10^{-3}$	0.419	1.00 (0.99 ~ 1.01)
Creatinine (Cr)	$5.88*10^{-4}$	$4.23*10^{-4}$	0.165	1.00 (1.00 ~ 1.00)	$8.51*10^{-4}$	$2.93*10^{-4}$	0.004	1.00 (1.00 ~ 1.00)	$5.43*10^{-4}$	$5.22*10^{-4}$	0.303	1.00 (1.00 ~ 1.00)
Platelet count (PLT)	$-1.40*10^{-3}$	$5.09*10^{-4}$	0.006	1.00 (1.00 ~ 1.00)	$-1.50*10^{-3}$	$3.86*10^{-4}$	<0.001	1.00 (1.00 ~ 1.00)	$-6.65*10^{-4}$	$5.56*10^{-4}$	0.237	1.00 (1.00 ~ 1.00)
Time (day)	$1.65*10^{-2}$	$5.13*10^{-3}$	0.002	1.02 (1.01 ~ 1.03)	$1.49*10^{-2}$	$3.98*10^{-3}$	<0.001	1.02 (1.01 ~ 1.02)	$2.52*10^{-2}$	$7.91*10^{-3}$	0.004	1.03 (1.01 ~ 1.04)

For covariate definitions and abbreviations, see the note to Tables S18–S37.

Table S32. Subgroup analysis stratified by hypoxemia severity (mild vs. moderate vs. severe), Model 5 (4×ΔP+RR)

	Mild hypoxemia				Moderate hypoxemia				Severe hypoxemia			
**Variables**	***β***	***Se***	***P***	***HR* (95% *CI*)**	***β***	***Se***	***P***	***HR* (95% *CI*)**	***β***	***Se***	***P***	***HR* (95% *CI*)**
Age	$2.76*10^{-2}$	$4.36*10^{-3}$	<0.001	1.03 (1.02 ~ 1.04)	$2.12*10^{-2}$	$3.33*10^{-3}$	<0.001	1.02 (1.01 ~ 1.03)	$8.86*10^{-3}$	$4.61*10^{-3}$	0.055	1.01 (1.00 ~ 1.02)
Non-pulmonary SOFA score	$8.73*10^{-2}$	$2.21*10^{-2}$	<0.001	1.09 (1.04 ~ 1.14)	$9.45*10^{-2}$	$1.70*10^{-2}$	<0.001	1.10 (1.06 ~ 1.14)	$5.05*10^{-2}$	$2.19*10^{-2}$	0.021	1.05 (1.01 ~ 1.10)
Septic shock	$-1.35*10^{-1}$	$1.39*10^{-1}$	0.333	0.87 (0.67 ~ 1.15)	$-6.39*10^{-2}$	$1.15*10^{-1}$	0.577	0.94 (0.75 ~ 1.17)	$1.86*10^{-1}$	$1.60*10^{-1}$	0.245	1.20 (0.88 ~ 1.65)
Chronic lung disease	$-1.54*10^{-1}$	$1.35*10^{-1}$	0.254	0.86 (0.66 ~ 1.12)	$7.58*10^{-2}$	$1.00*10^{-1}$	0.450	1.08 (0.89 ~ 1.31)	$2.85*10^{-1}$	$1.39*10^{-1}$	0.04	1.33 (1.01 ~ 1.74)
Cardiac disease	$2.38*10^{-1}$	$1.39*10^{-1}$	0.087	1.27 (0.97 ~ 1.66)	$-9.83*10^{-2}$	$1.26*10^{-1}$	0.434	0.91 (0.71 ~ 1.16)	$1.27*10^{-1}$	$1.72*10^{-1}$	0.46	1.14 (0.81 ~ 1.59)
Neurologic disease	$-1.06*10^{-2}$	$1.41*10^{-1}$	0.940	0.99 (0.75 ~ 1.30)	$-6.50*10^{-2}$	$1.19*10^{-1}$	0.584	0.94 (0.74 ~ 1.18)	$-4.64*10^{-2}$	$1.72*10^{-1}$	0.788	0.95 (0.68 ~ 1.34)
Liver disease	$3.47*10^{-1}$	$2.25*10^{-1}$	0.124	1.42 (0.91 ~ 2.20)	$1.83*10^{-1}$	$2.41*10^{-1}$	0.447	1.20 (0.75 ~ 1.93)	$3.39*10^{-1}$	$3.65*10^{-1}$	0.353	1.40 (0.69 ~ 2.87)
Chronic kidney disease	$-3.42*10^{-1}$	$1.84*10^{-1}$	0.064	0.71 (0.50 ~ 1.02)	$-1.53*10^{-1}$	$1.46*10^{-1}$	0.295	0.86 (0.64 ~ 1.14)	$-8.63*10^{-2}$	$2.25*10^{-1}$	0.702	0.92 (0.59 ~ 1.43)
Immunocompromised status	$2.42*10^{-1}$	$1.18*10^{-1}$	0.041	1.27 (1.01 ~ 1.60)	$1.16*10^{-1}$	$9.51*10^{-2}$	0.221	1.12 (0.93 ~ 1.35)	$2.68*10^{-1}$	$1.34*10^{-1}$	0.046	1.31 (1.00 ~ 1.70)
Bacterial infection	$-1.91*10^{-1}$	$1.74*10^{-1}$	0.271	0.83 (0.59 ~ 1.16)	$-1.63*10^{-1}$	$1.35*10^{-1}$	0.229	0.85 (0.65 ~ 1.11)	$1.70*10^{-2}$	$2.21*10^{-1}$	0.939	1.02 (0.66 ~ 1.57)
Viral infection	$2.26*10^{-1}$	$1.37*10^{-1}$	0.100	1.25 (0.96 ~ 1.64)	$5.21*10^{-2}$	$1.07*10^{-1}$	0.625	1.05 (0.86 ~ 1.30)	$-3.17*10^{-1}$	$1.41*10^{-1}$	0.025	0.73 (0.55 ~ 0.96)
Fungal infection	$3.76*10^{-1}$	$1.23*10^{-1}$	0.002	1.46 (1.14 ~ 1.85)	$5.20*10^{-2}$	$9.60*10^{-2}$	0.588	1.05 (0.87 ~ 1.27)	$2.07*10^{-1}$	$1.41*10^{-1}$	0.142	1.23 (0.93 ~ 1.62)
Systemic glucocorticoid	$2.40*10^{-1}$	$1.28*10^{-1}$	0.062	1.27 (0.99 ~ 1.63)	$4.36*10^{-1}$	$1.06*10^{-1}$	<0.001	1.55 (1.26 ~ 1.90)	$1.46*10^{-1}$	$1.96*10^{-1}$	0.457	1.16 (0.79 ~ 1.70)
Neuromuscular blocker	$6.28*10^{-1}$	$1.52*10^{-1}$	<0.001	1.87 (1.39 ~ 2.53)	$3.08*10^{-1}$	$1.05*10^{-1}$	0.003	1.36 (1.11 ~ 1.67)	$2.57*10^{-1}$	$1.52*10^{-1}$	0.091	1.29 (0.96 ~ 1.74)
Mean arterial pressure (MAP)	$-2.48*10^{-3}$	$2.09*10^{-3}$	0.235	1.00 (0.99 ~ 1.00)	$4.71*10^{-9}$	$4.70*10^{-10}$	<0.001	1.00 (1.00 ~ 1.00)	$1.36*10^{-4}$	$1.98*10^{-4}$	0.496	1.00 (1.00 ~ 1.00)
Heart rate (HR)	$6.85*10^{-3}$	$1.23*10^{-3}$	<0.001	1.01 (1.00 ~ 1.01)	$9.17*10^{-3}$	$1.24*10^{-3}$	<0.001	1.01 (1.01 ~ 1.01)	$6.71*10^{-3}$	$1.81*10^{-3}$	<0.001	1.01 (1.00 ~ 1.01)
PEEP	$4.67*10^{-2}$	$1.97*10^{-2}$	0.047	1.05 (1.01 ~ 1.09)	$4.45*10^{-2}$	$1.55*10^{-2}$	0.014	1.05 (1.01 ~ 1.08)	$1.69*10^{-2}$	$1.27*10^{-2}$	0.186	1.02 (0.99 ~ 1.04)
[(4×ΔP)+RR]	$1.02*10^{-3}$	$1.13*10^{-3}$	0.374	1.00 (1.00 ~ 1.00)	$6.31*10^{-4}$	$4.84*10^{-4}$	0.224	1.00 (1.00 ~ 1.00)	$2.26*10^{-3}$	$2.09*10^{-3}$	0.285	1.00 (1.00 ~ 1.01)
Fluid balance (input − output)	$4.57*10^{-5}$	$5.05*10^{-5}$	0.405	1.00 (1.00 ~ 1.00)	$1.93*10^{-5}$	$3.14*10^{-5}$	0.565	1.00 (1.00 ~ 1.00)	$1.83*10^{-5}$	$2.61*10^{-5}$	0.490	1.00 (1.00 ~ 1.00)
PaCO2	$5.00*10^{-4}$	$4.52*10^{-3}$	0.913	1.00 (0.99 ~ 1.01)	$1.13*10^{-2}$	$2.76*10^{-3}$	<0.001	1.01 (1.01 ~ 1.02)	$4.72*10^{-3}$	$3.31*10^{-3}$	0.155	1.00 (1.00 ~ 1.01)
pH	-1.32	$5.46*10^{-1}$	0.026	0.27 (0.09 ~ 0.78)	$3.30*10^{-1}$	$3.93*10^{-1}$	0.407	1.39 (0.64 ~ 3.00)	$-7.21*10^{-1}$	$4.94*10^{-1}$	0.154	0.49 (0.18 ~ 1.28)

Mild hypoxemia

Variables	*β*	*Se*	*P*	*HR (95% CI)*
Lactate	$1.25*10^{-1}$	$1.85*10^{-2}$	<0.001	1.13 (1.09 ~ 1.17)
C-reactive protein (CRP)	$1.14*10^{-3}$	$5.36*10^{-4}$	0.050	1.00 (1.00 ~ 1.00)
Interleukin-6 (IL-6)	$1.22*10^{-4}$	$4.42*10^{-5}$	0.011	1.00 (1.00 ~ 1.00)
Procalcitonin (PCT)	$-1.67*10^{-3}$	$3.20*10^{-3}$	0.603	1.00 (0.99 ~ 1.00)
B-type natriuretic peptide (BNP)	$1.03*10^{-5}$	$5.10*10^{-6}$	0.045	1.00 (1.00 ~ 1.00)
Albumin	$-5.34*10^{-3}$	$6.51*10^{-3}$	0.412	0.99 (0.98 ~ 1.01)
Absolute neutrophil count (ANC)	$2.02*10^{-2}$	$6.10*10^{-3}$	0.001	1.02 (1.01 ~ 1.03)
Absolute lymphocyte count (ALC)	$-7.49*10^{-2}$	$7.45*10^{-2}$	0.320	0.93 (0.80 ~ 1.07)
Hemoglobin (Hb)	$-8.28*10^{-4}$	$2.19*10^{-3}$	0.705	1.00 (0.99 ~ 1.00)
D-dimer	$3.32*10^{-3}$	$4.64*10^{-3}$	0.483	1.00 (0.99 ~ 1.01)
Creatinine (Cr)	$5.90*10^{-4}$	$4.23*10^{-4}$	0.163	1.00 (1.00 ~ 1.00)
Platelet count (PLT)	$-1.39*10^{-3}$	$5.07*10^{-4}$	0.006	1.00 (1.00 ~ 1.00)
Time (day)	$1.66*10^{-2}$	$5.21*10^{-3}$	0.002	1.02 (1.01 ~ 1.03)

Moderate hypoxemia

Variables	*β*	*Se*	*P*	*HR (95% CI)*
Lactate	$1.10*10^{-1}$	$2.37*10^{-2}$	0.002	1.12 (1.07 ~ 1.17)
C-reactive protein (CRP)	$1.15*10^{-3}$	$3.31*10^{-4}$	0.001	1.00 (1.00 ~ 1.00)
Interleukin-6 (IL-6)	$1.28*10^{-4}$	$2.83*10^{-5}$	<0.001	1.00 (1.00 ~ 1.00)
Procalcitonin (PCT)	$-1.74*10^{-3}$	$2.09*10^{-3}$	0.407	1.00 (0.99 ~ 1.00)
B-type natriuretic peptide (BNP)	$1.03*10^{-5}$	$4.11*10^{-6}$	0.014	1.00 (1.00 ~ 1.00)
Albumin	$-6.53*10^{-3}$	$5.35*10^{-3}$	0.224	0.99 (0.98 ~ 1.00)
Absolute neutrophil count (ANC)	$2.36*10^{-2}$	$5.15*10^{-3}$	<0.001	1.02 (1.01 ~ 1.03)
Absolute lymphocyte count (ALC)	$-7.94*10^{-2}$	$5.04*10^{-2}$	0.116	0.92 (0.84 ~ 1.02)
Hemoglobin (Hb)	$-1.63*10^{-3}$	$1.57*10^{-3}$	0.297	1.00 (1.00 ~ 1.00)
D-dimer	$-8.21*10^{-5}$	$2.07*10^{-4}$	0.699	1.00 (1.00 ~ 1.00)
Creatinine (Cr)	$8.50*10^{-4}$	$2.94*10^{-4}$	0.004	1.00 (1.00 ~ 1.00)
Platelet count (PLT)	$-1.49*10^{-3}$	$3.87*10^{-4}$	<0.001	1.00 (1.00 ~ 1.00)
Time (day)	$1.55*10^{-2}$	$4.07*10^{-3}$	<0.001	1.02 (1.01 ~ 1.02)

Severe hypoxemia

Variables	*β*	*Se*	*P*	*HR (95% CI)*
Lactate	$1.07*10^{-1}$	$2.26*10^{-2}$	<0.001	1.11 (1.06 ~ 1.16)
C-reactive protein (CRP)	$5.07*10^{-4}$	$3.62*10^{-4}$	0.162	1.00 (1.00 ~ 1.00)
Interleukin-6 (IL-6)	$1.56*10^{-4}$	$4.59*10^{-5}$	0.003	1.00 (1.00 ~ 1.00)
Procalcitonin (PCT)	$-1.98*10^{-3}$	$3.07*10^{-3}$	0.520	1.00 (0.99 ~ 1.00)
B-type natriuretic peptide (BNP)	$8.20*10^{-6}$	$4.80*10^{-6}$	0.088	1.00 (1.00 ~ 1.00)
Albumin	$-1.57*10^{-2}$	$7.83*10^{-3}$	0.049	0.98 (0.97 ~ 1.00)
Absolute neutrophil count (ANC)	$2.47*10^{-3}$	$7.26*10^{-3}$	0.735	1.00 (0.99 ~ 1.02)
Absolute lymphocyte count (ALC)	$-3.17*10^{-2}$	$6.59*10^{-2}$	0.632	0.97 (0.85 ~ 1.10)
Hemoglobin (Hb)	$1.62*10^{-3}$	$2.26*10^{-3}$	0.476	1.00 (1.00 ~ 1.01)
D-dimer	$3.90*10^{-3}$	$4.65*10^{-3}$	0.412	1.00 (0.99 ~ 1.01)
Creatinine (Cr)	$5.30*10^{-4}$	$5.31*10^{-4}$	0.324	1.00 (1.00 ~ 1.00)
Platelet count (PLT)	$-6.61*10^{-4}$	$5.53*10^{-4}$	0.237	1.00 (1.00 ~ 1.00)
Time (day)	$2.55*10^{-2}$	$7.81*10^{-3}$	0.003	1.03 (1.01 ~ 1.04)

For covariate definitions and abbreviations, see the note to Tables S18–S37.

Table S33. Subgroup analysis stratified by immunocompromised status (immunocompromised vs. non-immunocompromised), Model 1 (VT/kg)

	Immunocompetent				Immunocompromised			
**Variables**	***β***	***Se***	***P***	***HR* (95% *CI*)**	***β***	***Se***	***P***	***HR* (95% *CI*)**
Age	$2.84*10^{-2}$	$3.41*10^{-3}$	<0.001	1.03 (1.02 ~ 1.04)	$1.15*10^{-2}$	$3.39*10^{-3}$	0.001	1.01 (1.00 ~ 1.02)
Non-pulmonary SOFA score	$7.52*10^{-2}$	$1.68*10^{-2}$	<0.001	1.08 (1.04 ~ 1.11)	$9.67*10^{-2}$	$1.72*10^{-2}$	<0.001	1.10 (1.07 ~ 1.14)
Septic shock	$-4.51*10^{-4}$	$1.11*10^{-1}$	0.997	1.00 (0.80 ~ 1.24)	$-7.30*10^{-2}$	$1.13*10^{-1}$	0.517	0.93 (0.75 ~ 1.16)
Chronic lung disease	$-2.36*10^{-2}$	$1.01*10^{-1}$	0.816	0.98 (0.80 ~ 1.19)	$1.56*10^{-1}$	$1.04*10^{-1}$	0.136	1.17 (0.95 ~ 1.43)
Cardiac disease	$1.24*10^{-1}$	$1.13*10^{-1}$	0.272	1.13 (0.91 ~ 1.41)	$2.67*10^{-3}$	$1.25*10^{-1}$	0.983	1.00 (0.79 ~ 1.28)
Neurologic disease	$-1.18*10^{-1}$	$1.16*10^{-1}$	0.306	0.89 (0.71 ~ 1.11)	$4.23*10^{-2}$	$1.19*10^{-1}$	0.722	1.04 (0.83 ~ 1.32)
Liver disease	$3.87*10^{-1}$	$1.95*10^{-1}$	0.047	1.47 (1.00 ~ 2.16)	$5.94*10^{-3}$	$2.51*10^{-1}$	0.981	1.01 (0.61 ~ 1.65)
Chronic kidney disease	$-3.59*10^{-1}$	$1.54*10^{-1}$	0.020	0.70 (0.52 ~ 0.94)	$-1.20*10^{-1}$	$1.49*10^{-1}$	0.419	0.89 (0.66 ~ 1.19)
Bacterial infection	$-1.65*10^{-1}$	$1.40*10^{-1}$	0.241	0.85 (0.64 ~ 1.12)	$-1.42*10^{-1}$	$1.41*10^{-1}$	0.313	0.87 (0.66 ~ 1.14)
Viral infection	$5.30*10^{-3}$	$1.15*10^{-1}$	0.963	1.01 (0.80 ~ 1.26)	$1.73*10^{-2}$	$1.02*10^{-1}$	0.866	1.02 (0.83 ~ 1.24)
Fungal infection	$2.29*10^{-1}$	$9.36*10^{-2}$	0.015	1.26 (1.05 ~ 1.51)	$1.25*10^{-1}$	$1.02*10^{-1}$	0.223	1.13 (0.93 ~ 1.38)
Systemic glucocorticoid	$3.55*10^{-1}$	$1.02*10^{-1}$	<0.001	1.43 (1.17 ~ 1.74)	$2.68*10^{-1}$	$1.22*10^{-1}$	0.028	1.31 (1.03 ~ 1.66)
Neuromuscular blocker	$6.76*10^{-1}$	$1.10*10^{-1}$	<0.001	1.97 (1.58 ~ 2.44)	$2.27*10^{-1}$	$1.04*10^{-1}$	0.029	1.26 (1.02 ~ 1.54)
Mean arterial pressure (MAP)	$4.70*10^{-9}$	$4.71*10^{-10}$	<0.001	1.00 (1.00 ~ 1.00)	$1.26*10^{-4}$	$2.80*10^{-4}$	0.653	1.00 (1.00 ~ 1.00)
Heart rate (HR)	$8.02*10^{-3}$	$1.39*10^{-3}$	<0.001	1.01 (1.01 ~ 1.01)	$7.35*10^{-3}$	$1.13*10^{-3}$	<0.001	1.01 (1.01 ~ 1.01)
Respiratory rate (RR)	$1.63*10^{-3}$	$2.71*10^{-3}$	0.548	1.00 (1.00 ~ 1.01)	$3.09*10^{-3}$	$2.15*10^{-3}$	0.153	1.00 (1.00 ~ 1.01)
PEEP	$5.73*10^{-2}$	$9.82*10^{-3}$	<0.001	1.06 (1.04 ~ 1.08)	$2.33*10^{-2}$	$8.21*10^{-3}$	0.005	1.02 (1.01 ~ 1.04)
Tidal volume per body weight (VT/kg)	$-3.18*10^{-3}$	$9.42*10^{-4}$	0.002	1.00 (0.99 ~ 1.00)	$-4.00*10^{-4}$	$1.11*10^{-2}$	0.972	1.00 (0.98 ~ 1.02)
Fluid balance (input − output)	$1.84*10^{-11}$	1.83*10-12	<0.001	1.00 (1.00 ~ 1.00)	$1.56*10^{-5}$	$2.01*10^{-5}$	0.441	1.00 (1.00 ~ 1.00)
PaO_2/FiO_2 ratio	$-1.01*10^{-3}$	$2.58*10^{-4}$	<0.001	1.00 (1.00 ~ 1.00)	$-1.43*10^{-3}$	$3.17*10^{-4}$	<0.001	1.00 (1.00 ~ 1.00)
PaCO2	$3.72*10^{-3}$	$2.67*10^{-3}$	0.167	1.00 (1.00 ~ 1.01)	$7.27*10^{-3}$	$2.72*10^{-3}$	0.008	1.01 (1.00 ~ 1.01)

	Immunocompetent				Immunocompromised			
**Variables**	***β***	***Se***	***P***	***HR* (95% *CI*)**	***β***	***Se***	***P***	***HR* (95% *CI*)**
pH	-1.06	$3.64*10^{-1}$	0.008	0.35 (0.17 ~ 0.71)	$2.93*10^{-2}$	$3.57*10^{-1}$	0.935	1.03 (0.51 ~ 2.07)
Lactate	$1.00*10^{-1}$	$1.73*10^{-2}$	<0.001	1.11 (1.07 ~ 1.14)	$1.28*10^{-1}$	$1.74*10^{-2}$	<0.001	1.14 (1.10 ~ 1.18)
C-reactive protein (CRP)	$9.86*10^{-4}$	$3.31*10^{-4}$	0.004	1.00 (1.00 ~ 1.00)	$9.63*10^{-4}$	$3.54*10^{-4}$	0.01	1.00 (1.00 ~ 1.00)
Interleukin-6 (IL-6)	$1.29*10^{-4}$	$3.63*10^{-5}$	0.003	1.00 (1.00 ~ 1.00)	$1.22*10^{-4}$	$3.17*10^{-5}$	<0.001	1.00 (1.00 ~ 1.00)
Procalcitonin (PCT)	$-2.05*10^{-3}$	$2.30*10^{-3}$	0.375	1.00 (0.99 ~ 1.00)	$-1.62*10^{-3}$	$2.58*10^{-3}$	0.531	1.00 (0.99 ~ 1.00)
B-type natriuretic peptide (BNP)	$8.12*10^{-6}$	$3.77*10^{-6}$	0.031	1.00 (1.00 ~ 1.00)	$1.08*10^{-5}$	$3.99*10^{-6}$	0.007	1.00 (1.00 ~ 1.00)
Albumin	$-1.19*10^{-2}$	$5.39*10^{-3}$	0.027	0.99 (0.98 ~ 1.00)	$-7.11*10^{-3}$	$5.39*10^{-3}$	0.190	0.99 (0.98 ~ 1.00)
Absolute neutrophil count (ANC)	$1.92*10^{-2}$	$4.73*10^{-3}$	<0.001	1.02 (1.01 ~ 1.03)	$1.83*10^{-2}$	$4.91*10^{-3}$	<0.001	1.02 (1.01 ~ 1.03)
Absolute lymphocyte count (ALC)	$-1.18*10^{-1}$	$5.50*10^{-2}$	0.035	0.89 (0.80 ~ 0.99)	$-3.29*10^{-2}$	$3.53*10^{-2}$	0.352	0.97 (0.90 ~ 1.04)
Hemoglobin (Hb)	$-2.63*10^{-3}$	$1.60*10^{-3}$	0.102	1.00 (0.99 ~ 1.00)	$1.39*10^{-3}$	$1.55*10^{-3}$	0.369	1.00 (1.00 ~ 1.00)
D-dimer	$2.71*10^{-5}$	$1.23*10^{-4}$	0.828	1.00 (1.00 ~ 1.00)	$3.80*10^{-4}$	$3.40*10^{-3}$	0.911	1.00 (0.99 ~ 1.01)
Creatinine (Cr)	$1.07*10^{-3}$	$3.04*10^{-4}$	0.001	1.00 (1.00 ~ 1.00)	$3.54*10^{-4}$	$3.62*10^{-4}$	0.328	1.00 (1.00 ~ 1.00)
Platelet count (PLT)	$-1.12*10^{-3}$	$3.61*10^{-4}$	0.002	1.00 (1.00 ~ 1.00)	$-1.59*10^{-3}$	$3.86*10^{-4}$	<0.001	1.00 (1.00 ~ 1.00)
Time (day)	$2.12*10^{-2}$	$3.90*10^{-3}$	<0.001	1.02 (1.01 ~ 1.03)	$1.86*10^{-2}$	$3.74*10^{-3}$	<0.001	1.02 (1.01 ~ 1.03)

For covariate definitions and abbreviations, see the note to Tables S18–S37.

Table S34. Subgroup analysis stratified by immunocompromised status (immunocompromised vs. non-immunocompromised), Model 2 (VE)

	Immunocompetent				Immunocompromised			
**Variables**	***β***	***Se***	***P***	***HR* (95% *CI*)**	***β***	***Se***	***P***	***HR* (95% *CI*)**
Age	$2.84*10^{-2}$	$3.41*10^{-3}$	<0.001	1.03 (1.02 ~ 1.04)	$1.15*10^{-2}$	$3.40*10^{-3}$	0.001	1.01 (1.00 ~ 1.02)
Non-pulmonary SOFA score	$7.49*10^{-2}$	$1.68*10^{-2}$	<0.001	1.08 (1.04 ~ 1.11)	$9.64*10^{-2}$	$1.72*10^{-2}$	<0.001	1.10 (1.06 ~ 1.14)
Septic shock	$-1.61*10^{-4}$	$1.11*10^{-1}$	0.999	1.00 (0.80 ~ 1.24)	$-7.32*10^{-2}$	$1.13*10^{-1}$	0.516	0.93 (0.75 ~ 1.16)
Chronic lung disease	$-2.21*10^{-2}$	$1.01*10^{-1}$	0.827	0.98 (0.80 ~ 1.19)	$1.56*10^{-1}$	$1.04*10^{-1}$	0.135	1.17 (0.95 ~ 1.43)
Cardiac disease	$1.23*10^{-1}$	$1.13*10^{-1}$	0.277	1.13 (0.91 ~ 1.41)	$1.31*10^{-3}$	$1.25*10^{-1}$	0.992	1.00 (0.78 ~ 1.28)
Neurologic disease	$-1.19*10^{-1}$	$1.16*10^{-1}$	0.302	0.89 (0.71 ~ 1.11)	$4.24*10^{-2}$	$1.19*10^{-1}$	0.721	1.04 (0.83 ~ 1.32)
Liver disease	$3.87*10^{-1}$	$1.96*10^{-1}$	0.048	1.47 (1.00 ~ 2.16)	$7.24*10^{-3}$	$2.51*10^{-1}$	0.977	1.01 (0.62 ~ 1.65)
Chronic kidney disease	$-3.58*10^{-1}$	$1.54*10^{-1}$	0.020	0.70 (0.52 ~ 0.94)	$-1.20*10^{-1}$	$1.49*10^{-1}$	0.420	0.89 (0.66 ~ 1.19)
Bacterial infection	$-1.62*10^{-1}$	$1.41*10^{-1}$	0.249	0.85 (0.65 ~ 1.12)	$-1.41*10^{-1}$	$1.41*10^{-1}$	0.317	0.87 (0.66 ~ 1.15)
Viral infection	$7.43*10^{-3}$	$1.15*10^{-1}$	0.949	1.01 (0.80 ~ 1.26)	$1.77*10^{-2}$	$1.02*10^{-1}$	0.863	1.02 (0.83 ~ 1.24)
Fungal infection	$2.27*10^{-1}$	$9.35*10^{-2}$	0.015	1.26 (1.04 ~ 1.51)	$1.24*10^{-1}$	$1.02*10^{-1}$	0.226	1.13 (0.93 ~ 1.38)
Systemic glucocorticoid	$3.55*10^{-1}$	$1.02*10^{-1}$	<0.001	1.43 (1.17 ~ 1.74)	$2.70*10^{-1}$	$1.22*10^{-1}$	0.027	1.31 (1.03 ~ 1.66)
Neuromuscular blocker	$6.78*10^{-1}$	$1.10*10^{-1}$	<0.001	1.97 (1.59 ~ 2.44)	$2.33*10^{-1}$	$1.04*10^{-1}$	0.025	1.26 (1.03 ~ 1.55)
Mean arterial pressure (MAP)	$4.70*10^{-9}$	$4.71*10^{-10}$	<0.001	1.00 (1.00 ~ 1.00)	$1.28*10^{-4}$	$2.73*10^{-4}$	0.639	1.00 (1.00 ~ 1.00)
Heart rate (HR)	$8.17*10^{-3}$	$1.41*10^{-3}$	<0.001	1.01 (1.01 ~ 1.01)	$7.39*10^{-3}$	$1.14*10^{-3}$	<0.001	1.01 (1.01 ~ 1.01)
PEEP	$5.75*10^{-2}$	$9.87*10^{-3}$	<0.001	1.06 (1.04 ~ 1.08)	$2.34*10^{-2}$	$8.18*10^{-3}$	0.005	1.02 (1.01 ~ 1.04)
Minute ventilation (VE)	$-2.06*10^{-3}$	$9.47*10^{-3}$	0.828	1.00 (0.98 ~ 1.02)	$2.24*10^{-3}$	$8.04*10^{-3}$	0.781	1.00 (0.99 ~ 1.02)
Fluid balance (input − output)	$1.84*10^{-11}$	1.83*10-12	<0.001	1.00 (1.00 ~ 1.00)	$1.57*10^{-5}$	$2.01*10^{-5}$	0.439	1.00 (1.00 ~ 1.00)
PaO_2/FiO_2 ratio	$-1.02*10^{-3}$	$2.58*10^{-4}$	<0.001	1.00 (1.00 ~ 1.00)	$-1.45*10^{-3}$	$3.17*10^{-4}$	<0.001	1.00 (1.00 ~ 1.00)
PaCO2	$3.73*10^{-3}$	$2.66*10^{-3}$	0.166	1.00 (1.00 ~ 1.01)	$7.33*10^{-3}$	$2.71*10^{-3}$	0.007	1.01 (1.00 ~ 1.01)
pH	-1.07	$3.61*10^{-1}$	0.006	0.34 (0.17 ~ 0.7)	$2.37*10^{-2}$	$3.57*10^{-1}$	0.947	1.02 (0.51 ~ 2.06)

	Immunocompetent				Immunocompromised			
**Variables**	***β***	***Se***	***P***	***HR* (95% *CI*)**	***β***	***Se***	***P***	***HR* (95% *CI*)**
Lactate	$9.99*10^{-2}$	$1.73*10^{-2}$	<0.001	1.11 (1.07 ~ 1.14)	$1.28*10^{-1}$	$1.73*10^{-2}$	<0.001	1.14 (1.10 ~ 1.18)
C-reactive protein (CRP)	$9.95*10^{-4}$	$3.31*10^{-4}$	0.004	1.00 (1.00 ~ 1.00)	$9.61*10^{-4}$	$3.54*10^{-4}$	0.010	1.00 (1.00 ~ 1.00)
Interleukin-6 (IL-6)	$1.30*10^{-4}$	$3.60*10^{-5}$	0.003	1.00 (1.00 ~ 1.00)	$1.22*10^{-4}$	$3.17*10^{-5}$	<0.001	1.00 (1.00 ~ 1.00)
Procalcitonin (PCT)	$-2.09*10^{-3}$	$2.30*10^{-3}$	0.367	1.00 (0.99 ~ 1.00)	$-1.59*10^{-3}$	$2.58*10^{-3}$	0.542	1.00 (0.99 ~ 1.00)
B-type natriuretic peptide (BNP)	$8.09*10^{-6}$	$3.77*10^{-6}$	0.032	1.00 (1.00 ~ 1.00)	$1.08*10^{-5}$	$3.98*10^{-6}$	0.007	1.00 (1.00 ~ 1.00)
Albumin	$-1.19*10^{-2}$	$5.39*10^{-3}$	0.028	0.99 (0.98 ~ 1.00)	$-7.18*10^{-3}$	$5.40*10^{-3}$	0.187	0.99 (0.98 ~ 1.00)
Absolute neutrophil count (ANC)	$1.92*10^{-2}$	$4.73*10^{-3}$	<0.001	1.02 (1.01 ~ 1.03)	$1.83*10^{-2}$	$4.92*10^{-3}$	<0.001	1.02 (1.01 ~ 1.03)
Absolute lymphocyte count (ALC)	$-1.18*10^{-1}$	$5.53*10^{-2}$	0.035	0.89 (0.80 ~ 0.99)	$-3.34*10^{-2}$	$3.55*10^{-2}$	0.347	0.97 (0.90 ~ 1.04)
Hemoglobin (Hb)	$-2.63*10^{-3}$	$1.61*10^{-3}$	0.102	1.00 (0.99 ~ 1.00)	$1.35*10^{-3}$	$1.55*10^{-3}$	0.383	1.00 (1.00 ~ 1.00)
D-dimer	$2.71*10^{-5}$	$1.24*10^{-4}$	0.828	1.00 (1.00 ~ 1.00)	$3.78*10^{-4}$	$3.40*10^{-3}$	0.912	1.00 (0.99 ~ 1.01)
Creatinine (Cr)	$1.07*10^{-3}$	$3.01*10^{-4}$	<0.001	1.00 (1.00 ~ 1.00)	$3.47*10^{-4}$	$3.63*10^{-4}$	0.340	1.00 (1.00 ~ 1.00)
Platelet count (PLT)	$-1.11*10^{-3}$	$3.61*10^{-4}$	0.002	1.00 (1.00 ~ 1.00)	$-1.59*10^{-3}$	$3.86*10^{-4}$	<0.001	1.00 (1.00 ~ 1.00)
Time (day)	$2.17*10^{-2}$	$3.87*10^{-3}$	<0.001	1.02 (1.01 ~ 1.03)	$1.90*10^{-2}$	$3.74*10^{-3}$	<0.001	1.02 (1.01 ~ 1.03)

For covariate definitions and abbreviations, see the note to Tables S18–S37.

Table S35. Subgroup analysis stratified by immunocompromised status (immunocompromised vs. non-immunocompromised), Model 3 (VR)

	Immunocompetent				Immunocompromised			
**Variables**	***β***	***Se***	***P***	***HR* (95% *CI*)**	***β***	***Se***	***P***	***HR* (95% *CI*)**
Age	$2.85*10^{-2}$	$3.41*10^{-3}$	<0.001	1.03 (1.02 ~ 1.04)	$1.19*10^{-2}$	$3.39*10^{-3}$	<0.001	1.01 (1.01 ~ 1.02)
Non-pulmonary SOFA score	$7.42*10^{-2}$	$1.68*10^{-2}$	<0.001	1.08 (1.04 ~ 1.11)	$9.58*10^{-2}$	$1.71*10^{-2}$	<0.001	1.10 (1.06 ~ 1.14)
Septic shock	$9.23*10^{-4}$	$1.12*10^{-1}$	0.993	1.00 (0.80 ~ 1.25)	$-7.63*10^{-2}$	$1.13*10^{-1}$	0.498	0.93 (0.74 ~ 1.16)
Chronic lung disease	$-9.11*10^{-3}$	$1.01*10^{-1}$	0.928	0.99 (0.81 ~ 1.21)	$1.74*10^{-1}$	$1.04*10^{-1}$	0.094	1.19 (0.97 ~ 1.46)
Cardiac disease	$1.21*10^{-1}$	$1.13*10^{-1}$	0.285	1.13 (0.90 ~ 1.41)	$-1.84*10^{-3}$	$1.25*10^{-1}$	0.988	1.00 (0.78 ~ 1.28)
Neurologic disease	$-1.20*10^{-1}$	$1.15*10^{-1}$	0.298	0.89 (0.71 ~ 1.11)	$4.08*10^{-2}$	$1.19*10^{-1}$	0.731	1.04 (0.83 ~ 1.31)
Liver disease	$3.79*10^{-1}$	$1.96*10^{-1}$	0.053	1.46 (0.99 ~ 2.14)	$3.78*10^{-2}$	$2.46*10^{-1}$	0.878	1.04 (0.64 ~ 1.68)
Chronic kidney disease	$-3.61*10^{-1}$	$1.54*10^{-1}$	0.019	0.70 (0.52 ~ 0.94)	$-1.28*10^{-1}$	$1.49*10^{-1}$	0.389	0.88 (0.66 ~ 1.18)
Bacterial infection	$-1.58*10^{-1}$	$1.41*10^{-1}$	0.262	0.85 (0.65 ~ 1.13)	$-1.41*10^{-1}$	$1.40*10^{-1}$	0.314	0.87 (0.66 ~ 1.14)
Viral infection	$9.68*10^{-3}$	$1.15*10^{-1}$	0.933	1.01 (0.81 ~ 1.27)	$1.98*10^{-2}$	$1.02*10^{-1}$	0.846	1.02 (0.83 ~ 1.25)
Fungal infection	$2.32*10^{-1}$	$9.35*10^{-2}$	0.013	1.26 (1.05 ~ 1.52)	$1.27*10^{-1}$	$1.02*10^{-1}$	0.216	1.14 (0.93 ~ 1.39)
Systemic glucocorticoid	$3.57*10^{-1}$	$1.02*10^{-1}$	<0.001	1.43 (1.17 ~ 1.75)	$2.73*10^{-1}$	$1.22*10^{-1}$	0.025	1.31 (1.04 ~ 1.67)
Neuromuscular blocker	$6.85*10^{-1}$	$1.09*10^{-1}$	<0.001	1.98 (1.60 ~ 2.46)	$2.46*10^{-1}$	$1.04*10^{-1}$	0.018	1.28 (1.04 ~ 1.57)
Mean arterial pressure (MAP)	$4.69*10^{-9}$	$4.71*10^{-10}$	<0.001	1.00 (1.00 ~ 1.00)	$1.25*10^{-4}$	$2.84*10^{-4}$	0.662	1.00 (1.00 ~ 1.00)
Heart rate (HR)	$8.25*10^{-3}$	$1.35*10^{-3}$	<0.001	1.01 (1.01 ~ 1.01)	$7.41*10^{-3}$	$1.14*10^{-3}$	<0.001	1.01 (1.01 ~ 1.01)
PEEP	$5.80*10^{-2}$	$9.83*10^{-3}$	<0.001	1.06 (1.04 ~ 1.08)	$2.28*10^{-2}$	$8.39*10^{-3}$	0.007	1.02 (1.01 ~ 1.04)
Ventilatory ratio (VR)	$-1.18*10^{-2}$	$8.32*10^{-3}$	0.157	0.99 (0.97 ~ 1.00)	$4.94*10^{-2}$	$5.26*10^{-2}$	0.351	1.05 (0.95 ~ 1.16)
Fluid balance (input − output)	$1.83*10^{-11}$	1.83*10-12	<0.001	1.00 (1.00 ~ 1.00)	$1.22*10^{-5}$	$1.93*10^{-5}$	0.528	1.00 (1.00 ~ 1.00)
PaO_2/FiO_2 ratio	$-1.11*10^{-3}$	$2.56*10^{-4}$	<0.001	1.00 (1.00 ~ 1.00)	$-1.58*10^{-3}$	$3.15*10^{-4}$	<0.001	1.00 (1.00 ~ 1.00)
pH	-1.38	$2.53*10^{-1}$	<0.001	0.25 (0.15 ~ 0.41)	$-4.55*10^{-1}$	$3.22*10^{-1}$	0.163	0.63 (0.34 ~ 1.19)
Lactate	$9.68*10^{-2}$	$1.61*10^{-2}$	<0.001	1.10 (1.07 ~ 1.14)	$1.24*10^{-1}$	$1.63*10^{-2}$	<0.001	1.13 (1.10 ~ 1.17)

	Immunocompetent				Immunocompromised			
Variables	*β*	*Se*	*P*	*HR* (95% *CI*)	*β*	*Se*	*P*	*HR* (95% *CI*)
C-reactive protein (CRP)	$9.86*10^{-4}$	$3.32*10^{-4}$	0.004	1.00 (1.00 ~ 1.00)	$9.79*10^{-4}$	$3.59*10^{-4}$	0.011	1.00 (1.00 ~ 1.00)
Interleukin-6 (IL-6)	$1.29*10^{-4}$	$3.60*10^{-5}$	0.003	1.00 (1.00 ~ 1.00)	$1.20*10^{-4}$	$3.19*10^{-5}$	<0.001	1.00 (1.00 ~ 1.00)
Procalcitonin (PCT)	$-2.28*10^{-3}$	$2.29*10^{-3}$	0.322	1.00 (0.99 ~ 1.00)	$-1.71*10^{-3}$	$2.60*10^{-3}$	0.515	1.00 (0.99 ~ 1.00)
B-type natriuretic peptide (BNP)	$8.36*10^{-6}$	$3.78*10^{-6}$	0.027	1.00 (1.00 ~ 1.00)	$1.08*10^{-5}$	$3.99*10^{-6}$	0.007	1.00 (1.00 ~ 1.00)
Albumin	$-1.15*10^{-2}$	$5.37*10^{-3}$	0.032	0.99 (0.98 ~ 1.00)	$-6.59*10^{-3}$	$5.37*10^{-3}$	0.223	0.99 (0.98 ~ 1.00)
Absolute neutrophil count (ANC)	$1.97*10^{-2}$	$4.74*10^{-3}$	<0.001	1.02 (1.01 ~ 1.03)	$1.86*10^{-2}$	$4.92*10^{-3}$	<0.001	1.02 (1.01 ~ 1.03)
Absolute lymphocyte count (ALC)	$-1.21*10^{-1}$	$5.51*10^{-2}$	0.031	0.89 (0.80 ~ 0.99)	$-3.52*10^{-2}$	$3.49*10^{-2}$	0.314	0.97 (0.9 ~ 1.03)
Hemoglobin (Hb)	$-2.55*10^{-3}$	$1.61*10^{-3}$	0.113	1.00 (0.99 ~ 1.00)	$1.48*10^{-3}$	$1.55*10^{-3}$	0.339	1.00 (1.00 ~ 1.00)
D-dimer	$2.73*10^{-5}$	$1.23*10^{-4}$	0.827	1.00 (1.00 ~ 1.00)	$2.34*10^{-4}$	$3.41*10^{-3}$	0.945	1.00 (0.99 ~ 1.01)
Creatinine (Cr)	$1.02*10^{-3}$	$3.03*10^{-4}$	0.001	1.00 (1.00 ~ 1.00)	$2.84*10^{-4}$	$3.65*10^{-4}$	0.439	1.00 (1.00 ~ 1.00)
Platelet count (PLT)	$-1.12*10^{-3}$	$3.61*10^{-4}$	0.002	1.00 (1.00 ~ 1.00)	$-1.58*10^{-3}$	$3.86*10^{-4}$	<0.001	1.00 (1.00 ~ 1.00)
Time (day)	$2.27*10^{-2}$	$3.87*10^{-3}$	<0.001	1.02 (1.02 ~ 1.03)	$1.96*10^{-2}$	$3.96*10^{-3}$	<0.001	1.02 (1.01 ~ 1.03)

For covariate definitions and abbreviations, see the note to Tables S18–S37.

Table S36. Subgroup analysis stratified by immunocompromised status (immunocompromised vs. non-immunocompromised), Model 4 (ΔP)

	Immunocompetent				Immunocompromised			
**Variables**	***β***	***Se***	***P***	***HR* (95% *CI*)**	***β***	***Se***	***P***	***HR* (95% *CI*)**
Age	2.83*10^{-2}	3.41*10^{-3}	<0.001	1.03 (1.02 ~ 1.04)	1.15*10^{-2}	3.39*10^{-3}	0.001	1.01 (1.00 ~ 1.02)
Non-pulmonary SOFA score	7.51*10^{-2}	1.68*10^{-2}	<0.001	1.08 (1.04 ~ 1.11)	9.67*10^{-2}	1.72*10^{-2}	<0.001	1.10 (1.07 ~ 1.14)
Septic shock	-6.26*10^{-4}	1.11*10^{-1}	0.996	1.00 (0.80 ~ 1.24)	-7.31*10^{-2}	1.13*10^{-1}	0.516	0.93 (0.75 ~ 1.16)
Chronic lung disease	-2.41*10^{-2}	1.01*10^{-1}	0.812	0.98 (0.80 ~ 1.19)	1.56*10^{-1}	1.04*10^{-1}	0.136	1.17 (0.95 ~ 1.43)
Cardiac disease	1.22*10^{-1}	1.13*10^{-1}	0.279	1.13 (0.91 ~ 1.41)	2.33*10^{-3}	1.25*10^{-1}	0.985	1.00 (0.78 ~ 1.28)
Neurologic disease	-1.19*10^{-1}	1.16*10^{-1}	0.305	0.89 (0.71 ~ 1.11)	4.18*10^{-2}	1.19*10^{-1}	0.725	1.04 (0.83 ~ 1.32)
Liver disease	3.86*10^{-1}	1.96*10^{-1}	0.048	1.47 (1.00 ~ 2.16)	6.33*10^{-3}	2.51*10^{-1}	0.980	1.01 (0.61 ~ 1.65)
Chronic kidney disease	-3.59*10^{-1}	1.54*10^{-1}	0.020	0.70 (0.52 ~ 0.94)	-1.20*10^{-1}	1.49*10^{-1}	0.420	0.89 (0.66 ~ 1.19)
Bacterial infection	-1.65*10^{-1}	1.41*10^{-1}	0.241	0.85 (0.64 ~ 1.12)	-1.43*10^{-1}	1.41*10^{-1}	0.312	0.87 (0.66 ~ 1.14)
Viral infection	8.13*10^{-3}	1.15*10^{-1}	0.944	1.01 (0.80 ~ 1.26)	1.73*10^{-2}	1.02*10^{-1}	0.865	1.02 (0.83 ~ 1.24)
Fungal infection	2.27*10^{-1}	9.36*10^{-2}	0.015	1.26 (1.04 ~ 1.51)	1.24*10^{-1}	1.02*10^{-1}	0.225	1.13 (0.93 ~ 1.38)
Systemic glucocorticoid	3.54*10^{-1}	1.02*10^{-1}	0.001	1.43 (1.17 ~ 1.74)	2.67*10^{-1}	1.22*10^{-1}	0.028	1.31 (1.03 ~ 1.66)
Neuromuscular blocker	6.76*10^{-1}	1.10*10^{-1}	<0.001	1.97 (1.58 ~ 2.44)	2.28*10^{-1}	1.04*10^{-1}	0.029	1.26 (1.02 ~ 1.54)
Mean arterial pressure (MAP)	4.71*10^{-9}	4.71*10^{-10}	<0.001	1.00 (1.00 ~ 1.00)	1.27*10^{-4}	2.79*10^{-4}	0.650	1.00 (1.00 ~ 1.00)
Heart rate (HR)	8.02*10^{-3}	1.39*10^{-3}	<0.001	1.01 (1.01 ~ 1.01)	7.35*10^{-3}	1.13*10^{-3}	<0.001	1.01 (1.01 ~ 1.01)
Respiratory rate (RR)	1.51*10^{-3}	2.78*10^{-3}	0.588	1.00 (1.00 ~ 1.01)	3.09*10^{-3}	2.14*10^{-3}	0.154	1.00 (1.00 ~ 1.01)
PEEP	5.71*10^{-2}	9.88*10^{-3}	<0.001	1.06 (1.04 ~ 1.08)	2.33*10^{-2}	8.20*10^{-3}	0.005	1.02 (1.01 ~ 1.04)
Driving pressure (ΔP)	4.38*10^{-3}	4.88*10^{-3}	0.374	1.00 (0.99 ~ 1.01)	7.18*10^{-4}	1.83*10^{-3}	0.697	1.00 (1.00 ~ 1.00)
Fluid balance (input − output)	1.83*10^{-11}	1.83*10-12	<0.001	1.00 (1.00 ~ 1.00)	1.56*10^{-5}	2.01*10^{-5}	0.442	1.00 (1.00 ~ 1.00)
PaO_2/FiO_2 ratio	-1.00*10^{-3}	2.58*10^{-4}	<0.001	1.00 (1.00 ~ 1.00)	-1.43*10^{-3}	3.17*10^{-4}	<0.001	1.00 (1.00 ~ 1.00)
PaCO2	3.62*10^{-3}	2.66*10^{-3}	0.177	1.00 (1.00 ~ 1.01)	7.25*10^{-3}	2.71*10^{-3}	0.008	1.01 (1.00 ~ 1.01)

	Immunocompetent				Immunocompromised			
**Variables**	***β***	***Se***	***P***	***HR* (95% *CI*)**	***β***	***Se***	***P***	***HR* (95% *CI*)**
pH	-1.06	$3.61*10^{-1}$	0.007	0.35 (0.17 ~ 0.7)	$3.12*10^{-2}$	$3.56*10^{-1}$	0.930	1.03 (0.51 ~ 2.07)
Lactate	$1.00*10^{-1}$	$1.72*10^{-2}$	<0.001	1.11 (1.07 ~ 1.14)	$1.28*10^{-1}$	$1.74*10^{-2}$	<0.001	1.14 (1.10 ~ 1.18)
C-reactive protein (CRP)	$9.90*10^{-4}$	$3.31*10^{-4}$	0.004	1.00 (1.00 ~ 1.00)	$9.64*10^{-4}$	$3.53*10^{-4}$	0.010	1.00 (1.00 ~ 1.00)
Interleukin-6 (IL-6)	$1.30*10^{-4}$	$3.62*10^{-5}$	0.003	1.00 (1.00 ~ 1.00)	$1.21*10^{-4}$	$3.17*10^{-5}$	<0.001	1.00 (1.00 ~ 1.00)
Procalcitonin (PCT)	$-2.09*10^{-3}$	$2.32*10^{-3}$	0.370	1.00 (0.99 ~ 1.00)	$-1.62*10^{-3}$	$2.57*10^{-3}$	0.532	1.00 (0.99 ~ 1.00)
B-type natriuretic peptide (BNP)	$8.05*10^{-6}$	$3.77*10^{-6}$	0.033	1.00 (1.00 ~ 1.00)	$1.08*10^{-5}$	$3.97*10^{-6}$	0.007	1.00 (1.00 ~ 1.00)
Albumin	$-1.19*10^{-2}$	$5.40*10^{-3}$	0.028	0.99 (0.98 ~ 1.00)	$-7.11*10^{-3}$	$5.37*10^{-3}$	0.188	0.99 (0.98 ~ 1.00)
Absolute neutrophil count (ANC)	$1.92*10^{-2}$	$4.73*10^{-3}$	<0.001	1.02 (1.01 ~ 1.03)	$1.83*10^{-2}$	$4.91*10^{-3}$	<0.001	1.02 (1.01 ~ 1.03)
Absolute lymphocyte count (ALC)	$-1.18*10^{-1}$	$5.51*10^{-2}$	0.035	0.89 (0.80 ~ 0.99)	$-3.29*10^{-2}$	$3.53*10^{-2}$	0.351	0.97 (0.90 ~ 1.04)
Hemoglobin (Hb)	$-2.63*10^{-3}$	$1.61*10^{-3}$	0.102	1.00 (0.99 ~ 1.00)	$1.39*10^{-3}$	$1.55*10^{-3}$	0.371	1.00 (1.00 ~ 1.00)
D-dimer	$2.82*10^{-5}$	$1.23*10^{-4}$	0.821	1.00 (1.00 ~ 1.00)	$3.69*10^{-4}$	$3.40*10^{-3}$	0.913	1.00 (0.99 ~ 1.01)
Creatinine (Cr)	$1.07*10^{-3}$	$3.03*10^{-4}$	0.001	1.00 (1.00 ~ 1.00)	$3.56*10^{-4}$	$3.62*10^{-4}$	0.326	1.00 (1.00 ~ 1.00)
Platelet count (PLT)	$-1.12*10^{-3}$	$3.60*10^{-4}$	0.002	1.00 (1.00 ~ 1.00)	$-1.58*10^{-3}$	$3.86*10^{-4}$	<0.001	1.00 (1.00 ~ 1.00)
Time (day)	$2.13*10^{-2}$	$3.87*10^{-3}$	<0.001	1.02 (1.01 ~ 1.03)	$1.87*10^{-2}$	$3.74*10^{-3}$	<0.001	1.02 (1.01 ~ 1.03)

For covariate definitions and abbreviations, see the note to Tables S18–S37.

Table S37. Subgroup analysis stratified by immunocompromised status (immunocompromised vs. non-immunocompromised), Model 5 (4×ΔP+RR)

	Immunocompetent				Immunocompromised			
**Variables**	***β***	***Se***	***P***	***HR* (95% *CI*)**	***β***	***Se***	***P***	***HR* (95% *CI*)**
Age	$2.83*10^{-2}$	$3.41*10^{-3}$	<0.001	1.03 (1.02 ~ 1.04)	$1.15*10^{-2}$	$3.40*10^{-3}$	0.001	1.01 (1.00 ~ 1.02)
Non-pulmonary SOFA score	$7.52*10^{-2}$	$1.68*10^{-2}$	<0.001	1.08 (1.04 ~ 1.11)	$9.65*10^{-2}$	$1.72*10^{-2}$	<0.001	1.10 (1.06 ~ 1.14)
Septic shock	$-6.44*10^{-4}$	$1.11*10^{-1}$	0.995	1.00 (0.80 ~ 1.24)	$-7.19*10^{-2}$	$1.13*10^{-1}$	0.523	0.93 (0.75 ~ 1.16)
Chronic lung disease	$-2.64*10^{-2}$	$1.02*10^{-1}$	0.795	0.97 (0.80 ~ 1.19)	$1.56*10^{-1}$	$1.04*10^{-1}$	0.136	1.17 (0.95 ~ 1.43)
Cardiac disease	$1.22*10^{-1}$	$1.13*10^{-1}$	0.279	1.13 (0.91 ~ 1.41)	$-1.05*10^{-3}$	$1.25*10^{-1}$	0.993	1.00 (0.78 ~ 1.28)
Neurologic disease	$-1.19*10^{-1}$	$1.16*10^{-1}$	0.304	0.89 (0.71 ~ 1.11)	$3.92*10^{-2}$	$1.19*10^{-1}$	0.742	1.04 (0.82 ~ 1.31)
Liver disease	$3.83*10^{-1}$	$1.96*10^{-1}$	0.051	1.47 (1.00 ~ 2.16)	$7.92*10^{-3}$	$2.51*10^{-1}$	0.975	1.01 (0.62 ~ 1.65)
Chronic kidney disease	$-3.58*10^{-1}$	$1.54*10^{-1}$	0.020	0.70 (0.52 ~ 0.95)	$-1.21*10^{-1}$	$1.49*10^{-1}$	0.417	0.89 (0.66 ~ 1.19)
Bacterial infection	$-1.65*10^{-1}$	$1.40*10^{-1}$	0.240	0.85 (0.64 ~ 1.12)	$-1.41*10^{-1}$	$1.41*10^{-1}$	0.316	0.87 (0.66 ~ 1.14)
Viral infection	$7.72*10^{-3}$	$1.15*10^{-1}$	0.947	1.01 (0.80 ~ 1.26)	$1.82*10^{-2}$	$1.02*10^{-1}$	0.859	1.02 (0.83 ~ 1.24)
Fungal infection	$2.27*10^{-1}$	$9.35*10^{-2}$	0.015	1.25 (1.04 ~ 1.51)	$1.23*10^{-1}$	$1.02*10^{-1}$	0.230	1.13 (0.93 ~ 1.38)
Systemic glucocorticoid	$3.53*10^{-1}$	$1.02*10^{-1}$	0.001	1.42 (1.17 ~ 1.74)	$2.67*10^{-1}$	$1.22*10^{-1}$	0.028	1.31 (1.03 ~ 1.66)
Neuromuscular blocker	$6.76*10^{-1}$	$1.10*10^{-1}$	<0.001	1.97 (1.59 ~ 2.44)	$2.32*10^{-1}$	$1.04*10^{-1}$	0.025	1.26 (1.03 ~ 1.55)
Mean arterial pressure (MAP)	$4.71*10^{-9}$	$4.72*10^{-10}$	<0.001	1.00 (1.00 ~ 1.00)	$1.31*10^{-4}$	$2.67*10^{-4}$	0.624	1.00 (1.00 ~ 1.00)
Heart rate (HR)	$8.02*10^{-3}$	$1.39*10^{-3}$	<0.001	1.01 (1.01 ~ 1.01)	$7.39*10^{-3}$	$1.13*10^{-3}$	<0.001	1.01 (1.01 ~ 1.01)
PEEP	$5.70*10^{-2}$	$9.89*10^{-3}$	<0.001	1.06 (1.04 ~ 1.08)	$2.32*10^{-2}$	$8.21*10^{-3}$	0.005	1.02 (1.01 ~ 1.04)
[(4×ΔP)+RR]	$1.06*10^{-3}$	$9.35*10^{-4}$	0.293	1.00 (1.00 ~ 1.00)	$6.07*10^{-4}$	$4.78*10^{-4}$	0.229	1.00 (1.00 ~ 1.00)
Fluid balance (input − output)	$1.84*10^{-11}$	1.83*10-12	<0.001	1.00 (1.00 ~ 1.00)	$1.53*10^{-5}$	$2.01*10^{-5}$	0.449	1.00 (1.00 ~ 1.00)
PaO_2/FiO_2 ratio	$-1.00*10^{-3}$	$2.59*10^{-4}$	<0.001	1.00 (1.00 ~ 1.00)	$-1.45*10^{-3}$	$3.17*10^{-4}$	<0.001	1.00 (1.00 ~ 1.00)
PaCO2	$3.53*10^{-3}$	$2.60*10^{-3}$	0.178	1.00 (1.00 ~ 1.01)	$7.23*10^{-3}$	$2.72*10^{-3}$	0.008	1.01 (1.00 ~ 1.01)
pH	-1.07	$3.57*10^{-1}$	0.005	0.34 (0.17 ~ 0.69)	$3.09*10^{-2}$	$3.58*10^{-1}$	0.931	1.03 (0.51 ~ 2.08)

	Immunocompetent				Immunocompromised			
**Variables**	***β***	***Se***	***P***	***HR* (95% *CI*)**	***β***	***Se***	***P***	***HR* (95% *CI*)**
Lactate	$1.00*10^{-1}$	$1.72*10^{-2}$	<0.001	1.11 (1.07 ~ 1.14)	$1.28*10^{-1}$	$1.73*10^{-2}$	<0.001	1.14 (1.10 ~ 1.18)
C-reactive protein (CRP)	$9.94*10^{-4}$	$3.32*10^{-4}$	0.004	1.00 (1.00 ~ 1.00)	$9.69*10^{-4}$	$3.52*10^{-4}$	0.009	1.00 (1.00 ~ 1.00)
Interleukin-6 (IL-6)	$1.30*10^{-4}$	$3.64*10^{-5}$	0.003	1.00 (1.00 ~ 1.00)	$1.22*10^{-4}$	$3.16*10^{-5}$	<0.001	1.00 (1.00 ~ 1.00)
Procalcitonin (PCT)	$-2.16*10^{-3}$	$2.29*10^{-3}$	0.348	1.00 (0.99 ~ 1.00)	$-1.58*10^{-3}$	$2.58*10^{-3}$	0.542	1.00 (0.99 ~ 1.00)
B-type natriuretic peptide (BNP)	$8.00*10^{-6}$	$3.78*10^{-6}$	0.035	1.00 (1.00 ~ 1.00)	$1.09*10^{-5}$	$3.98*10^{-6}$	0.007	1.00 (1.00 ~ 1.00)
Albumin	$-1.20*10^{-2}$	$5.40*10^{-3}$	0.027	0.99 (0.98 ~ 1.00)	$-7.04*10^{-3}$	$5.37*10^{-3}$	0.192	0.99 (0.98 ~ 1.00)
Absolute neutrophil count (ANC)	$1.92*10^{-2}$	$4.73*10^{-3}$	<0.001	1.02 (1.01 ~ 1.03)	$1.83*10^{-2}$	$4.91*10^{-3}$	<0.001	1.02 (1.01 ~ 1.03)
Absolute lymphocyte count (ALC)	$-1.18*10^{-1}$	$5.52*10^{-2}$	0.036	0.89 (0.80 ~ 0.99)	$-3.36*10^{-2}$	$3.55*10^{-2}$	0.344	0.97 (0.90 ~ 1.04)
Hemoglobin (Hb)	$-2.64*10^{-3}$	$1.61*10^{-3}$	0.101	1.00 (0.99 ~ 1.00)	$1.31*10^{-3}$	$1.55*10^{-3}$	0.396	1.00 (1.00 ~ 1.00)
D-dimer	$2.75*10^{-5}$	$1.23*10^{-4}$	0.825	1.00 (1.00 ~ 1.00)	$3.08*10^{-4}$	$3.40*10^{-3}$	0.928	1.00 (0.99 ~ 1.01)
Creatinine (Cr)	$1.07*10^{-3}$	$3.04*10^{-4}$	0.001	1.00 (1.00 ~ 1.00)	$3.51*10^{-4}$	$3.62*10^{-4}$	0.332	1.00 (1.00 ~ 1.00)
Platelet count (PLT)	$-1.11*10^{-3}$	$3.61*10^{-4}$	0.002	1.00 (1.00 ~ 1.00)	$-1.58*10^{-3}$	$3.87*10^{-4}$	<0.001	1.00 (1.00 ~ 1.00)
Time (day)	$2.16*10^{-2}$	$3.88*10^{-3}$	<0.001	1.02 (1.01 ~ 1.03)	$1.91*10^{-2}$	$3.77*10^{-3}$	<0.001	1.02 (1.01 ~ 1.03)

For covariate definitions and abbreviations, see the note to Tables S18–S37.
